# An iterative compound screening contest method for identifying target protein inhibitors using the tyrosine-protein kinase Yes

**DOI:** 10.1038/s41598-017-10275-4

**Published:** 2017-09-20

**Authors:** Shuntaro Chiba, Takashi Ishida, Kazuyoshi Ikeda, Masahiro Mochizuki, Reiji Teramoto, Y-h. Taguchi, Mitsuo Iwadate, Hideaki Umeyama, Chandrasekaran Ramakrishnan, A. Mary Thangakani, D. Velmurugan, M. Michael Gromiha, Tatsuya Okuno, Koya Kato, Shintaro Minami, George Chikenji, Shogo D. Suzuki, Keisuke Yanagisawa, Woong-Hee Shin, Daisuke Kihara, Kazuki Z. Yamamoto, Yoshitaka Moriwaki, Nobuaki Yasuo, Ryunosuke Yoshino, Sergey Zozulya, Petro Borysko, Roman Stavniichuk, Teruki Honma, Takatsugu Hirokawa, Yutaka Akiyama, Masakazu Sekijima

**Affiliations:** 1 Advanced Computational Drug Discovery Unit, Institute of Innovative Research, Tokyo Institute of Technology, J3-23 4259 Nagatsuta-cho, Midori-ku, Yokohama, 226-8501 Japan; 2 Education Academy of Computational Life Sciences, Tokyo Institute of Technology, J3-141 4259 Nagatsuta-cho, Midori-ku, Yokohama, 226-8501 Japan; 3 Department of Computer Science, Tokyo Institute of Technology, 2-12-1, Ookayama, Meguro-ku, Tokyo, 152-8550 Japan; 4 Level Five Co. Ltd., Shiodome Shibarikyu Bldg., 1-2-3 Kaigan, Minato-ku, Tokyo, 105-0022 Japan; 5 IMSBIO Co., Ltd., Level 6 OWL TOWER, 4-21-1 Higashi-Ikebukuro, Toshima-ku, Tokyo, 170-0013 Japan; 6 Forerunner Pharma Research, Co., Ltd., Yokohama Bio Industry Center, 1-6 Suehiro-cho, Tsurumi-ku, Yokohama, 230-0045 Japan; 7 Department of Physics, Chuo University, 1-13-27 Kasuga, Bunkyo-ku, Tokyo 112-8551 Japan; 8 Department of Biological Sciences, Chuo University, 1-13-27 Kasuga, Bunkyo-ku, Tokyo 112-8551 Japan; 9 Department of Biotechnology, Bhupat Jyoti Mehta School of Biosciences, Indian Institute of Technology Madras, Chennai, 600036 Tamilnadu India; 10 CAS in Crystallography and Biophysics and Bioinformatics Facility, University of Madras, Chennai, 600025 Tamilnadu India; 11 Division of Neurogenetics, Nagoya University Graduate School of Medicine, 65 Tsurumai, Showa-ku, Nagoya, 466-8550 Japan; 12 Department of Computational Science and Engineering, Nagoya University, Furocho, Chikusa, Nagoya, 464-8603 Japan; 13 Department of Complex Systems Science, Graduate School of Information Science, Nagoya University, Furocho, Chikusa, Nagoya, 464-8601 Japan; 14 Department of Biological Sciences, Purdue University, Indiana, 47907 USA; 15 Department of Computer Science, Purdue University, Indiana, 47907 USA; 16 Isotope Science Center, The University of Tokyo, 2-11- 16, Yayoi, Bunkyo-ku, Tokyo, 113-0032 Japan; 17 Department of Biotechnology, The University of Tokyo, 1-1-1 Yayoi, Bunkyo-ku, Tokyo, 113-8657 Japan; 18 Global Scientific Information and Computing Center, Tokyo Institute of Technology, 2-12-1, Ookayama, Meguro-ku, Tokyo, 152-8550 Japan; 19 Bienta/Enamine Ltd., 78 Chervonotkatska Street, Kyiv, 02660 Ukraine; 20 National Taras Shevchenko University of Kyiv, 64/13 Volodymyrska Street, Kyiv, 01601 Ukraine; 21 Center for Life Science Technologies, RIKEN, 1-7-22 Suehiro, Tsurumi, Yokohama, Kanagawa 230-0045 Japan; 22 Molecular Profiling Research Center for Drug Discovery, National Institute of Advanced Industrial Science and Technology, 2-4-7 Aomi, Koto-ku, Tokyo, 135-0064 Japan; 23 Division of Biomedical Science, Faculty of Medicine, University of Tsukuba, 1-1-1 Tennodai, Tsukuba-shi, Ibaraki, 305-8575 Japan; 24 Initiative for Parallel Bioinformatics, Level 14 Hibiya Central Building, 1-2-9 Nishi-Shimbashi Minato-Ku, Tokyo, 105-0003 Japan

## Abstract

We propose a new iterative screening contest method to identify target protein inhibitors. After conducting a compound screening contest in 2014, we report results acquired from a contest held in 2015 in this study. Our aims were to identify target enzyme inhibitors and to benchmark a variety of computer-aided drug discovery methods under identical experimental conditions. In both contests, we employed the tyrosine-protein kinase Yes as an example target protein. Participating groups virtually screened possible inhibitors from a library containing 2.4 million compounds. Compounds were ranked based on functional scores obtained using their respective methods, and the top 181 compounds from each group were selected. Our results from the 2015 contest show an improved hit rate when compared to results from the 2014 contest. In addition, we have successfully identified a statistically-warranted method for identifying target inhibitors. Quantitative analysis of the most successful method gave additional insights into important characteristics of the method used.

## Introduction

Introducing a new drug to a market has become an enormous undertaking because of expanding research and development costs, which are estimated at over one billion USD^1–4^. With a view to reducing these costs, computational technology-driven approaches have been proven to be useful and have begun to be applied at various stages of the drug discovery campaign, including from target identification to clinical phases^3, 5^. For these stages, including the hit-compound identification for a target molecule, many computational methods have been devised to find compounds that are active from a compound library without resorting to high-throughput screening.

These computational methods use various approaches and experimental information; however, they are often divided into two categories: structure-based (SB) and ligand-based (LB). SB methods use an atomic-level structure of a target molecule. Most typical SB methods are molecular docking approaches that search the complex structure of a ligand, included in a compound library, and a target-molecule structure based on a scoring function. A ranking of docked compounds is calculated using these scores^6^. In contrast, LB methods use information of known active and/or inactive compounds related to a target molecule. LB methods generally calculate a ranking of compounds in a library using techniques such as a similarity search and machine learning^7^. Currently, various methods based on both SB and LB algorithms have been proposed for identifying hit compounds^6–8^.

Although these methods are reasonably designed and seem to have the ability to enrich potent compounds toward higher ranks from a compound library, there are no set standards because the performance of a method often depends on the target molecule^9^. Hence, we cannot choose a method suitable for a specific target molecule before conducting experimental assessments. Thus, designating all resources for one method is risky. However, this risk may be reduced by collecting data from various computational methods. In addition, after conducting experimental assays, we can obtain information regarding a suitable method for the target.

To evaluate various methods for a target molecule, we held a compound-screening contest in 2014 to find inhibitors of the tyrosine-protein kinase Yes as an example target from a 2.2-million-compound library^10^. Ten groups participated in the contest and, in total, 600 compound-inhibition rates for enzymatic activity were assayed. We showed that the connected diversity of compounds proposed from all participant groups was larger than that proposed by any single group. This enabled the diversified screening of the compound library with reasonable methods. As a result, two compounds were identified as hit compounds. We had speculated that we could find methods that were significantly more likely to provide hit compounds than others based on the contest’s results. However, this was not possible with a statistically significant measure because of the shortage in number of assayed compounds. In the previous contest, the most successful group found 2 hit compounds from 55 compounds assayed. Provided that an average hit rate was 2/600, the *p*-value calculated by the binomial test for the group was 0.015. Taking the problem of multiple comparisons into account by the Bonferroni correction, there were no methods that outperformed others. In addition, the experiment may fail to detect other good methods, because, even if a method has a 3% hit-rate potential, 18.7% of the trials would return 0 hit compounds with 55 assays. Thus, many more assays are required for reliable evaluation.

To evaluate our approach for collecting various methods to reduce the risk of allocating all resources towards one method, and for obtaining useful information regarding promising methods, we conducted another contest in this study. We increased the number of compounds to be assayed for each group to more than 180. We chose the same target molecule as in the previous contest, i.e., the tyrosine-protein kinase Yes, because participants could use protein structural information as well as active and inactive compound information for this target, as well as related kinases in the same family. While the structure of Yes has not been reported, many homologous protein structures are deposited in the Protein Data Bank (PDB)^11^ (e.g., 1Y57 (Unphosphorylated state of the tyrosine-protein kinase Src. Positives to Yes=92%)^12^, 2SRC (Phosphorylated state of the tyrosine-protein kinase Src, positives=92%)^13^, 1OPK (the tyrosine-protein kinase Abl, positives=63%))^14^. Experimental information from active and inactive compounds for the target are deposited in open databases, such as BindingDB^15, 16^, ChEMBL^17^, DrugBank^18^, and PubChem^19^.

**Figure 1:**
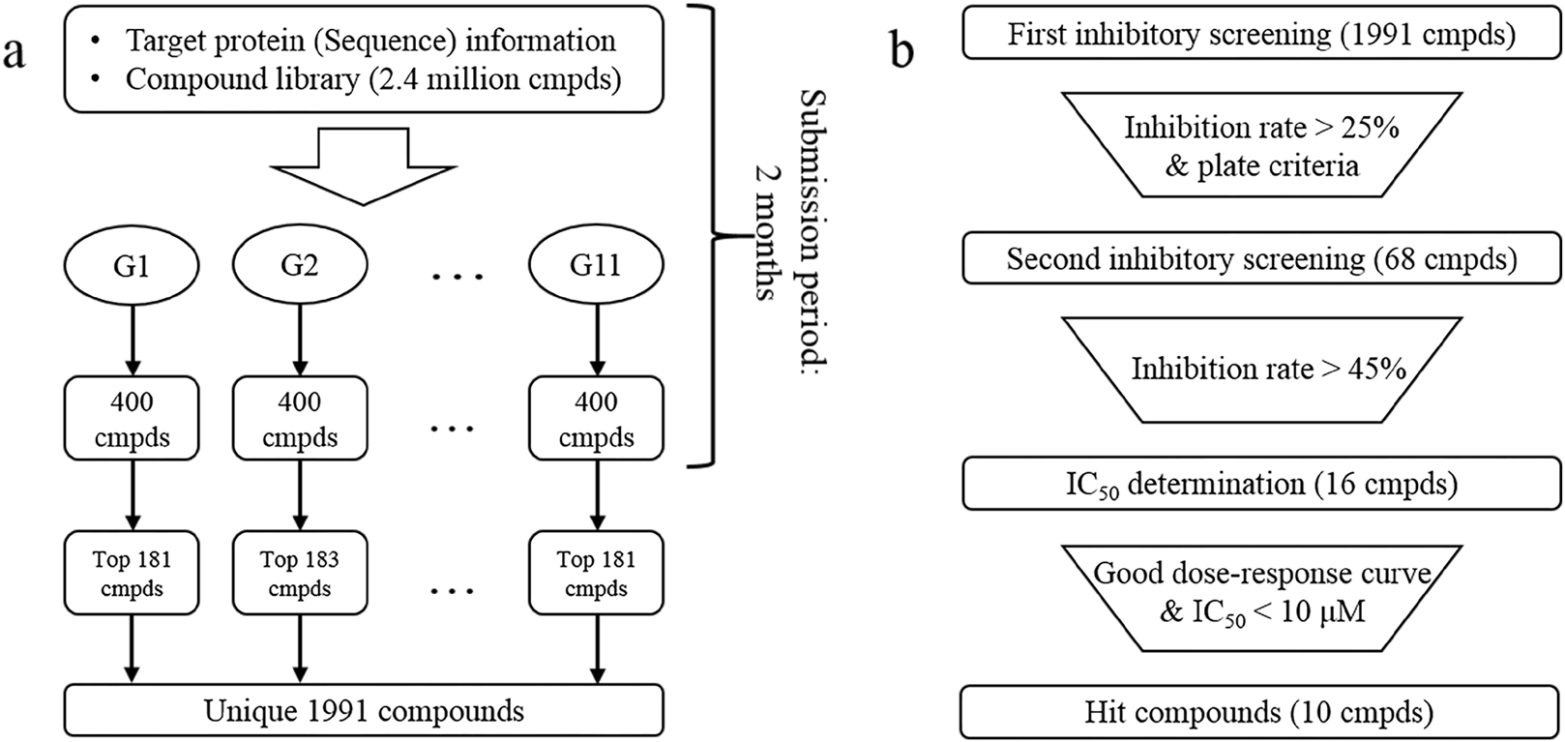
(**a**) The flowchart of the contest. The participated groups (G1–G11) proposed 400 compounds (cmpds) with a prioritized rank from compound library using their own methods. The proposed compounds that were not stocked-out were selected until the number of compounds reached 181 for each group. If there is a duplication in the proposed compounds from different groups, such group attained additional compounds to be assayed. This is the reason why there are differences among the number of selected compounds of each group. Finally, the selected compounds were assayed. (**b**) The screening flow of the compounds in the experimental assay. The filtering criteria are shown in a trapezium.

The compound screening contest was organized by the Initiative for Parallel Bioinformatics (IPAB). It started on January 15, 2015 and ended on March 20, 2015. Eleven groups participated in the contest. The participants were asked to propose a prioritized set of 400 compounds. We selected approximately top 180 compounds from the prioritized list from each group and, in total, 1,991 unique compounds were assayed. Ten potent compounds with half-maximal inhibitory concentrations (IC_50_) less than 10 μmol L^−1^ were identified. Overview of the procedure is shown in Fig. 1. Among the 11 methods, a successful method was identified for this target in terms of hit rate, and the salient features of this method are discussed.

## Methods

### Preparation of compound library

A compound library was originally provided by Enamine Ltd. and contained 2,382,017 of the available compounds in their inventory. We searched the known inhibitors of Src-family kinases shown in Table S1 (Supporting Information) that met with certain criteria from ChEMBL (version 19)^20^ and BindingDB^15, 16^ to eliminate them from the original library. These criteria included compounds with IC_50_ <10 μmol L^−1^, *K*_i_ <10 μmol L^−1^, *K*_d_ <10 μmol L^−1^, and inhibition rates >30%, where we did not take experimental conditions into consideration. We found 3,528 unique compounds, hereafter referred to as the known inhibitors of the contest, among which 24 compounds were identified and eliminated from the original compound library. We also excluded compounds interacting with a number of proteins. We searched compounds that inhibit more than four proteins from ChEMBL, using the same inhibition criteria, and 5009 compounds were identified. This number was reduced to 245 compounds by filtering for drug-likeness, as defined in Table S2 (Supporting Information). From there, 54 compounds were identified and eliminated from the original library. Finally, the processed library contained 2,381,939 compounds, and it was distributed to participants of the contest. All compound IDs in this study correspond to Enamine Ltd. IDs.

### Methods participated

We accepted 11 groups, which are referred to as G1−G11 hereafter, which proposed various methods (shown in Table 1). A detailed description from groups and proposed compounds of SMILES in prioritized order are given in the section *Methods used by each group* of the supporting information and supplemental materials. Here, we briefly describe each method.

**Table 1:** Summary of methods used by participant groups.

Group	Modeling of Yes structure		Ligand preparation	Processing method of compound library		
	**3D structure prediction methods/tools**	**Template PDB ID**		**Filter class**	**Actives**	**Inactive**
1	—	—	—	LB: 1D and 2D PaDEL descriptor^49^	The top 7 compounds in PubChem (AID 686947)^22^	The rest of compounds
2	—	—	—	LB: Morgan descriptor^50^	80% of PubChem (AID 686947)^22^ (The rest was used to validate the model built.)	
3	—	—	—	LB: Morgan2^50^ and atom pairs descriptors^51^ Protein: ProtFP^52^ and Z-scales^53^ Experimental conditions	Eliminated.sdf.zip,^*a*^ PubChem (see details in Table S6 of the Supporting Info.) & IPAB2014^*b*^	
4	Homology modeling (*FAMS*)^54^	1Y57^12^	*Open Babel* ^55^	SB: *ChooseLD* ^25^		
5	Homology modeling (*MODELLER*)^27^	1OPK^14^, 1IEP^26^	*LigPrep* ^56^	Hybrid (LB & SB): *Glide* ^28, 57–59^ and pharmacophore-based screening^29, 30^	IPAB2014^*b*^	IPAB2014^*b*^
6	Homology modeling (*MODELLER*)^27^	2SRC^13^	*OMEGA* ^60^	Hybrid (LB & SB): *VS-APPLE* ^31^	Eliminated.sdf.zip^*a*^	DUD-E^9^
7	—	—	—	LB: Physicochemical properties and topological descriptors complied in *Canvas* ^46, 47^	Eliminated.sdf.zip^*a*^	IPAB2014^*b*^
8	Homology modeling (*GalaxyTBM*)^61^	2H8H^62^, 1KSW^63^, 1FMK^64^	*OMEGA* ^60^	SB: *PL-PatchSurfer2* (primitive version)^65, 66^	—	—
9	Homologous protein structure themselves were used.	1YI6^67^, 3G5D^68^,	*OMEGA* ^60^	Hybrid (LB → SB) LB: Drug-like filtering (*SYBYL-X 2.0*), SB: *OEDocking* ^69–71^		
10	Homology modeling (*MODELLER* in *HHpred* ^72^, *PSIPRED*)^73^, followed by MD simulation^74–76^ (*GROMACS*)	2H8H^62^	*OMEGA* ^60^	Hybrid (LB → SB) LB: *ROCS* (ligand-shape-based method)^77^ SB: *Molegro Virtual Docker* ^78^	List 1–3 for Ligand-based filtering (See Section *Methods used by each group* in the Supporting Info.)	—
11	Homology modeling (*Prime* ^1, 2^)	1Y57^12^	*LigPrep* ^56^	SB: *Glide* ^57–59^, followed by filtering based on conserved binding modes of docking poses	Actives (IC_50_ <1 μM) in ChEMBL, IPAB2014^*b*^	300 compounds from IPAB2014^*b*^

Software names are given in italics.

^*a*^Known Src-kinase inhibitors distributed by IPAB (see Preparation of compound library section).

^*b*^Inhibitory assay results of the previous contest^10^, in which experimental conditions were the same as this study.

PDB = protein data bank; LB = ligand-based; SB = structure-based; IPAB = Initiative for Parallel Bioinformatics; MD = molecular dynamics;

G1: A structure-activity-relationship (SAR) model was built employing balanced random forests^21^. Ligand descriptors of PubChem bioactive data^22^ for Yes kinase were used as the training set, in which seven compounds with IC_50_ <1 nmol L^−1^ were selected as active compounds and the other 832 compounds were designated as inactive.

G2: An SAR model was built employing a deep neural network model, in which descriptors of randomly-chosen 80% of the PubChem bioactive data^22^ were used as a training set and the other 20% comprised the test set, each of which contained active and non-active compounds. Promising compounds based on the SAR model were selected, followed by a filtering of drug-likeness and diverse selection.

G3: Compounds that were physicochemically similar to those of known inhibitors were filtered using a modified QED^23^. A randomized tree model^24^ was built on the bases of the concatenated descriptors of known inhibitors, their target kinases, and experimental conditions (concentration of reagents) and was applied to filter compounds. Out-of-bag validation showed a good correlation between predicted and experimental values. The filtered compounds were re-ranked by three metrics: (1) the original ranking, (2) prioritized by ligand efficiency based on the number of heavy atoms, and (3) the novelty of compounds to the top 1,000 of the original ranking compared with Src-family inhibitors. The proposed compounds were rotationally picked up from the three ranks.

G4: The Yes protein structure was built using BLAST search with the Yes sequence. Homologous proteins having a ligand of the Yes sequence were searched and the bound ligands were remapped to the built protein, which was used for the docking^25^ of known inhibitors considering remapped ligands. Based on the ability to pick up inhibitors, Yes and ligand pairs were selected. These structures were used for the docking of library compounds.

G5: The crystal structures of Abl kinase, available for both IN and OUT conformations^14, 26^, were taken as templates and respective structures were built for Yes kinase^27^. PD166326, a type I inhibitor (IN), and imatinib, a type II inhibitor (OUT) were co-crystallized with Abl kinase and docked with the IN and OUT models built for Yes kinase. On the basis of physicochemical properties, the initial compound library was filtered. Actives and decoys^10^ were added to the filtered compounds and subjected to docking^28^ combined with pharmacophore-based virtual screening^29, 30^. The same set of actives and decoys were included to validate the screening results. Finally, the top hit compounds from the pharmacophore-based virtual screening of DFG-IN and DFG-OUT conformations were applied.

G6: A virtual screening method^31^ was applied to the compound library that performed 3D structural comparison based on a multiple-ligand template built from known multiple inhibitors using a geometric hashing technique. If a steric clash between a compound and the target protein was found, a score for a given ligand pose was penalized. Twenty complex structures of homologous proteins of Yes and its ligands deposited in the PDB were selected on the basis of the ability to discriminate actives from decoys through docking. The selected 20 proteins and their bound ligands were superimposed by the protein structure alignment program MICAN^32, 33^ to the Yes structure model built based on the closest homology of Yes^27^.

G7: A deep neural network was trained based on physicochemical and topological descriptors of active and inactive ligands. Hyperparameters of the deep neural network (e.g., a number of hidden layers) were also optimized using a random search^34^ based on receiver-operating-characteristic (ROC) curves calculated using 5-fold cross-validation procedures in terms of known ligands. The model that gave the best ROC curve was applied to filter the compound library.

G8: The target protein structure was built from homologous proteins and its binding pocket was converted into three-dimensional Zernike descriptors (3DZD). Ligand structures from the compound library were also converted to the 3DZD and the compatibility of each ligand to the pocket was used to select a potential inhibitor.

G9: Homologous proteins of Yes were downloaded from the PDB and docking pockets that were distant from the ATP/substrate-binding pockets were searched to find allosteric sites. Among the prepared candidate structures, two structures that showed higher docking scores from a relatively small number of compounds were chosen for the production run. Docked compounds were prepared by filtering similar known inhibitors (85% similarity) from the compound library. Visual inspection was applied to eliminate compounds that did not have drug-likeness.

G10: First, known potent compounds were used to filter the compound library to be used for subsequent docking. The Yes protein and ligand complex structure were built by homology modeling, followed by a molecular dynamics (MD) simulation of the complex to relax the structure. The 40-ns structure of the complex was used for docking.

G11: Protein ligand complex structures were built from three homologous proteins. Docking of active and inactive compounds was applied to each structure and the ability to separate active from inactive compounds was evaluated. Those displaying reasonable ability were used for docking of the compound library. Docking poses of high-ranked compounds were re-ranked using scores that considered the similarity and dissimilarity of docking poses among active and inactive compounds.

## Screening of Compounds

### Experimental procedure and screening of potential inhibitors

All inhibitory assays of the phosphorylation activity of Yes were performed in accordance with the Promega Technical Manual for the ADP-Glo™ Kinase Assay (Fitchburg, WI, USA. Catalog number: V9102). The human recombinant Yes [a.a. 2–543 (end)] was purchased from BPS Bioscience (catalog number: 40488). The details of the assay protocol and reagent information are given elsewhere^10^. Here, we briefly describe the screening of the compounds based on inhibition activity and results.

The screening was conducted in three steps consisting of the first screening, the second screening, and the IC_50_ determination, as can be illustrated in Fig. 1b. First, we determined the inhibition rates of the 1,991 compounds. Each compound was placed in 4 wells of a 384-well plate. In total, 80 compounds were assayed on one plate and the other wells were used for positive and negative controls. The compounds were randomly placed on plates so that compounds proposed by one group were not placed on a plate together. A mean of four inhibition rates for each compound was compared to criteria for the first screening. These criteria included that the inhibition rate was greater than 25% and the inhibition rate was greater than the mean plus three-fold of the standard deviation of the plate on which the compound was assayed, where observed inhibition rates of positive and negative controls were not taken into consideration. As a results, 68 compounds passed the screening. Information of the dropped compounds is given in Table S3 of the Supporting Information. Second, the inhibition rates of the screened compounds were determined on one plate using the same procedure of the first screening, where compounds were dissolved from fresh powder. As a result, 16 compounds showed inhibition rates greater than the threshold of the second screening (i.e., approximately 50%). Information for screened and dropped compounds is given in Table S4 of the Supporting Information. Screened compounds were then evaluated for their IC_50_ values. The chemical structure and assay results of these compounds are given in Table 2.

**Table 2:** IC_50_ values of compounds that passed the validation assay (the 2^nd^ screening).

Compound ID	Chemical Structure	IC_50_ μM	95% CI μM^*a*^		Group
			lower	upper	
Z64663950		0.26	0.22	0.31	3
Z49895016^*d*^		0.30	0.23	0.38	3
Z64663944		0.35	0.13	0.99	3
Z1229984790		0.71	0.24	2.10	10
Z57745314^*d*^		1.16	0.51	2.62	3
Z57745304^*d*^		1.9	1.5	2.4	3
Z199512484		3.0	1.9	4.7	3
Z410927360		3.4	3.2	3.6	10
Z295464022^*d*^		5.0	3.5	7.3	3
Z449737600^*d*^		7.0	5.2	9.3	11
Z1252403274^*b*^		20.0	15.6	25.6	11
Z275023406^*b*^		37.4	16.9	82.5	5
Z57745307^*cd*^		—	—	—	3
Z50080378^*cd*^		—	—	—	3
Z1283491630^*c*^		—	—	—	5
Z50080181^*cd*^		—	—	—	3

Inhibition rates from the first and second screenings are shown in Tables S1 and S2 of the Supporting Information along with the canonical SMILES. The final reagent concentrations were 5.5-nmol L^−1^ Yes, 0.013-mmol L^−1^ ATP, and 0.2-mg mL^−1^ substrate (poly Glu-Tyr peptides, Glu:Tyr=4:1).

(*a*) 95% confidence interval. Some compounds are not a hit because of insufficient potency (*b*) or a bad dose-dependence relationship (*c*). (*d*) These compounds are hydrazones or a potential Michel acceptor (see sections “Experimental procedure and screening of potential inhibitors” and “Comparison of ligand-based and structure-based methods”).

IC_50_ = inhibitory concentrations; CI = confidence interval.

Among the 16 compounds, 10 compounds showed an IC_50_ less than our hit criterion, which was an IC_50_ less than 10 μmol L^−1^, as shown in Table 2. These compounds showed a clear dose-response relationship (DRR) as can be seen in Figure S1 of the Supporting Information. As for Z1252403274 and Z275023406, which showed a good DRR, they were not defined as hit compounds because of insufficient potency. The other four compounds, Z50080378, Z57745307, Z50080181, and Z1283491630, did not reveal a DRR, having “inhibition activity” around 50% in the whole range of concentrations, which may be due to their non-specific interactions with the target (promiscuous protein binding, protein aggregation) or solubility-related issues. For these reasons, these compounds were excluded from consideration. Note that we confirmed that the threshold used for the second screening was reasonable as can be seen in Figure S2 of the Supporting Information.

The 10 hit compounds were compared to the pan-assay interference compounds (PAINS) filters, filters A, B, and C described in the literature^35^, which suggests potential functional groups of frequent hitters extracted from HTS assays. We found that the 10 compounds do not have these potential functional groups. This means that all the hit compounds are promising for further investigation. It should be noted that some hit compounds have “questionable” chemotypes from a medicinal chemistry point of view, i.e., hydrazones (Z49895016, Z57745314, Z57745304, Z295464022) and a potential Michael acceptor (Z449737600), which may present a reactivity/toxicity liability. In the present study, we did not exclude them because only the biochemical assays, not cell-based, were used for screening in this study. This strongly decreases the chance of getting false positives with these compounds during the primary screen. The emphasis was also placed on avoiding the potential loss of any active scaffolds identified by the competing computational groups, rather than on the early elimination of less desirable chemical series. Substituting hydrazones with their non-reactive isosteres during hit-to-lead optimization is a feasible medicinal chemistry endeavor as is illustrated by some research publications^36–38^. We will discuss these hydrazone-containing compounds in more details in the following section. The Michael acceptor could be substituted by an amide group between an acid and a cyclical secondary amine, to retain molecular rigidity.

## Discussion

### Hit rate of assayed compounds

**Figure 2:**
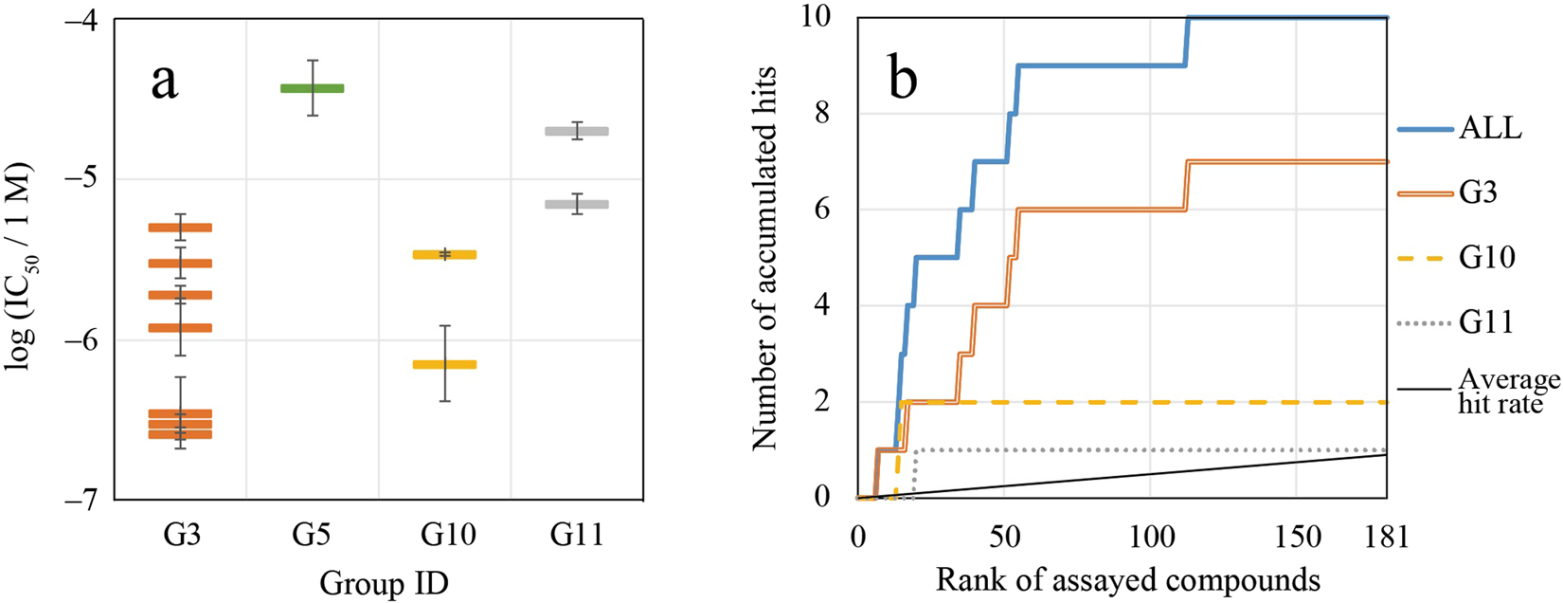
(**a**) The IC_50_ of compounds from each group, where results of those groups whose compounds did not proceed to the IC_50_ analysis are omitted. The compounds of log (IC_50_/1 M) less than −5 are hit compounds. The error bars represent a 68% confidence interval estimated from the IC_50_ assay. (**b**) The number of hit compounds included within a prioritized rank of compounds that were proposed from each group.

The total number of hit compounds was 10 (Table 2), seven of which were proposed by G3, two by G10, and one by G11. G3 outperformed the other groups in terms of the number of hits and potencies of the compounds and was followed by G10 and G11 as can be clearly seen in Fig. 2a. Performances of these methods in terms of a hit rate, compared to an average rate of all the methods, can be evaluated by the binomial test while eliminating the problem of multiple comparisons by applying the Bonferroni correction. Assuming the hit rate of all the compounds is 10/1991, the *p*-values for G3, G10, and G11 were 4 × 10^−5^, 0.2, and 0.6, respectively. Hence, we confirmed that the method of G3 was statistically warranted.

We can also evaluate these methods by how they enriched active compounds in their prioritized ranks. The hit compounds of these methods were enriched toward higher ranks as can be seen in Fig. 2b. These methods showed better enrichment compared to the average rate.

These results suggest that the methods employed by these groups could reasonably distinguish active compounds. In the present study, we will mainly focus on these three methods.

### Comparison of ligand-based and structure-based methods

The proposed methods were classified into LB and SB approaches. The LB approaches were defined as those methods that used active and/or inactive ligand information for relevant kinases regardless of the incorporation of protein structure. The SB approaches included methods that used protein structure and did not use ligand information for filtering of the compound library.

#### Hit rate

The groups that found hit compounds, G3, G10, and G11, can be classified into LB, LB→SB, and SB methods as tabulated in Table 1. G3 and G10 used ligand information in a direct way to filter the compound library; only G11 used ligand information in an indirect way, i.e., the selection of a protein structure used for docking. In this sense, it was only a single compound that was proposed by an SB method. Hence, compared to LB methods, it was very difficult to find a hit compound using an SB method in this study.

The proposed compounds from G3 were selected based on the three prioritized ranks (see the explanation of G3 in the section Methods participated). Four and three of the hit compounds of G3 were found by the original rank (1) and ligand-efficiency-based rank (2), respectively. No compounds were found from the novelty-based rank, which may indicate that finding novel compounds using an LB approach is difficult.

#### Novelty

**Figure 3:**
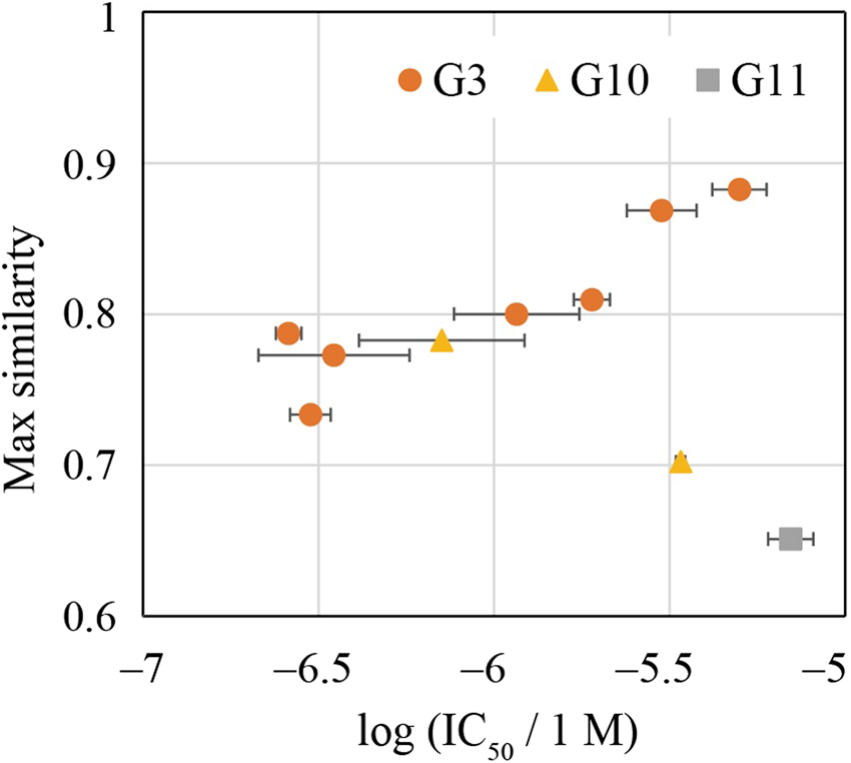
Similarity of each hit compound to known Src-family kinase inhibitors (see Section Preparation of compound library) is plotted against experimental inhibition activity. The error bars represent 68% confidence intervals estimated from IC_50_ assays. The similarity in these figures was calculated with the Tanimoto coefficient of the MACCS descriptor^41^. A chemical structure of the most similar compound of each hit is shown in Table S5 of the Supporting Information with its ChEMBL ID and literature.

It is of great importance to obtain a number of novel hit compounds in drug discovery^39^. We compared which hit compounds from LB or SB gave novel compounds in this study. First, we calculated similarities between each hit compounds and known Src-family inhibitors defined in the Preparation of compound library section. Among the similarities calculated for each of the compounds, the maximum value was assigned to the compound as the max similarity. The most novel compound was proposed by G11 (SB), which used docking for the selection of compounds, as can be shown in Fig. 3. The second was proposed by G10 (LB→SB), which used known inhibitors to filter the compound library followed by docking. Almost all the other compounds were proposed by G3 (LB), which used known active and inactive compounds to build a compound filter. Among seven hits from G3, four compounds were hydrazone (Z49895016, Z57745314, Z57745304, Z295464022) and had a similar scaffold as can be shown by their structures. This was because the training set G3 used contained 65 known hydrazone-containing compounds, in which 58 compounds had inhibition rate greater than 50%, in the total number of compounds used of 2040. Among the compounds used, 56 compounds of the hydrazone-containing compounds were derived from Published Kinase Inhibitor Set (PKIS), which collected results of kinase panel experiments of 367 kinase inhibitors and was released from GlaxoSmithKline. A similar scaffold was reported by a clustering analysis of PKIS^40^. This shows a clear dependency of the LB method on training data set used. Hence, we could say that an LB method is more likely to give similar hit compounds to known inhibitors in our contest. Conversely, one can resort to a method that uses an SB approach to obtain novel hit compounds. We also confirmed that hit compounds that were proposed by different groups were not similar to each other (see Figure S3 of the Supporting Information).

### Characteristics of the most successful method

Among all the groups, the hit rate of compounds proposed by G3 was statistically confirmed to be higher than the others. We summarize the salient characteristics of the method here. G3 employed a machine learning technique based on a training set that combined three kinds of data for known active and inactive compounds of the Src and relevant kinase families. These data included compound descriptors, experimental conditions when the inhibition rate was measured, and target protein information. Inhibition rates were used for training the model instead of inhibition constants or IC_50_ values because inhibition rates for compounds were relatively abundant. In some cases, G3 used inhibition rates that were measured for the determination of IC_50_ values. Among the LB methods, experimental conditions and protein information were not used except for G3. Here we focus on these two characteristics and investigate the significance of incorporating these features.

### Incorporation of experimental conditions for machine learning

G3 included several experimental conditions, as compiled in Table 3, when training the machine learning model using inhibition rates. This was based on the fact that an inhibition rate of a compound depends on experimental conditions (e.g., concentrations of compounds, enzyme, and ATP) and that experimental conditions can differ in different studies. Hence, incorporating these conditions in the training data sounds reasonable. Experimental conditions that accompanied the known compound information that G3 used were diverse, as seen in Table 3. The range of concentrations was broad, indicating that it is dangerous to build an SAR model based only on inhibition rates or IC_50_ values from different experimental studies.

**Table 3:** Range of experimental conditions used for training a machine learning technique^*a*^.

Feature	Reagent concentration (μmol L^−1^)			pH
	Compound	ATP	Mg^2+b^	
Average	4.6	91	5500	7.3
Minimum	4.6 × 10^−4^	10	0	7.5
Maximum	670	200	2 × 10^4^	7.0
Standard deviation	20	27	3900	0.2

^a^In addition to these features, dummy parameters that distinguish sources of experimental studies were combined with the training set.

^b^This range was calculated based on the actual training set used, which included trivial mistakes in retrieving experimental parameters. As Mg^2+^ usually coexists adequately in assay samples, it would not affect inhibition rates. G3 confirmed that removing the concentration of Mg^2+^ from the training set did not affect the result after participation in the contest.

To test the significance of incorporating experimental conditions, G3 conducted an OOB validation with and without experimental conditions. Excluding the experimental condition made prediction accuracy (*R* ^2^) decrease from 0.82 to 0.44. We believe that, especially in the case of building an SAR model as G3 conducted, considering experimental conditions would be crucially important if data sets are based on several experimental conditions. As which experimental conditions were significantly important was not clear in this study, further investigation and validation of the insights we obtained are needed. Further, incorporating substrate concentration, which was not used by G3, may help improve prediction accuracy.

### Incorporation of protein information for machine learning

**Figure 4:**
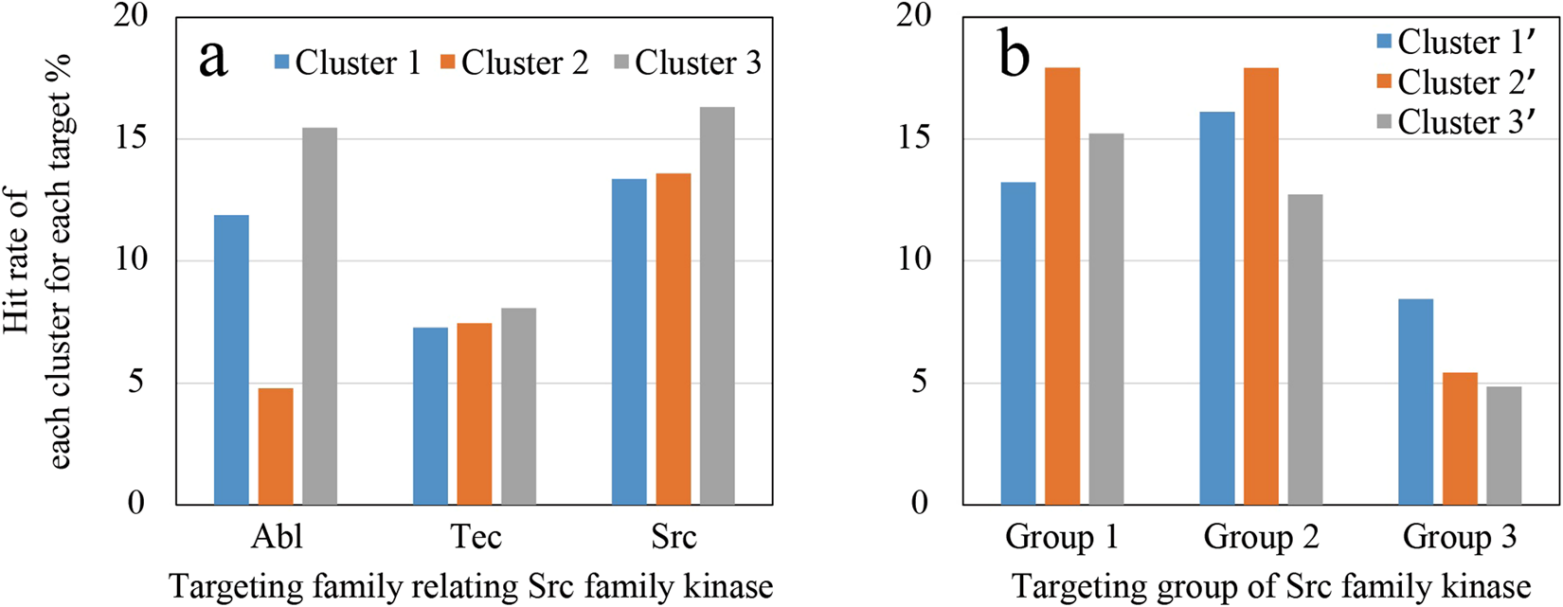
(**a**) Hit rate of compounds in each cluster with respect to the three kinase families. The hit rate was calculated by dividing the number of hit compounds by the number of compounds with inhibition rates that were measured to the family. (**b**) Hit rate of compounds in each cluster with respect to the three groups of Src-family kinases. The 11 kinases defined by the kinome were classified into three groups: Group 1: Src, Fyn, Yes, Fgr; Group 2: Blk, Hck, Lck Lyn; and Group 3: Frk Srm, Brk based on the kinome^42^. The clustering was calculated with Canvas^46, 47^ based on the k-means algorithm^48^ of the MACCS descriptor^41^. The clusters in Fig. 4a do not correspond to those in Fig. 4b.

G3 used compound information for Src, Tec, and Abl kinase families, which are closely related^42^ (The ChEMBL IDs and references used are tabulated in Table S6 of the Supporting Information). While some compounds interact with a broad range of kinases, others have selectivity to a specific kinase^43, 44^. To evaluate the selectivity of compounds that G3 used, we clustered the compounds into three groups and calculated the hit rates of compounds in each cluster with respect to each kinase group, in which the hit criterion was defined to be 50% inhibition. As can be seen in Fig. 4a, each cluster did not interact with each kinase family equally. This means that there is some selectivity of compounds in the three kinase families. To evaluate the selectivity of compounds within the Src family, we clustered the compounds that had experimental information available into three groups. As can be seen in Fig. 4b, the selectivity persists in these groups. The selectivity of Group 3 of Src-family kinases was different from the other two groups. This may be consistent with the fact that Group 3 is distantly related to the other groups^45^. Hence, incorporating protein information to compound descriptors and experimental conditions may improve prediction accuracy.

An OOB validation showed that excluding protein descriptors made the prediction accuracy worse, i.e., the *R* ^2^ decreased from 0.82 to 0.73, indicating that distinguishing protein targets was meaningful in this study. As the trained model can provide a potency of a compound for each kinase used in the training set, we could obtain a selective compound for a specific kinase. Interestingly, only combining a compound’s descriptor and protein information did not improve the prediction accuracy compared to using compound descriptors simply as a training set, i.e., the *R* ^2^ was only improved from 0.43 to 0.44 by introducing protein information. This means that protein information becomes useful when it is used with experimental conditions for a training set.

### Comparison to the previous contest

**Figure 5:**
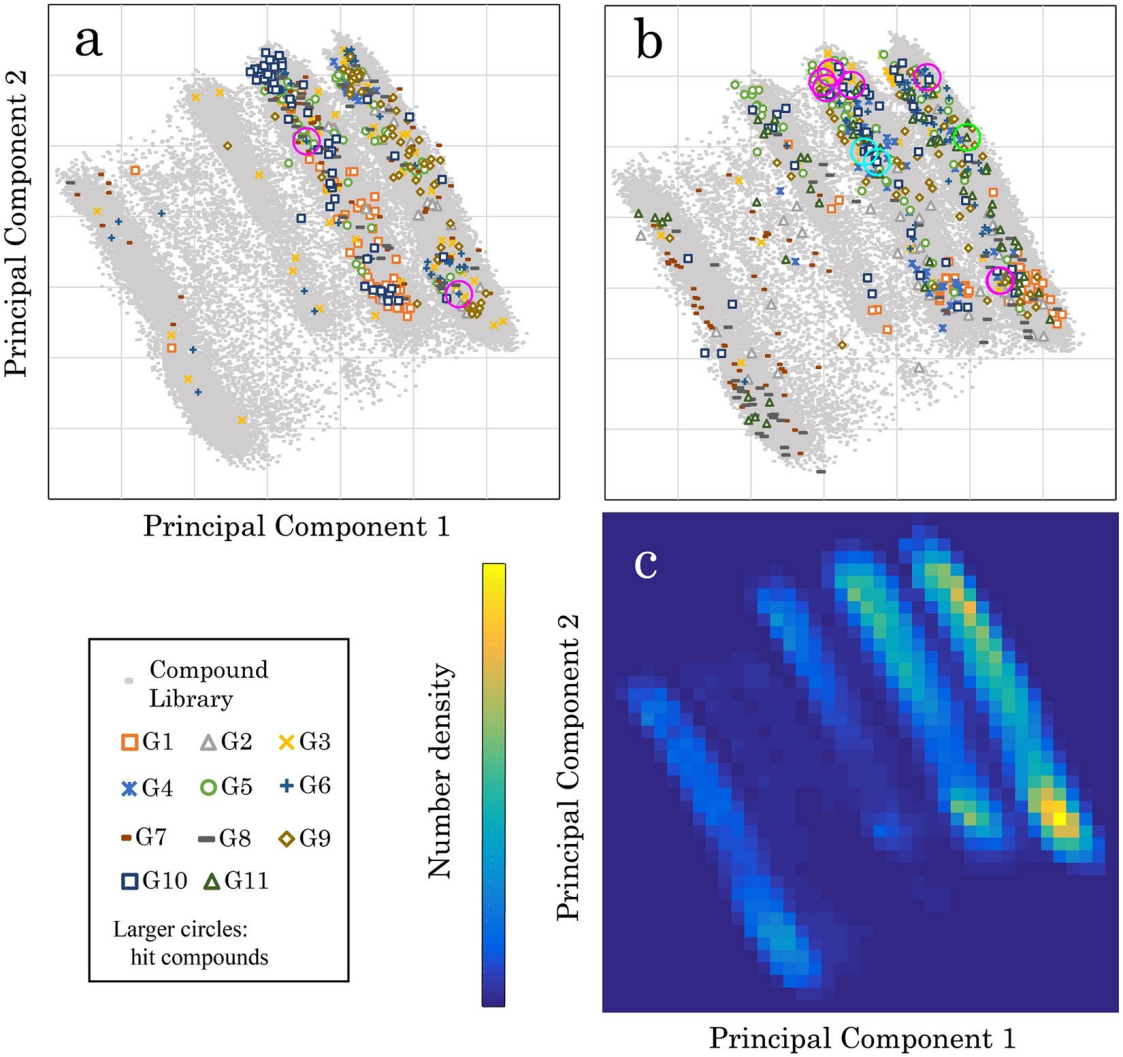
Diversified screening by collecting various computational methods. Principal component analysis of the library compounds in this study was applied, in which the MACCS fingerprint was used. The cumulative variance of the principal component (PC) 1 and 2 are 26% and 49%. (**a**) Compounds proposed from groups participating in the previous contest are projected to the PC1 and PC2. Two hit compounds confirmed based on IC_50_ determination are plotted. To avoid the complication of symbols, the top 60 compounds in the proposed list are shown. As for the compound library, a randomly chosen 2.5% of all the compounds are shown. (**b**) The same analysis as (**a**) is conducted using data from this study. Ten hit compounds (magenta for G3, cyan for G10, green for G11) are plotted. (**c**) Number density in the PC1 and PC2 of all the compounds are shown.

Comparing this study with the previous contest would give useful information. As we noted about the previous contest^10^, collecting various computational methods enables diversified screening in the chemical space of the contest library compared with a single method, as can be seen in Fig. 5 and Figure S4 of the Supporting Information. This reflects the diversity of hit compounds, as can be seen in Fig. 5b and Figure S3 of the Supporting Information. The contest-based approach can provide diverse hit compounds than a single method can do. In addition, comparing the chemical diversity of hit compounds of this study (Fig. 5b) to the previous contest (Fig. 5a), hit compounds obtained in this study had broader diversity.

The total hit rate improved from 2/600 to 10/1991 (hit compounds/assayed compounds). This improvement is remarkable considering that we eliminated known inhibitors of the Src-family from the contest library this time. In the previous contest, we eliminated known inhibitors of Yes, but all the hit compounds in the previous contest were known inhibitors of other Src-family kinases.

As we have discussed in “Experimental procedure and screening of potential inhibitors” and “Comparison of ligand-based and structure-based methods” sections, we decided not to exclude the hydrzones (Z49895016, Z57745314, Z57745304, Z295464022) and the potential Michael acceptor (Z449737600) from the hit list. However, it would be worth comparing this study to the previous contest with eliminating them from the list, because regarding them as possible compounds for lead optimization remains a matter of debate. The total hit rate decreases from 10/1991 to 5/1991, which is comparable to the previous hit rate of 2/600. Even though the questionable compounds were eliminated, considering the absence of known Src-family inhibitors in the compound library used, improvement of the second contest is warranted.

We speculate that iterative participation provides the opportunity for improvement in each method because the three groups that proposed hit compounds participated in both contests. Note that 92% of the compounds in the compound library in this study were included in the previous contest library and the ten hit compounds were also included in the compound library of the previous contest.

We expected to distinguish promising methods by increasing the number of compounds assayed. However, even if the number of assayed compounds for each group was reduced to the approximate number of assayed compounds in the previous contest (55), almost all hit compounds can be found (see Fig. 2b). The *p*-values for G3, G10, and G11 improve to 6 × 10^−7^, 0.04, and 0.26, respectively. Hence, the method of G3 is statistically warranted. Apparently, we could reduce the number of compounds to assay in this sense. However, a sufficient number of compounds to assay is necessary to detect a method with a modest hit rate. If a method has a hit rate of 3%, at least one hit compound can be found in 99.6% of the time in this experiment that assayed 180 compounds for each group. In this regard, the experiment did not miss promising methods with a significant hit rate.

## Conclusion

The compound screening contest to predict potential inhibitors of the tyrosine-protein kinase Yes from the 2.4-million-compound library was held not only to identify potent inhibitors for the target and but also to benchmark various methods based on the same experimental conditions, in which 11 groups participated. Among 1,991 assayed compounds, ten hit compounds with IC_50_ values less than 10 μmol L^−1^ were identified, which are not likely to be frequent hitters in terms of the fact that they passed PAINS filters. Comparing this study with the previous contest, which was held by the same organizer with the same target^10^, the hit rate improved and the diversity of hit compounds grew broader.

The participating groups employed various approaches, which were classified as LB or SB approaches. Comparison of the LB and SB approaches by the three groups which proposed hits showed that the LB approach was more likely to give hit compounds, whereas the SB approach gives more novel hit compounds in our contest.

The characteristics of the most successful LB method, which identified seven hit compounds, were studied in terms of the training data set that the group used for a machine learning technique. We found that incorporation of experimental conditions, e.g., concentration of compounds under which inhibition rates were measured, significantly contributed to the prediction accuracy. In addition, the incorporation of protein descriptors to distinguish known compounds’ target kinase was found partly to contribute to improved prediction accuracy.

We confirmed that a contest-based approach to identify potential inhibitors of a target protein can be successful in identifying promising hit compounds. Moreover, it can provide an initial benchmark of various methods and suggests promising approaches for the target system. Extensive exploitation and further investigation of these methods should lead to additional novel hit compounds in the drug discovery process.

## Electronic supplementary material

Supplementary Information
