# Supplementary material for: An iterative compound screening contest method for identifying target protein inhibitors using the tyrosine-protein kinase Yes
Source: Sci Rep. 2017 Sep 20;7:12038. doi: 10.1038/s41598-017-10275-4 (PMC5607274; doi:10.1038/s41598-017-10275-4)
Supplement: Supplementary file 1 — Supplementary Information [file 41598_2017_10275_MOESM1_ESM.pdf]

Supporting Information for

An iterative compound screening contest method for identifying target protein inhibitors using the tyrosine-protein kinase Yes

Shuntaro Chiba[1,2], Takashi Ishida[1,3], Kazuyoshi Ikeda[4], Masahiro Mochizuki[5], Reiji Teramoto[6], Y-h. Taguchi[7], Mitsuo Iwadate[8], Hideaki Umeyama[8], Chandrasekaran Ramakrishnan[9], A. Mary Thangakani[10], D. Velmurugan[10], M. Michael Gromiha[9], Tatsuya Okuno[11], Koya Kato[12], Shintaro Minami[13], George Chikenji[12], Shogo D. Suzuki[3], Keisuke Yanagisawa[3], Woong-Hee Shin[14], Daisuke Kihara[14,15], Kazuki Z. Yamamoto[16], Yoshitaka Moriwaki[17], Nobuaki Yasuo[3,], Ryunosuke Yoshino[17,18], Sergey Zozulya[19,20], Petro Borysko[19,20], Roman Stavniichuk[19], Teruki Honma[1,3,21], Takatsugu Hirokawa[22,23,24], Yutaka Akiyama[1,2,3,22,24], Masakazu Sekijima[1,2,3,18,24*]

[1]Advanced Drug Discovery Unit, Institute of Innovative Research, Tokyo Institute of Technology, J3-23 4259 Nagatsuta-cho, Midori-ku, Yokohama 226-8501, Japan

[2]Education Academy of Computational Life Sciences, Tokyo Institute of Technology, J3-141 4259 Nagatsuta-cho, Midori-ku, Yokohama 226-8501, Japan

[3]Department of Computer Science, Tokyo Institute of Technology, 2-12-1, Ookayama, Meguro-ku, Tokyo 152-8550, Japan

[4]Level Five Co. Ltd., Shiodome Shibarikyu Bldg., 1-2-3 Kaigan, Minato-ku, Tokyo 105-0022, Japan

[5]IMSBIO Co., Ltd., Level 6 OWL TOWER, 4-21-1 Higashi-Ikebukuro, Toshima-ku, Tokyo 170-0013, Japan

[6]Forerunner Pharma Research, Co., Ltd., Yokohama Bio Industry Center, 1-6 Suehiro-cho, Tsurumi-ku, Yokohama 230-0045, Japan

[7]Department of Physics, Chuo University, 1-13-27 Kasuga, Bunkyo-ku, Tokyo 112-8551, Japan

[8]Department of Biological Sciences, Chuo University, 1-13-27 Kasuga, Bunkyo-ku, Tokyo 112-8551, Japan

[9]Department of Biotechnology, Bhupat Jyoti Mehta School of Biosciences, Indian Institute of Technology Madras, Chennai 600036, Tamilnadu, India

[10]CAS in Crystallography and Biophysics and Bioinformatics Facility, University of

Madras, Chennai 600025, Tamilnadu, India
[11]Division of Neurogenetics, Nagoya University Graduate School of Medicine, 65 Tsurumai, Showa-ku, Nagoya 466-8550, Japan
[12]Department of Computational Science and Engineering, Nagoya University, Furocho, Chikusa, Nagoya 464-8603, Japan
[13]Department of Complex Systems Science, Graduate School of Information Science, Nagoya University, Furocho, Chikusa, Nagoya 464-8601, Japan
[14]Department of Biological Science, Purdue University, Indiana 47907, USA
[15]Department of Computer Science, Purdue University, Indiana 47907, USA
[16]Isotope Science Center, The University of Tokyo, 2-11- 16, Yayoi, Bunkyo-ku, Tokyo 113-0032, Japan
[17]Department of Biotechnology, The University of Tokyo, 1-1-1 Yayoi, Bunkyo-ku, Tokyo 113-8657, Japan
[18]Global Scientific Information and Computing Center, Tokyo Institute of Technology, 2-12-1, Ookayama, Meguro-ku, Tokyo 152-8550, Japan
[19]Bienta/Enamine Ltd., 78 Chervonotkatska Street, Kyiv 02660, Ukraine
[20]National Taras Shevchenko University of Kyiv, 64/13 Volodymyrska Street, Kyiv 01601, Ukraine
[21]Center for Life Science Technologies, RIKEN, 1-7-22 Suehiro, Tsurumi, Yokohama, Kanagawa 230-0045, Japan
[22]Molecular Profiling Research Center for Drug Discovery, National Institute of Advanced Industrial Science and Technology, 2-4-7 Aomi, Koto-ku, Tokyo 135-0064, Japan
[23]Division of Biomedical Science, Faculty of Medicine, University of Tsukuba, 1-1-1 Tennodai, Tsukuba-shi, Ibaraki 305-8575, Japan
[24]Initiative for Parallel Bioinformatics, Level 14 Hibiya Central Building, 1-2-9 Nishi-Shimbashi Minato-Ku, Tokyo 105-0003, Japan

* Correspondence to sekijima@c.titech.ac.jp

# contents

Figure S1. Dose-response curves for 16 best hit compounds.......................................S4
Figure S2. The relationship between the determined IC_{50} and its inhibition rate determined in the second screening. S5
Figure S3. Similarity among hit compounds S5
Figure S4. Similarity among assayed compounds. S6
Table S1. ChEMBL IDs of Src-family kinases used for making the compound library .S7
Table S2. Definition of druglikeness used for making the compound library S7
Table S3. Dropped compounds of the first screening. S8
Table S4. Inhibition rate of compounds assayed in secondary screening.....................S62
Table S5. Most similar known inhibitor of hit compounds...S65
Table S6. Known ligand information that Group 3 used..S67
References...S70
Methods used by each group...S71

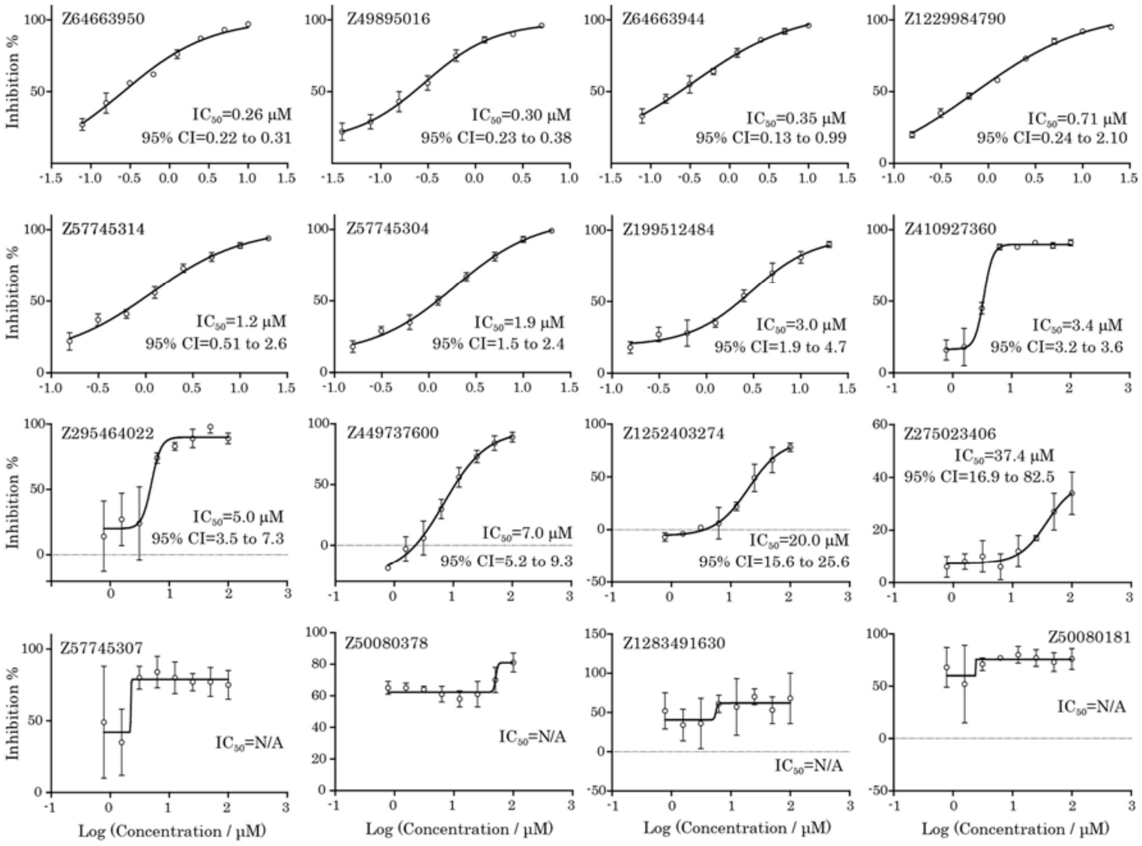

Figure S1. Dose-response curves for 16 best hit compounds.

Sixteen compounds with the highest Yes inhibitory activity at 10 µmol L^{-1} were tested to determine their IC_{50} values. Each dose-response curve was initially built using 8-point, 2-fold serial dilutions starting at 100 µmol L^{-1}, with each concentration point in 4 repeats. Concentration ranges were subsequently tuned for the compounds with submicromolar IC_{50} values. Assay was carried out under the same conditions as the primary screening, in summary, the final reagent concentrations were 5.5-nmol L^{-1} Yes, 0.013-mmol L^{-1} ATP, and 0.2-mg mL^{-1} substrate (poly Glu-Tyr peptides, Glu:Tyr=4:1). The curves were analyzed by GraphPad Prism (sigmoidal fit with variable slope). IC_{50} values in µmol L^{-1} and 95% confidence intervals are shown. The error bars represent standard deviations of the measurements. Compounds on the figure are sorted according to their IC_{50} values, starting from nanomolar to two-digit micromolar range. The compounds in the bottom row did not demonstrate a clear dose-response relationship.

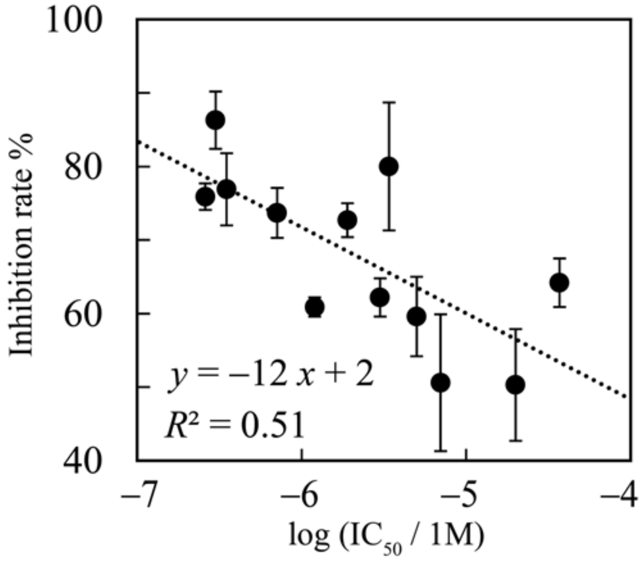

Figure S2. The relationship between the determined IC_{50} and its inhibition rate determined in the second screening.

It showed a negative correlation with the correlation coefficient of 0.71, indicating the threshold of approximately 50% used to determine if IC_{50} determination was conducted for a compound was reasonable because a compound with its inhibition rate less than the threshold would be unlikely to have IC_{50} better than 10 μmol L^{-1}.

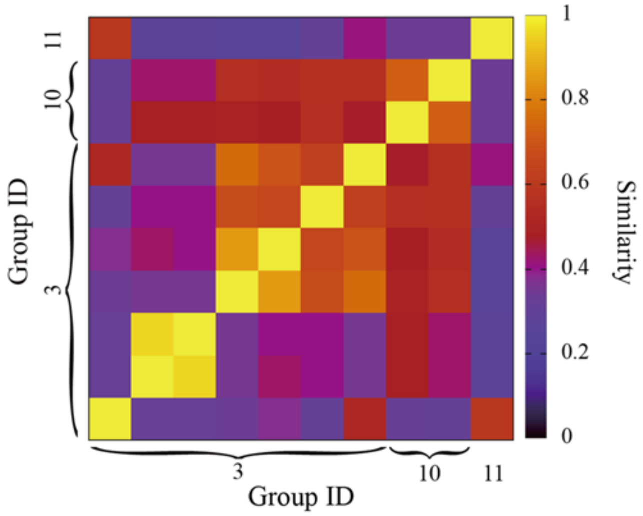

Figure S3. Similarity among hit compounds

Compounds are in order of Z199512484, Z64663950, Z64663944, Z57745314, Z295464022, Z49895016, Z57745304, Z410927360, Z1229984790, Z449737600. The similarity was calculated with the Tanimoto coefficient of the MACCS descriptor.[1]

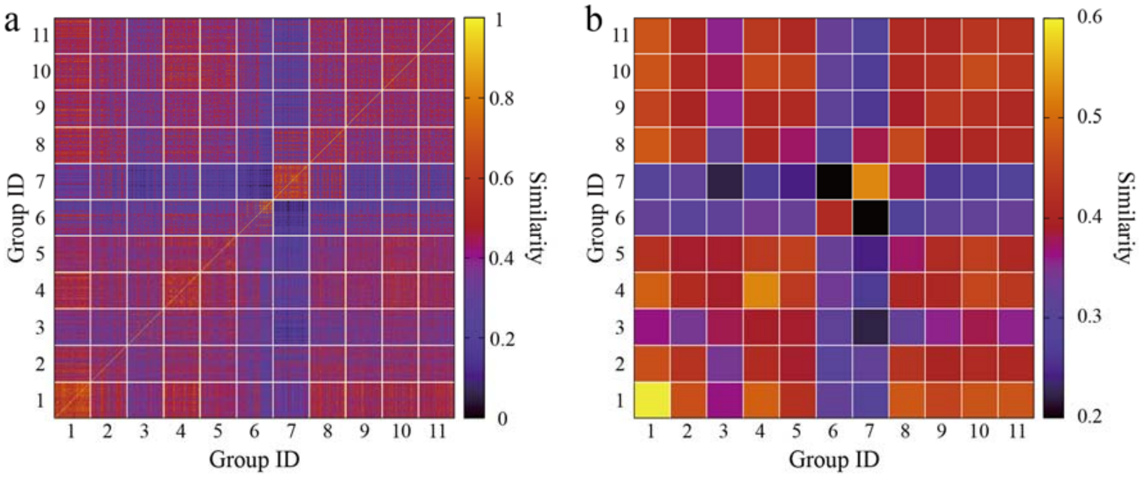

Figure S4. Similarity among assayed compounds.

(a) Similarities of all the assayed compounds are shown. (b) Similarities of assayed compounds from groups are averaged. When averaging similarities of compounds proposed from the same group, an identical compound are not included in the calculation. The compounds proposed from different groups tend to be different. The similarity was calculated with the Tanimoto coefficient of the MACCS descriptor.[1]

Table S1. ChEMBL IDs of Src-family kinases used for making the compound library

ChEMBL ID	Target molecule
CHEMBL4223	Tyrosine-protein kinase FRK
CHEMBL3234	Tyrosine-protein kinase HCK
CHEMBL3905	Tyrosine-protein kinase Lyn
CHEMBL2250	Tyrosine-protein kinase BLK
CHEMBL258	Tyrosine-protein kinase LCK
CHEMBL4454	Tyrosine-protein kinase FGR
CHEMBL5703	Tyrosine-protein kinase Srms
CHEMBL1841	Tyrosine-protein kinase FYN
CHEMBL267	Tyrosine-protein kinase SRC
CHEMBL2073	Tyrosine-protein kinase YES

Table S2. Definition of druglikeness used for making the compound library

Property	Range	Explanation
QPlogPo/w	–2.0 – 6.5	Predicted value of octanol / water partition
QPlogS	–6.5 – 0.5	Predicted value of solubility
#rtvFG	0 – 2	Number of reactive moieties

These values of each compound were calculated using Canvas Version 2.2.013.[2,3]

Table S3. Dropped compounds of the first screening.

Compound ID	canonical SMILES [a]	First screening % [b]	
		IR	SD
Z1000171966	N#CCSc(c1C)sc(n1)NC(=O)c2cc(ccc2)NC(=O)N	-2	4
Z1002220042	N#Cc1cc(F)c(cc1)N(CC2)CCC2C(=O)Nc(cc3C)ncc3	1	13
Z100622740	c1ccccc1Cn(c2)ncc2C(=O)Nc3ccc(cc3)S(=O)(=O)Nc4nccs4	61	12
Z100642558	CN(C)C(=O)c1c(nn(C)c1)-c(c2)ccc(c23)OCCCO3	17	15
Z1007027518	COc(cc1)c(OC)cc1-c2c(C#N)c(O)nc(c23)n(nc3C)-c4ccccc4	-1	4
Z1013750782	c1ccccc1C(=O)CC2(O)C(=O)Nc(c23)c(C)cc(C)c3	0	2
Z101840388	CNS(=O)(=O)c(cc1)ccc1NC(=O)c2c(-c3cccnc3)nn(c2)-c4ccccc4	11	9
Z101998542	n1c[nH]c(c12)ccc(c2)C(=O)NCCNc(nc3)c(Cl)cc3C(F)(F)F	25	10
Z1021076320	FC(F)(F)c1ccnc(n1)Nc2ccc(cc2)NC3CCOCC3	13	2
Z1021595256	O=C(O)c1cnc(s1)/C=C/c2cccnc2	0	7
Z102448822	CC(=O)c1c(C)c([nH]c1C)C(=O)NCCCn(cn2)c(c23)cccc3	20	20
Z102556578	o1cccc1C(=O)NC(CCSC)C(=O)NCc(cc2)ccc2CN(C)C	-3	7
Z1029489284	CNC(=O)c1cnc(s1)/C=C/c2cccnc2	5	16
Z103069550	c1cnccc1CCSc2ncnc(c23)n(nc3)-c4ccccc4	11	1
Z1031094372	CCN1CCN(CC1)S(=O)(=O)C2CS(=O)(=O)c(c23)cccc3	8	3
Z1031114832	CC1(C)CN(CCO1)S(=O)(=O)C2CS(=O)(=O)c(c23)cccc3	-4	8
Z1033300996	Cc1ccc(cc1)S(=O)(=O)C(¥NC=O)=C(/Sc2ccc(Cl)cc2)Sc3ccc(Cl)cc3	-9	8
Z1033301058	c1cccc(n2)c1nc(c23)n(c(N)c3C(=O)NCC(C)C)CCc4ccccc4	-2	5
Z1033314424	c1c(Cl)ccc(Cl)c1NC(=O)CN(CC2)CCC2CN(CC3C)CC(O3)C	2	18
Z1037335626	OCCNc1nc(nc(C)c1C)-c2ccc(Br)cc2	13	5
Z1037335968	Cc1cc(C)n(n1)-c2nc(-c(cc3)ccc3C)nc(c24)cccc4	10	12
Z1037385644	CCN(CC)C(=O)c1ccc(cc1)NS(=O)(=O)c(c2)c(Cl)cc(c23)NC(=O)C3	-4	2
Z1037939812	Cc(o1)ccc1C(=O)Nc(c(Cl)cc2)cc2C(=O)N3CCCCC3	3	2
Z1038829842	C1CN(C(=O)N)CCC1C(=O)N2CCCC2CC(C34)CC(C3)CC4	2	3
Z1039059796	N#Cc1cc(Cl)c(nc1)N2CCN(CC2)S(=O)(=O)c3c(Cl)ccc(Cl)c3	0	2
Z1039090738	N#Cc1cc(Cl)c(nc1)NCCC(=O)N(C2)CCc(c23)scc3	2	3
Z1039455578	NC(=O)c1cc(cn1C)NC(=O)Cc2c(C)oc(n2)-c3ccsc3	2	3
Z1040576806	c1cn(C)c(c12)cccc2NC(=O)NCc3cc(ncc3)N4CCCC4	-8	3
Z104378488	C1COCCN1CCNc(cc2)ccc2-c(cn3c4nc(c35)n(C)c(=O)n(C)c5=O)n4CCN6CCOCC6	26	9
Z104661722	c1ccccc1Cn(c(=O)[nH]c2=O)c(c23)nc(n3CCCC)CN4CCN(CC4)c5ccc(O)cc5	-9	5
Z104777504	o1cccc1C(=O)N2CCN(CC2)Cc(n3CCCC)nc(c34)n(c(=O)[nH]c4=O)Cc5ccccc5	-22	7

Z104925552	c1ccccc1Cn(c(=O)[nH]c2=O)c(c23)nc(n3CCCC)CN4CCN(CC4)c5ncccn5	9	8
Z104926054	c1ccccc1Cn(c(=O)[nH]c2=O)c(c23)nc(n3CCC(C)C)CN4CCN(CC4)c5ncccn5	-1	6
Z104935524	c1ccccc1Cn(c(=O)[nH]c2=O)c(c23)nc(n3CCCC)CN4CCN(CC4)c5ccccn5	2	7
Z104936026	c1ccccc1Cn(c(=O)[nH]c2=O)c(c23)nc(n3CCC(C)C)CN4CCN(CC4)c5ccccn5	9	3
Z105146244	c1cccc(c1C(F)(F)F)S(=O)(=O)N(CC2)CCN2Cc(o3)ncc3-c4ccc(Cl)cc4	-3	9
Z1051950052	Cc(n1)nn(c12)CC(CC2)NCc3c(n[nH]c3)-c(o4)ccc4C	20	12
Z105335628	s1c(Cl)ccc1S(=O)(=O)N(CC2)CCN2C(=O)CN3CCSc(c34)cccc4	-5	4
Z105957250	O1CCCC1Cn(c(C)c2)c(C)c2C(=O)CN3CCN(CC3)Cc4c(C)cccc4	15	5
Z105965792	O1CCCC1C(=O)N2CCN(CC2)Cc(n3CCCC)nc(c34)n(c(=O)[nH]c4=O)Cc5ccccc5	1	3
Z106611352	C1CCCCN1C(=O)C(c2ccccc2)N3CCN(CC3)CC(=O)Nc(c4C)c(C)ccc4	-11	16
Z106627256	CN(C)C(=O)CN1CCN(CC1)CC(=O)Nc(n(n2)-c3ccccc3)cc2-c(c4)ccc(c45)CCC5	-3	10
Z1067269008	CN(C)C(=O)Cn(c1)ncc1NC(=O)c(cc2)cc(c23)[nH]cc3	-5	9
Z1068296272	FC(F)(F)c1cc(ccc1)C2(CC2)C(=O)Nc3cnn(C)c3	25	12
Z106861102	C1CCCC1NC(=O)c2c(cccc2)NC(=O)CN3CCN(CC3)Cc4ccc(cc4)OC	4	2
Z1070397714	c1cnccc1-c(nc(c23)CCC2)nc3N(C4)CCCC4N5CCCC5	56	4
Z107308838	c1c(Br)ccc(c1C)NC(=O)Cn(n2)c(C)c(c2C)S(=O)(=O)N(CC3)CCC3C	-17	1
Z1082320758	c1ccccc1CCC(=O)Nc(c2C)c(ccc2)-c(on3)nc3C(C)C	4	7
Z1082504452	c1nccc(O)c1NC(=O)C2(CCCC2)S(=O)(=O)c(c3C)cc(C)cc3	-15	12
Z1082766798	CC(C)NC1CCN(CC1)c(c2[N+]([O-])=O)ccc(c2)S(=O)(=O)N(CC)CC	8	8
Z1082836308	c1ncccc1N(CC)S(=O)(=O)c2c(Cl)ccc(c2)S(=O)(=O)C	-7	13
Z1082836720	O=S1(=O)CCN(CC1)S(=O)(=O)N(C)C2CCCCC2	-4	4
Z1084012684	Cc1cccc(=O)n1CCCCNc(n2)cnc(c23)cccc3	10	3
Z108531934	c1cccc(F)c1C(=O)Nc([nH]n2)nc2-c3c(Br)cccc3	-2	2
Z108548594	c1cc(F)ccc1C(=O)Nc([nH]n2)nc2-c3ccc(F)cc3	44	12
Z108548640	COc(cc1)ccc1C(=O)Nc([nH]2)nnc2-c3ccc(F)cc3	6	9
Z108548764	c1cc(Cl)ccc1OC(C)(C)C(=O)Nc([nH]2)nnc2-c3ccc(F)cc3	16	2
Z108549090	c1ccccc1SCCCC(=O)Nc([nH]2)nnc2-c3ccc(F)cc3	6	12
Z108549754	c1cccc(F)c1C(=O)Nc([nH]2)nnc2-c3ccc(F)cc3	-11	3
Z108567148	C1COCCN1c2nc(C(F)(F)F)nc(c23)n(nc3)Cc4ccccc4	-2	12
Z108748406	C1CCCCC1CNC(=O)c2cc(-c3ccccc3)nc(c24)n(nc4)Cc5ccncc5	8	4
Z108907460	c1sccc1-c(n2)scc2C(=O)Nc(n3)sc(c34)CN(CC4)Cc5ccccc5	8	6
Z1090594860	CC(=O)N(CC1)c(c12)ccc(c2)C(=O)Nc(n3)n(c(c34)cccc4)CCCOCC	-9	9
Z109300154	CON(C)C(=O)c1cc(-c2ccccc2)nc(c13)n(nc3)Cc4ccncc4	-8	4
Z109522106	C1CC1NC(=O)CN(CC2)CCN2C(=O)c3cc(-c4ccccc4)nc(c35)n(nc5)Cc6ccncc6	12	8
Z1095448120	c1cccc(c12)oc(n2)CSc(n[nH]3)nc3-c4cccnc4	-2	5
Z109627928	CN(C)C(=O)CN(CC1)CCN1C(=O)c2cc(-c3ccccc3)nc(c24)n(nc4)Cc5ccncc5	-10	7

Z1097089047	c1cccc(F)c1C(=O)NCCNCC(CN2)Cn(c23)ncc3	-1	4
Z1097879141	c1cnn(c12)CC(CN2)CNCc3ccc(cc3)CN(C4)CCCC4C	-4	6
Z110143980	c1c(Cl)ccc(OC)c1CN(C)C(=O)c2cc(C(C)C)nc(c23)n(C(C)C)nc3	-14	11
Z1101445259	CC1CCCN(C1)c2nc(nc(N)c2[N+]([O-])=O)N3CCCC3	1	6
Z1102775620	Cc(n1)scc1-c(oc2)cc2C(=O)N(C3)CCCC3n4ccnc4	-5	4
Z1103241348	c1ccccc1C2=NN(CC2)C(=O)CN3CCCC3c4cnn(C)c4	1	6
Z1116704440	N#CCCN(CC)S(=O)(=O)c(ccc1)cc1S(=O)(=O)C	-6	6
Z1116705979	COCCN(CCC#N)S(=O)(=O)c(c1S(=O)(=O)C)cccc1	42	19
Z111781008	c1cccc(OC)c1C(=O)Nc2ccc(cc2)Nc(nc(c34)ccc(C)c4)nc3-c5ccccc5	8	6
Z1117914118	C1CCCc(c12)ccc(c2)C(C)NCc3c(O)ccc(n3)C	7	6
Z1119644111	C1CC(=O)N(C)N=C1C(=O)NCc2nc([nH]n2)-c3ccc(cc3)OC	4	5
Z1120830695	c1cc(Cl)ccc1C(C)(C)NCc(o2)nnc2C(C)C	17	10
Z1120846907	c1cc(F)ccc1C(C)(C)NCc(o2)nnc2C(C)C	3	10
Z1124129708	c1[nH]ncc1Nc(ccn2)nc2-c3ccccc3	16	6
Z112433702	c1cc(O)ccc1C(=O)CSc2n[nH]c(n3)n2c(c34)cccc4	11	11
Z112518822	[nH]1c(=O)[nH]c(c12)ccc(c2)NC(=O)C(C)Sc(cc3C)nc(c34)cccc4	-20	8
Z1126778415	c1cc(F)ccc1OCC(=O)Nc2ccc(cc2)SC(=O)N(C)C	-17	18
Z1126778674	c1ccc(F)cc1OCC(=O)Nc2ccc(cc2)SC(=O)N(C)C	-2	5
Z1126778839	Cc1cc(ccc1)OCC(=O)Nc2ccc(cc2)SC(=O)N(C)C	-6	3
Z1127155490	NC(=O)c1c(C)cc(cc1)N(C(C)C)Cc(n2)cn(c23)ccs3	-7	5
Z1127188257	CCN1CCCN(CC1)S(=O)(=O)c(c2S(=O)(=O)C)cccc2	43	2
Z1127660495	CN(C)S(=O)(=O)CCCS(=O)(=O)Cc1c(C)ccc(C)c1	12	9
Z1127660834	CN(C)S(=O)(=O)CCCS(=O)(=O)Cc1cc(F)ccc1	6	2
Z1127660880	CN(C)S(=O)(=O)CCCS(=O)(=O)Cc1ccc(Cl)cc1	-4	3
Z1128365659	n1cccn1-c(cc2)c(F)cc2C(=O)Nc([nH]3)nnc3-c4ccccn4	4	3
Z1129155203	O=C1CCCN1CCC(=O)Nc(nc2)ccc2Oc3ccc(F)cc3	-12	3
Z1129440199	CNc1snc(C)c1C(=O)NC(C)c2c(C)nc(s2)-c3ccc(F)cc3	29	18
Z112958446	Cc1c(C)sc(c12)nc(CN(CC3)CCC3C)n(c2=O)CC(=O)Nc4ccc(cc4)N5CCCC5	-4	8
Z1130339086	n1cn(C)c(Cl)c1S(=O)(=O)N2CCCc(c23)cc(Br)cc3Cl	8	13
Z1131322534	c1ccc(CC)c(c12)[nH]cc2C(=O)N(C3)CCCC3n4cccn4	19	15
Z1131596340	[nH]1cncc1C(=O)Nc(c2)ccc(c23)N(CCC3)C(=O)OC(C)(C)C	1	4
Z1134191614	CN(C)S(=O)(=O)CCCS(=O)(=O)c(c1C)ccc(C)c1	-1	6
Z1134191724	CN(C)S(=O)(=O)CCCS(=O)(=O)c1nccn1C	22	16
Z1135283259	c1ccc(Br)c(O)c1CNCc2ncc(o2)-c3ccccc3	-4	6
Z113592752	C1CCc(c12)sc(c2C(=O)N)NC(=O)C(C)Sc(c3)ccc(c34)CCC4	1	2
Z1137565280	Cc(n1)cccc1C(=O)NCc2nc(n[nH]2)-c3ccc(F)cc3	38	15

Z1137567528	N#Cc1cc(ccc1)C(=O)NCc2nc(n[nH]2)-c3ccc(F)cc3	55	10
Z1138834013	n1ncccc1N(C)C2CCNCC2	-2	3
Z1139215576	n1cccn1CC2CN(CCO2)c3ncnc(c34)n(C)nc4	18	24
Z1139260378	CNc1snc(C)c1C(=O)N(C2)CCc(c23)nnc(c3)NCC	7	3
Z1139343408	c1nccn1CCC(=O)N(C2)CCCC2n(n3)c(C)nc3C	0	2
Z1139411735	CC(C)c(n1)nn(c12)CC(CC2)NCc3cc(on3)-c4cccs4	-5	11
Z1139436344	CCc(nn1)n(c12)CCN(C2)CC(=O)NC3CCCc(c34)cccc4	-6	12
Z1139562831	n1cnn(c12)CC(CC2)NCc3c(nn(C)c3)-c4ccncc4	22	18
Z113967060	CC(C)CCCC(C)NC(=O)CSc(nn1)n(c12)c3c(cccc3)c(=O)n2-c4cccc(C)c4C	15	5
Z113972480	NC(=O)c1ccc(cc1)NC(=O)C(C)SC(SC2)=Nc(c23)cccc3	2	5
Z114088706	Cc1onc(C)c1COC(=O)CCc2nc(no2)-c3cccs3	9	9
Z114120486	C1CCc(c12)sc(c2C(=O)N)NC(=O)C(C)Sc(n3)nc(cc3C)-c4ccccc4	-3	6
Z1143064210	n1cccn1C(C)C(C)NCc2c([nH]nc2)-c(cc3C)ccc3	-7	11
Z1143403580	c1ccnc(c1C)NC(=O)CCC(=O)N2CCOc(c23)cccc3	8	4
Z1143428685	CC(C)c(n[nH]1)c(Br)c1C(=O)Nc(n2)sc(c2C)SC	3	5
Z1145185709	Cc1c(S(=O)(=O)C)cc(C)cc1S(=O)(=O)N2CCCCCC2	6	4
Z1145185785	Cc1c(S(=O)(=O)C)cc(C)cc1S(=O)(=O)N(C2)CCCC2C	14	32
Z1145185872	C=CCN(CC=C)S(=O)(=O)c1cc(C)cc(c1C)S(=O)(=O)C	4	8
Z1145197816	N#CCCN(CC)S(=O)(=O)c1cc(C)cc(c1C)S(=O)(=O)C	-2	5
Z1147565999	C1CCCC1S(=O)(=O)Cc2ccc(o2)S(=O)(=O)N(C)C	-1	4
Z114950222	N1C(=O)COc(c12)ccc(c2)C(=O)/C=C/c3cc(OC)c(O)cc3	1	5
Z114950486	N1C(=O)COc(c12)ccc(c2)C(=O)/C=C/c3ccc(cc3)N(CCC)CCC	26	15
Z115004236	c1cc(Cl)cc(F)c1NC(=O)c2c(C)nc(s2)-c3ccsc3	6	3
Z115152726	C1CCCCCN1S(=O)(=O)c(c(Cl)cc2)cc2C(=O)N(C)Cc3cnn(c3)-c4ccccc4	4	5
Z115241120	N#Cc1c(C)c(c(C)[nH]c1=O)CCC(=O)N2CCN(CC2)S(=O)(=O)c(c3C)c(C)on3	12	5
Z115678344	CC(C)(C)NC(=O)CN1CCCN(CC1)S(=O)(=O)c(c2C(F)(F)F)ccc(Cl)c2	2	6
Z1157725045	c1cccc(c1C)NC(=O)CSc(nn2)n(c23)c4c(c(=O)n3CC(C)C)ccc(c4)C(=O)NC(C)CC	-8	6
Z1157725046	Cc1cc(ccc1)NC(=O)CSc(nn2)n(c23)c4c(c(=O)n3CC(C)C)ccc(c4)C(=O)NC(C)CC	0	6
Z1157726321	C1COCCN1CCNC(=O)CCc(nn2)n(c23)c4c(cccc4)c(=O)n3Cc5ccc(C)cc5	-2	14
Z1157726355	Cc1ccc(cc1)NC(=O)CSc(nn2)n(c23)c4c(c(=O)n3CC(C)C)ccc(c4)C(=O)NC(C)CC	-5	9
Z1157834172	C1COCCN1CCCNC(=O)CCc(nn2)n(c23)c4c(cccc4)c(=O)n3Cc5ccc(Cl)cc5	-1	7
Z1157834228	COc(cc1)c(OC)cc1CCNC(=O)CSc(nc(c2c34)cccc2)n4nc(n3)CCN5CCCCC5	28	16
Z1157834229	O1COc(c12)ccc(c2)NC(=O)C(CC)Sc(nc(c3c45)cccc3)n5nc(n4)CCN6CCCCC6	-8	5
Z1157836557	o1cccc1CN(C(C)C)CCNC(=O)CCc(nn2)n(c23)c4c(cccc4)c(=O)n3Cc5c(Cl)cccc5	9	8
Z1160866358	c1cccc(c12)nc(C(C3C(=O)O)C(C4)C=CC34)n5c2nc(=O)c(n5)-c(cc6)ccc6C(C)C	17	19
Z116386688	O=C1CCCN1CCCNC(=O)c2cc(-c3ccccc3)nc(c24)n(nc4)Cc5cc(F)ccc5	3	5

Z1165943474	COc(c1)ccc(c12)[nH]cc2C3=CCN(CC3)c(nc4)ncc4Br	-6	7
Z1168201963	CC(C)C(C)NC(=O)C1CCN(CC1)c2cncc(n2)SCC	4	6
Z116821638	c1ccccc1-c(cc2C(=O)N)nc(c23)n(nc3)Cc4ccc(Cl)cc4	1	7
Z1168219830	NC(=O)c1cc(Cl)c(nc1)N(CC2)CCC2C3CCCCCN3C(=O)NC(C)C	13	13
Z116834630	c1ccccc1C(N(C)C)CNC(=O)c2cc(-c3ccccc3)nc(c24)n(nc4)Cc5ccncc5	-16	8
Z1169067733	o1cccc1CN(C(C)C)CCNC(=O)CCc(nn2)n(c23)c4c(cccc4)c(=O)n3Cc5ccccc5	3	9
Z1169067737	C1COCCN1CCCNC(=O)CCc(nn2)n(c23)c4c(cccc4)c(=O)n3Cc5ccc(C)cc5	2	2
Z1169067743	CC1CCN(CC1)CCCNC(=O)CCc(nn2)n(c23)c4c(cccc4)c(=O)n3Cc5ccc(C)cc5	18	16
Z1169067782	o1cccc1CN(C(C)C)CCNC(=O)CCc(nn2)n(c23)c4c(cccc4)c(=O)n3Cc5ccc(C)cc5	4	3
Z1169067814	CC1CCN(CC1)CCCNC(=O)CCc(nn2)n(c23)c4c(cccc4)c(=O)n3Cc5ccc(F)cc5	-3	12
Z1169067840	o1cccc1CN(C(C)C)CCNC(=O)CCc(nn2)n(c23)c4c(cccc4)c(=O)n3Cc5ccc(F)cc5	-10	9
Z1170101191	N#Cc1c(C)c(c(C)[nH]c1=O)CCc2nc(no2)-c3cc(Br)cs3	10	7
Z1170274264	CC1CCN(CC1)CCCNC(=O)CCc(nn2)n(c23)c4c(cccc4)c(=O)n3Cc5ccc(Cl)cc5	0	3
Z1170427223	COCC(=O)N1CCCCCC1C2CCN(CC2)c(nc3)c(Cl)cc3C(=O)N	2	5
Z1170432033	C1CC1C(=O)N2CCCCCC2C3CCN(CC3)Cc4nnc(o4)C5CC5	7	3
Z117073454	C1CCN1C(=O)c2cc(-c3ccccc3)nc(c24)n(nc4)Cc5cc(F)ccc5	5	7
Z1171689075	Cc(n1)[nH]cc1C(=O)NC(C)c2cc(ccc2)NC(=O)c3ccccc3	-17	21
Z1172045589	Cc1[nH]nc(C)c1C(C)C(=O)NCC(N2CCCC2)c3cc(Cl)ccc3	9	7
Z1172235206	CC(=O)Nc1ccc(cc1)Nc(cc(n2)C(C)(C)C)n(c23)nc(C)c3-c4c(Cl)cccc4	6	13
Z1172235406	OCCN1CCN(CC1)c2ncnc(c23)n(nc3)Cc4ccc(C)cc4	-13	7
Z1172235408	c1cccc(Cl)c1Cn(nc2)c(c23)ncnc3N4CCOCC4	-4	8
Z1172235413	c1ccccc1CCNc2ncnc(c23)n(nc3)Cc4c(Cl)cccc4	4	3
Z1172235419	c1cccc(Cl)c1Cn(nc2)c(c23)ncnc3N4CCCC4	45	8
Z1174718157	CCSc(nc1)ccc1C(=O)NCCc2nnc([nH]2)-c3ccco3	62	4
Z117548264	c1cccc(F)c1-n(n2)c(C)cc(=O)c2C(=O)N(CC3)CCC3C(=O)N4CCCCCC4	2	8
Z1177677805	[nH]1ncc(Br)c1C(=O)N(C2)CCCC2C(=O)Nc3nccs3	6	3
Z117807680	CCN(CC)Cc1c(cccc1)CNC(=O)c2c(C)nn(c2C)-c3ccc(F)cc3	9	1
Z1179617819	NC(=O)c1ccc(cc1)Nc(cc(n2)C)n(c23)nc(CC)c3-c4ccc(F)cc4	6	3
Z1179617914	c1cccc(c12)n(cn2)CC(O)COc3ccc(cc3)OCC(O)Cn(cn4)c(c45)cccc5	-9	10
Z1182665660	n1[nH]cc(c1C)Nc(cc2)nc(c23)nccn3	6	3
Z1184925017	Cc(c1)ccc(c12)nc3c(c2C(=O)O)CCCC¥3=C¥c4cnn(c4)C(C)C	-1	12
Z118512882	Cc1ccc(cc1)Nc2ccnc(n2)Nc(cc3)ccc3C	-5	4
Z118600166	NC(=O)c1c(O)ccc(c1)-c2csc(n2)NCc3ccc(F)cc3	4	4
Z118604520	C1CC1C(=O)Nc(ccc2)cc2-c(n3)scc3-c(c4C)cc(C)n4-c5nccs5	4	5
Z118688092	O=S1(=O)CC(CC1)NC(=O)c2c(Cl)c(Cl)c(Cl)c(n2)Cl	-7	3
Z118811128	[nH]1c(=O)[nH]c(c12)ccc(c2)NC(=O)c3c(cco3)-c4ccccc4	19	6

Z1189154512	N#Cc1cc(ccc1)NC(=O)NCCNC(=O)c2c(F)cccc2F	-6	4
Z1190394303	C1CC1C(C2CC2)NC(=O)C3CCCN3c4ncnc(c45)scc5	6	15
Z1191932195	c1ccnc(O)c1C(=O)NCc2cc(F)c(cc2)N(CC3=O)CCN3	5	5
Z1192169075	FC(F)(F)c1ccnc(n1)Nc(cc2)ccc2NCc3ccccn3	39	10
Z1193055616	CS(=O)(=O)C(C)C(C)N(C)C(=O)c(c1)[nH]c(c12)cc(Br)cc2F	8	3
Z1193297141	c1cc(F)ccc1NC(=O)NC2CCN(C2)c3ncnc(c34)nc[nH]4	-11	1
Z1193297244	COc(cc1)ccc1N(CC2)CCC2Nc3ccnc(c34)cc(F)cc4	16	11
Z1193464552	CCC(C)CS(=O)(=O)N(C)CC	-7	4
Z119708890	C1CCCc(c12)scc2C(=O)Nc([nH]3)nnc3-c4ccc(F)cc4	-5	9
Z119762492	c1ccc(OC)cc1C(=O)Nc([nH]2)nnc2-c(cc3)ccc3C	-10	4
Z119762582	c1cc(F)cc(F)c1C(=O)Nc([nH]2)nnc2-c(cc3)ccc3C	-5	8
Z119820616	N#Cc1c(C)c(c(C)[nH]c1=O)CCC(=O)Nc(s2)nnc2C(C)C	32	19
Z1198921141	COc(cc1)ccc1-n(n2)ccc2C(=O)N3CCCCC3c4cn[nH]c4	5	8
Z1199266078	FC(F)(F)c1ccnc(n1)Nc(c2)ccc(c23)NC(=O)C3	13	10
Z1199845522	C1COCCC1(N(C)C)CNC(=O)CNC(=O)c2cc(F)cc(F)c2	6	16
Z119992430	Cc(n1)scc1Cc2nnc(s2)Nc(cc3)ccc3-c(n4)sc(c45)cc(C)cc5	40	3
Z1203120735	C1COCCN1C2(CCCC2)C(=O)Nc(ccc3)cc3-c(n4)[nH]c(=O)cc4CC	3	8
Z1203241726	COC1CN(C1)C(=O)c2c(-c3cccnc3)nn(c2)-c4ccccc4	7	10
Z1203586543	NCC(S1(=O)=O)CCC1	6	3
Z1204166998	c1ncccc1C(N2C)C(CC2=O)C(=O)Nc3cc(ccc3)-n4c(=O)[nH]cc4C	-13	7
Z1204955922	C1CCCCC1C(=O)Nc(ccc2)cc2C(=O)N3CCN(CC3)c(nc4)ccc4CN(C)C	-2	10
Z1211655765	CN(C)C(=O)C1=C(C)NC(=S)NC1c(o2)ccc2-c3c(F)cccc3F	1	7
Z1212734184	C1COCCN1C(=O)c2cc(Cl)c(nc2)N(C)C3CCC(CC3)OC	-2	12
Z1220303895	CS(=O)CCNC(=O)c1ccc(s1)-c2cc(Cl)cc(Cl)c2	-13	9
Z1220618809	CC(C)(C)CC1CCCN1c2ncnc(c23)n(nc3)CC(=O)OC	25	7
Z1220618861	COC(=O)Cn(nc1)c(c12)ncnc2NC3CCN(CC(F)F)CC3	15	6
Z1220626900	COC(=O)Cn(nc1)c(c12)ncnc2NCCCc3cc(F)ccc3	-8	6
Z1227522997	c1cccc(OC)c1NC(=O)C(=C(N2)C)C(c3ccncc3)N(C=24)NC(=N4)SCc5ccccc5	-5	8
Z1229892973	Clc1cccc(F)c1C(=O)NCC(C(O2)C)N(C2(C)C)C(=O)OC(C)(C)C	-2	11
Z1230778510	CS(=O)(=O)CCCS(=O)(=O)N1CCCCC1CC	-14	6
Z1231909655	Fc1cc(Br)cc(c12)[nH]c(c2)C(=O)NC3CN(C3)C(=O)OC(C)(C)C	-12	11
Z1233805260	N1C(=O)NC(=O)C12CCN(C2)C(=O)c3ccc(cc3)CN(CC)CC	5	10
Z1238645651	CS(=O)(=O)CCS(=O)(=O)N(CC1)CCN1C	-2	2
Z123938232	NC(=O)C1CCN(CC1)C(C)C(=O)N2CCN(CC2)S(=O)(=O)c3ccccc3	0	4
Z1250487636	Cc1ccc(cc1)-n2cnc(c23)CNCC3	-7	3
Z1250701189	NC(=O)c1ccc(cc1)C(=O)N2CCN(CC2)C(S3)=NCC3(C)C	3	9

Z1251624666	Cn(c1)ncc1CCC(=O)N2CC(CC)N(CC2CC)C(=O)OCc3ccccc3	-2	7
Z1251687771	Cn(n1)c(C)cc1C(=O)Nc(n2)n(c(c23)cccc3)C4CCCC4	9	4
Z1252759503	COC(=O)c1cc(c(F)cc1)S(=O)(=O)N(C)C2C(C)(C)C(C23)OCC3	3	2
Z1255568722	c1cccc(c1C)N(CC)CCNC(=O)CCN2CCN(CC2)c3ccccn3	27	7
Z125608446	O1CCCC1Cn(c(C)c(C)c2C#N)c2NC(=O)CN3CCN(CC3)Cc(n4)sc(c45)cccc5	12	5
Z1256754058	CCc1cc(on1)CNC(=O)NCc(c2)ccc(c23)cc(cc3)OC	-8	9
Z1259162322	NS(=O)(=O)CCS(=O)(=O)c(cc1)ccc1F	4	10
Z126042696	C1CCCCCN1C(=O)CN2CCN(CC2)Cc([nH]c3=O)nc(c34)scc4-c5ccc(F)cc5	22	12
Z126044810	C1CCCCCN1C(=O)CN2CCN(CC2)Cc([nH]c3=O)nc(c34)sc(C)c4-c(cc5)ccc5C	-3	3
Z1262256786	CC(C)(C)OC(=O)N(CC1)CCC12C(=O)N(C(=O)N2)Cc3csc(n3)CCC(C)C	-2	7
Z1262455598	CCCCCNS(=O)(=O)CCS(=O)(=O)C	-9	6
Z1262519784	C1=CC(C(=O)O)=CN=S1(=O)C	52	9
Z1263913876	O1CCN(C1=O)c(ccc2)cc2C(=O)/C=C/c(cn3)n(c34)ccc(C)c4	44	13
Z126444476	c1cccc(c12)nc([nH]c2=O)C(C)N(C)CCOc3c(Cl)cccc3	12	13
Z1266299170	CC(C)(C)OC(=O)N1C(CC2)CC(C12)C(=O)N(C)c(n[nH]3)cc3-c4ccccc4	17	12
Z1266465668	c1cccc(c12)[nH]c(n2)CNC(=O)C3C(C3)c(c4)ccc(c45)OCCO5	3	2
Z1269414490	N#Cc1ccc(cc1)N(C)C(=O)c2cc(ccc2)NC(=O)c3ccc(o3)Br	-5	4
Z126941634	o1cccc1Cn(c(C)c(C)c2C#N)c2NC(=O)CN(CC3)CCC3n4c(=O)[nH]c(c45)cccc5	9	6
Z1270084447	C1CCCC(C12)N(C(C)C2)c3ncnc(c34)n(nn4)-c5ccccc5	45	8
Z1270166647	c1cccc(c12)nc(-c3ccc(F)cc3)cc2-c4ccc(Cl)cc4	43	15
Z1270579889	Cc1c(Br)ccc(c1)S(=O)(=O)Nc(n(n2)C)cc2-c3ccncc3	54	15
Z1271251540	CS(=O)(=O)CCCS(=O)(=O)N(C1)C(C)CCCC1C	8	3
Z1271277951	CS(=O)(=O)CCCS(=O)(=O)N(CC1)CCCC1(C)C	-3	9
Z1271542350	COCCCCCn(nc1)c(c12)cc([N+]([O-])=O)cc2	7	5
Z1272453859	C1CC1c(on2)nc2COc(cc3)ccc3Nc(n4)nccc4C	4	6
Z1274438175	CS(=O)(=O)C1CSCCN1S(=O)(=O)c2c(Cl)cc(Br)cc2	-18	12
Z1276379731	CC(C)(C)n(c1)ncc1C(=O)NCc2ccc(cc2)-c3cccnc3	5	6
Z1279679753	c1c(F)ccc(c12)OC(C2)C(=O)Nc([nH]3)nnc3-c(n4)cccc4C	-4	5
Z128012326	COCCNC(=O)CN1CCN(CC1)c2nc(-c3cccnc3)nc(c24)scc4-c5ccccc5	9	7
Z128086498	c1cccn(c12)c(nn2)C(C)Nc3c([N+]([O-])=O)cc([N+]([O-])=O)cc3	-9	7
Z128141646	CC(C)(C)NC(=O)CN1CCCN(CC1)c2nc(C)nc(c23)scc3-c4cc(OC)c(cc4)OC	-7	6
Z1285074692	n1occc1CN(CC2)CCN2C(=O)c3c(O)ccc(c3)Cc4ccccc4	21	20
Z1291253354	c1n[nH]c(c12)CC(CC2)C(=O)NS(=O)(=O)Cc3ccc(Cl)nc3	-13	13
Z130194258	s1cccc1Cn(c(C)c(C)c2C#N)c2NC(=O)CN(CC3)CCC3n4c(=O)[nH]c(c45)cccc5	22	22
Z1309300961	COC(=O)c1cc(cn1C)S(=O)(=O)Oc(cc2)cc(c23)oc(=O)cc3C(C)C	1	7
Z1309541011	n1c(Cl)cncc1NCc2nc(n[nH]2)-c3ccc(F)cc3	-11	10

Z131022412	C1S(=O)(=O)CCC1(C)N(C)S(=O)(=O)c(c2)ccc(Cl)c2Cl	2	7
Z131022526	C1S(=O)(=O)CCC1(C)N(C)S(=O)(=O)c(cc2)ccc2C	3	6
Z131206652	c1cccc(c12)CC(C)(OC2=O)C(=O)N(CCC3)c(c34)ccc(c4)S(=O)(=O)N5CCCC5	-1	4
Z131231284	COc(c1)ccc(Br)c1C(=O)NCc2ccc(cc2)-c3csc(n3)C	-17	2
Z131246512	C1CCCc(c12)sc(n2)NC(=O)CCc3cnn(c3)-c4ccccc4	-5	8
Z1315092412	CN1CCC(=O)Nc(c12)cc(cc2)C(=O)N(C)C(C3=O)CCCN3c4ccccc4	16	0
Z1317978562	c1ccc(C(F)(F)F)cc1C(C)(O)CN(C)CCCN2CCN(C)CC2	5	5
Z1318975474	c1nc(C)ccc1C(=O)Nc(cc2)cc(F)c2OCc3ccccn3	-2	14
Z1323958075	c1coc(C)c1CNC(=O)c2cc(-c3cccnc3)nc(c24)n(nc4)Cc5cccnc5	42	9
Z1324037636	CS(=O)c(cc1)ccc1C(C)N(C)c2nc(C)nc(c23)n(C)nc3	9	10
Z132693290	c1ccc([N+]([O-])=O)cc1NC(=O)C(C)Nc2ccc(cc2)N(CC3)CCC3C	26	13
Z1327803154	CC(C)c(n1)nn(c12)CC(CC2)NC(=O)Nc(sn3)cc3-c4ccccc4	17	7
Z1328888966	FC(F)(F)Oc(cc1)ccc1C(=O)N(S(=O)(=O)C)C2CCC(O)CC2	2	3
Z1329202056	NC(=O)N(C)c1ccc(cc1)OC(C)c2nnc(o2)-c3cc(Cl)ccc3	-16	8
Z1334487248	O=C(O)/C=C/c1cnc(s1)/C=C/c2cc(OC)c(O)c(Br)c2	25	17
Z133704180	CCN(S(=O)(=O)C)C(CC1)CS1(=O)=O	8	5
Z133987698	CC(C)CN(S(=O)(=O)C)C(CC1)CS1(=O)=O	12	6
Z1340434915	COc(cc1)c(CC(=O)O)cc1C(=O)/C=C/c2ccc(Cl)nc2	-2	6
Z1342205464	CCCCNS(=O)(=O)C(=CC1)CS1(=O)=O	9	2
Z1342206502	O=S1(=O)CC(=CC1)S(=O)(=O)N2CCCC2	6	9
Z1343207103	c1cccn(c12)c(nn2)C(C)Nc3cncc(n3)-n(n4)c(C)cc4C	5	11
Z1343239610	c1sccc1C2CCCN2c3ncnc(c34)nc[nH]4	12	2
Z1343261311	c1cccc(c1C)C2CCCN2c3ncnc(c34)nc[nH]4	42	6
Z1343740230	Cn(c1)ncc1NC(=O)c(c2C)c(O)nc(c23)cccc3	23	17
Z1347350562	CC(C)S(=O)(=O)CCS(=O)(=O)Cc1ccc(Cl)cc1	-14	17
Z1347350572	CC(C)S(=O)(=O)CCS(=O)(=O)Cc1ccccc1	2	4
Z1347351122	CC(C)S(=O)(=O)CCS(=O)(=O)C(C)c1ccc(F)cc1	12	20
Z1347351162	CC(C)S(=O)(=O)CCS(=O)(=O)CC1CCCCC1	-3	10
Z1347351377	CC(C)S(=O)(=O)CCS(=O)(=O)c1cc(Br)ccc1	-2	6
Z134825562	c1cccc(c12)nc(-c3c(O)cc(O)cc3)cc2-c4ccccc4	-4	2
Z134829678	c1c(Cl)ccc(c12)nc(-c3c(O)ccc(c3)O)cc2-c4c(Cl)cccc4	25	11
Z135099694	Cc1ccc(cc1)OCCSCc2nc(nc(n2)N)Nc3ccc(F)cc3	9	2
Z135100070	CSc(cc1)ccc1CSCc2nc(nc(n2)N)Nc3ccc(F)cc3	-6	3
Z135163592	c1cccn(c12)cc(n2)COc(cc3)ccc3C(NC4=O)Nc(c45)cccc5	6	4
Z1352068858	c1cc([N+]([O-])=O)ccc1C(=O)OCc2ccc(nc2)-n3ccnc3	7	7
Z135358570	c1c(Cl)ccc(O)c1C(=O)c2cc(n(c2)CC)-c([nH]c3=O)nc(c34)cccc4	7	8

Z1354412989	Cc1ccc(cc1)-c2cc(-c(cc3)ccc3C)nc(c24)cccc4	-11	10
Z1356079428	O=C(O)/C=C/c1cnc(s1)¥C=C¥c2cnnn2C	47	8
Z1356258517	C1NC(=O)N(C12)CCN(C2)C(=O)/C=C/c3ccc(cc3)N4CCOCC4	-3	5
Z136348868	Cc(c1)ccn(c12)cc(n2)C(=O)NCC(N(C)C)c3cc(F)ccc3	50	16
Z136752996	c1cccc(c12)n(cn2)-c(cc3)ccc3C(=O)NN(C(=O)N4)C(=O)C4(C)CCc5ccccc5	9	2
Z137367324	Cc(c1)ccn(c12)cc(n2)C(=O)NCCCn(n3)c(C)cc3C	2	14
Z1387091003	c1cc(C)cc(c12)nc(-c3ccc(Br)cc3)cc2-c4ccccc4	0	4
Z1393197050	NC(=O)c1cc(n[nH]1)C2CCCN(C2)C(=O)Nc3ccc(F)c(F)c3F	-9	6
Z139671368	C1CCCCN1S(=O)(=O)c(c2)ccc(c23)n(CC)c(n3)CNc(cc4)cc(c45)oc(=O)cc5C	-9	5
Z1396757600	O=S1CCN(CC1(C)C)c2nc(nc(c23)CCC3)-c4ccncc4	8	4
Z1396774495	CC1(C)C(C)(OC)CC1N(C)C(=O)NC2CCCn(c23)nc(n3)C(C)C	-1	8
Z1401935735	n1ccnc(c1C#N)N(CC2C)CCN2C(=O)c3cc(ccc3)OCc4ccc(F)cc4	35	12
Z1403244361	CCNC(=O)CN(CC)S(=O)(=O)c(c1C)cnn1-c2ccc(F)cc2	2	7
Z1403432995	CCC(C)CS(=O)(=O)N(CC1(C)C)CCS1(=O)=O	11	5
Z1404550995	CN(C)c(n1)oc(c12)cc(cc2)NC(=O)c3csc(n3)-c4ccoc4	10	9
Z140497072	NC(=O)C1CCN(CC1)C(=O)CNc2c(ccc(Cl)c2)Sc(cc3)ccc3F	-9	8
Z1407063064	s1cncc1-c2nc(N)ccn2	-14	12
Z1409331244	c1c(Br)ccc(c12)nc(-c3cc(OC)ccc3)cc2-c4ccccc4	-16	14
Z1411146082	N#CCN(C(C)C)S(=O)(=O)c(c1S(=O)(=O)C)cccc1	-35	13
Z1415883225	Cc1cc(O)nc(n1)C(C)Sc2ncn(n2)-c3ccccc3	34	16
Z14158957	FC(F)Oc(cc1)ccc1CN(C)C(=O)CN(C(=O)N2)C(=O)C2(C)c3c(Br)cccc3	23	2
Z1415907924	c1ccccc1C(=O)N(CC2)CCC2N(CCC)Cc3c(OC)nccc3	6	10
Z1417129003	n1cccn1CCCOC(=O)c2ccc(cc2)-c([nH]3)nnc3C	10	4
Z1417711005	CNc1snc(C)c1C(=O)N(C2)CCC2Oc(c3)ccc(Cl)c3Cl	-7	5
Z1418841673	C1CC=CCC1CNC(=O)c2cc(cnc2)-c3ccc(cc3)N(C)C	21	5
Z1419493384	OCc1cnc(n1C)SC(C)c2cn(nn2)-c3ccccc3	-8	8
Z1419966801	COC(=O)Cn(nc1)c(c12)ncnc2N3CCCCC3CCC(C)C	1	7
Z1420504830	COCCOC(=O)Nc1cc(ccc1)NCc(o2)ccc2CC	-7	6
Z1421828757	C1CC1c2cc(nn2CC)NCc3c(n[nH]c3)-c(o4)ccc4C	13	5
Z14224841	CN(C)C(=O)c1ccc(cc1)NC(=O)CN(C(=O)N2)C(=O)C2(CC)c3ccc(F)cc3	16	5
Z1423687928	n1ncn(c12)cc(cc2)C(=O)Nc([nH]n3)cc3-c4c(F)c(F)ccc4	11	7
Z1425793872	s1cccc1-c(no2)nc2Cn(nn3)cc3-c4ccccc4	13	15
Z1426135114	c1ccc(C(F)(F)F)cc1C(C)(CO)NCc2ccc(C)cn2	10	13
Z1426521974	c1cc(F)ccc1-c(on2)cc2C(=O)Nc([nH]3)nnc3-c4ccoc4	4	3
Z1430011273	c1c(F)c(C)cc(c1C(=O)N)NCc(n2)cn(c23)cccn3	12	17
Z1430532131	Cc1cnn(c1)-c2cccc(n2)N(CCO3)CC3c4ccc(F)cc4	0	5

Z1430823330	c1c(Cl)cc(Cl)cc1C(N(C)C)C(=O)NCCn2cnnc2C(C)C	5	8
Z1432870591	C1CC1c([nH]n2)cc2C(=O)N(CC3)CC¥C3=C¥c4c(Cl)ccc(Cl)c4	2	3
Z1433031919	O=c1ccccn1CC(=O)N(CC2)CCC2CCN3CCCCC3C	5	6
Z1433129748	c1c(Br)cnc(Cl)c1C(=O)Nc(n(n2)C)cc2C3CCC3	0	8
Z1436023895	o1c(Br)ccc1CN(C)c(n2)nccc2N3CCOCC3	17	29
Z1446648080	s1c(Cl)ccc1C2=NN=C(SC2)Nc3ccccn3	8	8
Z1451063255	NC(=O)c1cc([nH]n1)NC(=O)c2c(nccc2)Nc3cc(F)ccc3	2	4
Z145138304	C1CCCCN1C(=O)CN(CC2)CCN2C(=O)c3c(-c4c(OC)cccc4)nn(c3)-c5ccccc5	15	7
Z145445868	C1CCN1C(=O)c2cc(nc(c23)n(C(C)C)nc3)-c4ccccc4	4	4
Z1455845707	CNC1CCN(C1)C(=O)c(c2)ccc(c23)n(cn3)C(C)C	13	6
Z1457134316	Brc1cccc(F)c1C(=O)NCCC2CCCNC2	-2	7
Z1457890473	NCC1CN(C)CCN1C(=O)c2ccc(cc2)-c(cc3F)cc(F)c3F	13	8
Z1462562385	c1ncccc1-c(n[nH]c2)c2CNc(cc3)cc(F)c3-n4ccnc4	10	3
Z1462566317	CN(C)C(=O)Cc1c(cccc1)NCc2ccc(cc2)-n3ccnc3	13	4
Z1462621305	CS(=O)(=O)Cc1c(cccc1)NCc2ccc(cc2)-n3ccnc3	-8	6
Z1463415737	o1cccc1-c(ocn2)c2C(=O)OCCc3cn[nH]c3	57	11
Z1464424071	Cc1nc(n[nH]1)C2CCCN(C2)C(=O)c3cc(cnc3)-c(c4)ccc(c45)OCC5	-4	7
Z146461966	C1COCCN1c(cccc2)c2CN(C)C(=O)CCc3ccc(cc3)S(=O)(=O)N(CC)CC	-2	6
Z1465442209	C1CCCCN1c(c2C(=O)OC)cc(nc2)N3CCCCC3	0	15
Z146866146	C1COCCN1CC(c2ccc(C)cc2)NC(=O)Cn(c3=O)cnc(c34)sc(c4)-c5ccccc5	6	5
Z146890674	C1COCCN1CC(c2ccccc2)NC(=O)Cn(c3=O)cnc(c34)scc4-c(cc5)ccc5C	4	5
Z147054608	Cc1onc(C)c1COc(ccc2)cc2C(=O)N3CCN(CC3)Cc(n4)cc(=O)n(c45)ccs5	14	7
Z147064724	n1c[nH]c(c12)ccc(c2)C(=O)NCCCOc3c(F)cccc3	0	6
Z1470747510	C1COCCN1CCN(CCC#N)Cc2c(n[nH]c2)-c3c(F)cccc3F	-6	11
Z147118228	C1CCCCN1C(=O)CN(CC2)CCN2C(=O)c3cc(nc(c34)n(C(C)C)nc4)-c5ccccc5	-5	3
Z147119784	C1CCCCN1C(=O)CN(CC2)CCN2C(=O)Cn(c3=O)cnc(c34)scc4-c(cc5)ccc5C	8	8
Z1474089033	[nH]1ccnc1N(CC)C(=O)c(cc2)cc(c23)CC(OC3=O)c4ccccc4	-8	10
Z147580066	CCCCN(C(CC1)CS1(=O)=O)S(=O)(=O)c(c2)ccc(Cl)c2Cl	30	5
Z1481243647	Cc1cc(ccc1)-c([nH]nc2)c2CNc(c3)nc(C)cc3C	1	6
Z1482480186	c1ccc(F)cc1C(C)(C)CNC(=O)c2c(C)[nH]c(c23)ccc(c3)OC	70	4
Z148531038	Cn1c(Cl)cnc1COC(=O)Cc2c(C)n(c(c23)ccc(c3)OC)C(=O)c4ccc(Cl)cc4	0	5
Z1492998619	c1nccn1C(C2C)CN(CC2)Cc3ncc(o3)-c4cc(Cl)ccc4	33	7
Z1494913871	C1CCCn(c12)c(cn2)C(=O)N(CC3)C(C)c(c34)cc(Cl)cc4Cl	13	5
Z1495494068	COC(=O)c(c1)ccc(c12)N(CCC2)C(=O)c3c(O)cnc(c34)cccc4	34	9
Z1499326650	c1nnccc1¥C=C¥c2ccc(cc2)Oc3cnccn3	8	5
Z1500127626	CC1CC(C)CN(CC1)C(=O)c(n2)nn(c23)cc(Br)cn3	1	3

Z1508526295	O=C(O)c1cc(ncn1)¥C=C¥c2ccccc2	21	3
Z150918412	C1S(=O)(=O)CCC1(C)N(C)S(=O)(=O)c(c2)ccc(C)c2C	9	8
Z1509620411	CN(C)C(C)(C)CNC(=O)Cc1c[nH]c(c12)cccc2Br	-4	5
Z1511609753	CC(C)c([nH]n1)cc1C(=O)N(C)C2C(CCCC2)N(C)C(=O)c3cc([nH]n3)C(C)C	5	2
Z1513517715	CS(=O)(=O)CCOCCNc1cncc(n1)-n2ccnc2C	17	5
Z1514764424	n1cc(C)ncc1COC(=O)c2c(Cl)ccc(c2)-c3ccccn3	-4	4
Z1515387931	CC(=O)c1cc(ccc1)COC(=O)c2n[nH]c(c23)CCCC3	-3	24
Z1516256473	c1cc(C)cc(F)c1C(=O)Nc([nH]n2)cc2-c3cccnc3	45	3
Z1517090453	C1CC=CCC1S(=O)(=O)N	1	2
Z151845340	C1CCCCCN1C(=O)C(C)Sc(s2)nnc2NCc3ccc(F)cc3	33	14
Z1519080758	c1ncc(Br)cc1C(=O)NS(=O)(=O)c2cc(Cl)cnc2	-9	7
Z152371002	CC(=O)c1cc(n(C)c1)C(=O)OCC(=O)N(C(C)CC)CC(=O)Nc2cccc(C)c2C	-9	4
Z1527076116	COC(=O)Cn(nc1)c(c12)ncnc2N3CCCC34CCCCC4	-3	11
Z1527076521	Brc1nn(C)c(c12)ncnc2N3CCCC34CCCCC4	38	5
Z152755906	c1ccccc1Cn(c(C)c(C)c2C#N)c2NC(=O)CSc(nnc3C)n3-c(cc4)ccc4C	5	11
Z1532484795	c1ccc(CO)cc1C(C)NCc2ccc(cc2)CN3CCN(C)CC3	-4	8
Z1535762010	FC(F)(F)C(C)(O)CNC(=O)c1cc(-c2cccs2)nc(c13)n(CC)nc3	-4	4
Z1537172697	c1cc(N)nc(c12)ncnc2NC(C)c3ccc(cc3)-n4ccnc4C	53	13
Z1538193381	Fc1cccc(c12)[nH]cc2C(=O)N(CC3)CCC3Nc(n4)cccc4C	3	5
Z154098858	CCOC(=O)c1c(C)oc(c12)ncn(c2=O)Cc3c(F)c(F)ccc3	2	7
Z15431588	c1cc(F)c(F)cc1C(=O)COC(=O)c2c(Cl)ccc(n2)-n(n3)c(C)cc3C	20	16
Z1545018510	CS(=O)(=O)C(C)(C)CN(C)S(=O)(=O)c1c(Cl)cccc1	-10	6
Z1547285504	O=S1(=O)CC(CC1)CNS(=O)(=O)c2c[nH]c(c23)nccc3Cl	-14	7
Z1549038619	CCc1cc(ncn1)N(CC2)CCC2NCc3c(cccc3Cl)N4CCCC4	0	16
Z1549639934	Cc(c1)ccc(c1C)S(=O)(=O)C(C2)C(O)CS2(=O)=O	-1	6
Z1551172092	CC(C)c1cc([nH]n1)NS(=O)(=O)c(c2F)cc(F)c(Br)c2	5	6
Z1556993367	O=C1NCc(c12)cccc2NS(=O)(=O)c3c(Cl)ccnc3Cl	-3	5
Z1557589830	c1n[nH]c(c12)cc(cc2C)NC(=O)N3CCN(CC3)c(cc4C)nc(n4)C	42	14
Z1561183584	c1cc(CN)nc(C)c1C(=O)Nc(cc2)cc(F)c2Oc3c(Cl)cc(Cl)cc3	-5	5
Z1564730073	CC(C)(C)Oc(n1)cccc1CN(CC2)CCC2c3c[nH]c(c34)nccc4	12	2
Z1566941984	c1cc(F)cc(c12)N(C(=O)C(CC3)CS3(=O)=O)CC(C)N(C2)C(=O)OC(C)(C)C	-8	4
Z15674725	c1cccc(c12)nc3c(c2C(=O)OCC(=O)N)CC(C)CC/3=C¥c4ccc(cc4)OC	30	4
Z1567950299	COc(cc1)c(Br)cc1CNc2c(nn(C)c2)-c3ccncc3	-10	2
Z1569249080	o1c(Cl)ccc1CN2CCN(CC2)c3ccnc(n3)N(C)C	27	15
Z1570994914	s1ccnc1NC(=O)C(CC(C)C)Nc2ncnc(c23)cc(Cl)cc3	5	5
Z1572492543	c1ccc(OC)c(OC)c1CN(CCO2)CC2(C)c3cnn(C)c3	-2	1

Z1574547064	O=c1n(C)c(=O)n(C)c(c12)nc(cc2)C(=O)NCc3cc(Cl)ccn3	-2	6
Z1574682602	C1CCCCC1OCC(=O)N2CCN(CC2)Cc(c(n3)C)n(c34)CCCC4	2	3
Z1574815119	CC(C)(C)c1ccc(cc1)NC(=O)/C=C/c2cc(O)cnc2	6	10
Z1576995201	OCCNC1CCN(CC1)c(c([N+]([O-])=O)cc2)cc2C	-9	15
Z15780020	c1ccccc1Cn2c(SCC(=O)C(¥C#N)=C(/C)N)nnc2-c3c(F)cccc3	-17	17
Z1578972593	C1CCCCN1CCn(c2)ncc2Nc(cc3)nn(c34)c(nn4)-c5ccccc5	17	20
Z1579018538	CC(C)(C)OC(=O)N(CC1C)C(C)CN1C(=O)Cc2csc(n2)N(C3=O)CCN3	2	8
Z1583197068	c1ccc(F)cc1C(C)NCc2cc(ncc2)OC3CCOC3	21	16
Z1587266149	CCCn1[nH]cc/c1=N¥C(=O)CN(C)C(C)c2cc(ccc2)-c3ccncc3	17	5
Z15891611	c1cccc(c12)n(c(n2)CC)CC(=O)N3CCN(CC3)S(=O)(=O)c(c4)ccc(c45)cccc5	33	10
Z1590996465	CN(C)c(nc1)ncc1NC(=O)N2CCCCCC2c(c3C)c(C)[nH]n3	-1	4
Z1591866613	OC1CCN(CC1)C(=O)CN(C)S(=O)(=O)c(c2)cnc(Cl)c2C	-1	8
Z1592563027	CN(C)C(=O)c1cc(ccc1)NC(=O)CNC(C(C)C)c2c(C)sc(C)c2	-2	7
Z1593120476	c1cccc(c12)nc(nc2N)NC(C)CN(C)Cc3ccccc3	-10	22
Z1601587562	COCc1nnc(o1)-c(ccc2)cc2-c(on3)nc3-c4cccs4	11	6
Z1601616896	C1COCCN1CC(C)(O)CNc2ccnc(c23)c(F)cc(Br)c3	-21	9
Z1602023618	Cc1cnc(o1)COC(=O)c2cc(cnc2)-c3ccc(cc3)N(C)C	-9	10
Z1603513622	c1ccccc1CC(C(=O)O)CNC(=O)c2ccc(cc2)-n3ccnc3	15	3
Z160742094	COc(nc1)ccc1C(=O)Nc([nH]2)nnc2-c3ccc(F)cc3	4	7
Z16078482	c1cccc(c12)nc([nH]c2=O)SCC(=O)NCCc3ccc(F)cc3	40	14
Z1614098446	FC(F)n1ccnc1CN(C)C(=O)C(C2)CCn(c23)c(nc3)C4CCCCC4	1	3
Z1614685203	c1cc(O)cc(F)c1C(=O)N(C)CCCc(n2)[nH]c(c23)cccc3	9	4
Z16158532	Cc1c(C)ccc(c1)S(=O)(=O)NCC(=O)OCc2csc(n2)Nc3ccc(cc3)OC	-1	4
Z1616590793	N#Cc1ccc([nH]1)C(=O)OCc2ccc(Br)cn2	9	7
Z1618388683	c1c(F)c(C)cc(c1C(=O)N)NCc2nnc(C)n2C	-35	11
Z16202306	c1ccccc1-c2ccc(cc2)NC(=O)CN3C(=O)NC(C3=O)Cc4c[nH]c(c45)cccc5	6	4
Z1624201749	s1cccc1C(=O)N(CC2)CCC2c3c[nH]c(c34)ccc(c4)C(=O)N	-35	9
Z16253844	C1COCCN1c(c(c2)OCC)cc(OCC)c2NC(=O)CSc(n3-c4ccccc4)nnc3-c(c5)ccc(c56)OCO6	-3	7
Z16254213	Cc(c1)n(C)c(C)c1C(=O)CSc(n2-c3ccccc3)nnc2-c(c4)ccc(c45)OCO5	6	6
Z16254229	[nH]1cccc1C(=O)CSc(n2-c3ccccc3)nnc2-c(c4)ccc(c45)OCO5	7	17
Z16254794	CN(C)c1ccc(cc1)NC(=O)CSc(n2-c3ccccc3)nnc2-c(c4)ccc(c45)OCO5	-7	11
Z16256913	c1cccc(C)c1C(C)NC(=O)CSc(n2)[nH]c(c23)ccc(Cl)c3	4	11
Z16257955	c1ccc(C)c(c1C(C)C)NC(=O)CSc(n2)[nH]c(c23)cc(Cl)cc3	4	6
Z1626134934	Fc1cccc(c1C#N)N(C2)CCCC2c(n[nH]c3)c3NS(=O)(=O)C	-3	4
Z1626977832	CN(C)C(=O)N(CC1)CCC1c2nc(no2)C3CCCCCN3	-4	9
Z1629496420	c1cnccc1C(=O)N2CCN(CC2)C(C3=O)CCCN3c(n(n4)C)cc4C	17	8

Z16316201	Cc1onc(C)c1CSc2ncnc3sc(c4c23)CC(C)CC4	-8	10
Z1632805805	n1ccncc1CCN(C)Cc2c(OC)cc(cc2Br)OC	7	9
Z1633590863	c1ccc(O)cc1C(=O)NCC2CC(C)N(C2)Cc3ccccc3	1	6
Z1633934253	c1ccnc(c1C#N)NCCOC(=O)c(c2)cnc(c23)n(C)c(=O)[nH]c3=O	-1	10
Z1634598040	c1c(Br)c(O)c(OC)cc1¥C=C¥C(=O)N2CCCC2c(c3C)c(C)n(n3)C	41	4
Z1638295320	Cn1nc(C(C)C)cc1C(=O)N(C2)CC2S(=O)(=O)NC3CCCC3	5	4
Z1642148240	c1ncc(O)cc1¥C=C¥c2nc(no2)-c(cc3)cc(F)c3C	-4	19
Z1642148255	c1ncc(O)cc1¥C=C¥c2nc(no2)-c3ccc(cc3)OC	3	13
Z1642148338	c1ncc(O)cc1¥C=C¥c2nc(no2)-c3ccc(nc3)Oc4ccc(F)cc4	-13	7
Z1642185485	Cc(c1)ccc(c1CN(C)C)NC2CCN(CC2)Cc3ccncc3	2	8
Z16426742	c1cc(S(=O)(=O)N)ccc1CCNC(=O)CSc(nnc2-c3c(F)cccc3)n2CCc4ccccc4	-1	3
Z16427126	c1cccc(F)c1-c2nnc(SCC(=O)c3ccc[nH]3)n2CCc4ccccc4	-9	6
Z1646916001	CC(C)c1ccc(cc1)C(C2)C2C(=O)Nc(cc3)cc(F)c3NC(=O)OC	7	7
Z164780402	C1CC(=O)N(C)N=C1C(=O)N2CCN(CC2)S(=O)(=O)c(c3C(F)(F)F)cccc3	7	8
Z164941068	C1CC(=O)Nc(c12)ccc(c2)C(=O)Nc3nnc(s3)SCc4c(Cl)cccc4Cl	-3	7
Z164951688	c1ccc(F)cc1CC(=O)NCc2ccc(o2)-c3csc(n3)C	-7	7
Z164965524	O=C1CC(C)(C)Cc(c12)[nH]c(c2C)C(=O)N3CCN(CC3)C(=O)C(CC4)CS4(=O)=O	25	19
Z165003518	CCN(CC)S(=O)(=O)c(cc1)ccc1C(=O)NC2CCSc(c23)c(F)ccc3	12	4
Z165050208	CC(=O)c1cc([nH]c1)C(=O)NCCc2ccc(s2)-c3csc(n3)C	3	5
Z1651676792	NC(=O)C(C)Nc1ccc(cc1)Oc2cccc(c23)cccn3	6	4
Z165229378	c1cccc(Cl)c1NC(=O)CN2CCN(CC2)c(nc(c34)cccc4)cc3C(=O)N5CCOCC5	39	6
Z165261492	C1CCCCN1C(=O)CN2CCN(CC2)c3nc(-c4ccccn4)nc(c35)scc5-c6ccccc6	13	6
Z165261498	C1CCCCN1C(=O)CN2CCN(CC2)c3nc(-c4cccnc4)nc(c35)scc5-c6ccccc6	9	6
Z1654002148	c1cccc(F)c1OCC(=O)NCc(c2)ccc(c23)[nH]c(=O)[nH]3	3	7
Z16580596	CC1CCCCN1C(=O)CSc(n2-c3ccccc3)nnc2-c4cc(ccc4)S(=O)(=O)N(CC)CC	0	5
Z1658298345	CC(O)C1CCN(C1)C(=O)NCC2CC(C)N(C2)Cc3ccccc3	-9	6
Z165885974	COc(cc1)cc(c1C(=O)N)OCc2cc(C)cc(C)c2	4	3
Z16593263	c1cccn(c12)cc(n2)C(=O)OCCOc3c(Cl)cccc3	0	8
Z166077836	NC(=O)c1cc([N+]([O-])=O)c(cc1)COC(=O)CCc(cc2)nc(c23)cccc3	-4	7
Z166077876	c1cccc(c12)nc(cc2)CCC(=O)OCCOc3ccc(cc3)S(=O)(=O)N4CCOCC4	-20	12
Z1661110236	NC(=O)N(C1)CCCC1Nc2nc(-c3cc(Cl)ccc3)nc(c24)CCCC4	2	5
Z1661117530	CC(C)(C)n(nc1)c(c12)ncnc2NCCc(nn3)n(c34)CCCC4	7	17
Z1661333590	Ic1cccc(c12)ncnc2NC3CCC(O)CC3	10	6
Z1661416188	FC(F)(F)CN(C1)CCC1CNc2ncnc(c23)cc(F)c(Br)c3	9	6
Z16616292	[nH]1c(=O)[nH]c(c12)ccc(c2)NC(=O)COC(=O)CSc(c3)ccc(C)c3C	-13	13
Z166256344	CNc1snc(C)c1C(=O)OCc2csc(n2)-c3ccc(F)cc3	-7	3

Z166257002	c1cccc(c12)[nH]cc2C(=O)C(C)OC(=O)c3c(C)nsc3NC	5	26
Z166362686	c1ccccc1N(CCC(=O)N)C(=O)CSc(nc2)n(c23)cccc3	55	6
Z1664164245	Cc1ccc(cc1)-c2cc(-c3cc(OC)ccc3)nc(c24)cccc4	-18	3
Z16658902	c1ccc(C)c(c1C(C)C)NC(=O)CSc(n(c2=O)CC(C)C)nc(c23)ccc(Cl)c3	-5	11
Z166605136	c1ccccc1-c2c(O)c(-c3ccccc3)nc(c24)ccc(Cl)c4	8	3
Z166605882	Cc(c1)cc(C)cc1C(=O)N(C(C2)C=CS2(=O)=O)c(c3)cc(C)cc3C	34	14
Z166704886	CC1CCC(CC1)NS(=O)(=O)c(c2)ccc(c2[N+]([O-])=O)N(C3)CCCC3c(nn4)n(c45)cccc5	5	7
Z166707558	N#Cc1c(N)sc(c12)CN(CC2)c(c3[N+]([O-])=O)ccc(c3)S(=O)(=O)N(CC)CC	1	4
Z1667221615	CCCCn(c1Cl)nc(C)c1C(=O)N(CC2)CCC2(C)CN3CCN(C)CC3	29	5
Z166811416	c1cccc(Cl)c1C(N2CCCC2)CNC(=O)CCc3ccc(cc3)S(=O)(=O)N4CCCC4	-3	9
Z166819076	c1cccn(c12)cc(n2)CNC(=O)c(cc3)cc(c34)[nH]c(=O)n(c4=O)-c5ccccc5	14	5
Z1669230171	n1c[nH]c(c12)nc(cc2)C(=O)NC(C)c3ccc(cc3)-c4cncnc4	8	4
Z166962724	C1COCCN1CCCNC(=O)c2cc(-c3ccccc3)nc(c24)n(nc4)Cc5cc(F)ccc5	7	7
Z167063860	c1cccc(c12)nc(cc2)NC(=O)Cc3coc(n3)-c4ccccc4	-6	9
Z1671371372	CC(C)(C)OC(=O)N(C1)CC(C1C#N)NC(=O)c(c2)[nH]c(c23)cc(F)cc3C	6	5
Z16715300	c1cccn(c12)cc(n2)COC(=O)c(c3)ccc(c34)C(=O)N(C4=O)CC=C	29	24
Z167254296	C1CCCCN1CCCNC(=O)c2cc(-c3ccccc3)nc(c24)n(nc4)Cc5ccncc5	-28	8
Z167364352	CC(C1)Oc(c12)c(OC)cc(c2)C(=O)Nc(c3)ccc(c34)OC(C)C(=O)N4	6	3
Z1675157483	CC(=O)[C@@H](C1(C)C)C[C@@H]1CC(=O)NCCc2ccn(n2)-c3ccc(F)cc3	5	12
Z1675288656	c1ccccc1CN2CCN(CCC2C)c3nc(N)nc(c34)nc[nH]4	48	10
Z1677743628	c1cncc(F)c1NC(=O)NCc([nH]2)ncc2-c3ccccc3	11	4
Z1677751393	c1cncc(F)c1NC(=O)NCCCc2c[nH]c(c23)cccc3	19	3
Z1677754180	c1cncc(F)c1NC(=O)NCCN(CC2)Cc(c23)cccc3	0	4
Z1677761301	c1cncc(F)c1NC(=O)NCc2cnn(c2)Cc3ccccc3	4	2
Z1682981267	C1CN(C)CCC1C(C)N(C)Cc2c(Br)ccc(c2)OC	2	10
Z168616264	n1c[nH]c(c12)ccc(c2)C(=O)NCCCOc(cc3C)ccc3	-4	2
Z1686719326	c1cc(F)ccc1C(C(=O)N)Nc2cnn(c2)Cc3ccccc3	0	18
Z168726218	N1C(=O)NC(C1=O)CCC(=O)Nc2ccc(cc2)Oc3c(Cl)cccc3	11	4
Z1687428428	n1cc(C)nc(c1C)NCc2cc(ccc2)Cn3ccnc3C	9	7
Z168757250	C1CCc(c12)ccc(c2)C(=O)N(CC3)Cc(c3c45)sc4ncnc5N	4	2
Z168801896	CS(=O)(=O)c(cc1)cc(S(=O)(=O)C)c1N2CCCC2	13	5
Z1688195829	n1cc(C)ncc1CCSc(o2)ncc2-c3ccc(F)cc3	15	12
Z1688689058	CN(C)c(nc1)ccc1NC(=O)N(C)C2CS(=O)(=O)c(c23)ccc(Cl)c3	-9	5
Z168931562	N=c(n1)[nH]c(N(C)C)nc1COc(c2)ccc(c23)cc(Br)cc3	5	13
Z168975100	Nc(n1)nc(N(C)C)nc1COc(c2)ccc(c23)ccc(c3)OC	6	4
Z1694072123	CC(C)(C)OC(=O)c1ccc(cc1)CNc(ccc2)cc2-c3nnc[nH]3	-11	15

Z169558548	C/C(C)=C/C1C(C1(C)C)C(=O)Nc(c2C)c(C)cc(C)c2	2	4
Z1695663536	Cn1ccnc1C(=O)c2cc([N+]([O-])=O)c(cc2)N(CC3)CC=C3c(c4C)cncc4	7	2
Z1697043335	OCCc(c1C(F)(F)F)cn(n1)Cc2nnc(s2)-c3ccccc3	-6	3
Z1699207489	n1ccccc1CNC(=O)CCNc2ncnc(c23)n(C)nc3	-11	3
Z1699229618	[nH]1cnc(c12)nc(N)nc2N(CC3)CC=C3c4c(F)cccc4	60	7
Z169940730	Cc(c1)sc(C)c1C(=O)CCC(=O)Nc(n2)sc(c23)CN(C)CC3	5	3
Z1702883776	CC(C)COCCCNS(=O)(=O)CCCS(=O)(=O)N(C)C	-12	25
Z17093922	Cc1cc(no1)NC(=O)COC(=O)c(c2)ccc(c23)[nH]c(C)c3C	-13	7
Z17094388	Cc1onc(C)c1COC(=O)c(c2)ccc(c23)[nH]c(C)c3C	4	6
Z17101116	c1c(Cl)cnc(c12)[nH]c(n2)SCC(=O)N(C(C3)c4ccco4)N=C3c5ccc(cc5)OC	24	14
Z1720728610	CN(C)S(=O)(=O)CCCS(=O)(=O)N(C)C1CCC1	-6	7
Z1722673239	n1cncn1-c(cc2)ccc2CNc(no3)nc3-c4ccccc4	-10	7
Z1723428243	Cn1ccnc1C(C(F)(F)F)(O)CCNC(=O)c2cc(ccc2)-c(c3)cnc(c34)[nH]c(=O)n4C	17	6
Z1728621325	N#Cc1ccnc(n1)¥C=C¥c(ccc2)cc2C(=O)O	-2	5
Z1728621359	O=C(O)c1cc(ccc1)/C=C/c2ccnnc2	-9	5
Z1728623559	n1ccncc1C(¥C)=C/c(c2)ccc(c23)nccc3	2	6
Z17289317	S1CCSC1c2ccc(cc2)OCC(=O)Nc(cc3)ccc3C(=O)Nc4c(OC)cccc4	1	8
Z1735017324	c1cc(O)cc(F)c1C(=O)NCC2CC(C)N(C2)Cc3ccccc3	2	1
Z17356722	c1cccn(c12)cc(n2)CSc([nH]3)nnc3-c4c(F)cccc4	0	9
Z1737110153	Cn1cncc1CC(=O)Nc(c2)ccc(c23)[nH]c(n3)-c4c(F)ccc(F)c4	6	9
Z1739334569	c1ncc(O)cc1¥C=C¥c2nc(no2)-c3ccccn3	11	11
Z1739342675	c1ncc(O)cc1¥C=C¥c2nc(no2)-c3ccncc3	8	6
Z1739489055	CC(C)Oc(ncc1)cc1CN(C2CC2)c3ncnc(c34)n(C)nc4	3	6
Z1740429081	c1c(O)cc(O)cc1C(=O)NCC2CC(C)N(C2)Cc3ccccc3	3	5
Z1742902065	C1COCCN1C(=O)C2CCCN(C2)C(C)c3cc(F)c(cc3)SC	26	12
Z1743524859	CNC(=O)c1cccc(n1)NCc2c(O)ccc(C)c2	37	7
Z1743534706	CN(C)C(=O)c1cc(ncc1)NCc(cc2)cc(c23)[nH]nc3	-11	3
Z1746485864	n1cccc(O)c1C(=O)Nc(cc2)ccc2Cn3ccnc3C	55	9
Z1765170433	CNc(c1Br)nc(nc1)NC2C(OCC2)c3cc(F)c(F)cc3	3	6
Z1769013356	C1CCCc(c12)cc(O)c(c2)C(=O)N(CC3)CCC3(C)NC(=O)OC(C)(C)C	-20	8
Z1784719533	CC(C)n1cc(Br)cc1C(=O)N2CCN(CC2)c(n[nH]3)cc3C	-5	6
Z1787994415	COCC(C#C)NS(=O)(=O)CCCS(=O)(=O)C	4	5
Z17893104	COCCCn1c(=O)c(cccc2)c2n(c13)c(nn3)SCc(n4)cn(c45)cccc5	-35	7
Z17895832	CC1C(C)CCCC1NC(=O)NC(=O)CSc(nn2)n(c23)c4c(cccc4)c(=O)n3CCCCC	-5	1
Z17964966	C=CCNc(n1)scc1C(=O)OCc2csc(n2)-c3ccccc3	18	6
Z17965676	C=CCNc(n1)scc1C(=O)OCCCn2c(=O)[nH]c(c23)cccc3	5	5

Z1798299891	c1ccccc1-c(on2)cc2CNc3cncc(n3)-n4ccnc4C	19	6
Z1798329942	CCNC(=O)c1ccc(nc1)NC(C)C(O)c2ccc(F)cc2	35	8
Z18025700	CC(C)C(C#N)(C)NC(=O)C(C)OC(=O)c1cc(OC)c(cc1)Oc(c2[N+]([O-])=O)ccc(Cl)c2	1	6
Z1820642882	O=C1CCCCN1C(C)CNC(=O)c2c(O)cn(n2)-c(cc3)ccc3C(C)C	-3	11
Z18212324	C1CCc(c12)ccc(c2)NC(=O)CSc(c3)ccc(c34)OCCCO4	14	6
Z1822689954	CC(C)S(=O)(=O)CCCS(=O)(=O)c(c1C)cco1	-9	4
Z1823608663	[nH]1ccnc1C(O)C2CCN(CC2)c(n3)ccc([N+]([O-])=O)c3-c4ccccc4	-51	10
Z18264769	c1cccc(c12)NC(/N2)=C(¥C#N)C(=O)COC(=O)CCc3ccccc3	5	6
Z1833465604	c1ccccc1N(C2CCN(C)CC2)S(=O)(=O)c(cc(Cl)c3)c(c34)OCC4	9	14
Z1833478806	N#Cc1cc(ncc1)N(C)S(=O)(=O)c(c2S(=O)(=O)C)cccc2	7	4
Z183473124	Clc1cccc(Cl)c1S(=O)(=O)N(CC2)CCN2C(=O)NCCCN3CCCCCC3=O	-3	6
Z1837787694	N#Cc1cc(cn1C)-c(n2)n(C)c(c23)ccc(c3)S(=O)(=O)NC	-2	13
Z18387854	C1CCc(c12)ccc(c2)NC(=O)COC(=O)c3c(cccc3)OCc4ccccc4	26	15
Z18432818	c1cc(Cl)ccc1CSCc2nc(nc(n2)N)Nc(c3C)cccc3	6	5
Z1844441844	c1cccc(c12)nc(-c3cc(OC)ccc3)cc2-c4ccc(Cl)cc4	48	23
Z1844441892	c1cccc(c12)nc(-c(cc3C)ccc3)cc2-c(cc4)ccc4C	9	4
Z184538526	c1cccc(c12)[nH]c(n2)NC(=O)c3cc([nH]n3)-c4ccccc4	3	7
Z18459341	c1cccc(Cl)c1SCc2nc(nc(n2)N)Nc(c3C)cccc3	17	3
Z1847669462	Cc1ccc(cc1)-c2cc(-c3cc(Br)ccc3)nc(c24)cccc4	0	7
Z18519048	c1cccc(c12)[nH]c(-c3ccccc3)c2C(=O)CSc4nnnn4C	0	9
Z18519758	O1COc(c12)ccc(c2)NC(=O)C(C)Sc3nnnn3C	5	2
Z185334464	c1cccc(c12)OCC(O2)CNC(=O)c3cc(ccc3)OCc(n4)cn(c45)cccc5	-12	11
Z18537178	Cc(c1)c(C)cc(C)c1C(=O)COc2ncnc(c23)cccc3	7	3
Z18537695	c1ccc(OC)cc1NC(=O)COc2ncnc(c23)cccc3	-1	10
Z18537891	FC(F)(F)Cn(c(C)c1)c(C)c1C(=O)COc2ncnc(c23)cccc3	6	4
Z185383718	Cn1ccnc1C(c2ccccc2)Nc(c3Cl)cnn(c3=O)Cc4ccccc4	30	26
Z185587852	Cc1nc(nc(n1)N)Nc2ccccc2	-16	6
Z18607575	NC(=O)c1cc(C(=O)N)cc(c1)NC(=O)COC(=O)c2c(cccc3)c3nc(c24)¥C(CC4)=C¥c5c(Cl)ccc c5	10	7
Z18614891	C1CCCCC1NC(=O)NC(=O)C(C)Sc(n2c3CCCCC2)nc(c34)nn(c4=O)-c5ccccc5	7	4
Z1862067853	Clc1cccc(SC(C)C)c1/C=C/c([nH]c2=O)nc(c23)cncc3	1	2
Z1863492097	O=C(O)C1CCN(CC1)c2ccc(nn2)Sc(cc3)ccc3C(C)C	1	4
Z1863649257	C#CCN(C)S(=O)(=O)c1c(Cl)sc(c1)S(=O)(=O)C	-18	9
Z18647134	Cc1cc(C)cc(C)c1C(=O)CSc(n2CC3CCCO3)nnc2-c4c[nH]c(c45)cccc5	-1	8
Z18647898	CN(C)c1ccc(cc1)NC(=O)C(C)Sc(n2CC3CCCO3)nnc2-c4c[nH]c(c45)cccc5	0	7
Z18650720	C1CCc(c12)ccc(c2)NC(=O)CSc(o3)nnc3-c4ccco4	15	4

Z18666705	C1COCCN1c(cc2)ccc2NC(=O)CSc(n3-c(c4C)ccc(C)c4)nnc3-c5cccnc5	54	5
Z18672879	CCOc(cc1)ccc1-n2c(SCC(=O)c3ccc[nH]3)nnc2-c4c(Cl)cccc4	7	9
Z18712040	c1cccc(c12)CCCC2NC(=O)COC(=O)c3c(cccc3)NS(=O)(=O)c(cc4)ccc4F	-7	1
Z18738204	c1cccc(c12)NC(/N2)=C(¥C#N)C(=O)COC(=O)CSc(c3C)ccc(Br)c3	43	3
Z18771813	c1cccc(c12)NC(/N2)=C(¥C#N)C(=O)COC(=O)c3c(Cl)cc(F)c(F)c3	8	11
Z18791966	CNC(=O)COC(=O)c1cc(nc(c12)cccc2)-c(c3)ccc(c34)cc(cc4)OC	4	5
Z18792577	Cc1cc(ccc1)NC(=O)COC(=O)c2cc(nc(c23)cccc3)-c(c4)ccc(c45)cc(cc5)OC	-12	7
Z18792611	CCN(CC)S(=O)(=O)c(c1)ccc(Cl)c1NC(=O)COC(=O)c2cc(nc(c23)cccc3)-c(c4)ccc(c45)cc(c c5)OC	-19	7
Z18799147	c1cc(Cl)ccc1C2=NN(C(C2)c3ccco3)C(=O)COC(=O)c4cc(-c5ccco5)nc(c46)cccc6	0	4
Z18838070	c1ccnc(SC)c1C(=O)OCC(=O)N(C(C2)c3ccccc3)N=C2c(c4)ccc(c45)cccc5	9	5
Z18885559	NC(=O)COC(=O)c1c(nccc1)Nc(cc2C(F)(F)F)ccc2	6	3
Z188949814	O=C1CCCc(c12)[nH]c(c2C)C(=O)NCCN(CC)c(cc3C)ccc3	-5	5
Z19092990	NC(=O)c1cc(C(=O)N)cc(c1)NC(=O)COC(=O)c2c(C)c(CC)nc(c23)cccc3	2	5
Z1911236831	C#CC(C)(C)N(C)S(=O)(=O)c(cc1)ccc1S(=O)(=O)C	-10	7
Z191288442	CC(C)(C)C1CCC(CC1)C(=O)N(CC2)CCC2C(=O)N3CCCCCC3	7	4
Z19182299	c1cccc(Cl)c1NC(=O)CN(C)C(=O)COC(=O)c2c(CC(C(C)(C)C)CC3)c3nc(c24)cccc4	-2	9
Z192830436	CC(C)n(nc1)c(c12)nc(C)c(c2)C(=O)Nc(s3)nc(c3C)-c4ccc(F)cc4	6	4
Z19314901	Cc1c(F)cc(cc1)NC(=O)CSc2ncnc(c23)cccc3	4	8
Z19316042	C1CC(C)CC(C1C(C)C)OC(=O)CSc2ncnc(c23)cccc3	0	2
Z19316082	c1cc(Cl)cc(c1C#N)NC(=O)CSc2ncnc(c23)cccc3	18	4
Z19326161	c1ccccc1CNc2nnc(s2)SCc3nc(nc(n3)N)Nc(c4C)cccc4	1	7
Z193270258	Cc1onc(C)c1CCC(=O)Nc(n2)n(Cc3ccco3)c(c24)ccc(C(F)(F)F)c4	0	9
Z1936477144	CC(C)(C)S(=O)(=O)c1ccc(cc1)S(=O)(=O)N	2	3
Z1936607769	n1cncc(Br)c1C(=O)NCC(C)CN2CCN(C)CC2	-11	7
Z193722710	Nc(n1)nc(N(C)C)nc1CSc(n2C)nnc2-c3ccccc3	6	5
Z193785446	c1cc(F)ccc1NC(=O)C(CC)Sc([nH]2)nnc2C(C)C	-8	4
Z19394488	c1cc(Cl)ccc1NC(=O)c2c(sc(c23)CCCC3)NC(=O)COC(=O)c4c(c5nc(c46)cccc6)CCCC/5=C ¥c7ccc(cc7)OC	7	4
Z193998480	CN(C)C(=O)COC(=O)c1cc(-c2ccccc2)nc(c13)n(nc3)Cc4cc(F)ccc4	3	7
Z193999678	n1oc(C)cc1COC(=O)c2cc(-c3ccccc3)nc(c24)n(nc4)Cc5cc(F)ccc5	-4	3
Z19446050	C1COCCN1c(cc2)ccc2NC(=O)CSc(n3-c(c4C)cccc4)nnc3-c5ccncc5	-3	4
Z19449863	c1cc(F)ccc1C(C)Sc([nH]n2)nc2-c3cccs3	-5	5
Z19449989	s1cccc1-c2nnc([nH]2)SCC(=O)Nc(cc3)ccc3-c4ccccc4	4	5
Z19450299	c1cccc(c12)[nH]cc2C(=O)C(C)Sc([nH]3)nnc3-c4cccs4	17	13
Z19451548	c1cccc(c12)nc([nH]c2=O)C(C)Sc([nH]3)nnc3-c4cccs4	2	3

Z19511505	c1cc(Cl)ccc1-n2c(SCC(=O)N(C3=O)CCN3)nnc2-c4c[nH]c(c45)cccc5	13	10
Z19512617	C1CCCN1C(=O)CSc(n2-c3ccc(Cl)cc3)nnc2-c4c[nH]c(c45)cccc5	7	13
Z19513253	c1cccc(c12)[nH]cc2C(=O)CSc(n3-c4ccc(cc4)OCC)nnc3-c5c[nH]c(c56)cccc6	1	5
Z19513456	O=C1CCCN1C(=O)CSc(n2-c3ccc(cc3)OCC)nnc2-c4c[nH]c(c45)cccc5	7	5
Z195395734	Cc(c1)ccc(C)c1C(=O)CCC(=O)NC(C)c(n2)[nH]c(c23)cccc3	1	16
Z19539846	c1cccc(c12)nc3c(c2C(=O)OC)CCCC/3=C¥c(c4)ccc(c45)OCO5	-2	9
Z195846234	c1ccc(F)cc1-n(c(C)c(C)c2C#N)c2NC(=O)CN3CCN(CC3)CC(=O)N4CCCCC4	0	4
Z195913274	Cc(c1)ccc(c1C)NC(=O)c(c2C)sc(c23)nc([nH]c3=O)CN(CC4)CCN4Cc5ccco5	6	2
Z195914778	c1ccccc1Cn(c(=O)[nH]c2=O)c(c23)nc(n3CCC(C)C)CN(CC4)CCN4Cc5ccco5	-3	7
Z196146788	CC(C)CNC(=O)CN1CCN(CC1)c2nc(-c3ccccn3)nc(c24)scc4-c5ccccc5	2	8
Z196157908	CC(=O)N(CC1)CC=C1c2ccc(cc2)Oc3nc(-c4cccnc4)nc(c35)cccc5	-13	4
Z196352976	c1cccc(c12)nc3c(c2C(=O)O)CN(C)CC¥3=C¥c4ccc(cc4)SC(F)(F)F	-14	2
Z196370782	Cc(s1)cn(c12)c(c(n2)C)¥C=C¥C(=O)c3ccc(cc3)OCC(=O)N(C)C	-2	7
Z19641461	c1ccccc1CC2CCN(CC2)C(=O)COC(=O)c3c(cccc3)NS(=O)(=O)c4cccs4	-9	8
Z19649872	c1cccc(c12)[nH]c(n2)SCC(=O)NC(=O)NCc3ccccc3	7	3
Z19654122	c1cccc(c12)sc(n2)SCC(=O)Nc3cccc(c34)C(=O)c5c(C4=O)cccc5	6	6
Z19678477	CCc1cc(ccc1)NC(=O)COC(=O)Cc2cccc(c23)cccc3	7	5
Z19690549	c1cccc(c1c23)n(CC)c2ccc(c3)NC(=O)COC(=O)c4c(CCCC5)c5nc(c46)cccc6	1	5
Z19690670	c1ccccc1N(C(C)C)c2ccc(cc2)NC(=O)COC(=O)c3c(CCCC4)c4nc(c35)cccc5	10	11
Z19728700	c1cc(Cl)ccc1SCc2nc(nc(n2)N)Nc(c3C)cccc3	-5	5
Z197452730	Clc1cccc(c1Cl)NC(=O)CSCc2nc(N(C)C)nc(n2)N	4	5
Z197453168	c1cccc(F)c1OCCSCc2nc(N(C)C)nc(n2)N	9	21
Z197453372	c1cc(F)ccc1OCCSCc2nc(N(C)C)nc(n2)N	-8	11
Z197454120	C1CC1n(c(C)c2)c(C)c2C(=O)CSCc3nc(N(C)C)nc(n3)N	4	9
Z197454476	c1cc(F)cc(F)c1NC(=O)C(C)SCc2nc(N(C)C)nc(n2)N	-5	10
Z197454478	c1c(F)ccc(F)c1NC(=O)C(C)SCc2nc(N(C)C)nc(n2)N	15	11
Z197454502	C1CCCCCC1NC(=O)C(C)SCc2nc(N(C)C)nc(n2)N	1	3
Z197454542	C1CCc(c12)ccc(c2)NC(=O)C(C)SCc3nc(N(C)C)nc(n3)N	5	7
Z19751109	c1ccccc1SCc2nc(nc(n2)N)Nc(c3C)cccc3	28	12
Z197873940	Cc(c1)ccc(OC)c1C(C)NC(=O)CCc(n2)[nH]c(c23)cccc3	60	10
Z1980444406	c1ccccc1-c2c(CN3CCCCC3)c(O)nc(c24)cc(Cl)cc4	1	4
Z19811005	C1CCCCN1C(=O)COc(c(Br)cc2Br)c(c23)nccc3	5	13
Z19829052	c1ccccc1-c2ccc(cc2)OCC(=O)Nc(cc3)ccc3C(=O)Nc4c(OC)cccc4	3	4
Z19837406	C1COCCN1CCCn(c2=O)c(nc(c23)cccc3)SCc4nc(nc(n4)N)Nc(c5C)cccc5	-7	5
Z19837911	c1cccc(c12)ccc(c2)NC(=O)CSc(nc(c34)cccc4)n(c3=O)CCCN5CCOCC5	46	4
Z19871234	C1CCCCCN1C(=O)CSc(n2-c3ccc(cc3)OC)nnc2-c4ccc(Cl)cc4	2	12

Z198743700	c1ccccc1OCCn(c2=O)ncc(c2Cl)Nc3c(OC)ccc(c3)[N+]([O-])=O	7	3
Z199055452	c1cccc(O)c1C(=O)c(c2)cnn(c23)c(N)nc3-c4ccccc4	7	8
Z199055460	c1ccccc1-c2c(O)c(-c3ccc(Cl)cc3)nc(c24)cccc4	-6	5
Z199499666	c1nccc(c12)ccc(O)c2CN(C)Cc3ccc(cc3)N4CCOCC4	-18	11
Z199538208	CS(=O)(=O)c(cc1)cc(S(=O)(=O)C)c1N2CCCCC2	33	10
Z199538264	OCCN(C)c1c(S(=O)(=O)C)cc(cc1)S(=O)(=O)C	3	16
Z199538358	CCCCN(C)c1c(S(=O)(=O)C)cc(cc1)S(=O)(=O)C	8	5
Z199620098	c1cccc(c12)n(c(n2)C)CCNc3c(S(=O)(=O)C)cc(cc3)S(=O)(=O)C	-19	6
Z199628344	N#Cc1ccc(cc1)S(=O)(=O)N(CC2)CCN2c3c(S(=O)(=O)C)cc(cc3)S(=O)(=O)C	26	6
Z19982748	C1COCCN1C(=O)CSc(n2-c(cc3)ccc3C)nnc2-c4cc(OC)ccc4	27	7
Z20151781	c1cccc(c12)CN(CC2)C(=O)CSc(n3-c4ccccc4)nnc3COc5c(Cl)cccc5	-5	5
Z20162836	c1cccc(c12)[nH]cc2CC(C(=O)O)NC(=O)CSc(s3)nnc3Nc(c4C)c(C)ccc4	3	4
Z20227630	Cc1ccc(cc1)NC(=O)C(C)Sc2ncnc(c2c34)sc3CCC4	-9	6
Z20229401	Clc1cnc(c(Cl)c1C)NC(=O)CSc2ncnc(c23)sc(C)c3C	-1	8
Z20241270	c1cccn(c12)c(nn2)SC(C)C(=O)Nc(n3)scc3-c4cc(Cl)c(Cl)cc4	4	4
Z20241790	c1cccn(c12)c(nn2)SC(C)C(=O)Nc(n3)scc3-c4cc(F)c(F)cc4	21	14
Z20243594	C1CCCC1NC(=O)NC(=O)CSc(cc2C)nc(c23)cccc3	8	4
Z20243606	c1cccc(F)c1-n(c(C)c2)c(C)c2C(=O)CSc(cc3C)nc(c34)cccc4	5	13
Z203056244	c1cc(C)ccc1C(CC)NC(=O)c2cc(C)nc(c23)n(C(C)C)nc3	-5	5
Z203177000	COc1ccc(cc1)S(=O)(=O)N[C@@H](C)C(=O)N(CC2)CC=C2c3c(OC)cc(OC)cc3OC	0	2
Z203269322	Fc1c(F)ccc(c1)NC(=O)c2cc(C)nc(c23)n(C(C)C)nc3	39	3
Z203909940	c1cccc(c1C#N)CSCCNc(n2)cc(=O)n(c23)cccc3	-8	4
Z203963094	CC(C)c1cc(ccc1)NC(=O)CN(C)CC(=O)N2C(C)CC(=O)Nc(c23)cccc3	-10	10
Z204253774	c1cccc(c12)n3c(n2)N(CC3)C(=O)c4cc(c(Cl)cc4)S(=O)(=O)N5CCCCC5	-4	4
Z204341220	C1CCc(c12)sc(c2)C(=O)Nc(c3C(=O)O)cc(s3)-c4cc([N+]([O-])=O)ccc4	-3	5
Z204341820	o1cccc1¥C=C¥C(=O)Nc(c2C(=O)O)cc(s2)-c3cc([N+]([O-])=O)ccc3	-3	3
Z204341942	c1ccc([N+]([O-])=O)cc1-c(s2)cc(c2C(=O)O)NC(=O)c(c3)ccc(c34)OCO4	-8	10
Z204342356	c1ccc([N+]([O-])=O)cc1-c(s2)cc(c2C(=O)O)NC(=O)c(c3)ccc(c34)OCCO4	-1	7
Z204342388	c1ccc([N+]([O-])=O)cc1-c(s2)cc(c2C(=O)O)NC(=O)c(c3)ccc(c34)CCC4	-1	7
Z207321514	c1cc(Cl)cc(Cl)c1NC(=O)C(C)Sc(n2)nn(c23)cccn3	5	3
Z208192218	c1cccc(c12)[nH]c(n2)SCc3ccc(cc3)-c(n4)nn5c(=O)[nH]c(c6c45)cccc6	16	2
Z215601214	n1oc(C)cc1COC(=O)c2cc(-c3cccs3)nc(c24)n(C(C)C)nc4	-2	9
Z216407296	c1cccc(F)c1NC(=O)c2cc(-c3ccco3)nc(c24)n(C(C)C)nc4	37	15
Z216416610	CC(=O)c1cc(ccc1)NC(=O)c2cc(-c3ccco3)nc(c24)n(C(C)C)nc4	-1	2
Z216417110	c1cc(F)cc(Cl)c1NC(=O)c2cc(-c3ccco3)nc(c24)n(C(C)C)nc4	21	5
Z216541782	c1cc([N+]([O-])=O)cc(c1C)NC(=O)c2cc(-c3ccco3)nc(c24)n(C(C)C)nc4	-4	3

Z216541962	[O-][N+](=O)c1c(F)ccc(c1)NC(=O)c2cc(-c3ccco3)nc(c24)n(C(C)C)nc4	-1	8
Z21689617	Clc1ccc(nc1)NC(=O)C(C)Sc2nnnn2-c(c3C)cc(C)cc3	8	6
Z217430364	CC(C)n(nc1)c(c12)ncc(c2)C(=O)Nc(c3)ccc(c34)[nH]c(=O)[nH]4	-10	6
Z217923492	N#Cc1cc(F)c(cc1)COc(c2C(=O)N)cc(C)cc2	9	7
Z218238558	o1cccc1C(=O)NCc2ccc(o2)-c(n3)sc(c34)cccc4	12	6
Z218834328	COc(c1)cc(OC)cc1NC(=O)COC(=O)c2cc(-c3cccs3)nc(c24)onc4C	6	5
Z219200548	c1ccc(O)c2Cc(c3c12)c(c4c(n3)ccc(Cl)c4)-c5c(Cl)cccc5	-8	2
Z219812624	Cc1cc(ncc1)NC(=O)c2cc(-c3ccc(F)cc3)nc(c24)onc4C	-14	19
Z219813228	n1ccccc1NC(=O)c2ccc(cc2)N(C3=O)CCN3	9	5
Z219953062	O=c1[nH]c(=O)[nH]c(C)c1CCC(=O)N(CC2)CCC2CCc3ccccc3	4	6
Z220381008	c1cccn(c12)cc(c2C#N)CN3CCN(CC3)S(=O)(=O)c4c(Cl)ccc(Cl)c4	-9	2
Z220421452	n1nnn(c12)nc(cc2)NC(C)c(c3)ccc(c34)CCCC4	-3	7
Z221422064	c1cc(Cl)cn(c12)cc(n2)CSc([nH]3)nnc3-c(c4C)cccc4	36	4
Z222976544	O1COc(c12)ccc(c2)NC(=O)c3cc(-c4ccccc4)nc(c35)n(C)nc5C	-2	3
Z223026186	CNC(C)c1ccc(cc1)-n(cn2)c(c23)cccc3	-12	9
Z223042638	c1ccccc1-c2c(F)c(-c3ccccc3)nc(c24)ccc(Cl)c4	-12	5
Z223042658	c1ccccc1-c2c(F)c(-c3ccccc3)nc(c24)cccc4	30	12
Z223263980	CCCCN(C(CC1)CS1(=O)=O)S(=O)(=O)c2c(Cl)cc(Br)cc2	5	10
Z223337292	c1cc(C)c(OC)cc1C(=O)NCCc2ccc(cc2)-n3cccn3	3	7
Z223450274	c1cccc(c12)sc(n2)C(¥C#N)=C3/C=C(C)N(C(C)=C3)c(c4)ccc(c45)OCCO5	-6	6
Z223639244	c1nn(C)c(c12)ncnc2N3CCNCC3	-11	5
Z223690154	c1cccc(c12)sc(n2)C(¥C#N)=C3/C=C(C)N(C(C)=C3)c4ccc(cc4)OCC	20	9
Z223844412	c1ccccc1-c(cc2C(=O)O)nc(c23)n(nc3)Cc4c(Cl)cccc4	7	11
Z223848612	CC(C)NC(=O)c1cc(nc(c12)n(CC)nc2)-c3ccccc3	6	4
Z223859126	c1ccccc1CN(C)C(=O)c2cc(nc(c23)n(CC)nc3)-c4ccccc4	-16	1
Z223881508	CCN(CC)C(=O)c1cc(-c(c2C)cc(o2)C)nc(c13)n(C(C)C)nc3	-12	6
Z223887016	n1ccccc1CNC(=O)c2cc(nc(c23)n(CC)nc3)-c4ccccc4	-12	14
Z223925998	N#Cc1ccc(cc1)NC(=O)c2cc(-c3cccs3)nc(c24)n(CC)nc4	6	7
Z223943920	C1CCCC(Cl)=C1C(=O)Nc(c2)cc(C(=O)OC)cc2C(=O)OC	-1	4
Z224007418	[nH]1c(=O)[nH]c(c12)ccc(c2)NC(=O)c3cc(nc(c34)n(CC)nc4)-c5ccccc5	11	3
Z224289160	O=C(O)/C=C/c1ccc(cc1)NC(=O)CSC(c2ccccc2)c3ccccc3	-8	7
Z224316064	Cc1cnc(s1)NC(=O)c2cc(nc(c23)n(CC)nc3)-c4ccccc4	10	7
Z224328394	c1ccc(OC)cc1CNC(=O)c2cc(nc(c23)n(CC)nc3)-c4ccccc4	6	3
Z224344580	FC(F)(F)c1cc(ccc1)-c(no2)nc2CN3CCN(CC3)S(=O)(=O)c4c(Cl)cccc4	-5	6
Z224588706	CSc(cc1)ccc1CN(C)C(=O)c2cc(nc(c23)n(CC)nc3)-c4ccccc4	-5	3
Z224933090	Cc1c(C)cccc1NC(=O)CN(CC2)CCN2C(=O)c3cc(nc(c34)n(CC)nc4)-c5ccccc5	-4	3

Z224953910	c1ccccc1-c(nc(c23)n(CC)nc2)cc3C(=O)NCc4c(OC)cc(OC)c(c4)OC	2	5
Z225035912	n1ccccc1C(C)NC(=O)c2cc(-c(cc3)ccc3C)nc(c24)n(C(C)C)nc4	16	6
Z225289396	C#CCNC(=O)c1cc(-c2cccs2)nc(c13)n(CC)nc3	-4	2
Z225316878	[O-][N+](=O)c1c(F)ccc(c1)NC(=O)c2cc(-c3cccs3)nc(c24)n(CC)nc4	31	7
Z225376048	CC(C)NS(=O)(=O)c(ccc1)cc1C(=O)Nc(n(c23)cccc2C)c(n3)-c4ccccc4	0	2
Z225443610	CCOC(=O)c([nH]c1C)c(C)c1C(=O)CSc([nH]2)nnc2-c3ccccc3	-8	4
Z225893888	Cc1csc(n1)CNC(=O)c2cc(nc(c23)n(C(C)C)nc3)-c4ccccc4	1	2
Z225896678	s1cc(C)nc1CNC(=O)c2cc(c[nH]2)S(=O)(=O)N(CC)CC	9	6
Z225919834	Cn1ccnc1C(c2c(F)cccc2)NC(=O)c3cc(-c(s4)ccc4C)nc(c35)n(C(C)C)nc5	-2	3
Z226257158	s1cccc1C(=O)Nc(c(C)on2)c2/C=C/c3ccc(cc3)OC	4	8
Z226277016	n1c[nH]c(c12)ccc(c2)C(=O)NCc3ccc(cc3)COCC(F)(F)F	13	12
Z226329302	CC1CN(CC(O1)C)C(C)(C)CNC(=O)c2ccc(cc2)NS(=O)(=O)c3ccccc3	6	13
Z226430254	o1cccc1C(=O)NCCC(=O)Nc(cc2)cc(c23)sc(n3)N4CCCC4	3	8
Z226851604	c1cccc(c12)nc(nc2N)CSc(o3)ncc3-c4ccc(F)cc4	-34	5
Z226924464	COCCOC(=O)c1cc(-c(cc2)ccc2C)nc(c13)n(C(C)C)nc3	6	5
Z227189870	N#CCOC(=O)c1cc(-c2cccs2)nc(c13)n(CC)nc3	2	4
Z227321660	o1nc(C)cc1COC(=O)c2cc(nc(c23)n(C(C)C)nc3)-c(c4)ccc(c45)OCCO5	48	4
Z227429876	CN(C)S(=O)(=O)c(c1)ccc(c12)n(CCC)c(n2)CSc3ncn(n3)-c4ccccc4	-10	6
Z227991512	CCNC(=O)c1cc(-c2ccccc2)nc(c13)n(nc3)Cc4ccccc4	6	6
Z228032620	FC(F)(F)COc(nc1)ccc1C(=O)Nc(n2)n(CCN(CC)CC)c(c23)cccc3	14	10
Z228081748	CC(=O)NCCNC(=O)c1cc(-c2ccccc2)nc(c13)n(nc3)Cc4c(Cl)cccc4	50	9
Z228153102	c1cccc(c12)[nH]c(n2)CCNC(=O)c3cc(-c4ccccc4)nc(c35)n(nc5)Cc6ccco6	7	5
Z228220646	CC(C)n(nc1)c(c12)nc(-c(c3C)cc(o3)C)cc2C(=O)Nc4ccc(nc4)N(CC5)CCN5C	15	4
Z228473210	c1csc(c12)nc([nH]c2=O)SCc3coc(n3)-c4ccccc4	-4	4
Z228517946	NC(=O)COC(=O)c1cc(-c2ccccc2)nc(c13)n(nc3)Cc4ccccc4	4	8
Z228518838	CNC(=O)NC(=O)COC(=O)c1cc(-c2ccccc2)nc(c13)n(nc3)Cc4ccccc4	-8	4
Z228519880	n1oc(C)cc1COC(=O)c2cc(-c3ccccc3)nc(c24)n(nc4)Cc5ccccc5	-10	5
Z228588940	c1cc(S(=O)(=O)Cl)ccc1S(=O)(=O)N2CCCCC2	21	15
Z228595564	N#Cc1cc(ccc1)/C=C¥2C(=O)Nc(c23)cccc3	8	4
Z228607818	Cc(c1)ccc(c12)nc3c(c2C(=O)O)CCCC¥3=C¥c4cnn(C)c4	-3	9
Z228695802	[nH]1c(=O)[nH]c(c12)ccc(c2)NC(=O)c3cc(-c4ccccc4)nc(c35)n(C)nc5C	-6	4
Z228734424	c1cc(C(F)(F)F)ccc1C(=O)N(CC2)CCC2C(=O)Nc3cccnc3	-1	5
Z229078222	CSc(cc1)ccc1CN(C)C(=O)c2cc(-c3ccccc3)nc(c24)n(C)nc4C	9	7
Z229235170	C1CCCN1C(=O)CCNC(=O)c2cc(-c3ccccc3)nc(c24)n(nc4)Cc5ccco5	3	13
Z229243940	c1ccccc1C2(CC2)CNc3ncnc(c34)n(C)nc4	-5	7
Z229381330	N#Cc1ccc(cc1)CN(CC2)CCCN2C(C)C(=O)NC(C)c3ccccc3	-12	8

Z229474284	N#Cc1cc(F)c(cc1)CN(CC2)CCC2C(=O)c(c3)ccc(c34)OCCO4	9	10
Z229553850	CONC(=O)c1cc(-c2ccccc2)nc(c13)n(nc3)Cc4ccco4	-9	12
Z229615400	c1cccc(c1C)-c(cc2C(=O)N)nc(c23)n(C(C)C)nc3	14	8
Z229828974	c1cc(Cl)ccc1-c(n[nH]2)cc2C(=O)Nc3ccc(nc3)N4CCOCC4	8	13
Z230096354	c1ccccc1C(Cc2ncc[nH]2)NC(=O)CSCc3ccc(C)cc3	28	22
Z230132198	c1cccc(c12)C(=O)N(C2=O)CC(=O)NCc3c(-c4ccccc4)nn(c3)Cc5ccccc5	24	12
Z231937310	CCn(c(C)c1)c(C)c1C(=O)N(C2)CCc(c2c34)[nH]c3cccc4	6	6
Z232651876	c1cccc(c12)sc(n2)CSCc3nc(N(C)C)nc(n3)N	-13	4
Z232651902	c1cc(F)ccc1NC(=O)C(C)SCc2nc(N(C)C)nc(n2)N	-3	6
Z234515634	c1ccccc1-n(c2)ncc2C(=O)NCCc3cnn(c3)-c4ccccc4	-7	5
Z234860216	o1cccc1CNC(=O)c2cc(-c3cccnc3)nc(c24)n(nc4)Cc5c(Cl)cccc5	19	4
Z234865770	c1ccccc1Cn(c(C)c(C)c2C#N)c2NC(=O)CN3CCN(CC3)c4ncnc(c45)n(C)nc5	13	5
Z235428434	c1cc(Cl)ccc1OC(C)(C)C(=O)Nc2ncnc(c23)oc(C)c3C	9	7
Z235446856	c1cccc(O)c1NC(=O)c2cc(C3CC3)nc(c24)n(C)nc4C	27	12
Z235467022	c1cccc(O)c1C(=O)c(c2)cc(C(=S)N)c(n3)n2c(c34)cccc4	5	4
Z235827834	o1cccc1Cn(c2=O)c(nc(c23)cccc3)CN(CC4)CCN4Cc5nc(no5)-c(cc6)ccc6C	3	4
Z236551530	c1ccccc1¥C=C¥c2nnc([nH]2)SC(C)c([nH]c3=O)nc(c34)cccc4	-2	1
Z236994250	CC(=O)c1cc([nH]c1)C(=O)NCc2csc(n2)-c3ccccc3	4	5
Z237479802	CN(C)CCNCCOc(c1C)c(Br)cc(Cl)c1	9	24
Z237480338	NS(=O)(=O)c1cc(S(=O)(=O)Cl)ccc1	3	6
Z237523250	CCCCN(S(=O)(=O)C)C(CC1)CS1(=O)=O	-21	4
Z237731508	C1COCCN1CCn(c2=O)c(=O)n(C)c(c23)nc4n3cc(-c5ccccc5)n4-c(cc6)ccc6C	14	4
Z237731644	c1ccccc1¥C=C¥Cn2c(nc(c23)n(C)c(=O)[nH]c3=O)N(CC4)CCN4Cc5c(C)ccc(C)c5	5	4
Z237733696	c1cc(Cl)ccc1Cn2c(nc(c23)n(C)c(=O)[nH]c3=O)N(CC4)CCN4Cc5c(C)ccc(C)c5	8	4
Z237733700	c1ccccc1¥C=C¥CN2CCN(CC2)c(nc(c34)n(C)c(=O)[nH]c3=O)n4Cc5c(C)ccc(C)c5	36	11
Z237744790	NS(=O)(=O)c1cc(ccc1)S(=O)(=O)N2CCCCC2	-8	4
Z237745062	NS(=O)(=O)c1cc(ccc1)S(=O)(=O)N2CCCCCC2	-9	8
Z237751694	CCCN(CCC)S(=O)(=O)c1cc(S(=O)(=O)N)ccc1	-9	13
Z237862822	c1ccccc1C(N(CC)CC)CNC(=O)C2CCN(CC2)c(cc3)nn(c34)c(C(F)(F)F)nn4	0	6
Z238053762	c1ccccc1N(CC)C(=O)c2cc(-c3ccccc3)nc(c24)n(nc4)Cc5cccnc5	-11	7
Z238073806	CC(C)(C)OC(=O)N(CC1)CCN1C(=O)c2cc(-c3ccccc3)nc(c24)n(nc4)Cc5ccncc5	5	26
Z238298390	c1cccc(c12)sc(n2)N(C)C(=O)c(cc3)cc(c34)CC(OC4=O)c5ccccc5	-4	4
Z238616284	C1COCCN1CC(=O)N2CCN(CC2)Cn(c(=S)n3-c4ccc(F)cc4)nc3-c5ccccc5	-5	3
Z238676184	c1ccccc1-c2c(C)sc(c23)nc(-c4ccccn4)nc3N5CCC5	-9	2
Z238683848	CCOC(=O)c(c1C)sc(c12)nc(CN3CCOCC3)nc2Nc4ccc(nc4)N5CCCCC5	37	10
Z238688270	CC1CN(CC(O1)C)CCCNc2ccc([N+]([O-])=O)c(c23)non3	4	16

Z238765362	s1c(Cl)ccc1-c2csc(n2)Nc3ccccn3	28	13
Z238811284	Cc(c1)[nH]c(C)c1C(=O)CN2CCN(CC2)c(cc3)c(F)cc3C(=O)C	1	6
Z238831180	c1ccccc1C(c2ccccc2)NC(=O)C(C)N3CCN(CC3)c4ncnc(c45)n(C)nc5	64	7
Z238883342	c1ccccc1CCCn2c(nc(c23)n(C)c(=O)[nH]c3=O)CN4CCN(CC4)c5cc(Cl)ccc5	-1	3
Z239083338	c1ccccc1-n2nnnc2SCCn3c(nc(c34)n(C)c(=O)[nH]c4=O)N(CC5)CCC5Cc6ccccc6	2	4
Z239092730	c1ccccc1-n2nnnc2SCCn3c(nc(c34)n(C)c(=O)[nH]c4=O)N(CC5)CCN5Cc6ccccc6	47	7
Z239539964	Cc1nn(C)c(c12)ncc(c2)NC(=O)c3cc(-c4ccco4)nc(c35)onc5C	1	4
Z239623050	NC(=O)C1CCCN(C1)c2nc(-c3ccncc3)nc(c24)cccc4	13	7
Z24102979	C1CCCC(C1C)NC(=O)NC(=O)Cn(c2=O)c(=O)n(Cc3ccccc3)c(c24)ncn4CCOC	-2	3
Z24103907	c1ccccc1Cn(c(c23)ncn3CC)c(=O)n(c2=O)CC(=O)N(CC4)CCC4Cc5ccccc5	9	4
Z241121498	C1CCCCN1CCn(c2=O)c(=O)n(C)c(c23)nc4n3cc(-c5ccccc5)n4-c6ccc(cc6)OC	-22	17
Z24149625	COC(=O)c1ccc(cc1)CNC(=O)Cn(c2=O)c(=O)n(Cc3ccccc3)c(c24)ncn4CCC(C)C	8	8
Z24172012	s1ccn(c12)c(=O)cc(n2)CSc(n3CC=C)nnc3-c4ccncc4	4	7
Z241817962	CC(=O)c1ccc(cc1)NC(=O)CCn2c(=S)[nH]nc2-c3cccs3	-1	7
Z24188026	c1cccc(F)c1OCc2nnc(n2CC=C)SCCCn3c(=O)[nH]c(c34)cccc4	-6	6
Z242175442	n1ccccc1CNC(=O)c2cc(-c3cccnc3)nc(c24)n(nc4)Cc5ccco5	22	14
Z24303483	Cc1ccc(cc1)C2=NN(C(C2)c3ccco3)C(=O)CSc(n4C)nnc4-c5cccnc5	44	16
Z24312958	c1cc(C)ccc1C(=O)C(c2ccc(C)cc2)Sc(n3C)nnc3C(O4)COc(c45)cccc5	0	7
Z24395276	FC(F)(F)c1c(Cl)ccc(c1)NC(=O)COC(=O)CNS(=O)(=O)c(cc2)ccc2OC	8	6
Z24398732	c1cccc(c1C(F)(F)F)S(=O)(=O)N(CC=C)CC(=O)Nc2ccc(cc2)S(=O)(=O)N3CCOCC3	0	6
Z244011694	o1cccc1C(N(C)C)CNC(=O)C(=C2)COc(c23)cccc3	3	5
Z244561242	c1cc(C)ccc1Cn(c2=O)c(=O)n(C)c(c23)nc4n3cc(C)n4-c5ccc(cc5)S(=O)(=O)N	9	3
Z244561600	O=C(O)CNc(nc1N)nc(c1[N+]([O-])=O)NCC2CCCO2	9	5
Z245295930	COc(cc1)ccc1Nc(c2[N+]([O-])=O)nc(nc2N)N3CCCCC3	8	9
Z245304076	c1ccccc1C(C(=O)N)N(CC2)CCC2CCc3ccc(O)cc3	-19	8
Z245503032	n1n[nH]c(c12)ccc(c2)C(=O)Nc(n3)n(Cc4ccco4)c(c35)ccc(C(F)(F)F)c5	0	3
Z24578166	c1ccccc1C2=NN(CC2)C(=O)CSc(n3N)nnc3COc(cc4)ccc4C(C)(C)C	40	8
Z246071802	c1ccccc1¥C=C¥CN2CCN(CC2)c(nc(c34)n(C)c(=O)[nH]c3=O)n4Cc5ccc(Cl)cc5	-14	8
Z246801514	c1cccc(c12)ncc(c2)NS(=O)(=O)c(c3)ccc(Cl)c3Cl	3	5
Z247616328	C1CCCCN1CCn(c2=O)c(=O)n(C)c(c23)nc4n3cc(C)n4-c5ccc(cc5)Oc6ccccc6	11	15
Z24778709	c1ccnc([N+]([O-])=O)c1OCC(=O)Nc(c2)ccc(c23)OCCO3	9	10
Z24799056	CC(=O)c1cc(c(cc1)OC)CSc2n[nH]c(=O)n2CCC	28	16
Z24835676	Cc1c(C)ccc(c1)SCc2nc(nc(n2)N)Nc(c3C)cccc3	-13	3
Z24930592	N#Cc1ccc(cc1)NC(=O)CSc(nc(c23)cccc3)n(c2=O)CCc4ccc(F)cc4	17	9
Z25047555	Cc(c1)ccc(c1C)SCc2nc(nc(n2)N)Nc(c3C)cccc3	5	4
Z25066492	CC(=O)Nc1ccc(cc1)SCc2csc(n2)-c3ccc(F)cc3	-1	8

Z25096354	c1cc(O)ccc1-n2nnnc2SCC(=O)Nc(cc3)ccc3C(=O)Nc4ccc(cc4)OCC	24	24
Z25138787	Cc(c1)cc(C)c(c1C)NC(=O)CNC(=O)COc(cccc2)c2C(=O)N3CCCCC3	-1	5
Z25181441	n1nncn1-c2ccc(cc2)OCC(=O)Nc(cc3)ccc3C(=O)Nc4c(OC)cccc4	17	4
Z25191222	c1cc(F)ccc1NC(=O)C(C)Sc([nH]2)nnc2-c3c(Br)cccc3	4	3
Z25344405	c1cccc(c12)nc(cc2)¥C=C¥C(=O)OCCCn3c(=O)[nH]c(c34)cccc4	-5	6
Z25398010	c1cc(C)cn(c12)cc(n2)CSc([nH]3)nnc3-c4ccccc4	-4	6
Z25398193	c1cccc(c12)[nH]cc2C(=O)C(C)Sc([nH]3)nnc3-c4ccccc4	49	15
Z25398215	s1cccc1-c(n2)occ2CSc([nH]3)nnc3-c4ccccc4	-6	15
Z25398505	COC(=O)c1c(C)oc(c1)CSc([nH]2)nnc2-c3ccccc3	1	13
Z25399394	c1cc(Cl)ccc1C2=NN(C(C2)c3ccco3)C(=O)CSc([nH]4)nnc4-c5ccccc5	13	4
Z254387106	[nH]1cc(Cl)cc1C(=O)NCC(c2ccc(o2)C)N3CCOCC3	-10	4
Z254580980	CN(C)C(=O)c1cc(-c2cccs2)nc(c13)n(CC)nc3	-9	6
Z254636716	N1CCN(CC1=O)C(=O)c2nn(c(c23)CCCCC3)-c4ccc(F)cc4	10	6
Z25470704	CC(C)c1nc(n[nH]1)SCc2ncc(o2)-c3ccccc3	-8	8
Z255115986	c1c(Br)ccc(c12)nc(cc2C(=O)O)¥C=C¥c(cc3C(F)(F)F)ccc3	-7	3
Z25645184	O=S1(=O)CC(CC1)N(C)C(=O)COc(cc2)cc(c23)oc(C)c(c3=O)-c4c(F)cccc4Cl	-1	4
Z25766910	s1cccc1CNC(=O)Cn(c(c23)cccc2)c(n3)NCC(C)O	-11	5
Z25824142	CC(=O)CCc1ccc(cc1)OCc(n2)cc(=O)n(c23)cc(C)cc3	-24	9
Z25969255	Cc1c(Cl)cc(OC)c(c1)NC(=O)CN(Cc2ccco2)S(=O)(=O)c3cc(Cl)ccc3	38	15
Z25980301	c1ccccc1CN(S(=O)(=O)c(cc2C(F)(F)F)ccc2)Cc3cc([N+]([O-])=O)cc(c34)COCO4	7	23
Z26082391	CC(=O)Nc1ccc(cc1)S(=O)(=O)N(CC=C)Cc2nnc(o2)-c3ccc(Cl)cc3	3	4
Z26091205	N#Cc1cc(ccc1)NC(=O)CN(CC=C)S(=O)(=O)c(c2F)cccc2	3	11
Z26125608	c1cc(F)ccc1CN(S(=O)(=O)c(c2C)cc(C)cc2)CC(=O)Nc3ccc(cc3)Oc4ccccc4	-6	6
Z26325749	COc(cc1)cc(c1C(=O)N)OCc(n2)c(C)nc(c23)cccc3	35	5
Z26345967	Cc1cc(ccc1)OCC(=O)Nc(c2C(=O)N)sc(c23)CCC3	18	1
Z26347491	C1CCc(c12)sc(c2C(=O)N)NC(=O)COc3ccc(F)cc3	-6	1
Z26400530	c1ccc(C)c(c1C(C)C)NC(=O)c2cc(OC)c(cc2)OCc3c(C)noc3C	1	4
Z26593504	C1COCCN1c(cc2)ccc2NC(=O)CN(C)C(=O)c3c(-c(cc4)ccc4C)nn(c3)-c5ccc(F)cc5	46	14
Z26593505	C1COCCN1c(cc2)ccc2NC(=O)CN(C)C(=O)c3c(-c(cc4C)ccc4)nn(c3)-c5ccccc5	2	2
Z26596638	N1C(=O)C(C)Sc(c12)ccc(c2)C(=O)NCC(N(C)C)c3ccc(cc3)OC	27	12
Z26661789	c1ccc(C)n(c12)cc(n2)C(=O)Nc(n3)scc3-c(c4C)cc(C)n4C	8	19
Z26735071	n1ccccc1N(CC2)CCN2C(=O)c3c(-c4cccnc4)nn(c3)Cc5ccccc5	21	22
Z26735553	c1cc(Cl)ccc1S(=O)(=O)N(Cc2ccccc2)CC(=O)N3CCN(CC3)c4ccccn4	16	3
Z26780414	c1cccc(c12)[nH]c(n2)CNC(=O)c3ccc(cc3)OCc4ccccc4	5	6
Z26782031	c1ccccc1OCC(=O)NCc(n2)[nH]c(c23)cccc3	-12	52
Z26792816	[nH]1c(=O)[nH]c(c12)ccc(c2)NC(=O)c(c3)sc(c34)c5c(CC4)cccc5	4	12

Z26815125	NC(=O)Nc(cc1)ccc1C(=O)NCCC(c2ccccc2)c3ccccc3	-8	7
Z26828279	FC(F)(F)c1cc(ccc1)N(CC2)CCN2C(=O)c3c(-c4cccnc4)nn(c3)Cc5ccccc5	4	5
Z26849648	C1CC1C(=O)N(CC2)CCC2C(=O)N3CCN(CC3)c4cccc(C)c4C	-2	14
Z269649850	C1COCCN1CC(c2ccccc2)NC(=O)c3ccc(cc3)-c(nc4N)nc(c45)sc(C)c5C	15	29
Z26970092	CNS(=O)(=O)c(c(Cl)cc1)cc1C(=O)N2CCN(CC2)Cc3c(OC)ccc(c3)OC	28	5
Z269761360	s1cccc1-c2nnc(n2CCC(=O)N)SCc(n3)cn(c34)cccc4	7	3
Z26987740	c1ccc(F)cc1NC(=O)CN(C)C(=O)c(c2)c(O)cc(c23)cccc3	21	16
Z27019069	C1COCCN1C2(CCCCC2)CNC(=O)c3ccc(cc3)S(=O)(=O)N(C4)CCCC4C	7	1
Z27081729	c1cccc(OC(F)F)c1NC(=O)c(c2)ccc(c23)C(=O)N(C3=O)Cc4ccncc4	20	4
Z271080962	s1cccc1C#Cc2cc(ccc2)NC(=O)C(C3)CC(C(=O)C34)CCC4	-2	5
Z271111810	CC(C)Cn(c1=O)c(=O)n(C)c(c12)nc3n2cc(C)n3-c4ccc(cc4)S(=O)(=O)N	4	4
Z27116942	s1ccn(c12)cc(n2)CC(=O)NCCN(C(=O)S3)C(=O)/C3=C¥c4cccnc4	12	5
Z271700602	c1cnccc1-c2nnc([nH]2)Sc(nc(c34)cccc4)nc3-c5ccccc5	-1	9
Z27234574	c1cccc(c1C#N)S(=O)(=O)N(CC2)CCN2C(=O)/C=C/c(c3)ccc(c34)OCCCO4	36	6
Z27271554	Cc1c(C)cccc1NC(=O)CNC(=O)c(n2)cn(c23)c(C)ccc3	3	3
Z27305591	CC(C)c1c(C)cc(cc1)OCC(=O)NC(C)c2ccc(cc2)-n3ccnc3	-7	4
Z27305643	CCc1cc(ccc1)OCC(=O)NC(C)c2ccc(cc2)-n3ccnc3	28	11
Z27306302	CC(C)Oc(c(Cl)c1)c(OC)cc1C(=O)NC(C)c2ccc(cc2)-n3ccnc3	7	4
Z27306758	o1cccc1C(=O)NC(C(C)C)C(=O)NC(C)c2ccc(cc2)-n3ccnc3	4	0
Z27307020	CCN(CC)c(cc1)ccc1C(=O)NC(C)c2ccc(cc2)-n3ccnc3	9	2
Z27307326	c1cc(C)cc(c1C(C)C)OCC(=O)NC(C)c2ccc(cc2)-n3ccnc3	11	3
Z275104396	c1cccc(F)c1C(=O)N2CCN(CC2)c3nc(CN(CC4)CCC4C)nc(c35)sc(C)c5C	0	13
Z275500628	CN(C)S(=O)(=O)c(c1)ccc(c12)n(CC)c(n2)CSc(o3)nnc3-c(c4C)sc(n4)C	49	6
Z27562098	c1cccc(c1CC)NC(=O)CNC(=O)c(c2)[nH]c(c23)cccc3	48	7
Z27664836	c1cccc(c12)[nH]c(n2)NC(=O)c(c3)ccc(c34)C(=O)N(C4=O)c5ccc(cc5)OCC	-4	11
Z27664887	c1cccc(c12)[nH]c(n2)NC(=O)c3ccc(cc3)N4CCCC4=O	8	7
Z27665121	CC(=O)c1cc(ccc1)OCC(=O)Nc(n2)[nH]c(c23)cccc3	-6	3
Z27666510	c1cccc(c12)[nH]c(n2)NC(=O)COc(c3)ccc(c34)cccc4	8	6
Z276849522	c1ccccc1/C=N/c(cc2)ccc2-c(nc(c34)ccc(Cl)c4)cc3-c5ccccc5	-1	2
Z27763477	c1ccccc1CN(CC2)CCN2C(=O)C3CCCN3C(=O)C45CC6CC(C4)CC(C5)C6	-3	11
Z27764077	c1ccccc1CN(CC2)CCN2C(=O)Cc3c(C)n(c(c34)ccc(c4)OC)C(=O)c5ccc(Cl)cc5	10	7
Z278205390	CCn(c1=O)c(=O)n(C)c(c12)nc3n2cc(C)n3-c(cc4)ccc4N(C)C	10	3
Z27970230	FC(F)(F)c1cc(ccc1)OCC(=O)Nc(n2)sc(c23)cc(F)cc3	-8	4
Z27972425	CN1CCN(CC1)c(c2)ccc(c2C)NC(=O)c(c3)ccc(c34)C(=O)N(C4=O)c(c5C)cc(C)cc5	8	2
Z27972767	CN1CCN(CC1)c(c2)ccc(c2C)NC(=O)c3c(OC)ccc(c3)CN(C4=O)C(=O)c(c45)cccc5	17	8
Z27974061	COc(cc1)c(OC)cc1-c(nc(c23)cccc3)cc2C(=O)Nc(c4C)ccc(c4)N(CC5)CCN5C	3	6

Z279742778	CS(=O)(=O)c1ccc(cc1)S(=O)(=O)N	21	14
Z28027525	C1COCCN1c(cc2)cc(N3CCOCC3)c2NC(=O)c(c4)sc(c45)n(nc5C)Cc6ccc(F)cc6	3	9
Z28028679	O=C1CCC(=O)N1c(cc2)ccc2C(=O)Nc3c(N4CCOCC4)cc(cc3)N5CCOCC5	-4	4
Z28032810	Cc1ccc(cc1)-n(nc2C)c(c23)nc(-c4ccccc4)cc3C(=O)Nc(n5)scc5CN6CCOCC6	-1	5
Z28129480	Cc(c1)cc(C)c(c1C)NC(=O)c(c2=O)cn3C(C)COc(c3c24)c(c(F)c4)N(CC5)CCN5C	9	1
Z281652038	n1ccnc(N)c1C(=O)Nc(s2)ncc2Cc3cc(F)cc(F)c3	-1	5
Z281877508	CC(C)Cn(c1=O)c(=O)n(C)c(c12)nc3n2cc(C)n3-c(cc4)ccc4N(C)C	6	1
Z28296849	Cc(n1)sc(c12)cc(cc2)NC(=O)c(c3)c(=O)oc(c34)cccc4	13	4
Z283831144	n1cscc1CSc(cccc2)c2C(=O)NCc(cc3)ccc3CN(CC)CC	33	7
Z283846026	c1cc(F)cc(F)c1CN(C)C(=O)c2cc(-c3ccccc3)nc(c24)n(nc4)Cc5ccncc5	11	4
Z284160386	O1COc(c12)ccc(c2)CN(CC)C(=O)c(c3)cnc(c34)n(CCC)c(=O)[nH]c4=O	5	5
Z28450151	c1cccc(c12)[nH]c(c2)C(=O)NCCc3cc(OCC)c(cc3)OCC	-5	19
Z28459327	c1cc(F)ccc1CC(=O)Nc2ccc(cc2)S(=O)(=O)N(C)C3CCN(C)CC3	13	6
Z28468306	c1ccc(Cl)c(c12)oc(c2C)C(=O)Nc3ccnn3C4CCCC4	0	2
Z28541828	FC(F)(F)c1cc(ccc1)C(C)NC(=O)CCc([nH]c2=O)nc(c23)cccc3	-5	2
Z28547957	n1c[nH]c(c12)ccc(c2)C(=O)NC(C)c3cc(F)c(F)cc3	-1	4
Z285525778	CC(=O)N(CC1)c(c12)ccc(c2)NC(=O)c3c(cccc3)SCc(n4)cn(c45)cccc5	-11	11
Z285664940	CS(=O)(=O)c1ccc(cc1)S(=O)(=O)N2CCCC2	8	7
Z285664942	c1cccc(c1S(=O)(=O)C)S(=O)(=O)N2CCCC2	-3	3
Z285665066	CC1CCCN(C1)S(=O)(=O)c(cc2)ccc2S(=O)(=O)C	1	4
Z285665176	CC1CC(C)CN(C1)S(=O)(=O)c(c2S(=O)(=O)C)cccc2	55	5
Z285665192	CCCN(CCC)S(=O)(=O)c(cc1)ccc1S(=O)(=O)C	14	7
Z285666434	c1cccc(Cl)c1CN(C)S(=O)(=O)c(c2S(=O)(=O)C)cccc2	-2	12
Z285666740	c1ccc(Cl)cc1CN(C)S(=O)(=O)c(c2S(=O)(=O)C)cccc2	3	4
Z285674740	FC(F)(F)c1ccnc(n1)Nc(c2)ccc(c23)OCO3	21	19
Z285674790	FC(F)(F)c1ccnc(n1)Nc(c2)ccc(c23)OCCO3	-7	9
Z285676094	FC(F)(F)c1ccnc(n1)Nc2ccc(cc2)OC	-2	3
Z285679120	FC(F)(F)c1ccnc(n1)Nc2ccc(cc2)N3CCOCC3	7	4
Z285694006	FC(F)(F)c1ccnc(n1)Nc(ccc2)cc2/C=C/c3ccccn3	12	2
Z285697278	FC(F)(F)c1ccnc(n1)Nc2ccc(cc2)N3CCCC3=O	-12	13
Z285697736	FC(F)(F)c1ccnc(n1)Nc(cc2)cc(c2OC)S(=O)(=O)N3CCCCC3	22	8
Z285702074	FC(F)(F)c1ccnc(n1)Nc(c2)ccc(c23)[nH]nc3	0	3
Z285712986	FC(F)(F)c1ccnc(n1)Nc(c2)ccc(O3)c2OC34CCCCC4	4	8
Z285929216	c1cccc(c12)nc3c(c2C(=O)O)CN(C)CC¥3=C¥c4ccc(cc4)-n5cncn5	1	12
Z285950112	CCCN(CCC)S(=O)(=O)c1cc(ccc1)S(=O)(=O)N(C)C	-4	2
Z285953016	CN(C)S(=O)(=O)c1cc(ccc1)S(=O)(=O)N(C)C	13	1

Z285953204	CN(C)S(=O)(=O)c1cc(ccc1)S(=O)(=O)NC	2	5
Z286115224	CC(C)c1cc(ccc1)NS(=O)(=O)c(cc2)ccc2-n(c3=O)ncc(Cl)c3Cl	6	3
Z286135428	c1cccc(Cl)c1NC(=O)CN(C)CN(C(=O)N2)C(=O)C23C(C)CCCC3C	5	1
Z28653555	c1ccnc(Cl)c1C(=O)Nc2nnc(s2)SCc3ccc(F)cc3	3	8
Z28653653	s1cccc1C(=O)Nc2nnc(s2)SCc3ccc(F)cc3	10	7
Z287022862	o1cccc1-c(n2)scc2CC(=O)Nc(cc3)cc(c34)sc(n4)N5CCOCC5	10	12
Z28714833	N#Cc1ccc(cc1)C(=O)Nc(n2)scc2-c3cc(F)c(F)cc3	16	6
Z28877665	c1cc(C)ccc1C(CC)NC(=O)c(c(Cl)c2)cc(c23)S(=O)(=O)N=C4N3CCCCC4	2	6
Z28905033	c1cc(F)ccc1CC(=O)N2CCN(CC2)S(=O)(=O)Cc3ccccc3	-2	6
Z290228874	Cn1cccc1C(=O)CN(C(=O)C2=O)C(=O)N2C(C)c3ccccc3	-8	5
Z290622280	CC(C)c1ccc(cc1)SCc2nc(N(C)C)nc(n2)N	4	5
Z29076459	N1C(=O)C(C)Sc(c12)ccc(c2)C(=O)Nc(n3)n(CCC)c(c34)cccc4	51	3
Z29082077	c1csc(c1C#N)NC(=O)CCCSc(n2)[nH]c(c23)cccc3	16	8
Z291423432	C1CCCN1C(=O)CNC(=O)COC(=O)c2c(C)c(C)c(C)c(C)c2C	-6	5
Z291973276	CN(C)S(=O)(=O)c(ccc1)cc1CS(=O)(=O)c2ccccc2	46	7
Z29249346	CCN(CC)CCn(c(c12)cccc1)c(n2)NC(=O)c(c3)ccc(c34)C(=O)N(C4=O)CC5CCCO5	-8	8
Z29249972	CCN(CC)CCn(c(c12)cccc1)c(n2)NC(=O)c(c3)sc(c34)n(nc4C)-c5ccc(Cl)cc5	-12	15
Z29267150	CN(C)S(=O)(=O)c(cc1)ccc1NC(=O)c2ccc(cc2)S(=O)(=O)N(CC)c3ccccc3	2	5
Z29317409	c1nc(O)ccc1C(=O)N(C)C(c2ccccc2)c3ccccc3	-3	6
Z29324663	Cc1cnc(s1)NC(=O)CCCc([nH]c2=O)nc(c23)cccc3	9	7
Z29467100	CC(C)Cn(nc1C)c(c12)sc(c2)C(=O)Nc(n3)scc3-c4ccccn4	0	2
Z29467784	CC(C)(C)C1CCC(CC1)C(=O)Nc(n2)scc2-c3ccccn3	7	4
Z29531247	COc1cccc(c12)cc(c(=O)o2)C(=O)N3CCN(CC3)S(=O)(=O)c(c4C(F)(F)F)cccc4	18	3
Z295403358	O=C(O)C(C)S(=O)(=O)CCC	10	10
Z295403360	O=C(O)C(C)S(=O)(=O)C(C)C	4	5
Z295455358	O=C(O)CCn(nc1C)c(c12)nc(-c3cccs3)cc2C(=O)O	-2	10
Z295463532	COc(cc1)ccc1-n(c(c23)ncnc3O)cc2-c4ccccc4	-5	4
Z295508576	c1cc(F)cc(F)c1C(C)NC(=O)C2=NN(C(C2)C(=O)N)c3ccc(F)cc3	6	8
Z295883036	CCOC(=O)c1cc(-c2ccccc2)nc3[nH]c(=O)c(c4c13)cccc4	9	3
Z296244664	o1cccc1-c(n2)scc2C(=O)Nc(n3)scc3-c4ccc[nH]4	-6	7
Z29631119	C1C(C2)CC(C3)CC2(Cl)CC13C(=O)Oc(cc4)ccc4N(C)S(=O)(=O)c(c5F)cccc5	5	8
Z29631809	C1C(C2)CC(C3)CC2(NC(=O)C)CC13C(=O)Oc(cc4)ccc4N(C)S(=O)(=O)c(c5C)cc(C)cc5	1	7
Z296452432	o1cccc1-c(n2)scc2C(=O)Nc(n3)sc(c34)CN(CC4)C(=O)OC(C)(C)C	-4	9
Z29699890	c1ccccc1C2=CCN(CC2)C(=O)/C=C/c3c(-c4cccnc4)nn(c3)-c5ccccc5	10	8
Z297331992	CC(=O)Nc(c1)ccc(F)c1NC(=O)c2cncn2-c3ccccc3	35	11
Z297680094	o1cccc1-c(no2)nc2CNc(cc3)ccc3-c4ccccc4	1	9

Z298272042	CC(C)(C)c1nc(on1)C(C)Sc([nH]2)nnc2/C=C/c3ccccc3	12	8
Z29827650	c1c(F)ccc(c1C)NC(=O)C(C)Oc2cc(Cl)ccc2	11	5
Z298494086	O=S1(=O)N=C(C)C(=C(C)N1)CCC(=O)Nc(cc2)cc(Cl)c2N3CCCC3=O	0	5
Z29859952	FC(F)(F)c1cc(ccc1)CNC(=O)c2n[nH]c(=O)c(c23)cccc3	6	4
Z299002446	FC(F)(F)c1cc(ccc1)-c(o2)ccc2C(=O)Nc3ccc(nc3)-n4cccn4	56	11
Z29903633	Cc1cc(no1)NC(=O)C(C)SCC(=O)Nc2cccc(c23)nccc3	-3	6
Z29956667	c1ccccc1CCC(=O)Nc(c2C(=O)OCC)cc(s2)-c3cc([N+]([O-])=O)ccc3	-1	2
Z299680982	CS(=O)(=O)c1cc(S(=O)(=O)N)ccc1	5	7
Z300098278	CCC1CCCCN1S(=O)(=O)c(cc2)ccc2S(=O)(=O)C	51	26
Z300114426	CN(C)S(=O)(=O)c(cc1)ccc1S(=O)(=O)C	-8	4
Z300417272	Brc1cc(Cl)cc(c1OC)S(=O)(=O)N(C)c2cccc(n2)OC	4	16
Z300467774	Cc1ccc(cc1)S(=O)(=O)N2CCCN(CC2)S(=O)(=O)N(C3)CC(C)CC3C	4	2
Z30046778	c1nc(O)ccc1C(=O)Nc2c(cccc2)Oc(cc3C)ccc3	8	3
Z300508876	CS(=O)(=O)c1cc(ccc1)S(=O)(=O)N(C)C2CCCCCC2	-7	6
Z30051508	n1[nH]c(=O)ccc1C(=O)Nc2ccc(cc2)Sc(c3C)cc(C)cc3	44	4
Z300664804	c1c(F)ccc(c12)[nH]c(c2)C(=O)NCC3CN(CCO3)CC(C)C	-5	2
Z300797868	C1COCCN1CCCNc(s2)nn(c23)cc(n3)-c4ccccc4	11	7
Z301214762	c1c(F)ccc(c12)[nH]c(c2)C(=O)NCCCn(n3)c(C)cc3C	8	12
Z30166588	n1ccccc1CNC(=O)c(c2)sc(c23)n(nc3C)-c4ccccc4	1	7
Z30216742	c1cc(F)ccc1C(=O)N(CC2)CCC2C(=O)NCCCn3ccnc3	41	4
Z30220173	CCN(CC)S(=O)(=O)c(ccc1)cc1NC(=O)c2cc(OC)c(c(Br)c2)OCCC	20	15
Z30270226	c1cccc(c12)[nH]c(n2)SCCCC(=O)Nc(cc3)ccc3-c(n4)[nH]c(c45)cccc5	17	4
Z30463043	n1c[nH]c(c12)ccc(c2)C(=O)NCCSc3ccc(Br)cc3	7	5
Z30576843	FC(F)(F)c1cc(ccc1)NC(=O)c2cc(ccc2)NC(=O)c3cnccn3	14	8
Z30610492	n1ccccc1COC(=O)c2cc(-c3cc(OC)c(cc3)OC)nc(c24)cccc4	-9	7
Z30640594	NC(=O)NCc(cc1)ccc1C(=O)N2CCN(CC2)c(nc3)c(Cl)cc3C(F)(F)F	4	9
Z30888972	Cc1cc(C)cc(c1)NC(=O)c(c2)ccc(c23)C(=O)N(C3=O)CC=C	20	8
Z30903878	CC(=O)c1ccc(cc1)-c(o2)ccc2C(=O)Nc(c3C)cc(F)cc3	-3	5
Z30983122	c1ccc(Cl)cc1N(CC2)CCN2CC(=O)Nc3sc(c(C)c3C(=O)OCC)Cc(c4)ccc(c45)OCO5	12	4
Z31058092	n1ccccc1-c(nc2NS(=O)(=O)C)nc(c23)scc3-c4ccccc4	18	14
Z31089732	CC(C)(C)c1ccc(cc1)C(=O)Nc2ccc(cc2)Oc3nc(nc(c34)sc(C)c4C)CN5CCOCC5	-13	10
Z31123519	c1ccnc(c1C#N)NCC(c2ccc(cc2)N(C)C)c3c[nH]c(c34)cccc4	0	2
Z31132453	NC(=O)C1CCCN1c2nc(nc(c23)sc(C)c3C)CSc4ccccc4	9	2
Z31132454	NC(=O)C1CCCN1c2nc(nc(c23)sc(C)c3C)CSc4ccc(Cl)cc4	19	5
Z31148700	C1COCCN1c(cc2)ccc2NC(=O)CN(C)c3nc(CN4CCCC4)nc(c35)sc(C)c5C	45	15
Z31152357	CCCN1CCN(CC1)c(c2[N+]([O-])=O)ccc(c2)S(=O)(=O)NC(CC3)CCC3C	-14	9

Z31158506	c1c(Cl)ccc(OC)c1NC(=O)CN(C)c2nc(CN3CCOCC3)nc4sc(c5c24)CC(C)CC5	7	7
Z31169752	c1cccc(c1C#N)S(=O)(=O)N(CC2)CCN2c(nc3)ccc3S(=O)(=O)N(CC)CC	6	5
Z31179156	CN(C)C(=O)c1ccc(cc1)Nc2ccnc(c23)cc(Cl)cc3	32	16
Z31191207	Cc1c(C)sc(c12)ncnc2Nc(c3)ccc(c34)OCO4	14	26
Z31191224	C1CCc2sc(c3c12)ncnc3Nc(c4)ccc(c45)OCO5	15	6
Z31196583	c1ccccc1CN(C)c2nc(CN3CCOCC3)nc(c2c45)sc4CCC5	8	6
Z31211547	c1ccccc1-c2c(C)sc(c23)nc(-c4cccnc4)nc3N5CCOCC5	13	5
Z31232965	Cc1c(C)sc(c12)nc(-c3cccnc3)nc2NC4CC4	-3	3
Z31238841	n1ccccc1-c(nc2N(CC)CCO)nc(c23)scc3-c4ccccc4	19	3
Z31262492	C1CC(C)Cc(c1c23)sc2nc(-c4cccnc4)nc3N5CCCCCCC5	4	27
Z31275874	CCOC(=O)c1ccc(cc1)N(C(=O)c(c23)cccc3)C(=O)C¥2=C¥NCc4ccccn4	-12	1
Z31276645	C1COCCN1CCCN/C=C/2C(=O)N(C(=O)c(c23)cccc3)c4ccc(cc4)OCC	-6	6
Z314617634	c1cc(F)ccc1-n(c(c23)ncnc3O)cc2-c4ccccc4	7	9
Z314931660	CNC(=O)c1ccc(cc1)/C=C¥2CCCc(c3C(=O)O)c2nc(c34)ccc(C)c4	4	5
Z31596603	c1ccccc1C(=O)c2ccc(cc2)OC(C)C(=O)NNC(=O)c3n[nH]c(=O)c(c34)cccc4	14	7
Z316118630	O=C(O)CNS(=O)(=O)C(CC1)CS1(=O)=O	0	8
Z316156320	c1ccc(OC)cc1C(=O)C2=C(O)C(=O)N(C2c3cc([N+]([O-])=O)ccc3)CCCn4ccnc4	-1	5
Z316169876	c1cc(F)ccc1C(=O)C2=C(O)C(=O)N(CCCn3ccnc3)C2c4cc(OC)c(cc4)OCCC	10	3
Z316169960	COc(cc1)ccc1C(=O)C2=C(O)C(=O)N(CCCn3ccnc3)C2c4ccc(cc4)OC(C)C	12	4
Z316264326	c1ncccc1CSc(nn2)n(-c3c(F)cccc3)c2-c4c(Cl)cccc4	1	3
Z316438002	c1ccc(Cl)cc1C2(CC2)C(=O)Nc3cc(ccc3)S(=O)(=O)/N=C4¥CCCCCN4	23	25
Z317042750	c1ccc(F)cc1C(C2)N(C(=O)C3CCCCC3)N=C2c4cc(ccc4)NS(=O)(=O)C	-15	19
Z317186982	C1CCc(c12)ccc(c2)NC(=O)CSCc3cc(no3)-c4ccc(cc4)OC	-4	6
Z317187130	COc(cc1)ccc1-c(no2)cc2CSCc3nc(nc(n3)N)Nc4ccccc4	-7	4
Z317369924	Nc(n1)nc(N(C)C)nc1C(C)Sc2ncn(n2)-c3ccccc3	-4	3
Z317663344	o1cccc1-c([nH]n2)cc2C(=O)Nc(n3)sc(c34)CN(CC4)Cc5ccccc5	-10	6
Z317736476	o1cccc1-c([nH]n2)cc2C(=O)NCc3ccc(nc3)-n4cccn4	-2	3
Z31827545	CCN(CC)S(=O)(=O)c(cc1)ccc1NC(=O)CNC(=O)C23CC4CC(C2)CC(C3)C4	8	12
Z31879630	c1cccc(c12)n(c(n2)C(F)(F)F)CC(=O)NNC(=O)CNC(=O)c(c3)ccc(c34)cccc4	-10	15
Z318799754	FC(F)(F)c1ccnc(n1)Nc(cc2)cc(c2C)S(=O)(=O)N(C)C	6	4
Z318833320	N#CCOc(cc1)ccc1Nc(n2)nccc2C(F)(F)F	7	7
Z318854232	FC(F)(F)c1ccnc(n1)Nc(cc2Cl)cc(Cl)c2N	7	5
Z318995726	FC(F)(F)c1ccnc(n1)Nc(cc2)cc(c23)sc(n3)N4CCCC4	22	7
Z318998372	FC(F)(F)c1ccnc(n1)Nc2ccc(nc2)N3CCOCC3	-7	15
Z319012342	FC(F)(F)c1ccnc(n1)Nc2ccc(nc2)N3CCCC3	-5	7
Z319012738	FC(F)(F)c1ccnc(n1)Nc2ccc(N(C)C)nc2	2	4

Z319025642	C1CCCN1C(=O)c2ccc(cc2)Nc(n3)nccc3C(F)(F)F	-36	16
Z319076580	N#Cc1ccc(nc1)N(CC2)CCN2C(=O)c3ccc(C(C)C)cc3	-2	6
Z319092016	c1nn(C)c(c12)ncnc2N(CCO3)CC3c4ccc(F)cc4	-2	5
Z31930719	c1ccc(F)cc1CC(=O)NNC(=O)Cn(c(c23)cccc2)c(n3)COc4ccc(Cl)cc4	-2	9
Z319403812	c1cnccc1C(=O)/C=c(s2)/[nH]c(=O)¥c2=C¥c3ccc(Cl)cc3	8	4
Z319895028	NC(=O)c1ccc(cc1)CN(C2CC2)S(=O)(=O)c3cc(Cl)ccc3	-5	11
Z320065982	c1ccccc1C(C(=O)N)NCCCN(CC2)c(c23)cccc3	-17	12
Z320075170	CC(=O)N(CC1)c(c12)ccc(c2)NC(C3=O)CC(=O)N3C4CC4	-3	3
Z32207785	c1ccccc1CC(=O)NCC(=O)NC(C)c2ccc(cc2)-n3ccnc3	8	1
Z32220177	C1COCCN1C(C)(C)CNC(=O)c2cc(ccc2)N(CC3=O)C(N)=C3c(n4)sc(c45)cccc5	8	3
Z32361141	c1ccc(Cl)cc1CNC(=O)CCC(=O)N(CC2)N=C2c3ccccc3	30	3
Z32407809	[O-][N+](=O)c1c(F)ccc(c1)NC(=O)CCc2c[nH]c(c23)cccc3	4	10
Z32439393	Cc(c1)sc(C)c1C(=O)CCC(=O)NCCc2c[nH]c(c23)cccc3	21	31
Z325218452	c1cc(F)ccc1NC(=O)c2nnc(s2)CSc(n3)[nH]cc3-c4ccccc4	0	3
Z327260972	c1ccccc1NC(=O)CCSCc2nc(N(C)C)nc(n2)N	-8	4
Z32729001	o1cccc1CNS(=O)(=O)c(cc2)ccc2C(=O)Nc(cc3)cc(c3N4CCCCC4)S(=O)(=O)Nc5ccc(Cl)cc5	-5	9
Z32804094	OCc1cc(ccc1)NC(=O)c2ccc(cc2)NS(=O)(=O)c(c3C)ccc(F)c3	11	4
Z32811942	c1cccc(c12)n(cc2)CCCNC(=O)c3ccc(cc3)OCc4c(C)noc4C	3	3
Z328656306	c1nn(C)c(c12)ncnc2N(CCC)CC(O3)COc(c34)cccc4	21	9
Z328693166	FC(F)(F)CNC(=O)c1ccc(cc1)CN(C)c2cc(Cl)nc(n2)N	10	6
Z328694162	c1cccc(c12)oc(c2)C(C)N(C)c3ncnc(c34)n(C)nc4	-5	5
Z328694806	c1cccc(F)c1CN(C2CC2)c3ncnc(c34)n(C)nc4	-21	7
Z328694928	c1cc(F)c(F)cc1C(C)N(C)c2ncnc(c23)n(C)nc3	-1	4
Z328815354	N#CCCn(nc1C)c(c12)nc(cc2C(=O)OC)-c3ccc(cc3)OC	24	3
Z33270206	Cc(n1)sc(c12)cc(cc2)NC(=O)CNC(=O)c3c(F)cccc3	4	4
Z332932578	c1ccccc1-n(nc2)c(CC)c2C(=O)Nc([nH]3)nnc3-c(cc4)ccc4C	1	3
Z332936670	COc(cc1)ccc1-c2nnc([nH]2)NC(=O)c3ccc(cc3)NC(=O)c4ccccc4	14	4
Z333170264	c1cccc(c1C(F)(F)F)C(C2)C2C(=O)NCc(n3)cn(c34)ccs4	-2	6
Z335452550	COc(cc1)c(OC)cc1C(=O)C2=C(O)C(=O)N(C2c3cccnc3)CCCn4ccnc4	-9	6
Z335491150	c1cnccc1C(C)NC(c2ccccn2)c3ccccc3	3	3
Z335842274	O1COc(c12)ccc(c2)C(=O)CSc(nc3)ccc3S(=O)(=O)N(C)C	-12	7
Z335869204	O1COc(c12)ccc(c2)C(=O)CSc(nc3)ccc3S(=O)(=O)N4CCCC4	12	22
Z335913836	OCCOc(c(Cl)c1)cc(c12)oc(=O)cc2-c3ccccc3	15	6
Z335958518	C1CCc(c12)ccc(c2)C(=O)CSc([nH]3)nnc3C(C)(C)C	-7	8
Z336032014	Cn1nccc1/C=C¥2C(=O)Nc(c23)cccc3	2	3

Z337770612	C1COCCN1c(cccc2)c2NC(=O)c3cc(C4CC4)nc(c35)n(CCC)c(=O)[nH]c5=O	-5	3
Z338592020	O=c1[nH]c(=O)n(CC(C)C)c(c12)nc(C3CC3)cc2C(=O)NCc4ccc(cc4)CN5CCOCC5	6	11
Z339024634	c1nccn1CCCNc2c([N+]([O-])=O)c(=O)n(c(c23)cccc3)-c4ccccc4	-22	5
Z339292340	c1cccc(c12)n(cn2)NC(=O)c3c(-n4cccc4)n(nc3)-c5c(F)cccc5	11	6
Z339583070	c1cccc(c12)nc(cc2)NC(=O)c3ccc(cc3)OCc4cccnc4	-12	2
Z339586288	CC(C)(C)NC(=O)CN(CC1)CCN1C(=O)c2c(CC)n(nc2)-c3cc(Cl)ccc3	0	20
Z352490104	c1cccc(c1C#N)CSCCNC(=O)N2CCN(CC2)c(nc3)c(Cl)cc3C(F)(F)F	-2	9
Z353969056	O=C(O)CCCN(C)S(=O)(=O)c1c(Cl)ccc(c1)S(=O)(=O)C	44	5
Z353972988	CS(=O)(=O)c(c1)ccc(Cl)c1S(=O)(=O)N	2	3
Z355127288	CCC1CCCCN1S(=O)(=O)c2c(Cl)ccc(c2)S(=O)(=O)C	7	5
Z355129038	CC1CC(C)CN(C1)S(=O)(=O)c2c(Cl)ccc(c2)S(=O)(=O)C	12	8
Z355135118	C=C(C)CN(CC)S(=O)(=O)c1c(Cl)ccc(c1)S(=O)(=O)C	-31	11
Z355174726	C1CCCC1N(C)S(=O)(=O)c2c(Cl)ccc(c2)S(=O)(=O)C	-31	5
Z355654248	CC(C)(C)c1nc(on1)COC(=O)c2cc([nH]n2)-c(s3)ccc3C	12	6
Z355820588	Cc1nn(C)c(c12)nc(C)c(c2)C(=O)NCCNc3c([N+]([O-])=O)cccc3	-9	5
Z357095098	Cc1cc(no1)NC(=O)C(C)SCC(=O)Nc2ccc(cc2)SC3CCCC3	-13	7
Z357945110	C1CCCCC1N(C)S(=O)(=O)N(CC2)CCN2c(c3Cl)cn[nH]c3=O	-10	4
Z358275910	c1cccc(F)c1C(=O)N2CCN(CC2)Cn(c(=S)n3CC=C)nc3-c4ccc(Cl)cc4	5	5
Z359311942	CN1CCCN(CC1)c2nc(C(F)(F)F)nc(c23)n(nc3)Cc4ccccc4	-23	5
Z359324360	CCN(CC)C(=O)CN1CCCN(CC1)c2ncnc(c23)n(C)nc3	-4	3
Z359326586	c1ccccc1Cn(nc2)c(c23)nc(C(F)(F)F)nc3Nc4ccc(cc4)S(=O)(=O)NC(C)C	-21	11
Z359326590	c1ccccc1Cn(nc2)c(c23)nc(C(F)(F)F)nc3Nc4ccc(cc4)S(=O)(=O)NC	4	26
Z362662212	c1cccc(OC2)c1c(c23)sc(c3)C(=O)Nc(ccc4)cc4Cn5ccnc5	6	8
Z362662788	c1cc(Br)ccc1OCC(=O)Nc(ccc2)cc2Cn3ccnc3	8	3
Z363094390	c1cccc(F)c1NC(=O)c2c(C3CC3)n(nc2)-c(cc4)cc(Cl)c4C	-5	8
Z363639532	c1ccccc1-c(cc2)ccc2C(=O)NCc3ccc(nc3)-n4ccnc4	3	4
Z364474850	o1c(Br)ccc1¥C=C¥C(=O)Nc2ccc(cc2)S(=O)(=O)C3CCCC3	0	3
Z365246124	C1CCc(c12)ccc(c2)NC(=O)c3cc(C)c(cc3)-n4cnnn4	9	7
Z368055116	C1CCc(c12)sc(c2)C(=O)Nc(c3C(=O)N(C)C)cc(OC)c(c3)OC	-7	6
Z368093014	c1cccc(c12)oc(c2)C(=O)N3CCN(CC3)C(=O)c4ccc(O)nc4	48	9
Z368298320	NC(=O)c1c(F)ccc(c1)NC(=O)c2cc([nH]n2)-c3c(Cl)cccc3	-18	19
Z369173876	FC(F)S(=O)(=O)c1ccc(cc1)S(=O)(=O)N(C)C	3	8
Z369246436	CCC(CC)N(C)S(=O)(=O)c1ccc(cc1)S(=O)(=O)N	-4	14
Z369933958	c1ccccc1CNC(=O)Nc(c2)ccc(c23)oc(n3)C4CC4	11	17
Z370333260	c1cccc(c12)sc(n2)NC(=O)c3cc([nH]n3)-c4ccc(F)cc4	-6	4
Z370715150	CC(=O)N1CCN(CC1)c(nc(n2)C(F)(F)F)c(c23)scc3	6	8

Z370715174	N1CCN(CC1=O)c(nc(n2)C(F)(F)F)c(c23)scc3	26	23
Z370787214	c1ccnc(c12)cccc2NC(=O)c3ccc(=O)n(n3)-c4ccc(F)cc4	43	9
Z373317194	c1occc1C(=O)N2CCN(CC2)c(ncn3)c(c34)sc(c4)-c5ccc(F)cc5	48	11
Z381174904	N#Cc1ccc(cc1)NC(=O)c2ccn(n2)-c3ccc(F)cc3	44	8
Z381180216	Cc1nnc(s1)NC(=O)C(C2)ON=C2c3cc(F)ccc3	-5	5
Z381381388	Nc(s1)nc(c1C)-c(s2)ccc2C	-1	7
Z381428874	c1cccc(OC)c1C(=O)N/N=C¥2C(=O)Nc(c23)ccc(C)c3	15	2
Z381659312	c1cccc(O)c1C(=O)N2CCN(CC2)c3nc(CN(CC4)CCC4C)nc(c35)sc(C)c5C	-12	2
Z383135670	NC(=O)Nc(cc1)ccc1NC(=O)Cc2csc(n2)Cc3ccccc3	-14	3
Z383523130	c1nccn1Cc2cc(ccc2)NC(=O)Cc3csc(n3)Cc4ccccc4	46	5
Z383786992	n1[nH]c(=O)ccc1C(=O)Nc(ccc2)cc2COc3cc(F)ccc3	-2	2
Z384684808	c1ncccc1C(=O)NCC(O)c2ccc(C)cc2	4	13
Z385450636	Cc1c(S(=O)(=O)N)cc(cc1)S(=O)(=O)C	26	10
Z388276110	CC(=O)Nc1ccc(cc1)SCc2nc(on2)-c(cc3C)ccc3	12	14
Z395169542	Cc(n1)oc(c12)ccc(c2)NC(=O)c3c(C)n(c(C)c3)-c4cccnc4	6	2
Z395825976	Cc1noc(c12)ncc(c2)C(=O)NCc3cc(ccc3)Cn4ccnc4	-16	6
Z396609184	CC(=O)N1CCCC1C(=O)NCc2cc(OC)c(cc2)OCc3cccnc3	7	12
Z397745198	FC(F)(F)c1ccnc(n1)Nc2cn[nH]c2	2	5
Z397897560	Cc(n1)cn(c12)nc(s2)NCc3ccc(cc3)N(CC4=O)CCN4	39	11
Z401874244	c1occc1C(=O)N(CC2)CCC2NCc3c(n[nH]c3)-c4ccc(F)cc4	36	10
Z407006622	c1nccn1-c(nc2)ccc2NC(=O)CS(=O)(=O)CCCc3ccccc3	-20	8
Z408469636	CC(C)c(s1)nc(C)c1C(=O)N2CCN(CC2)C(=O)OC(C)(C)C	2	9
Z409207538	CCC(C)N(C)S(=O)(=O)c1cc(S(=O)(=O)N)ccc1	-5	8
Z409420032	C1NCCc(c12)n(nc2)-c3ccccc3	-11	3
Z409956934	CNC(=O)c1ccc(cc1)CN(C)C(=O)Nc(c(c2)OC)cc3oc(c4c23)cccc4	15	5
Z411168810	c1c(Cl)ccc(F)c1NC(=O)NCc(nn2)n(c23)CCC3	6	7
Z412774176	o1cccc1C(=O)Nc2ccc(cc2)NC(=O)NC(C)CCc3ccc(O)cc3	-12	7
Z412775872	o1cccc1C(=O)Nc2ccc(cc2)NC(=O)NCc(cc3C(F)(F)F)ccc3	-5	4
Z413336660	CN(C)C(=O)c1cc(c(Cl)cc1)NC(=O)N(CC2)Cc(c23)cccc3	5	9
Z415024646	COC(=O)c1c(C(=O)OC)cn(C)c1-c2ccc(cc2)NC(=O)c3ccc(cc3)CN4CCCCC4	-1	3
Z415722772	c1c(Br)ccc(c12)nc(-c(cc3)ccc3C#N)cc2-c4ccccc4	-4	5
Z415722774	c1cccc(c12)nc(-c(cc3)ccc3C#N)cc2-c4ccc(Cl)cc4	6	11
Z416890194	CCS(=O)(=O)Cc1cc(ccc1)S(=O)(=O)N(C)C	-10	1
Z420283836	c1cccc(c12)OC(C2)CNC(=O)Cc3csc(n3)NC(=O)c4c(F)cccc4	-5	6
Z421731088	CC(C)(C)C(=O)N(CC1)CCC1NC(=O)c(c2)sc(c23)n(C)nc3C	3	4
Z421986674	N1CCN(CC1=O)c2c(Cl)cc(cc2)NC(=O)c3cc(C)nc(c34)cccc4	7	4

Z424305696	c1cc(F)ccc1-c(n[nH]c2)c2C(=O)NCc3cc(ncc3)Oc4ccccc4	-15	8
Z424307182	c1ncccc1COc(cc2)ccc2C(=O)NCc3cc(ncc3)Oc4ccccc4	-22	3
Z424802904	Cc1[nH]nc(C)c1¥C=C¥C(=O)c2ccc(cc2)N3CCCC3=O	6	2
Z425227654	Cc1cc(O)c(cc1)NC(=O)c2ccc(cc2)N(CC3=O)C(N)=C3c(n4)sc(c45)cccc5	1	13
Z425236088	C1CCCCc(c12)sc(n2)NC(=O)c3ccc(cc3)NC(=O)c4ccccn4	15	7
Z431511574	C1CC1C2=NN(C(=O)C)C(C2)c(c3)c(Cl)nc(c34)cc(OC)c(c4)OC	-6	18
Z431623528	c1cccc(F)c1-c(o2)ccc2CCC(=O)Nc3cc(ccc3)NC(=O)CCn4ccnc4	5	6
Z431773138	CC(C)CCN1CCN(CC1)Cn(n2)c(=S)n(c23)c4c(cccc4)c(=O)n3-c(c5C)cccc5	44	6
Z431947052	c1ccccc1-c2c(CC)nn(c23)c(cc(n3)C)Nc4ccc(F)cc4	52	7
Z431948992	c1ccccc1-c2c(CC)nn(c23)c(cc(n3)C)Nc4ccc(Cl)cc4	6	2
Z431973470	c1cc(Cl)ccc1-c2c(C)nn(c23)c(cc(n3)C(C)(C)C)N(CC4)CCN4CCOc5cc(OC)ccc5	12	5
Z432047124	O=S1(=O)CCN(S(=O)(=O)Cl)CC1	0	6
Z432102126	c1cc(F)ccc1-c2c(C(F)(F)F)nn(c23)c(cc(n3)C)N(CC4)CCN4CCOc5cc(OC)ccc5	-1	4
Z432185696	FC(F)(F)c1cc(c(=O)[nH]c1)NC(=O)CCc2ccsc2	4	3
Z432326346	CN1CCN(CC1)c2ccc(nc2)NC(=O)CSc(nn3)n(c34)c5c(cccc5)c(=O)n4CC=C	8	4
Z432528000	CN(C)S(=O)(=O)c(ccc1)cc1CS(=O)(=O)c2ccccn2	4	6
Z433969260	c1c(F)ccc(F)c1C(=O)C2CCN(CC2)C(=O)Nc3ccc(nc3)OC	-3	5
Z434613616	c1ccccc1C(=O)Nc(c2)ccc(c2C(F)(F)F)NC(=O)NCc3ccc(cc3)Cn4cccn4	-1	5
Z435639920	N1CCN(CC1=O)C(=O)Nc2ccc(nc2)Oc(cc3)ccc3C	-14	9
Z435980234	c1ccc(OC)cc1OCCN2CCN(CC2)c(cc(n3)C)n(c34)nc(C)c4-c5ccc(Cl)cc5	3	4
Z436400008	c1ccccc1¥C=C¥CS(=O)(=O)C(C)c(on2)nc2C(C)C	6	3
Z438022702	NC(=O)C1CCN(C1)C(=O)/C=C/c(c(n2)Cl)n(c23)cccc3	5	2
Z439257332	c1cc(F)ccc1-c2c(C)nn(c23)c(cc(n3)C(C)(C)C)N(CC4)CCN4CCOc5cc(OC)ccc5	-1	4
Z44104256	c1cc(O)ccc1N(CC2)CCN2Cn(nc3-c(cc4)ccc4C)c(=S)n3-c5c(OC)ccc(c5)OC	10	4
Z44116384	c1ccc(Cl)cc1N(CC2)CCN2Cn(nc3-c(cc4)ccc4C)c(=S)n3-c5c(OC)ccc(c5)OC	31	9
Z44124119	c1ccccc1¥C=C¥CN2CCN(CC2)CN(C(=O)N3)C(=O)C3(C)c(c4)ccc(c45)cc(cc5)OC	1	6
Z441582602	Cc1nn(C(C)(C)C)c(c12)nc(C3CC3)cc2C(=O)NC(C)C	-6	4
Z44255985	FC(F)(F)c1ccc(nc1)N(CC2)CCN2CN(C(=O)N3)C(=O)C34CC(C)(C)CC(C)C4	18	8
Z44304381	c1c(Cl)ccc(c12)NC(=O)C¥2=C¥c3ccc(cc3)OCCCC	6	8
Z44304730	CCCOc(c(cc1)OC)cc1/C=C¥2C(=O)Nc(c23)ccc(Cl)c3	9	7
Z44304760	FC(F)Oc(c(Cl)c1)c(OC)cc1/C=C¥2C(=O)Nc(c23)ccc(Cl)c3	4	4
Z44304769	c1c(Cl)ccc(c12)NC(=O)C¥2=C¥c3c(O)ccc(c3)OCC	23	9
Z44304809	Cc(n1)scc1/C=C¥2C(=O)Nc(c23)ccc(Cl)c3	13	7
Z44305789	c1cc(O)ccc1/C=C¥2C(=O)Nc(c23)cccc3	25	17
Z44305821	COc(cc1)cc(OC)c1/C=C¥2C(=O)Nc(c23)cccc3	37	13
Z44319326	C1CCCCC1NC(=O)C(¥C#N)=C/c2cc(OC)c(cc2)OCC(=O)Nc3ccc(Br)cc3	-16	2

Z443982930	c1ncccc1-c(on2)cc2C(=O)Nc(n3)sc(c34)cc(cc4)S(=O)(=O)N5CCCC5	20	3
Z44489053	Fc1c(F)c(F)ccc1NC(=O)C(C)Nc(c2)ccc(c23)OCCO3	4	8
Z444968022	[nH]1ncnc1Sc(cc2)ccc2NS(=O)(=O)c3c(Cl)cccc3	18	13
Z445169626	c1c(Cl)ccc(Cl)c1S(=O)CCN2CCN(CC2)c3ncnc(c34)n(C)nc4	4	7
Z44556013	c1cccc(c1C#N)NC(=O)C(C)N(C)Cc([nH]c2=O)nc(c23)cccc3	11	3
Z44561353	CC(C)(C)c1ccc(cc1)CN(C)Cc2nc(nc(n2)N)Nc(c3C)cccc3	5	6
Z446209214	Cc1ccc(o1)-c(n[nH]c2)c2CNCc3ccc(nc3)OC	-2	4
Z44828504	CN(C)c(cc1)ccc1/C=N/NC(=O)c2ccc(cc2)N¥N=C¥c3ccc(cc3)N(C)C	16	6
Z44853786	c1cccc(c12)N(C)C(=O)C¥2=N¥NC(=O)c3ccc(cc3)COc(cccc4)c4-c5ccccc5	9	26
Z449089632	CN(C)c(cc1)ccc1CNC(=O)N2CCN(CC2)C3=NS(=O)(=O)c(c34)cccc4	1	6
Z449311908	O1COc(c12)ccc(c2)CCNC(=O)N3CCC3c4ccccc4	1	4
Z449800704	Cc1cc(ccc1)-n(c(C)c2)c(C)c2C(=O)CSc(o3)nnc3-c4cccnc4	-3	6
Z450112784	CC(C)CNC(=O)c1nn(c(c12)CCC2)-c(c3)ccc(F)c3F	-7	7
Z45342622	COc(cc1)c(F)cc1CSCc2nc(nc(n2)N)Nc(c3C)cccc3	6	2
Z45354084	C1CCCCN1C(=O)CSCc2nc(nc(n2)N)Nc(c3C)cccc3	18	14
Z45354574	FC(F)(F)c1ccc(nc1)SCc2nc(nc(n2)N)Nc(c3C)cccc3	12	5
Z45358726	FC(F)(F)c1ccc(nc1)SCc(n2)[nH]c(c23)cc([N+]([O-])=O)cc3	7	2
Z45360560	Cc(c1)n(C)c(C)c1C(=O)CSCc2nc(nc(n2)N)Nc(c3CC)cccc3	-2	0
Z45415135	Cc1c(C)ccc(c1)S(=O)(=O)N2CCN(CC2)S(=O)(=O)c(c3)ccc(C)c3C	-5	11
Z45415601	C1CCCN1S(=O)(=O)c(cc2)ccc2S(=O)(=O)N	-6	9
Z45415602	CCN(CC)S(=O)(=O)c1ccc(cc1)S(=O)(=O)N	1	4
Z45415603	CN(C)S(=O)(=O)c1ccc(cc1)S(=O)(=O)N	11	6
Z45421992	c1cc(Br)ccc1S(=O)(=O)N(CCC)S(=O)(=O)c2ccc(Cl)cc2	-3	9
Z45422005	Cc1ccc(cc1)S(=O)(=O)N(CCC)S(=O)(=O)c2ccc(Cl)cc2	3	7
Z45422032	Cc(c1)ccc(c1C)S(=O)(=O)N(CCC)S(=O)(=O)c2ccc(Cl)cc2	5	8
Z454409356	Clc1c(Cl)ccc(c1)NC(=O)CN(C)C(=O)c(c2O)cnc(c23)nc(C)cc3	45	16
Z45467078	O=S1(=O)CC(CC1)N(CC)S(=O)(=O)c2ccc(Cl)cc2	-33	13
Z45467079	O=S1(=O)CC(CC1)N(CC)S(=O)(=O)c2ccc(Br)cc2	4	12
Z45467092	O=S1(=O)CC(CC1)N(CC)S(=O)(=O)c(cc2)ccc2C	9	3
Z45467101	O=S1(=O)CC(CC1)N(CC)S(=O)(=O)c(c2)ccc(Cl)c2Cl	12	10
Z45467114	O=S1(=O)CC(CC1)N(CC)S(=O)(=O)c(c2C)cc(C)cc2	-1	5
Z45467115	O=S1(=O)CC(CC1)N(CC)S(=O)(=O)c(c2)ccc(C)c2C	10	2
Z45467139	O=S1(=O)CC(CC1)N(CC)S(=O)(=O)c2c(Cl)cc(Br)cc2	2	7
Z45467174	O=S1(=O)CC(CC1)N(CC)S(=O)(=O)c(c(C)c2C)c(C)c(C)c2C	8	5
Z45467189	O=S1(=O)CC(CC1)N(CC)S(=O)(=O)c(c2C)c(C)on2	-5	2
Z45476409	CC(=O)Nc(cc1)cc(c1OC)S(=O)(=O)N(C)Cc2c(OC)c(OC)ccc2	2	5

Z45480638	O=S1(=O)CC(CC1)N(CC(C)C)S(=O)(=O)c2ccc(Cl)cc2	1	6
Z45480661	O=S1(=O)CC(CC1)N(CC(C)C)S(=O)(=O)c(c2)ccc(Cl)c2Cl	1	8
Z45480819	O=S1(=O)CC(CC1)N(CCC)S(=O)(=O)c2c(Cl)cccc2	-1	4
Z45480862	O=S1(=O)CC(CC1)N(CCC)S(=O)(=O)c(c2C)c(C)on2	-1	5
Z45488256	CC(C)(C)c1ccc(cc1)S(=O)(=O)NC(C)c2ccc(cc2)-n3ccnc3	12	4
Z45488257	CC(C)c1ccc(cc1)S(=O)(=O)NC(C)c2ccc(cc2)-n3ccnc3	-8	7
Z45503858	c1cccc(c12)sc(n2)CN3CCN(CC3)S(=O)(=O)c4c(Cl)cccc4Cl	3	3
Z45516173	CCN(CC)S(=O)(=O)c1ccc(cc1)S(=O)(=O)N(C)C	3	5
Z45539789	CC(C)N(C)S(=O)(=O)c1ccc(cc1)S(=O)(=O)N(CC)CC	11	3
Z45549760	N#Cc1ccc(cc1)S(=O)(=O)N(C)S(=O)(=O)c(cc2)ccc2C	3	2
Z45660528	FC(F)(F)c1c(Cl)ccc(c1)S(=O)(=O)Nc2ccc(cc2)N3CCOCC3	-2	5
Z45768220	CN(C)S(=O)(=O)c(ccc1)cc1NC(=S)NC2CCCC(C)C2C	27	5
Z45779271	c1ccccc1CNC(=S)Nc2c(OC)ccc(c2)S(=O)(=O)N(CC)CC	13	7
Z45795780	CC12CC(CC(C)(C)C2)N(C1)C(=S)Nc(cc3)ccc3CCCC	-6	10
Z45931417	c1cc(C(F)(F)F)cc(n2)c1c(=O)n(c23)CCC/3=C¥c4cccnc4	0	5
Z46035726	c1cccc(c12)nc3c(c2C(=O)O)CCCC/3=C¥c4ccccc4	-14	8
Z46035730	CC(=O)Nc(cc1)ccc1/C=C/2CCCc(c3C(=O)O)c2nc(c34)cccc4	5	5
Z46035754	c1cccc(c12)nc3c(c2C(=O)O)CCCC/3=C¥c4ccc(cc4)OC	9	5
Z46035759	c1cccc(c12)nc3c(c2C(=O)O)CCCC/3=C¥c4cc(Cl)c(Cl)cc4	0	7
Z46035763	c1cccc(c12)nc3c(c2C(=O)O)CCCC/3=C¥c(c4C(F)(F)F)cccc4	8	2
Z46035770	c1cccc(c12)nc3c(c2C(=O)O)CCCC/3=C¥c(cc4)ccc4C(=O)O	1	5
Z46035772	c1cccc(c12)nc3c(c2C(=O)O)CCCC/3=C¥c4cc(OC)ccc4	11	21
Z46035805	c1cccc(c12)nc3c(c2C(=O)O)CCCC/3=C¥c4ccsc4	6	10
Z46036098	c1cccc(c12)nc3c(c2C(=O)O)CCCC/3=C¥c4cc(Br)c(O)cc4	-2	7
Z46041361	c1cc(Cl)c([N+]([O-])=O)cc1C(=O)/C=C/c2c(-c3cccnc3)nn(c2)-c4ccccc4	19	1
Z46051719	c1c(Cl)ccc(c12)NC(=O)C/2=C¥c3ccc(cc3)Nc(c4C)c(=O)n(n4C)-c5ccccc5	15	4
Z46060818	s1ccn(c12)c(c(n2)Cl)/C=C/3C(=O)Nc(c34)cccc4	24	10
Z46080472	c1ncccc1¥C=C¥c(n2)oc(c23)ccc(c3)-c4ccccc4	11	2
Z46107807	c1cccc(c12)nc3c(c2C(=O)O)CC(C)CC/3=C¥c4cc(OC)c(O)cc4	-4	10
Z46107814	c1cccc(c12)nc3c(c2C(=O)O)CC(C)CC/3=C¥c4c([N+]([O-])=O)cccc4	10	5
Z46107817	c1cccc(c12)nc3c(c2C(=O)O)CC(C)CC/3=C¥c4c(Cl)cc(Cl)cc4	3	6
Z46107821	c1cccc(c12)nc3c(c2C(=O)O)CC(C)CC/3=C¥c4c(OC)cc(cc4)OC	-10	8
Z46107852	c1cccc(c12)nc3c(c2C(=O)O)CC(C)CC/3=C¥c4c(OC)cccc4	-1	8
Z46107857	c1cccc(c12)nc3c(c2C(=O)O)CC(C)CC/3=C¥c4cc([N+]([O-])=O)c(Cl)cc4	11	2
Z46107859	c1cccc(c12)nc3c(c2C(=O)O)CC(C)CC/3=C¥c4c(C)cccc4	-3	5
Z46107862	N#Cc1ccc(cc1)/C=C/2CC(C)Cc(c3C(=O)O)c2nc(c34)cccc4	-2	2

Z46107886	COC(=O)c1ccc(cc1)/C=C/2CC(C)Cc(c3C(=O)O)c2nc(c34)cccc4	-6	4
Z46108034	c1cccc(c12)nc3c(c2C(=O)O)CC(C)CC/3=C¥c4cc([N+]([O-])=O)c(cc4)NC	11	11
Z46108144	c1cccc(c12)nc3c(c2C(=O)O)CC(C)CC/3=C¥c4cc(OC)c(O)c(Cl)c4	3	6
Z46108733	c1cccc(c12)nc3c(c2C(=O)O)CC(C(C)(C)C)CC¥3=C¥c(c4)ccc(c45)OCO5	8	9
Z46108738	CC(=O)Nc(cc1)ccc1/C=C/2CC(C(C)(C)C)Cc(c3C(=O)O)c2nc(c34)cccc4	26	24
Z46108841	COC(=O)c1ccc(cc1)COc(cc2)c(OC)cc2/C=C¥3CC(C(C)(C)C)Cc(c4C(=O)O)c3nc(c45)cccc5	15	5
Z46114673	c1cccc(c12)nc3c(c2C(=O)O)CN(C)CC¥3=C¥c(c4)ccc(c45)OCO5	-9	5
Z46114693	c1cccc(c12)nc3c(c2C(=O)O)CN(C)CC¥3=C¥c4ccc(F)cc4	-2	3
Z46114695	c1cccc(c12)nc3c(c2C(=O)O)CN(C)CC¥3=C¥c4cc(Br)ccc4	-9	5
Z46114702	c1cccc(c12)nc3c(c2C(=O)O)CN(C)CC¥3=C¥c4ccc(cc4)OC	-7	2
Z46114740	c1cccc(c12)nc3c(c2C(=O)O)CN(C)CC¥3=C¥c4ccc(cc4)-c5ccccc5	8	7
Z46114754	c1cccc(c12)nc3c(c2C(=O)O)CN(C)CC¥3=C¥c4ccc(CC)cc4	0	9
Z46116606	c1cccc(c12)nc3c(c2C(=O)O)CC(C(C)(C)CC)CC¥3=C¥c4ccc(C(C)C)cc4	13	9
Z46116622	c1cccc(c12)nc3c(c2C(=O)O)CC(C(C)(C)CC)CC¥3=C¥c4cc(OC)c(O)c(Br)c4	5	6
Z46116634	c1cccc(c12)nc3c(c2C(=O)O)CC(C(C)(C)CC)CC¥3=C¥c4c(Cl)cccc4Cl	45	5
Z46116650	c1cccc(c12)nc3c(c2C(=O)O)CC(C(C)(C)CC)CC¥3=C¥c4c(Br)cccc4	23	4
Z46116775	c1cccc(c12)nc3c(c2C(=O)O)CC(C(C)(C)CC)CC¥3=C¥c4cc([N+]([O-])=O)c(cc4)Sc5nncn5C	11	3
Z46117088	c1ccc(O)cc1¥C=C¥c2nc(cs2)-c(cc3)ccc3C	9	11
Z46146538	c1cccc(c12)nc3c(c2C(=O)O)CN(CC)CC¥3=C¥c4ccc(F)cc4	5	5
Z46164581	CC(=O)Nc(n1)scc1CN2CCN(CC2)c3c(OC)cccc3	3	3
Z46179735	C1COCCN1CC(=O)Nc(cccc2)c2C(=O)Nc3ccc(cc3)OCC	14	13
Z46204583	c1ccccc1Cn(c(=O)[nH]c2=O)c(N)c2N(CC)C(=O)CN3CCN(CC3)Cc(c4)ccc(c45)OCO5	4	14
Z46204622	CCOC(=O)c1c(-c(c2C)ccc(C)c2)csc1NC(=O)CN3CCN(CC3)Cc(c4)ccc(c45)OCO5	44	4
Z46205433	c1ccccc1Cn(c(=O)n(c2=O)CC)c(N)c2C(=O)CN3CCN(CC3)Cc(c4)ccc(c45)OCO5	1	4
Z462374130	Cc1nn(C(C)(C)C)c(c12)nc(C3CC3)cc2C(=O)NNC(=O)c4c(C)cccc4	2	4
Z46278335	C1CCCCCC1NC(=O)CN2CCN(CC2)S(=O)(=O)c(c3Cl)cccc3Cl	-12	12
Z46301463	COc(cc1)ccc1C2=NN(C(C2)c3ccco3)C(=O)CN(CC)Cc([nH]c4=O)nc(c45)cccc5	-6	15
Z46302626	c1c(Cl)ccc(Cl)c1S(=O)(=O)CCN(C)Cc([nH]c2=O)nc(c23)cccc3	-9	5
Z46302631	c1cc(C)cc(c1C)S(=O)(=O)CCN(C)Cc([nH]c2=O)nc(c23)cccc3	-9	5
Z46302902	c1cccc(c12)CN(CC2)C(=O)CN(C)Cc([nH]c3=O)nc(c34)cccc4	-12	9
Z46356995	c1ccc(OC)cc1N(CC2)CCN2CC(=O)Nc3scc(c3C(=O)OCC)-c4ccc(cc4)OC	9	5
Z46357060	c1ccc(OC)cc1N(CC2)CCN2CC(=O)Nc3scc(c3C(=O)OCC)-c4cc(OC)c(cc4)OC	5	7
Z46378572	c1cccc(c12)cccc2N(CC)C(=O)CN(C)CC(=O)Nc3ccc(Cl)cc3	33	8
Z46385937	c1cc(Cl)cc(c1C#N)NC(=O)CN(C)CC(=O)Nc2cc(OC)ccc2	3	2

Z46402002	c1cc(F)ccc1-c(c2C(=O)OCC)csc2NC(=O)CN3CCN(CC3)Cc4c(OC)ccc(c4)OC	5	6
Z46402006	CCOC(=O)c1c(-c(cc2)ccc2C)csc1NC(=O)CN3CCN(CC3)Cc4c(OC)ccc(c4)OC	13	24
Z46404320	c1ccccc1C(=O)N2CCN(CC2)Cc(n3)n(CC)c(c34)ccc(c4)S(=O)(=O)N5CCOCC5	1	3
Z46404770	COc(cc1)c(OC)cc1-c(c2C(=O)OCC)csc2NC(=O)CN(CC3)CCN3C(=O)c4ccccc4	2	12
Z46405175	CCC1NC(=O)NC(=C1C(=O)OCC)CN2CCN(CC2)Cc([nH]c3=O)nc4sc(c5c34)CCCC5	0	6
Z46455760	c1cccc(c12)cccc2N(CC)C(=O)CN(C)CC(=O)Nc(c3C)c(C)cc(C)c3	-4	11
Z46510296	O1CCCC1CN(CC2)CCN2CC(=O)Nc(ccc3)cc3-c([nH]c4=O)nc(c45)sc(C)c5C	2	3
Z46514499	c1cc(S(=O)(=O)N)ccc1NC(=O)CN2CCCC2c(c3)ccc(c34)OCCO4	4	2
Z46532935	c1cccc(c12)nc(cc2C(=O)O)¥C=C¥c3cc(C)ccc3	46	8
Z46584361	c1cc(Cl)c(Cl)cc1/C=C(¥C2=O)CCc(c23)cc(cc3)OC	30	2
Z46584406	c1ccc(C)cc1/C=C(¥C2=O)CCc(c23)cc(cc3)OC	-6	7
Z46590414	c1cccc(c12)nc(N3CCOCC3)c(c2)¥C=C¥C(=O)c4ccc(cc4)S(=O)(=O)N5CCCC5	1	2
Z466082788	NS(=O)(=O)c1c(C)oc(C)c1C(=O)N2CCN(CC2)Cc3c(F)cccc3Cl	-3	2
Z46615661	CC(C)(C)C(=O)/C=c(s1)/[nH]c(=O)¥c1=C¥c2ccc(cc2)N(C)C	-8	9
Z46615885	CC(C)(C)C(=O)/C=c(s1)/[nH]c(=O)¥c1=C¥c2c(C)cc(cc2)N(CC)CCO	8	5
Z46636102	Cc(c1)ccc(c12)nc3c(c2C(=O)O)CCCC/3=C¥c4cc(O)c(cc4)OC	2	7
Z47217803	COCCOC(=O)C1=C(C)N(c(c(cc2)OC)cc2C)C(=S)NC1c3ccc(cc3)N4CCOCC4	26	16
Z47840324	C1COCCN1CCNC(=O)C(¥C#N)=c2¥n(-c(c3C)ccc(C)c3)c(=O)/c(s2)=C¥c(c4)ccc(c45)OCO5	-11	10
Z47852683	C1COCCN1CCNC(=O)C(¥C#N)=c2¥n(-c(c3C)cc(C)cc3)c(=O)/c(s2)=C¥c4ccc(cc4)OC(F)F	15	8
Z48462494	Cc1cc([N+]([O-])=O)c(cc1)NC(=O)CC(C2=O)SC(=N¥c(c3C)cccc3)¥N2CCCN4CCOCC4	-3	4
Z485637938	n1oc(C)cc1CN(C)C(=O)NCc2ccc(cc2)N3CCCC3=O	-16	10
Z48712692	CC1CCN(CC1)C(=O)Cn(¥c(s2)=C¥C(=O)OCC)c(=O)¥c2=C¥c3cc([N+]([O-])=O)c(cc3)N4CCCCC4	25	6
Z48712790	CC1CCN(CC1)C(=O)Cn(¥c(s2)=C¥C(=O)OCC)c(=O)¥c2=C¥c3cc([N+]([O-])=O)c(cc3)N4CCOCC4	-4	5
Z48770507	c1cc(C)ccc1Cn(¥c(s2)=C¥C(=O)C(C)(C)C)c(=O)¥c2=C¥c3cc([N+]([O-])=O)c(cc3)N4CCOCC4	31	12
Z48805202	c1cccc(c12)NC(/N2)=C(¥C#N)C(=O)CN3CCN(CC3)S(=O)(=O)c(c4C#N)cccc4	-4	7
Z48851233	NC(=O)c1c(O)ccc(c1)-c2csc(n2)NCc3ccc(C)cc3	10	6
Z48857068	COc(cc1)ccc1Nc(n2)scc2-c3ccc(s3)Cl	4	3
Z48950754	CCc1ccc(cc1)-c(nc(c23)ccc(Cl)c3)cc2-c4ccccc4	48	14
Z48950786	c1c(Cl)ccc(c12)nc(-c3ccc(F)cc3)cc2-c4ccccc4	-4	5
Z48950797	c1c(Cl)ccc(c12)nc(-c3cc(OC)ccc3)cc2-c4ccccc4	1	2
Z48950809	c1c(Cl)ccc(c12)nc(-c3ccccc3)cc2-c4ccccc4	17	22

Z48950813	c1ccccc1-c2c(C)c(-c3ccccc3)nc(c24)ccc(Cl)c4	20	13
Z48950825	c1c(Cl)ccc(c12)nc(-c(cc3)ccc3C#N)cc2-c4ccccc4	58	4
Z48950854	Cc(c1)ccc(O)c1-c(nc(c23)ccc(Cl)c3)cc2-c4ccccc4	-11	4
Z48950876	c1c(Cl)ccc(c12)nc(-c3c(O)cccc3)cc2-c4ccccc4	3	8
Z48950878	c1ccccc1-c2c(CCCl)c(-c3ccc(F)cc3)nc(c24)ccc(Cl)c4	10	7
Z48950994	c1cccc(c12)oc(c2)-c(nc(c34)cccc4)cc3-c5ccccc5	-7	3
Z48951009	c1cccc(c12)nc(-c3c(F)cccc3)cc2-c4ccccc4	5	4
Z48951014	c1cccc(c12)nc(-c3ccc(F)cc3)cc2-c4ccccc4	18	12
Z48951025	c1cccc(c12)nc(-c3cc(OC)ccc3)cc2-c4ccccc4	-6	6
Z48951036	c1cccc(c12)nc(-c3ccc(Br)cc3)cc2-c4ccccc4	5	4
Z48951039	c1cccc(c12)nc(-c(cc3)ccc3C)cc2-c4ccccc4	11	6
Z48951041	c1ccccc1-c2c(C)c(-c3ccccc3)nc(c24)cccc4	37	9
Z48951045	c1cccc(c12)nc(-c3ccc(O)cc3)cc2-c4ccccc4	24	22
Z48951047	c1cccc(c12)nc(-c3ccc(Cl)cc3)cc2-c4ccccc4	8	20
Z48951051	c1cccc(c12)nc(-c3c(OC)cccc3)cc2-c4ccccc4	3	1
Z48951053	c1cccc(c12)nc(-c(cc3)ccc3C#N)cc2-c4ccccc4	3	25
Z48951057	c1ccccc1-c2c(C)c(-c3ccc(O)cc3)nc(c24)cccc4	1	6
Z48951059	Fc1cccc(F)c1-c(nc(c23)cccc3)cc2-c4ccccc4	4	8
Z48951073	FC(F)Oc(cc1)ccc1-c(nc(c23)cccc3)c(C)c2-c4ccccc4	4	5
Z48951082	Cc(c1)ccc(O)c1-c(nc(c23)cccc3)cc2-c4ccccc4	1	9
Z48951725	c1cccc(Cl)c1-c2c(C)c(-c3ccccc3)nc(c24)ccc(Cl)c4	-2	7
Z48951729	c1c(Cl)ccc(c12)nc(-c3ccc(O)cc3)cc2-c4c(Cl)cccc4	10	3
Z48951731	c1c(Cl)ccc(c12)nc(-c3ccc(Cl)cc3)cc2-c4c(Cl)cccc4	-3	21
Z48951737	c1c(Cl)ccc(c12)nc(-c(cc3)ccc3C#N)cc2-c4c(Cl)cccc4	1	3
Z48951741	c1cccc(Cl)c1-c2c(C)c(-c3ccc(O)cc3)nc(c24)ccc(Cl)c4	40	3
Z48952473	c1cccc2oc(c3c12)c(-c4ccco4)c5c6c(CCc5n3)cccc6	-4	2
Z48952614	Cc1ccc(cc1)-c2c(CC(C(C)(C)C)CC3)c3nc(c2c45)sc4CCC5	4	5
Z48955788	c1cccc(F)c1-c2c3COc(cccc4)c4c3nc(c25)ccc(Cl)c5	-15	19
Z48955797	c1c(Cl)ccc(c12)nc(-c3c(F)cccc3)cc2-c4c(F)cccc4	11	4
Z48955802	c1c(Cl)ccc(c12)nc(-c3ccc(F)cc3)cc2-c4c(F)cccc4	0	7
Z48955803	c1cccc(F)c1-c2c3CCc(cccc4)c4c3nc(c25)ccc(Cl)c5	15	4
Z48955825	c1c(Cl)ccc(c12)nc(-c3ccccc3)cc2-c4c(F)cccc4	-1	7
Z48955827	c1c(Cl)ccc(c12)nc(-c(cc3)ccc3C)cc2-c4c(F)cccc4	12	4
Z48955829	c1cccc(F)c1-c2c(C)c(-c3ccccc3)nc(c24)ccc(Cl)c4	9	7
Z48955841	c1c(Cl)ccc(c12)nc(-c(cc3)ccc3C#N)cc2-c4c(F)cccc4	3	7
Z48955845	c1cccc(F)c1-c2c(C)c(-c3ccc(O)cc3)nc(c24)ccc(Cl)c4	3	5

Z48955855	c1cccc(F)c1-c2c(C)c(-c3ccc(F)cc3)nc(c24)ccc(Cl)c4	-3	6
Z49539423	c1ccnc(c12)ccc(c2)/C=N/Nc(n3)nc(C)cc3C	-6	16
Z49575448	c1ncccc1C(=O)N¥N=C¥c2cc(OC)c(cc2)OC(=O)c3cccnc3	10	11
Z49584790	O=C(O)c1cc(ccc1)N¥N=C¥c2ccc(cc2)N(c3ccccc3)c4ccccc4	18	17
Z49625857	c1ccc(O)cc1/C=N/Nc2ccnc(c23)cc(Cl)cc3	-13	4
Z49625899	CCOc(c(O)cc1)cc1/C=N/Nc2ccnc(c23)cc(Cl)cc3	9	3
Z49626092	FC(F)Oc(cc1)c(OC)cc1/C=N/Nc2ccnc(c23)cc(Cl)cc3	-10	12
Z49638448	c1cccc(c12)nc(-c3cccnc3)cc2C(=O)N¥N=C¥c4cc(OC)c(cc4)OCc5ccc(Cl)cc5	-1	2
Z49643067	c1cccc(c12)N(CC)C(=O)C¥2=N¥Nc3c(cc([N+]([O-])=O)cc3)S(=O)(=O)Nc(c4C)ccc(C)c4	44	4
Z49646816	c1cc(Br)ccc1S(=O)(=O)N¥N=C¥c2ccc(cc2)N(c3ccccc3)c4ccccc4	27	15
Z49660185	c1c(Br)ccc(c12)NC(=O)C¥2=N/Nc3ccccn3	-9	15
Z49726877	C1CC1C(=O)N/N=C¥2C(=O)Nc(c23)ccc(c3)C(C)C	2	3
Z49732101	c1cccc(c12)nc(C)n(c2=O)¥N=C¥c3c(cccc4)c4cc(c35)cccc5	2	4
Z49889617	c1cc(O)c(OC)cc1/C=N/Nc2ncnc(c23)scc3-c4ccccc4	13	5
Z49890518	c1ncccc1/C=N/Nc2ncnc(c23)sc(C)c3C	2	7
Z49890529	c1cc(O)ccc1/C=N/Nc2ncnc(c23)sc(C)c3C	51	21
Z49890548	c1cc(O)c(OC)cc1/C=N/Nc2ncnc(c23)sc(C)c3C	21	3
Z49890557	c1ccc(Br)cc1/C=N/Nc2ncnc(c23)sc(C)c3C	5	2
Z49890729	c1cnccc1/C=N/Nc2ncnc(c23)sc(C)c3C	3	9
Z49890739	c1ccc(O)cc1/C=N/Nc2ncnc(c23)sc(C)c3C	14	8
Z49890770	COc(c1)c(O)c(OC)cc1/C=N/Nc2ncnc(c23)sc(C)c3C	26	4
Z49890788	c1ccc(C)cc1/C=N/Nc2ncnc(c23)sc(C)c3C	3	5
Z49890825	COc(cc1)c(O)cc1/C=N/Nc2ncnc(c23)sc(C)c3C	4	9
Z49890840	FC(F)(F)c1cc(ccc1)-n(c2Cl)nc(C)c2/C=N/Nc3ncnc(c34)sc(C)c4C	8	4
Z50018323	c1cnccc1/C=N/Nc(cc2C)nc(c23)cc(cc3)OC	3	7
Z50138431	c1cnccc1-c(n2CC)nc(c23)n(c(=O)[nH]c3=O)Cc4ccccc4	-3	2
Z50141536	CCCCn(c(=O)[nH]c1=O)c(c12)nc(n2CCCCC)-c3cc([N+]([O-])=O)c(cc3)Sc(cc4)ccc4C	-20	17
Z508129336	s1c(Cl)ccc1CN(CC)C(=O)NCc2cc(ncc2)N(CC3)CCN3C	9	4
Z51088101	Fc1cccc(F)c1C(=O)OCc2nc(no2)-c3cccs3	8	3
Z51122297	s1cccc1-c(no2)nc2COC(=O)c3c(C)onc3-c4c(F)cccc4Cl	20	8
Z51170832	c1cccc(c12)nc(cc2)C(=O)OCc3nc(no3)-c4cccs4	19	6
Z51180604	Clc1cccc(F)c1C(=O)OCc2nc(no2)-c3cccs3	8	4
Z51187459	CC(=O)Nc(cc1)cc(F)c1C(=O)CSc(n2-c3ccccc3)nnc2-c4c(OC)cccc4	0	6
Z51550903	Cc(o1)nnc1COC(=O)c2cc(nc(c23)n(C(C)C)nc3)-c4ccccc4	-3	2
Z51737757	O=c1ccc(=O)[nH]n1CCC(=O)Nc2ccc(cc2)-n3cnnn3	14	3
Z51750245	NC(=O)c1ccc(cc1)C(=O)N2CCN(CC2)c3ccccn3	0	9

Z51761022	Fc1c(F)c(F)ccc1NC(=O)CN(C)C(=O)c2cc(nc(c23)n(C(C)C)nc3)-c4ccccc4	12	5
Z51786759	c1cccc(O)c1CCC(=O)N2CCN(CC2)c(nc3)ccc3C(F)(F)F	6	6
Z51800409	O=C1CCCc(c12)[nH]c(c2C)C(=O)NCCOc3ccccc3	4	8
Z51820408	c1ccccc1NC(=O)c2cc(nc(c23)n(C(C)C)nc3)-c4ccccc4	-10	12
Z51995316	O=C1CCCc(c12)[nH]c(c2C)C(=O)NCCOc3c(F)cccc3	36	5
Z51995634	O=C1CCCc(c12)[nH]c(c2C)C(=O)NCCCn(n3)c(C)cc3C	3	8
Z51996046	O=C1CCCc(c12)[nH]c(c2C)C(=O)NCCSc3ccc(Br)cc3	8	8
Z52005969	c1ccccc1-c(nc(c23)n(C(C)C)nc2)cc3C(=O)N4CCN(CC4)c5ccc(F)cc5	27	25
Z52012464	s1cccc1CN(C)C(=O)c2cc(nc(c23)n(C(C)C)nc3)-c4ccccc4	9	7
Z52023681	COc(c1)cc(OC)cc1NC(=O)c2cc(nc(c23)n(C(C)C)nc3)-c4ccccc4	-11	6
Z52058293	c1cc(Cl)ccc1C(NC(=O)N)CC(=O)Nc(ccc2)cc2-c3csc(n3)C	-6	6
Z52242496	c1cccc(Cl)c1Sc(nccc2)c2C(=O)/N=c3¥cc[nH]n3C4CCN(C)CC4	5	2
Z52554880	c1ccccc1OCCN2CCN(CC2)CN(C(=O)N3)C(=O)C3(C)c(c4)ccc(c45)cc(cc5)OC	-6	4
Z52670207	c1cc(F)ccc1-c(no2)nc2CN3CCN(CC3)S(=O)(=O)c(c4Cl)cccc4Cl	-5	2
Z52671252	o1cccc1C2=NN(C(C2)c3cccs3)C(=O)CN(C)Cc([nH]c4=O)nc(c45)cccc5	-3	9
Z52744280	c1cccc(F)c1NC(=O)NC(=O)CN(C)C2CCCc(c23)cccc3	-6	1
Z53007232	Cc1ccc(cc1)Nc(nc(n2)N)nc2CN3CCN(CC3)Cc([nH]c4=O)nc(c45)sc(C)c5C	9	4
Z540725378	FC(F)(F)S(=O)(=O)CS(=O)(=O)C	-4	7
Z54072868	c1cccc(c12)n(CC)c(-c3ccccc3)c2C(=O)CSc(n4N)nnc4-c5cccnc5	-13	5
Z54379277	c1cc(F)c(F)cc1C(=O)C(C)OC(=O)c2ccc(cc2)-c3ccc(O)cc3	27	18
Z54410536	c1ccccc1C(=O)CCC(=O)Nc(n2)sc(c23)CN(C)CC3	-2	6
Z54469331	c1sccc1CC(=O)OCc([nH]c2=O)nc(c23)cc(cc3)C(=O)OC	2	5
Z54813762	c1cccc(c12)N(C)C(/C2(C)C)=C¥C(=O)CN(C(=O)N3)C(=O)C34c5c(CCC4)scc5	-20	8
Z55163996	Cc1ccc(cc1)-c(nn2c3=O)c(cccc4)c4c2nc5sc(c6c35)CCCC6	4	7
Z55164044	Cc1c(C)sc(c12)nc3c4c(cccc4)c(nn3c2=O)-c(cc5)ccc5C	-6	3
Z55164116	Cc1ccc(cc1)-c(nn2c3=O)c(cccc4)c4c2nc(c35)scc5-c6cccs6	24	8
Z55404718	s1cccc1-c(cc2C(F)(F)F)nc(c23)n(nc3C)CCc4nc(cs4)-c5ccco5	38	13
Z55426459	c1ccccc1-c(cc2C(F)(F)F)nc(c23)n(nc3C)-c4ccccc4	-6	6
Z55426831	N#CCCn(nc1C)c(c12)nc(cc2C(F)(F)F)-c3cccnc3	-2	3
Z55426833	N#CCCn(nc1C)c(c12)nc(cc2C(F)(F)F)-c3ccccc3	-11	3
Z55516559	C1CCCCN1c(nc2[nH]c(n3)C)c(C#N)c4c2c3nc(c45)N(C(=O)C5)c(cc6)ccc6C	-13	15
Z55589600	C1CCCN1c(nc2N)c(C#N)c3c2c(N)nc(c34)N(C(=O)C4)c(c5)ccc(C)c5C	15	5
Z55589601	C1CCCN1c(nc2N)c(C#N)c3c2c(N)nc(c34)N(C(=O)C4)c(cc5)ccc5C(C)C	6	5
Z55718436	c1cc(F)cc(F)c1-n2c(SCCN)nnc2-c3ccccc3	3	6
Z55723243	c1cc(C(=O)O)cc(n2)c1nc(c2c3c45)c4cccc5ccc3	15	13
Z558380580	c1nc(C)nc(c12)CCN(C2)C(=O)NCc3cc(no3)-c4ccccc4	1	6

Z55871149	Cc1ccc(cc1)-c(cc2C(=O)O)nc(c23)n(nc3C)-c(c4C)ccc(C)c4	40	16
Z55874673	N#CCCn(nc1C)c(c12)nc(cc2C(F)(F)F)-c3ccc(Br)cc3	-4	9
Z55874741	c1ccccc1-c(cc2C(F)(F)F)nc(c23)n(nc3C)CCc4nn[nH]n4	1	6
Z55892329	Cc1ccc(cc1)S(=O)(=O)NC(=O)c2c(C)n(c(C)c2)CCCn3ccnc3	-11	4
Z56576419	FC(F)(F)c1cc(ccc1)N(CC2)CCN2C(=O)c3c(cccc3)NS(=O)(=O)c4c(C)c(C)cc(C)c4C	21	11
Z56595958	c1cccc(c12)[nH]c(n2)C(C#N)C(=O)COc(ccc3)cc3CC	2	8
Z56631499	N#CCCn(nc1C)c(c12)nc(-c(cc3)ccc3C#N)cc2C(=O)OCC	-7	20
Z56631515	N#CCCn(nc1C)c(c12)nc(cc2C(=O)OCC)-c(c3)ccc(c34)OCCO4	17	10
Z56633381	CCOC(=O)c1cc(nc(c12)n(CC)nc2)-c3ccccc3	34	20
Z56643458	Cc1ccc(cc1)-c(cc2C(=O)O)nc(c23)n(C(C)C)nc3	0	4
Z56643534	CC(C)n(nc1)c(c12)nc(cc2C(=O)O)-c3c(F)cccc3	-8	12
Z56643618	CC(C)n(nc1)c(c12)nc(cc2C(=O)O)-c3cc(OC)c(cc3)OC	9	10
Z56643642	CC(C)n(nc1)c(c12)nc(cc2C(=O)O)-c(c3)ccc(c34)OCCO4	2	12
Z56643722	c1cccc(c1C)-c(cc2C(=O)O)nc(c23)n(C(C)C)nc3	8	7
Z56646434	c1cnccc1-c(cc2C(=O)O)nc(c23)onc3C(C)C	-2	8
Z56648330	CCn(nc1)c(c12)nc(cc2C(=O)O)-c3ccccc3	-43	7
Z56755035	CCc1ccc(cc1)-n(c2=O)c(=O)c(c23)cc4c(c3)c(=O)n(c4=O)-c(cc5)ccc5CC	-12	4
Z56755807	ClCCCCS(=O)(=O)NS(=O)(=O)C	-15	9
Z56757812	CCCCS(=O)(=O)C(CC1)CS1(=O)=O	-9	3
Z56757815	Cc1ccc(cc1)S(=O)(=O)C(C2)C(O)CS2(=O)=O	-3	5
Z56757817	c1ccccc1S(=O)(=O)C(CS2(=O)=O)C(C2)S(=O)(=O)c3ccccc3	15	5
Z56757962	O=C(O)/C=C/c1cc(ccc1)NS(=O)(=O)¥C=C¥c2ccccc2	1	4
Z56758188	CS(=O)(=O)C1(S(=O)(=O)C)CCCCC1	14	7
Z56758239	COC(=O)CCS(=O)(=O)CCS(=O)(=O)CCCCCCCCCCCC	17	6
Z56758417	c1cccc(c12)nc(cc2)-c(n3)cc(C(=O)O)c(c4)c3cc5[nH]c(c6c45)cccc6	14	6
Z56758790	Cc1ccc(cc1)S(=O)(=O)/C(S(=O)(=O)c(cc2)ccc2C)=C/c3ccc(cc3)N(C)C	3	9
Z56759577	c1cc(Cl)ccc1S(=O)(=O)C2(CCCC2)S(=O)(=O)c3ccc(Cl)cc3	-18	8
Z56759801	c1ccccc1S(=O)(=O)C2(CCCC2)S(=O)(=O)c3ccccc3	14	23
Z56760799	N#C/C(C#N)=C/C(CCC1)=C(C12)OC(c3ccccc3)=CC=2c4ccccc4	14	7
Z56760858	c1cccc(CC2)c1n(c23)c(-c4ccc(cc4)OC)c[n+]3-c5ccc(cc5)OC	-21	6
Z56761787	c1cccc(c12)c3[n+](c4c2cccc4)c(C)cc(c3)-c5ccccc5	4	4
Z56764753	C1C(Br)C=CS1(=O)=O	3	8
Z56764769	C1CC=CS1(=O)=O	9	8
Z56764970	c1cccc(c1c23)ccc3nc(-c(cc4)nc(c45)cccc5)cc2-c6ccc([N+]([O-])=O)cc6	10	7
Z56767508	C1C=CCP1(=O)N2CCCCCC2	11	2
Z56768422	c1cccc(c12)n3c(n2)c4c5c6c(cc4)c7n(c(=O)c6ccc5c3=O)c8c9c(ccc8)cccc9n7	-6	3

Z56768427	c1ccccc1Nc2nc(N)ncn2	16	5
Z56768488	Cc1ccc(cc1)Nc(c2)c3c(=O)n(-c4ccccc4)c(=O)c5ccc6c(=O)n(c(=O)c2c6c35)-c7ccccc7	2	3
Z56768715	CCN(CC)CCC(S1(=O)=O)S(=O)(=O)CC(C1)c2ccccc2	8	11
Z56768765	c12c3c(N)ccc2n(C)c(=O)cc1c4c(C3=O)cccc4	-16	3
Z56769590	C1CCCCC1NC(=O)N¥N=C¥C2=C(N3CCOCC3)/C(CC2)=C¥c4ccc(cc4)N(CC)CC	-9	7
Z56770599	FC(F)Oc(cc1)ccc1/C=N/Nc2ncnc(c23)n(nc3)-c4ccccc4	-2	6
Z56772171	C1CCCc(c1c23)sc2nc4c5c(cccc5)CCc4c3N	-17	10
Z56773009	c1cccc(c12)n3c(n2CCN(C)C)nc(c3)-c4ccc(cc4)-c5ccccc5	5	26
Z56773378	c1ccccc1/C(Cl)=C¥P(=O)(¥C=C(Cl)¥c2ccccc2)Nc3cc(ccc3)S(=O)(=O)NCc4ccco4	-1	11
Z56774874	c1cccc(c12)sc(n2)C(¥C#N)=C3/C=C(C)N(C(C)=C3)c4ccc(cc4)OC(F)(F)F	32	11
Z56780341	c1cc(O)c(OC)cc1/C=N/Nc(c(c23)cccc3)nnc2-c4ncc[nH]4	5	11
Z56780975	CCOC(=O)C(¥C#N)=C¥C(CCC1)=C(C12)OC(c3ccccc3)=CC=2c4ccccc4	-7	6
Z56781644	COC(=O)C(¥C(=O)C)=C¥C(CCC1)=C(C12)OC(c3ccccc3)=CC=2c4ccccc4	7	5
Z56783141	CCN(CC)c(c1)ccc(c12)P(N(CC)CC)(=Nc(cc3)ccc3C)N(C(=O)O2)c(c4)ccc(Cl)c4Cl	-2	6
Z56784264	c1cc(F)ccc1/C=C(CCC2)¥c(n3)c2c(n(c34)ncn4)-c5ccc(F)cc5	8	10
Z56784273	c1ccccc1/C=C(CCC2)¥c(n3)c2c(n(c34)ncn4)-c5ccccc5	0	8
Z56784274	c1cc(Br)ccc1/C=C(CCC2)¥c(n3)c2c(n(c34)ncn4)-c5ccc(Br)cc5	3	6
Z56784275	c1cc(Cl)ccc1/C=C(CCC2)¥c(n3)c2c(n(c34)ncn4)-c5ccc(Cl)cc5	-10	10
Z56786201	CC(C)(C)c(cc1)ccc1C(=O)Nc(c2C(=O)OCC)n(c(c23)nc4c(n3)cccc4)CCCN5CCOCC5	4	4
Z56786772	c1cc(Br)ccc1Nc2nc(N)ncn2	24	4
Z56786854	c1ccccc1OP2(Oc3ccccc3)=NC(c4ccc(cc4)OC)=NC(=N2)Oc5ccccc5	-1	10
Z56787522	CCN(CC)c(cc1)ccc1C(=O)NC(=S)Nc(n2)scc2-c3cccs3	-9	8
Z56788048	Cc1ccc(cc1)-n(c(C)c2C=O)c3CCc(c4c23)cn(n4)-c5ccccc5	-7	4
Z56789369	C1CCCCN1c(cc2)c([N+]([O-])=O)cc2/C(O)=C(¥C#N)c3nnc(N4CCOCC4)n3-c5ccccc5	-4	2
Z56789382	c1c(Cl)cc(Cl)c(c12)NC(=O)C/2=C(¥C#N)C(=O)c3c(C)n(c(C)c3)-c(cc4C(F)(F)F)ccc4	-23	15
Z56789395	Cc(c1)ccc(c12)NC(=O)C¥2=N¥n(cn3)c(c34)cccc4	3	4
Z56790636	N#CC(C#N)=Cc1c(C)n(-c(cc2)ccc2C)c3CCc(c4c13)cn(n4)-c5ccccc5	-9	7
Z56790662	C1CC1C(=O)NC(C)Cn(c(N)c2C(=O)OCC)c(c23)nc4c(n3)cccc4	-2	10
Z56791293	c1cccc(n2)c1n3c2c(C#N)cc(c4)c3nc(c45)n(nc5C)-c6ccccc6	11	15
Z56791351	c1c(Cl)ccc(Cl)c1-n(c(C)c2)c(C)c2/C=N/Nc(n3)scc3-c4ccc(F)cc4	-8	4
Z56791564	Cc1ccc(cc1)Nc2nc(N)ncn2	23	7
Z56791580	c1cccc(c1C)Nc2nc(N)ncn2	15	6
Z56793119	CC(C)(C)c1c(O)c(C(C)(C)C)cc(c1)-n(c(c23)cc4c(c2)OCCO4)c(n3)-c5cccnc5	6	11
Z56793398	c1ccc(C)c(c1C)OCc(n2)nn(c2c34)cnc4oc(-c5ccccc5)c3-c6ccccc6	20	4
Z56794781	O=S(=O)(F)CCS(=O)(=O)c1ccc(Cl)cc1	4	4
Z56795594	c1cc(Cl)ccc1/C=N/Nc2ncnc(c23)n(nc3)-c4ccccc4	13	12

Z56797998	C1=CC(C2)C(C(C#N)(C12)C(=O)O)c3c(Br)cc(cc3)N(C)C	9	6
Z56798059	c1cccc2c1c(cccc3)c3c(c24)n(-c5ccccc5)c(n4)-c6ccccc6	6	4
Z56798990	c1cc(Cl)cc(Cl)c1/C=C(CCC2)¥c(n3)c2c(n(c34)ncn4)-c5c(Cl)cc(Cl)cc5	3	6
Z56799004	c1cc(C)ccc1/C=C(CCC2)¥c(n3)c2c(n(c34)ncn4)-c(cc5)ccc5C	-7	2
Z56799005	c1cccc(Cl)c1/C=C(CCC2)¥c(n3)c2c(n(c34)ncn4)-c5c(Cl)cccc5	6	4
Z56799636	c1cccc(OC)c1Nc2nc(N)ncn2	-3	6
Z56801351	C1COCCN1Cc2c(O)c(CC)cc(c2O)-c3nn(CN4CCOCC4)c(C)c3-c(n5)n(C)c(c56)cccc6	-9	35
Z56802485	c1cc(Cl)c(Cl)c(Cl)c1-c(nc(c23)cccc3)cc2C(=O)Nc4ccc(cc4)Oc(cc5)ccc5NC(=O)c6cc(-c7c(Cl)c(Cl)c(Cl)cc7)nc(c68)cccc8	7	8
Z56803956	c1ccc(CC)c(c1CC)NC(=O)COC(=O)CC2N(S(=O)(=O)c(cc3)ccc3F)CCc(c24)cccc4	27	7
Z56813369	O=S1(=O)CC(CC1)N(C)S(=O)(=O)c(cc2)ccc2F	-5	8
Z56813446	C1C=C(CBr)CS1(=O)=O	-4	7
Z56816068	Cc1cc(C)nc(n1)SCc2ccc(cc2)-c3[nH]nc(-c4ccccc4)c3-c(n5)[nH]c(c56)cccc6	11	6
Z56818449	C1CCCCCN1c(cc2)c([N+]([O-])=O)cc2C(=O)Nc(cc3)ccc3C4(CCCCC4)c5ccc(cc5)NC(=O)c6cc([N+]([O-])=O)c(cc6)N7CCCCCC7	-7	2
Z56830412	CN(C)c(cc1)ccc1/C=C(CCC2)¥c(c23)nc(-c(cc4)ccc4CC)cc3-c5ccccc5	-14	9
Z56830945	CN(C)c(cc1)ccc1/C(c2ccc(cc2)N(C)C)=N/C(=O)c3cc(OC(F)F)ccc3	-12	4
Z56835405	c1ccccc1-c(c2)c(n(c23)cccc3)C(=O)C(=O)Nc(cc4)cc(c4C)S(=O)(=O)N5CCOCC5	-2	5
Z56839033	Cc1cc(C)n(n1)-c2nc(-c3cccnc3)nc(c24)cccc4	12	7
Z56841676	c1cc(O)c(OC)cc1/C=N/Nc2nc(-c3ccncc3)nc(c24)cccc4	18	7
Z56842178	Cc1n[nH]c(c12)nc3c(C(=O)CC(C)(C)C3)c2-c(c4C)cc(C)n4C5CC5	1	6
Z56849070	Cc1c(Br)ccc(c1)/N=C¥2C(=O)Nc(c23)c(Cl)cc(Cl)c3	1	4
Z56850769	c1ccc(Br)cc1C(n(c23)ncn2)C=C(N3)c4ccccc4	9	5
Z56850824	COCCN(C(=S)S1)C(=O)¥C1=C¥c(c(=O)n(c23)cc(C)cc2)c(n3)N(CC4)CCN4Cc5ccccc5	-11	3
Z56851971	COc(cc1)ccc1CCNC(=O)C(C#N)c(n2)c(nc(c23)cccc3)N(CC4)CCN4Cc5ccccc5	3	12
Z56852588	c1cccc(c12)n(C)c(n2)-c3c(N)n(c(c34)c(=O)n(C)nc4[N+]([O-])=O)C5CCCCC5	5	6
Z56854281	CCN1CCN(CC1)c(nc(c23)cccc2)c(n3)C(C#N)C(=O)NCCc4cc(OC)c(cc4)OC	38	6
Z56854297	O1COc(c12)ccc(c2)CNC(=O)C(C#N)c(n3)c(nc(c34)cccc4)N(CC5)CCN5Cc6ccccc6	-7	1
Z56854331	c1cc(Cl)ccc1C(=O)NCCn(c(c23)nc4c(n3)cccc4)c(N)c2S(=O)(=O)c5ccccc5	9	8
Z56854384	CCOC(=O)c1c(-c(cc2)ccc2C)csc1Nc(n3)c(nc(c34)cccc4)N(CC5)CCN5CC	-3	9
Z56857970	c1cccc(C2=O)c1C(=O)c3c2c(Nc(cc4)ccc4C)cc(c35)n(nn5)-c6ccc(cc6)OC	7	5
Z56857974	Cc1ccc(cc1)-n(nn2)c(c23)cc(Nc4ccc(cc4)OC)c5c3C(=O)c6c(C5=O)cccc6	2	2
Z56857975	Cc1cc(O)c(cc1)-c2cc([nH]n2)-c(cc3)ccc3C	-4	7
Z56859708	COc(cc1)ccc1CCNC(=O)c2c(N)n(CCc3cc(OC)c(cc3)OC)c(c24)nc5c(n4)cccc5	-4	9
Z56862791	CN(C)S(=O)(=O)c1c(Cl)ccc(c1)S(=O)(=O)c(c2)ccc(Cl)c2S(=O)(=O)N(C)C	1	7
Z56863397	N1C(=O)COc(c12)ccc(c2)C(=O)CSc(n3)[nH]c(=O)cc3C	-14	7

Z56863426	C1CN(C)CCN1C(=O)C2=C(Nc(c3C)c(C)ccc3)SC(C2=N)C(=O)Nc4ccc(cc4)OC(F)F	15	14
Z56863942	CCCCC(CC)CN(C(=S)S1)C(=O)¥C1=C¥c(c(=O)n(c23)cccc2C)c(n3)NCCCn4ccnc4	0	10
Z56863983	CCCCCCCCN(C(=S)S1)C(=O)¥C1=C¥c(c(=O)n(c23)cccc2C)c(n3)NCCCN4CCOCC4	0	6
Z56864837	c1ccccc1SC(=O)NC(O2)(C(F)(F)F)Oc(c23)cccc3	42	3
Z56865657	COc(cc1)ccc1C(=O)C2=C(O)C(=O)N(CCN3CCNCC3)C2c4cc(ccc4)Oc5ccccc5	6	7
Z56865693	CC(C)COc(cc1)c(C)cc1C(=O)C2=C(O)C(=O)N(C2c3ccc(F)cc3)CCN4CCOCC4	-1	5
Z56866112	CCOC(=O)c1c(C)c(C)sc1Nc(n2)c(nc(c23)cccc3)N(CC4)CCN4Cc5ccccc5	2	3
Z56867786	COC(=O)c1c(C)c(C)sc1Nc(n2)c(nc(c23)cccc3)N(CC4)CCN4C/C=C/c5ccccc5	11	5
Z56870429	c1cccc(c12)[nH]c(n2)-c3c(-c4ccccc4)n[nH]c3-c5cc(ccc5)S(=O)(=O)N6CCCC6	-20	7
Z56872965	C1COCCN1CCNC(=O)c2c(N)n(CCCCC)c(c23)nc4c(n3)cccc4	48	13
Z56877166	NS(=O)(=O)c1c(Cl)ccc(c1)S(=O)(=O)N	0	2
Z56877204	CN(C)S(=O)(=O)c1c(Cl)ccc(c1)S(=O)(=O)N(C)C	7	4
Z56877233	CN(C)S(=O)(=O)c(ccc1)cc1S(=O)(=O)c2cc(ccc2)S(=O)(=O)N(C)C	7	5
Z56877281	c1ccccc1C(=O)Nc(c2S(=O)(=O)c3ccccc3)n(C(C)c4ccccc4)c(c25)nc6c(n5)cccc6	-3	7
Z56885893	Cc(c1)cc(C)c(c12)[nH]c(=O)cc2-c3ccc([N+]([O-])=O)cc3	14	5
Z56888775	NS(=O)(=O)c1c(Cl)ccc(c1)S(=O)(=O)c(c2)ccc(Cl)c2S(=O)(=O)N	-18	2
Z56896575	c1cccc(c12)sc(n2)NC(=O)COC(=O)c3c(SC)nccc3	-4	8
Z56900652	c1cc(F)ccc1NC(=O)CC(C2=O)SC(¥N2CC3CCCCC3)=N¥c(c4C)c(=O)n(n4C)-c5ccccc5	8	15
Z56913193	c1cc(C)cc(c1C)NC(SC2)=NN=C2c3ccc(s3)Cl	10	10
Z56913268	c1c(Cl)ccc(c12)nc(-c3ccc(N)cc3)cc2-c4ccccc4	-5	5
Z56914008	Cc1ccc(cc1)Nc2sc(C(=O)Nc(ccc3)cc3C(=O)C)c(N)c2C(=O)NCCN4CCOCC4	-3	3
Z56918070	COc1c(Cl)cc(cc1)NC(=O)c2c(N)c(C(=O)Nc(c3)ccc(c34)OCO4)sc2Nc5ccc(cc5)OC(F)F	16	23
Z56919929	c1ccccc1-n(c2=O)[nH]c(C)c2/C(c3ccccc3)=N/NC(=O)c4cc(n[nH]4)-c5ccccc5	1	6
Z56929220	CC(C)(C)C(CC1)Cc(c1c23)sc2nc4c5c(cccc5)c(nn4c3=O)-c(cc6)ccc6C	-24	15
Z56929366	c1nccn1CCCn2/c(=N/c3cccnc3)scc2-c4cccs4	-5	2
Z56931701	c1cc(F)ccc1Nc2nc(N)ncn2	-4	2
Z56931704	c1cccc(F)c1Nc2nc(N)ncn2	12	11
Z56931708	FC(F)(F)c1cc(ccc1)Nc2nc(N)ncn2	13	4
Z56933724	NC(=S)CCn(nc1C)c(c12)nc(-c3ccc(cc3)OC)cc2C(=O)OCC	-5	3
Z56938001	COc(cc1)ccc1Nc2nc(N)ncn2	14	8
Z56941227	c1ccccc1-c(c2C#N)c(-c3ccccc3)oc2NC(=O)COC(=O)c4c(-c5cccnc5)nn(c4)-c6ccccc6	-9	5
Z56952971	COc(cc1)ccc1CCNC(=O)Cn(c2=O)c(CN3CCOCC3)nc4sc(c5c24)CC(C)CC5	-10	4
Z56970107	CC(C)(C)c1ccc(cc1)C(=O)N(C(C2)C=CS2(=O)=O)c(cc3)ccc3C	4	9
Z56979291	C1CCCN1S(=O)(=O)c2ccc(S(=O)(=O)Cl)cc2	9	11
Z56984731	c1cccc(c12)N(C)C(/N2C)=C(¥C#N)C(=O)COC(=O)c(c3c(n4)cccc3)c(c45)CCC/5=C¥c6ccc s6	-20	1

Z56995197	CN(C)S(=O)(=O)c1ccc(S(=O)(=O)Cl)cc1	4	7
Z57042877	Cc(c1)cc(C)c(c1C)NC(=O)C2CCN(CC2)c3ncnc(c34)sc(C)c4C	0	5
Z57049315	C1COCCN1CCNc2nc(Nc3ccc(cc3)OC)nc(n2)Nc4ccc(cc4)OC	6	7
Z57056476	C1C(Br)C(Cl)=CS1(=O)=O	1	5
Z57056477	O=S1(=O)CC(CC1)SSC(CC2)CS2(=O)=O	3	11
Z57056479	O=S1(=O)CC(CC1)S(=O)(=O)c2ccccc2	4	0
Z57163399	C1CC(S(=O)(=O)Cl)CS1(=O)=O	-7	7
Z57163400	C1C=C(S(=O)(=O)Cl)CS1(=O)=O	4	7
Z57172226	c1cc(C)ccc1C(=O)CC2(O)C(=O)Nc(c23)ccc(Cl)c3	2	6
Z57229152	c1cccc(c1c2c34)c3nn(c(=O)c4ccc2)-c5ccccc5	18	10
Z57237929	o1cccc1C(=O)C2=C(O)C(=O)N(C2c3ccc(Cl)cc3)CCCn4ccnc4	20	26
Z57239420	COc(cc1)ccc1C(=O)NC(¥C(=O)NCCOC)=C¥c2ccc(o2)-c3cc(Cl)ccc3	46	10
Z57239426	c1nccn1CCCNC(=O)/C(NC(=O)c2ccccc2)=C/c3ccc(o3)-c4cc(Cl)ccc4	2	3
Z57239976	c1cc(Cl)ccc1C(=O)C2=C(O)C(=O)N(C2c3ccc(F)cc3)CCCn4ccnc4	-5	9
Z57289339	Cc1ccnc(n1)Nc(n2)sc(c23)cccc3	-13	3
Z57289575	c1cccn(c12)cc(n2)-c3cc(ccc3)NS(=O)(=O)c4ccccc4	41	13
Z573181936	n1ccccc1NC(=O)C2CCCN(C2)Cn(n3)c(=S)n(Cc4ccccc4)c3N5CCOCC5	-31	3
Z573186768	Cc1cc(ncc1)NC(=O)C2CCN(CC2)Cn(n3)c(=S)n(c34)c5c(cccc5)c(=O)n4CCC	-4	5
Z573238670	Cc1cccc(n1)NC(=O)C2CCCN(C2)c3nc(nc(c34)sc(C)c4C)CN5CCOCC5	-5	5
Z573928840	c1ccc(C(F)(F)F)cc1C(=O)Nc2c(O)ccc(c2)N3CCCS3(=O)=O	-18	24
Z57393573	s1c(Br)ccc1-c(n[nH]2)cc2C(=O)N¥N=C¥c3ccco3	-9	15
Z57440306	C1COCCN1CCN(CN2)CN=C2Nc(nc(c34)ccc(Cl)c4)nc3-c5ccccc5	4	6
Z57455467	c1ccccc1-c2cn(-c3ccc(F)cc3)c(c24)ncnc4N5CCOCC5	5	7
Z57455630	c1ccccc1-c2cn(-c(cc3C)ccc3)c(c24)ncnc4N(CC5)CCN5C	9	5
Z57455872	OCCCNc1ncnc(c12)n(-c3ccc(cc3)OC)cc2-c4ccccc4	1	3
Z57455912	COCCNc1ncnc(c12)n(-c3ccc(cc3)OC)cc2-c4ccccc4	16	12
Z57455953	OCCNc1ncnc(c12)n(-c3ccc(F)cc3)cc2-c4ccccc4	-6	6
Z57455954	OCCCNc1ncnc(c12)n(-c3ccccc3)cc2-c4ccccc4	5	5
Z57456069	OCCNc1ncnc(c12)n(-c3ccc(Cl)cc3)cc2-c4ccccc4	8	5
Z57456112	CN1CCN(CC1)c2ncnc(c23)n(-c4cc(Cl)ccc4)cc3-c5ccccc5	13	4
Z57456127	OCCOCCNc1ncnc(c12)n(-c3ccc(cc3)OC)cc2-c4ccccc4	-8	7
Z57456164	OCCNc1ncnc(c12)n(-c3ccc(cc3)OCC)cc2-c4ccccc4	20	5
Z57458515	c1cc(C)ccc1Cn(nc2)c(c23)ncnc3NN	9	9
Z57460727	c1ccccc1C(c2ccccc2)N3CCN(CC3)c4ncnc(c45)n(nc5)Cc6ccc(Cl)cc6	-5	5
Z57514609	CCS(=O)(=O)Nc(cc1)ccc1C2=NN(C(C2)c3ccco3)S(=O)(=O)c(cc4)ccc4C	23	19
Z57604399	c1c(F)ccc(O)c1C2=NNC(C2)c3cc(Br)cc(c34)COCO4	-12	3

Z57669031	S1CCSC1(C)CC(=O)N¥N=C¥c2ccc(o2)Br	1	4
Z57724322	c1cccc(c12)n3c(c(C#N)c(C)cc3=O)n2CC(O)CN4CCN(CC4)CCOc5ccc(Cl)cc5	7	11
Z57805545	OCCNC(=O)COc(cc1)ccc1-c(nc(c23)ccc(Cl)c3)cc2-c4ccccc4	39	3
Z57981090	c1cccc(c12)n(c(n2)NCCO)-c3ncnc4sc(c5c34)CCCC5	7	4
Z57984419	c1cccc2c1c(cccc3)c3c(c24)[nH]c(n4)-c5cc(ccc5)Oc6ccc(Cl)cc6	2	3
Z57989376	c1ccccc1-c2c(C#N)c(-c3ccc(Cl)cc3)nc(c24)cccc4	2	5
Z57991317	c1cccc(Cl)c1-c2c(Cl)c(-c3ccc(Br)cc3)nc(c24)ccc(Cl)c4	32	11
Z58286917	c1ccccc1Cn(nc2)c(c23)ncnc3N4CCCC4	0	9
Z58286928	c1ccccc1Cn(nc2)c(c23)ncnc3NN	-12	2
Z58287009	c1ccccc1¥C=C¥CN2CCN(CC2)c3ncnc(c34)n(nc4)Cc5ccccc5	0	4
Z58490632	c1ccnc(c1C)NC(=O)c2ccc(cc2)CSc3ccccc3	-1	4
Z594627070	c1cc(Cl)cc(c12)nc([nH]c2=O)C(C#N)C(=O)CCc3ccc(F)cc3	1	8
Z594884744	C1CCCC(C12)OCCN2C(=O)Nc3ccc(cc3)S(=O)(=O)N(CC)c4ccccc4	-4	2
Z594945904	s1cccc1C(=O)NCCC(=O)Nc(c2)ccc(c23)[nH]c(n3)-c4cc(F)ccc4	-4	8
Z595406192	c1ncccc1C(=O)N2CCN(CC2)C(=O)Nc(cc3)cc(F)c3N(C)C4CCCCC4	2	9
Z596098802	C1COCCN1C(=O)CCNC(=O)c2c(O)c(Br)cc(C)c2	7	10
Z60362928	O1COc(c12)ccc(c2)CNC(=O)c3cc(C4CC4)nc(c35)n(C(CC6)CS6(=O)=O)nc5C	5	6
Z605682156	c1ccccc1-n(c2C)nc(C)c2C(=O)C(=O)Nc(c3)ccc(OC)c3OCc(n4)cn(c45)cccc5	-5	5
Z60779201	NC(=O)c1ccc(cc1)NC(=O)c2cc(C3CC3)nc(c24)n(C(CC5)CS5(=O)=O)nc4C	17	11
Z62746884	o1cnnc1-c2ccc(cc2)OCC(=O)Nc3c(OC)cc(c(c3)OC)NC(=O)c4ccccc4	46	18
Z62804747	o1cccc1CNC(=O)CN(C)C(=O)/C=C/c2ccc(cc2)-n3ccnc3	2	4
Z635062124	C1CC(O)CCC1NCc2c(n[nH]c2)-c3cc(OC)ccc3	3	6
Z635707390	Clc1n(C)nc(C(C)C)c1CNCc2cc(F)c(cc2)N3CCOCC3	-12	19
Z641940000	CC(C)N(C1=O)S(=O)(=O)c(c12)cc(cc2)C(=O)Nc(n3)n(CCC)c(c34)cccc4	10	5
Z642699568	FC(F)(F)c1ccnc(n1)Nc2ccc(cc2)N3CCCCCC3	-21	10
Z643690740	C1CC1C(=O)N(C)S(=O)(=O)c(c2)c(Br)cc(OC)c2OC	0	3
Z644822950	N#Cc1ccc(nc1)NC(C)c2ccc(cc2)OCc3cccnc3	2	6
Z645121524	c1coc(C)c1C(=O)Nc2c(F)ccc(c2)Nc(n3)nccc3C(F)(F)F	14	5
Z64526536	c1cc(F)ccc1NC(=O)CN2CCN(CC2)CN(C(=O)N3)C(=O)C3(C)c(c4)ccc(c45)cccc5	9	12
Z645525338	COCCOc(nc1)ccc1CNc2ncnc(c23)cccc3	-2	5
Z645555414	CCOC(=O)NC1CCCN(C1)c2ncnc(c23)n(C)nc3	-2	6
Z645593692	O1COc(c12)ccc(c2)OCCNc3ncnc(c34)n(C)nc4	-38	14
Z645877938	c1ccccc1C(C)(C)C(=O)N2CCN(CC2)C(=O)C(CN3)CCC3=O	-20	21
Z646031500	c1ccc(C)c(c1C)OC2CCN(CC2)c3ncnc(c34)n(C)nc4	8	2
Z646341564	n1ccncc1NC(=O)C2CCCN(C2)c3ncnc(c34)n(C)nc4	42	4
Z646432252	FC(F)(F)c1ccnc(n1)Nc(cc2)ccc2CN(C3=O)CCO3	11	5

Z646439314	FC(F)(F)c1ccnc(n1)Nc2cnn(C)c2	5	7
Z64655268	CC(C)(C)NC(=O)CN(CC1)CCN1C(=O)CN(C)c(ncn2)c(c23)sc(c3)-c4ccc(F)cc4	6	9
Z64660968	c1ccc(F)cc1NC(=O)CN2CCN(CC2)c3nc(nc(c34)sc(C)c4C)CN5CCOCC5	6	8
Z64681160	c1cc(F)ccc1NC(=O)CN2CCN(CC2)c3nc(nc(c34)sc(C)c4C)CN5CCOCC5	1	6
Z647256318	c1cccc(c12)[nH]c(n2)C(C)NC(=O)Cn(n3)c(=O)ccc3-c4ccccc4	5	3
Z647588818	n1cccn1-c(nccc2)c2CNC(=O)c3ccc(cc3)NC(=O)c4ccccn4	-11	8
Z64779311	CCN(S(=O)(=O)C)c(cc1)ccc1-c2csc(n2)Nc3ccc(cc3)OC	-4	2
Z647976584	CCOC(=O)c1c(C)c([nH]c1C)C(=O)NC(C)Cn2ccnc2	5	8
Z649184702	c1nccc(O)c1NC(=O)c(cc2)cc(c23)CC(OC3=O)c4ccccc4	-7	7
Z649200126	n1ccccc1NC(=O)CCNC(=O)c(c2)[nH]c(c23)ccc(F)c3	-10	2
Z649805708	CC(C)c(cc1)cc(c12)[nH]c(c2)C(=O)NCCN(CC3)CCS3(=O)=O	-31	20
Z651750688	n1cc(C)ncc1C(=O)Nc(c2)ccc(c23)[nH]c(n3)-c4ccc(F)cc4	-2	2
Z652718652	CS(=O)(=O)c(c1F)cc(cc1)S(=O)(=O)C	31	21
Z653343234	n1ccccc1-c2nnc([nH]2)NC(=O)CCc3cnn(c3)-c4ccccc4	-14	11
Z653347886	Cc(c1)ccc(c12)[nH]c(C)c2CC(=O)Nc([nH]3)nnc3-c4ccccn4	-3	6
Z653349694	n1ccccc1-c2nnc([nH]2)NC(=O)c3ccc(nc3)OC(C)c4ccccc4	-4	7
Z65478686	Cn(c1)ncc1/C=N/Nc2ccnc(c23)cc(Cl)cc3	2	4
Z65566827	CCc1ccc(cc1)S(=O)(=O)N(CC)C(CC2)CS2(=O)=O	2	6
Z65599853	c1cc(S(=O)(=O)N)ccc1C(C)N(C)S(=O)(=O)c2cc(Cl)cc(Cl)c2	0	4
Z65695011	CC(C)(C)NC(=O)CN(CC1)CCN1C(=O)CSc(nc(c23)cccc3)n(c2=O)-c4ccccc4	57	7
Z65734306	CC(C)(C)NC(=O)CN(CC1)CCN1C(=O)CSc(n2)n(c(c23)cccc3)-c4ccc(cc4)OC	10	6
Z65769442	c1cccc(c12)OCC(c3ccccc3)N2C(=O)CSc(n4C)nnc4-c5ccncc5	-1	6
Z65980497	c1cccc(c12)OCC(c3ccccc3)N2C(=O)CSc(n4C)nnc4-c5ccc(cc5)OC	-3	6
Z66064269	NS(=O)(=O)c(c1)ccc(c12)n(CCCC)c(n2)CSc([nH]n3)nc3-c4ccc(cc4)OC	4	2
Z666133744	CS(=O)(=O)c(cc1)cc(S(=O)(=O)C)c1N2CCC2	8	5
Z666139280	CC(C)CN(CC)c1c(S(=O)(=O)C)cc(cc1)S(=O)(=O)C	7	7
Z666144310	CS(=O)(=O)c(cc1)cc(S(=O)(=O)C)c1N2CCSCC2	0	6
Z666145136	C1CC1CN(C)c2c(S(=O)(=O)C)cc(cc2)S(=O)(=O)C	52	8
Z666252570	NC(=O)COc(cc1)c(OC)cc1CNC(=O)c2c(O)nccc2	1	6
Z666279820	c1nc(O)ccc1C(=O)Nc(cc2)ccc2-c(n3)cn(c34)cccc4C	-9	3
Z66976986	c1cc(F)ccc1-c(s2)ccc2C(=O)Nc(ccc3)cc3/C=C/c4ccccn4	10	10
Z68435865	s1ccn2c1nc(c23)sc(c3)C(=O)Nc(n4)sc(c45)CN(CC5)Cc6ccccc6	-4	4
Z70775126	O=C1CCC(N1)C(=O)Nc(c2)ccc(OC(F)F)c2-c3ccc(cc3)OC	-6	4
Z70909087	Cc(n1)oc(c12)ccc(c2)NC(=O)COc(cc3)ccc3N(C)S(=O)(=O)c(cc4)ccc4C	17	8
Z70909650	Cc(n1)oc(c12)ccc(c2)NC(=O)COc3ccc(cc3)OCCC	11	5
Z70970124	[O-][N+](=O)c(c1)ccc(N(CC)CC)c1C(=O)Nc(cc2)cc(c23)sc(n3)N4CCOCC4	9	3

Z70997275	c1ccccc1-c(n2)c(n(c23)cc(C)cc3)NC(=O)C(CC4=O)CN4CCc5cc(OC)c(cc5)OC	-15	11
Z71311814	Cc1c(C)cc(C)c(C)c1S(=O)(=O)N(C)C(CC2)CS2(=O)=O	1	9
Z71311982	O=S1(=O)CC(CC1)N(C)S(=O)(=O)c(c2)ccc(Cl)c2Cl	-2	2
Z727167436	[nH]1cnnc1NC(=O)c2c(C)nn(c2C)-c3ccc(Br)cc3	-2	4
Z728482294	c1cc(C)c(F)cc1C(=O)Nc2ccc(cc2)OCc3nccn3C	50	7
Z729056288	FC(F)(F)c1ccnc(n1)Nc2ccc(NC)nc2	-20	10
Z729056808	NC(=O)c1cc(Cl)c(nc1)NCc2cc(ncc2)N3CCCCC3	2	6
Z729060218	FC(F)(F)c1ccnc(n1)Nc2cnn(c2)CC	6	3
Z729700802	CCCNC(=O)CN(CCC)C(=O)c1cc(nc(c12)n(CC)nc2)-c3ccccc3	0	7
Z729741578	c1noc(C)c1C(=O)Nc2ccc(cc2)NC(=O)c(c3)[nH]c(c34)cc(Br)cc4	39	29
Z729744666	c1cc(F)cc(c12)NC(=O)CC2C(=O)Nc([nH]n3)cc3CC4CCCCC4	22	15
Z729842360	CN(C)c(cc1)ccc1CN(C(C)C)Cn(n2)c(=S)n(c23)c4c(cccc4)c(=O)n3CCCOC	-9	7
Z729847414	C1CCCN1c(ccc2)cc2Nc3c([N+]([O-])=O)cc([N+]([O-])=O)cc3	20	13
Z729847600	c1cnccc1C(=O)NC2CCN(CC2)c3nc(CN(CC4)CCC4C)nc(c35)sc(C)c5C	3	5
Z730571712	c1cc(Cl)ccc1NC(=O)CCCNC(=O)c2ccc(o2)-c3c(F)cccc3	2	4
Z73300898	s1cccc1C(=O)Nc(cc2)ccc2C(=O)Nc(n3)scc3-c4ccccn4	5	2
Z73364722	CC(=O)NCc1ccc(s1)-c2csc(n2)NC(=O)c3c(C)oc(n3)-c4ccccc4	-1	4
Z73381327	n1c[nH]c(c12)ccc(c2)C(=O)NCCOc3ccc(Cl)cc3	12	2
Z734126534	O=S1(=O)CC(CC1)N(CC#C)S(=O)(=O)c(c2F)cccc2	-10	5
Z734296158	c1cccc2c1c(=O)c(C#N)c(n3C)n2c(c34)cccc4	-2	5
Z734467112	c1cccc(c12)n3c(n2C)c(C#N)c(=O)c4c3cccc4F	-1	3
Z73456724	C1COCCN1C(=O)CN(CC2)CCN2C(=O)c3c(-c(cc4)ccc4C)nn(c3)Cc5ccccc5	10	9
Z73457343	C1COCCN1C(=O)CN(CC2)CCN2C(=O)c3cc(nc(c34)n(C(C)C)nc4)-c5ccccc5	7	9
Z735448522	CC(C)(C)n(n1)c(C2CC2)cc1C(=O)Nc(ccc3)cc3C4SCCCS4	2	24
Z736057568	c1cc(Br)cn(c12)cc(n2)CNCc3cnc(nc3)-c4ccccc4	4	4
Z736543988	CCS(=O)(=O)c1ccc(cc1)S(=O)(=O)N2CCCSCC2	32	17
Z736710192	CC(C)N(CC)S(=O)(=O)c(cc1)ccc1S(=O)(=O)C	-4	4
Z737550454	C¥C=C¥C=C¥C(=O)NCc(ccc1)cc1C(=O)NCc2ccco2	0	7
Z738379554	O1CCCC1C(=O)Nc(cc2)cc(Cl)c2C(=O)N(C)C3CCCCC3	3	2
Z73973253	CC(C)(C)C(=O)NCc(s1)ccc1C(=O)COC(=O)c(c2)sc(c23)CCC3	9	5
Z740170140	CN(C)S(=O)(=O)c(c1C)cc(cc1)S(=O)(=O)C	1	1
Z740420934	NC(=O)c1ccc(cc1)N(C)C(=O)c2ccc(cc2)C3SCCCS3	0	2
Z741831914	O=S1(=O)CC(CC1)N(CC#C)S(=O)(=O)c(cc2)cc(Cl)c2OC	-5	8
Z747178828	c1cccc(c12)n(C)c(c2)C(=O)Nc([nH]3)nnc3-c4ccccn4	-6	16
Z74745150	O1COc(c12)ccc(c2)CN(C)S(=O)(=O)c(cc3)cc(Br)c3OC	-25	3
Z751335838	c1cccc(c12)n(C)c(c2)C(=O)Nc(cc3)cc(Cl)c3NC(=O)c4ccco4	55	9

Z751573116	n1cccn1CCNc2nc(C)nc(c23)n(nc3)-c4ccccc4	14	10
Z751922260	c1[nH]nc(C)c1CNCc2ccc(nc2)N3CCCCCC3	17	21
Z751922524	CC(C)(C)c1c(c[nH]n1)CNCc2ccc(cc2)COC(C)C	13	3
Z754942410	Brc1cc(Cl)cc(c1OC(F)F)NC(=O)C(=O)N2CCCSCC2	13	7
Z757638538	Cn1ccnc1C(C(F)(F)F)(O)CC(=O)Nc2ccc(cc2)Oc3ncccn3	-7	6
Z757780044	c1nc(Cl)ccc1C(=O)Nc(c2)ccc(c23)[nH]c(n3)-c4ccco4	51	17
Z757830056	C1C(C2)CC(C3)CC2CC13C(=O)NCC(=O)Nc4cnc(nc4)-c5cc(F)ccc5	-9	4
Z762214972	o1cccc1C(=O)Nc2ccc(cc2)OC(=O)c(c3)n(C)c(c34)cccc4	-1	7
Z76798588	c1cccc(c12)NC(/N2)=C(¥C#N)C(=O)COC(=O)CSc(nc3)nc(N)c3C(=O)OCC	5	11
Z76887050	C1CC(C)Cc(c1c23)sc2nc4c(c3-c5ccco5)c6c(CC4)cccc6	8	6
Z785638578	NC(=O)Nc(cc1)ccc1-c(cs2)nc2CCc3ccccc3	0	12
Z786279284	CC(C)N(C)C1CCN(CC1)c2nc(-c3ccncc3)nc(c24)cccc4	4	1
Z79434439	C1COCCN1C(=O)/C=C/c2c(C)n(c(C)c2)-c3ccnn3C(C)C	15	11
Z79575803	Cc1onc(C)c1CC(=O)N¥N=C(¥c2ccccc2)c3ccccc3	-9	14
Z79604730	CCN(CC)CCNC(=O)c1ccc(cc1)NC(=O)C2CCN(CC2)c3ncnc(c34)sc(C)c4C	-1	6
Z796939908	s1c(Cl)ccc1-c(c[nH]c2)c2C(=O)OCc3c(C)noc3C	0	5
Z803067872	NCCN(C1=O)C(=O)C(C12)C3C=CC2C3	7	3
Z805455424	CCOC(=O)N(C)c1ccc(cc1)Nc(n2)nccc2C(F)(F)F	-19	8
Z805631084	CS(=O)(=O)c(c1)ccc(Br)c1S(=O)(=O)N2CCSCC2	0	8
Z805634984	CCS(=O)(=O)N1CCN(CC1)S(=O)(=O)C(CC2)CS2(=O)=O	2	4
Z805651486	c1cc(I)ccc1N(C)S(=O)(=O)c2cn[nH]c2	-1	4
Z808591212	CS(=O)(=O)CCS(=O)(=O)c(cc1)ccc1N	-1	2
Z809056560	C1CCn(c12)c(nn2)C3CCN(CC3)C(=O)c4ccc(cc4)OCC5CC5	2	4
Z815677014	FC(F)Oc1cccc(c12)CCCN2S(=O)(=O)c(c3)ccc(c34)NC(=O)C4	-4	3
Z815791244	CCCN1CCN(CC1)c2c(Cl)cc(cc2)Nc(cc3)nn(c34)nnn4	-24	9
Z815806580	c1cc(F)ccc1OCC(O)CNc(cc2C)nc(c23)cccc3	-15	6
Z815820560	c1nn(C)c(c12)nc(C)nc2NCC3CCN(C3)c4ccc(F)cc4	-4	4
Z815840280	c1cccc(c12)ncc(c2)NC(=O)c3ccc(cc3)-n(n4)ccc4C(F)(F)F	9	5
Z816021628	c1cc(C(=O)N)cc(F)c1COc2ccc(I)cc2	-2	4
Z81619452	CC(C)(C)NC(=O)CN(CC1)CCN1C(=O)Cn(c2=O)cnc(c23)scc3-c(c4)ccc(C)c4C	-8	5
Z817034034	OCC1CCCN1c2ncnc(c23)n(C)nc3	3	6
Z817738176	c1ccnc(Br)c1OC(=O)c2nn(c(=O)c(c23)cccc3)-c4ccccc4	23	18
Z818806818	Fc1c(Cl)cc(cc1)NC(=O)c(c2)sc(c23)n(C)c(=O)n(C)c3=O	-3	7
Z821199838	CC(C)(C)c1c(c[nH]n1)/C=C¥2CCn(c23)c(=O)c4c(n3)cc(Cl)cc4	-3	9
Z82176567	c1cccc(c1c23)Cc2ccc(c3)-c(n4)cn(c45)cc(cc5)S(=O)(=O)N	27	19
Z822335078	CS(=O)(=O)CCS(=O)(=O)Cc1cc(C)ccc1	-2	6

Z825945954	CC(C)(C)c(s1)ncc1C(=O)Nc(c2)ccc(F)c2NC(=O)C3CC3	-24	9
Z826157056	CS(=O)(=O)c(cc1)ccc1C(=O)NCc2cc(F)c(cc2)-n3ccnc3	-29	3
Z826159196	CC(C)n(nc1)c(c12)ncc(c2)C(=O)NCc3cc(F)c(cc3)-n4ccnc4	9	7
Z826666316	Cc1c(C)cccc1NC(=O)CN(CC2)CCN2C(=O)CN(C(=O)C3)C(=O)c(c4)n3c(c45)cccc5	-25	1
Z826713438	N#CCCn(n1)c(cc1C)NC(=O)c2ccn(n2)-c3ccccc3	5	5
Z826713788	N#CCCn(n1)c(cc1C)NC(=O)/C=C/c2csc(n2)C	-15	6
Z826713980	N#CCCn(n1)c(cc1C)NC(=O)c(c2)sc(c23)cccc3F	-5	2
Z826941792	c1ccc(O)cc1CN(C2CC2)c(ncn3)c(c34)scc4	1	4
Z827167912	O=S1(=O)CC(CC1)N(CC2)CCN2Cc3c(O)c(cc(Cl)c3)C4CCCCC4	7	3
Z827524892	n1ccccc1NC(=O)N2CCN(CC2C)C(=O)Nc3ccccn3	-21	7
Z830071152	O=S1(=O)CC(CC1)N(CCC)S(=O)(=O)CCC	16	5
Z838103434	c1ccc(C)c(c1C)OCC(O)CNc2ncnc(c23)sc(C)c3C	13	6
Z839335172	o1cccc1¥C=C¥C=N¥NC(=O)c2ccc(s2)-c3ccc(F)cc3	6	3
Z839413590	n1cccn1-c(cccc2)c2C(=O)/C=C/c3ccc(cc3)NC(=O)C	-15	3
Z839958866	CCOC(=O)COc(cc1)ccc1C(C)(NC2=O)Nc(c23)ccc(Cl)c3	5	7
Z839968520	CC(=O)c1c(CC)c([nH]c1C)C(=O)CN2CCCC2c3ccsc3	0	5
Z84671779	C1COCCN1C(c2cccs2)CNC(=O)CNC(=O)c3ccc(cc3)OCCCC	6	6
Z847574324	s1c(Cl)ccc1-c(c[nH]c2)c2C(=O)OCc3nc(on3)C4CC4	10	4
Z850607168	CC(C)(C)C(=O)Nc(cc1)cc(Cl)c1NC(=O)C(C)n(n2)c(C)cc2C	3	3
Z850748812	C1C(C2)CC(C3)CC2CC13C(¥C)=C/C(=O)N4CCN(CC4)c5cnccn5	12	15
Z85145044	CCN(CC1)CCC1NC(=O)C2CCN(CC2)S(=O)(=O)c3c(C)c(C)cc(C)c3C	6	4
Z854317650	Cc1ccc(s1)C(=N2)OC(=O)/C2=C¥c3ccc(cc3)OCc4ncon4	5	6
Z855152014	C1CC1c(n[nH]2)c(Br)c2C(=O)N(C3CC3)CC4CCOC4	15	7
Z856541214	C1COCCC1(C(=O)N)CNC(=O)c(c2)c(O)cc(c23)CCCC3	17	11
Z85927834	CCCCN(S(=O)(=O)C)S(=O)(=O)c(cc1)ccc1OC	1	2
Z85929120	Cc1cc(O)c(cc1)Nc2ncnc3sc(c4c23)CCCC4	2	17
Z85929771	o1cccc1C(=O)OCC(=O)Nc(n2)sc(c23)cc([N+]([O-])=O)cc3	10	3
Z85934217	c1cccc(c1c2c34)oc4nc(N)c(C#N)c3nc(c2)-c5ccc(cc5)OC	5	3
Z86108588	C1CCCc(c1c23)sc2nc(CN4CCOCC4)n(c3=O)CC(=O)Nc5ccc(cc5)OCc6ccccc6	16	8
Z86212654	c1cc(F)cc(F)c1NC(=O)NC(C)c2ccc(cc2)-n3cncn3	-3	7
Z86240485	c1cccc(c12)nc(C(F)(F)F)nc2NCC(O)c3ccc([N+]([O-])=O)cc3	-1	13
Z86979745	c1cc([N+]([O-])=O)cc(Cl)c1C(=O)Nc([nH]n2)nc2-c3ccccc3	-11	13
Z87002147	CS(=O)(=O)c1c(Cl)ccc(c1)S(=O)(=O)C	0	8
Z872035512	COC(=O)c(c1C)sc(c12)ncc(c2)/C=C¥3C(=O)Nc(c34)cccc4	-4	5
Z873001918	c1cnccc1CNC(=O)CCc2c(C)nc([nH]c2=O)-c3ccncc3	12	6
Z875041828	CS(=O)(=O)CCS(=O)(=O)N	2	2

Z87524039	c1cccc(c12)sc(c2)C(=O)Nc(n3)scc3-c4ccccc4	20	9
Z87649109	C1CCCCN1C2(CCCCC2)CNC(=O)c3ccc(cc3)S(=O)(=O)NC4CC4	-3	5
Z87656555	c1cc(S(=O)(=O)N)ccc1C(C)NC(=O)c2ccc(cc2)-n(cn3)c(c34)cccc4	-5	2
Z87671607	s1cccc1C(=O)N(CC2)CCC2C(=O)NCC(N3CCCC3)c4c(Cl)cccc4	-35	9
Z88060567	C1CCCC1(C#N)NC(=O)COC(=O)c2ccc(cc2)-n(cn3)c(c34)cccc4	3	6
Z88237436	c1cccc(c1C#N)CSCCNC(=O)c(c2)ccc(c23)[nH]cn3	-1	5
Z88468962	C1CCCN1c(nccc2)c2C(=O)N3CCN(CC3)C(=O)C(O4)COc(c45)cccc5	-8	3
Z89008298	c1ccccc1NC(=O)c2ccc(cc2)CNC(C)c(c3)ccc(c34)OCCO4	-38	14
Z89065969	c1cccn(c12)cc(n2)CN3CCN(CC3)S(=O)(=O)c4c(Cl)cc(Br)cc4	-1	9
Z89137945	N#Cc1ccc(cc1)CN(CC2)CCN2Cc(n(c34)ccs4)c(n3)-c5ccccc5	-31	5
Z89154973	o1cccc1Cn(c(C)c(C)c2C#N)c2NC(=O)CN3CCN(CC3)Cc(n4)sc(c45)cccc5	30	22
Z89155296	c1cccn(c12)cc(n2)CN3CCN(CC3)S(=O)(=O)c4c(Cl)cccc4	15	7
Z89203975	s1cccc1CN(CC=C)CC(=O)Nc2ccc(cc2)S(=O)(=O)N(CC)CC	16	3
Z89207636	c1ccccc1-c(n2)c(n(c23)cccc3)CN4CCN(CC4)Cc5cc(OC)c(cc5)OC	45	13
Z89215884	C1COCCN1C(=O)CN2CCN(CC2)Cc([nH]c3=O)nc(c34)sc(C)c4-c(cc5)ccc5C	5	7
Z89215922	c1ccccc1-c2csc(c23)nc(n(c3=O)CC#N)CN(CC4)CCN4CC(=O)N5CCOCC5	-2	5
Z89320750	O=S1(=O)CC(CC1)N(C)S(=O)(=O)c(cc2)ccc2OC	2	5
Z89320796	Cc1c(C)ccc(c1)S(=O)(=O)N(C)C(CC2)CS2(=O)=O	2	4
Z89320874	O=S1(=O)CC(CC1)N(C)S(=O)(=O)c2cc(Cl)cc(Cl)c2	17	7
Z89320983	CCc1ccc(cc1)S(=O)(=O)N(C)C(CC2)CS2(=O)=O	10	26
Z89321005	c1cc(Br)cc(c1C)S(=O)(=O)N(C)C(CC2)CS2(=O)=O	0	2
Z89321012	c1cc(C)cc(c1C)S(=O)(=O)N(C)C(CC2)CS2(=O)=O	-3	2
Z89358343	O=S1(=O)CC(CC1)N(CC)S(=O)(=O)c(c2C)cc(Br)cc2	1	4
Z89460412	C1S(=O)(=O)CCC1(C)NC(=O)c(c2)ccc(c23)N(C(C)C3)S(=O)(=O)C	10	9
Z89489183	c1cccn1-c2oc(C)c(c2C#N)C(=O)Nc(ccc3)cc3/C=C/c4ccccn4	9	5
Z89513568	c1cc(Cl)ccc1SCCNC(=O)c2c(cccc2)OCc3c(C)noc3C	17	4
Z89582531	c1sccc1¥C=C¥C(=O)NC(c2cccs2)c3ccccc3	1	6
Z89622401	O=S1(=O)CC(CC1)N(CC2)CCN2C(=O)/C=C/c3c(C)n(c(C)c3)-c(c4)ccc(c45)OCO5	-12	10
Z89671452	Cc(s1)ccc1C(=O)OCc2nc(nc(n2)N)Nc3ccccc3	-2	3
Z89784395	c1ccccc1Cn(c2Cl)nc(C)c2C(=O)OCc3nc(nc(n3)N)Nc4ccccc4	0	8
Z89857426	c1cc(C)ccc1Cn(c2Cl)nc(C)c2C(=O)OCc3nc(nc(n3)N)Nc4ccccc4	22	11
Z90118492	s1cccc1-c(no2)nc2CSc(n3C4CC4)nnc3C5CC5	4	5
Z901305410	CC1CCN(CC1)c(ccc(c2)[N+]([O-])=O)c2C(=O)N3CCN(CC3)Cc(n4)cn(c45)cc(Cl)cc5	-17	14
Z90171664	CCn(nc1)c(c12)nc(cc2C(=O)OC)-c3ccccc3	11	5
Z90171904	COC(=O)c1cc(-c2cccs2)nc(c13)n(CC)nc3	20	10
Z90231169	c1ccc(Cl)cc1-c(no2)nc2CN3CCN(CC3)S(=O)(=O)c4c(Cl)cccc4Cl	-8	7

Z90240663	c1ccc(Cl)cc1-c(no2)nc2CN3CCN(CC3)S(=O)(=O)c4c(Cl)cccc4	-3	4
Z90289702	c1cc(F)ccc1-c(cc2C(=O)OC)nc(c23)n(C(CC4)CS4(=O)=O)nc3C	-1	3
Z90671680	Cc1ccc(cc1)-c(cc2C(=O)OC)nc(c23)n(C(C)C)nc3	26	15
Z90681964	c1c(Cl)ccc(c1C(F)(F)F)S(=O)(=O)N(CC2)CCN2Cc3cc(C)on3	13	11
Z90694917	FC(F)(F)c1ccc(cc1)S(=O)(=O)NCc(n2)n(C)c(c23)cccc3	-18	11
Z90703515	n1oc(C)cc1CSc(nccc2)c2C(=O)Nc3ccc(cc3)OCc4ccccc4	-10	8
Z908095506	N#CCCn(n1)c(cc1C)NC(=O)c2cc(on2)-c3cccs3	-3	4
Z908116464	C1CCc(c12)sc(c2)C(=O)Nc([nH]3)nnc3-c4ccccn4	-54	4
Z908597782	CC1CCC(CC1)N(C2CC2)C(=O)c3c(C)n(nc3)-c4ccc(=O)[nH]n4	-8	2
Z90896104	c1cccc(c12)OCC(=O)N2CCC(=O)Nc(n3)scc3-c4c(C)n(c(C)c4)CCC	-7	9
Z91026356	C1CCCCC1CCCC(=O)Nc(cc2)ccc2-c3c[nH]cn3	0	12
Z91037735	c1cccc(c12)oc(c2)C(C)NC(=O)CCc3c[nH]c(c34)cccc4	1	4
Z91043666	COc(cc1)c(OC)cc1-c(cc2C(=O)OC)nc(c23)n(C(CC4)CS4(=O)=O)nc3C	2	3
Z91043804	CC(C)n(nc1)c(c12)nc(cc2C(=O)OC)-c3c(F)cccc3	-6	1
Z910632940	Fc1cc(F)cc(O)c1C(=O)Nc(cc2)ccc2N(C)C3CCN(C)CC3	6	4
Z91135336	C1COCCN1Cc(cccc2)c2CNC(=O)CN(C(=O)N3)C(=O)C3(C)c(c4)ccc(c45)cccc5	3	12
Z91211893	Cc(c1)ccc(OC)c1CN(CC2)CCN2C(=O)CCc([nH]c3=O)nc(c34)n(nc4)-c5ccccc5	5	5
Z91218489	CN(C)S(=O)(=O)c(cc1)ccc1C(=O)NC(C(C)C)c(c2)ccc(c23)OCCCO3	5	6
Z913725738	C1CCCC1CC(=O)Nc(c(cc2)OC)cc2C(=O)N3CCCC3	7	8
Z914096084	Cc1onc(C)c1CSc(cccc2)c2C(=O)NCC3CCN(CC3)Cc4ccc(cc4)OC	-7	3
Z915389422	Nc1ncnc(c12)n(cn2)CCCSc(c3)ccc(c34)cccc4	18	4
Z915764402	C1CCCCN1C(=O)CN(CC2)CCN2C(=O)c(oc(c34)cccc4)c3CSc(n5)nccc5O	-15	6
Z915925636	n1ccccc1CC(=O)N2CCCN(CC2)c3nc(CN(CC4)CCC4C)nc(c35)sc(C)c5C	0	13
Z917537988	COc(c1)c(OC)cc(c1C(=O)N)NC(=O)Cc2c(C)[nH]c(c23)cccc3	-25	8
Z91879174	[nH]1cnnc1SCC(=O)NC(c2cccs2)c3ccc(C)cc3	-1	4
Z92475228	O1CCCC1Cn(c(C)c2)c(C)c2C(=O)CSc(n3-c(c4C)cccc4)nnc3N5CCOCC5	14	9
Z92479374	O1CCCC1Cn(c(C)c2)c(C)c2C(=O)CSc(n3-c(cc4C)ccc4)nnc3N5CCOCC5	3	4
Z92534919	c1cc(F)ccc1-c2csc(n2)NC(=O)CSc(n3C4CC4)nnc3-c5cccnc5	10	0
Z92593810	Cc1cc(no1)NC(=O)Cn(n2)c(C)c(c2C)S(=O)(=O)N3CCCCC3	-4	6
Z927306002	Clc1cccc(c1Cl)OC(C)C(=O)Nc(c2)ccc(c23)nc[nH]c3=O	-9	10
Z927972178	n1cncn1Cc(cc2)ccc2C(=O)Nc([nH]n3)cc3-c4c(OC)cccc4	13	4
Z929767438	FC(F)(F)c1ccnc(n1)Nc(cc2)cc(c23)sc(n3)C4CC4	-3	6
Z934680220	COc(nc1)ccc1N(C)C(=O)c2c(Br)c(n[nH]2)-c3ccccc3	-1	4
Z935140604	C1COCCN1CCOc(ccc2)cc2N3CCn4c3nc(c45)n(C)c(=O)n(C)c5=O	4	4
Z941223136	O1CCOc(c12)ccc(c2)C(C)(C)C(=O)Nc(ccc3)cc3-c4nccn4C	4	15
Z943285536	CCOc(cc1)c(OCC)cc1C(=O)Nc(c2)ccc(c23)CNC3=O	18	11

Z94561070	FC(F)(F)c1cc(ccc1)COC(=O)c2n[nH]c(=O)c(c23)cccc3	-11	13
Z94572736	N1C(=O)COc(c12)ccc(c2)C(=O)/C=C/c3ccc(cc3)OC(F)F	3	3
Z94609806	c1cccc(c1C(F)(F)F)S(=O)(=O)N(CC2)CCN2Cc3c(C)nn(c3Cl)-c4ccccc4	3	3
Z94739043	c1cnccc1CNC(=O)c2c(C(F)(F)F)n(nc2)-c3ccc(Cl)cc3	6	9
Z94751514	c1cccc(c12)oc(c2)-c(nc(c34)n(C(C)C)nc3)cc4-c5ccc(cc5)OC	3	5
Z95045593	c1cccc(c12)NC(=O)C¥2=C¥c3cnn(c3)-c4ccccc4	32	13
Z95046696	c1cc(Cl)cc(c12)NC(=O)C¥2=C¥c3cnn(c3)-c4ccccc4	4	6
Z951371090	O=c1[nH]ncc(c1Cl)NCc2ccc(cc2)Cn(cn3)c(c34)cccc4	5	22
Z95207137	FC(F)(F)c1cc(ccc1)/C=C(¥C(=O)O)Sc(o2)nnc2-c3ccco3	-11	12
Z95254533	c1cc(F)c(F)cc1¥C=C(Cl)¥c(n2)n(CCC)c(c23)ccc(c3)S(=O)(=O)N4CCOCC4	14	1
Z954394146	o1nc(C)nc1C(C)NC(=O)C2CSCN2C(=O)c3ccccc3	-12	10
Z954497746	C1CC1c(c2C)n[nH]c2NC(=O)C(CC3=O)CN3c(c4)ccc(c45)CCC5	-9	4
Z955363064	c1ccccc1-n(c2)nc(-c3ccc(cc3)OC)c2C(=O)NCc4cc(ncc4)N(CC5)CCN5CC	-5	3
Z956276516	C1OCc(c12)ccc(c2)C(=O)Nc([nH]n3)cc3-c4ccc(s4)Br	68	10
Z959760312	c1ccnc(S(=O)(=O)C(F)(F)F)c1C(=O)N(C)c(ccc2)cc2C(C)C	32	8
Z95982589	O=C1CCCN1c(ccc2)cc2NC(=O)c3c(-c4cccnc4)nn(c3)Cc5ccccc5	10	4
Z96018682	NC(=O)NCc(cc1)ccc1C(=O)NC(C(C)C)c2ccccc2	-1	5
Z96031302	c1ccccc1NC(=O)C2CCN(CC2)C(=O)C3CCOCC3	1	15
Z96041573	c1ccccc1-n(c2)nc(-c3cccnc3)c2C(=O)NCCCN(CC)c4ccccc4	-8	10
Z960870544	c1ccccc1-c(nc2)ncc2C(=O)NCCc3c[nH]c(c34)cc(F)cc4	43	14
Z961847812	c1cc(F)ccc1-n(nn2)c(C)c2C(=O)Nc(c3)ccc(c34)n(cc4)CCN5CCOCC5	-11	8
Z961849222	Cc1cc(c(cc1)OC)N2CC(CC2=O)C(=O)Nc(c3)ccc(c34)n(cc4)CCN5CCOCC5	23	14
Z96342510	c1cccc(c1C)Nc2nnc(s2)SCC(=O)N3CCCC3c4c(OC)ccc(c4)OC	9	4
Z96748519	s1cccc1-c(no2)nc2CCCSc(n3C)nnc3C4CC4	-7	5
Z968524204	CCOC(=O)Cn(cc1)c(c12)ccc(c2)NC(=O)c3c(C)nc(s3)-c4ccccc4	2	6
Z968739862	O=S1(=O)CCC(CC1)S(=O)(=O)NC2CCCCCC2	50	3
Z968740596	c1cccc(C)c1CN(C)S(=O)(=O)C(CC2)CCS2(=O)=O	-2	6
Z968814168	NC(=O)C1CCCN1C2CCN(CC2)C(=O)c3ccc(cc3)-n4nccc4C	-8	8
Z969651240	n1ccnc(c12)ccc(c2)/C=N/Nc(nc(c34)cccc3)c(n4)-c5ccccc5	11	4
Z970272914	O1CCN(C1=O)c(cc2)ccc2C(=O)/C=C/c3cccnc3	16	20
Z97092537	c1cccc(c12)OCC(O2)CN(C)C(=O)COc(cc3)cc(c34)oc(=O)cc4CCC	30	8
Z97289933	CC(C)CCCC(C)NC(=O)CSc(nn1)n(c12)c3c(cccc3)c(=O)n2-c4cc(OC)ccc4	19	17
Z97290306	CC(C)CCCC(C)NC(=O)CSc(nn1)n(c12)c3c(cccc3)c(=O)n2-c4ccc(cc4)OC	-3	2
Z975048678	N#Cc1ccc(cc1)CN(CC2)CCC2C(=O)NC(C)C(C)C	5	5
Z97565837	O1CCOc(c12)ccc(c2)CN(CC)C(=O)CSc(nnc3C(C)N(C)C)n3-c4ccc(F)cc4	15	7
Z978895402	c1cnc(N(C)C)cc1C(=O)Nc(c2C)ccc(c2)CN3CCSCC3	0	2

Z979061162	o1cccc1CN(CCC#N)C(=O)c2ccc(cc2)NC(=O)NC(C)C	7	7
Z982182198	c1cccc(c12)n(C)c(n2)CSc([nH]3)nnc3-c4c(F)cccc4	4	5
Z98268667	O1CCCOc(c12)ccc(c2)C(=O)CSc(n3C4CC4)nnc3-c5cccnc5	5	5
Z983418922	CS(=O)(=O)Cc(cc1)ccc1CNc2ccnc(c23)c(F)ccc3F	-17	9
Z98361471	COc(cc1)c(OC)cc1C(=O)N[C@@H]([C@@H]2c(cc3C(F)(F)F)ccc3)C(=O)Nc(c24)n(C)nc4C	17	6
Z98369603	O1CCCC1Cn(c(C)c2)c(C)c2C(=O)CSc(n3-c(cc4)ccc4C)nnc3N5CCOCC5	22	5
Z98370329	CC(C)(C)C(=O)Nc(cc1)ccc1C(=O)CSc(n2-c(cc3)ccc3C)nnc2N4CCOCC4	11	8
Z98638708	c1ncccc1-c(cc2C(=O)O)nc(c23)n(nc3)Cc4cccnc4	13	20
Z98638790	c1ncccc1-c(cc2C(=O)OC)nc(c23)n(nc3)C(C)C4CC4	12	7
Z98638831	o1cccc1-c(cc2C(=O)O)nc(c23)n(CCO)nc3C	-9	4
Z987964770	C1CSCCC1NC(=O)Nc2cc(ccc2)OCCn3cccn3	8	2
Z98941608	c1c(Cl)ccc(c12)oc(c2)C(=O)CSc(nnc3C)n3C4CC4	10	9
Z99004386	Cc1csc(n1)-c2ccc(cc2)OCC(=O)Nc(cc3)ccc3C(=O)Nc4c(OC)cccc4	5	5
Z990436502	Cc1cccc(n1)NCC2CCN(C2)c3ncnc(c34)n(C)nc4	6	8
Z990550860	NC(=O)c1cc(ccc1)CN(C2)CCC2CNc(cc3C)ncc3	-3	5
Z990551490	NC(=O)c1cc(ccc1)CN(C2)CCC2CNc(n3)cccc3C	2	6
Z990838348	N#CCCn(nc1)c(c12)ncnc2NCc(c3)ccc(c34)OCCO4	2	3
Z990848218	c1cccn(c12)c(nn2)CCNc3ncnc(c34)n(nc4)CCO	-6	5
Z990852254	N#CCCn(nc1)c(c12)ncnc2N(CC3)CCN3Cc4ccco4	3	11
Z990855820	N#CCCn(nc1)c(c12)ncnc2N(CCO3)CC3C	4	11
Z990858522	N#CCCn(nc1)c(c12)ncnc2NC(CC3C(F)(F)F)CCC3	-12	4
Z990868196	N#CCCn(nc1)c(c12)ncnc2NC3CCN(CC3)C(=O)C(C)C	-8	11
Z990883644	n1cccn1CC(C)CNc2ncnc(c23)n(nc3)CCO	11	6
Z991774294	c1cc(Br)ccc1-c(n[nH]2)cc2C(=O)NCCc3coc(n3)-c4ccccc4	-8	10
Z991809296	c1ccccc1-c(cc2)ccc2C(=O)NCc3nc(n[nH]3)-c4ccccc4	-14	7
Z991836758	FC(F)Oc(cc1)ccc1CC(=O)N(CC2)CCC2c([nH]n3)nc3-c4ccccc4	-3	4
Z991917352	c1ccc(C)c(c1C(C)C)NC(=O)CN2CCCCC2Cc3ccccc3	-4	11
Z992207680	CCN1CCN(CC1)Cc2ccc(cc2)NC(=O)C3CCCN3C(=O)c(c4O)ccc(c45)cccc5	51	18
Z99468124	c1sccc1-c(n2)scc2CC(=O)Nc(cc3)cc(c34)sc(n4)N5CCOCC5	8	7
Z99552008	Nc(n1)nc(N)nc1CSCc2ccc(cc2)-c3ccccc3	-6	7
Z99558919	CSc(cc1)ccc1CSCc2nc(nc(n2)N)Nc3ccccc3	-15	2
Z996487836	c1cccc(c12)N([C@@H](C2)C(=O)N)C(=O)c(c3)sc(c34)n(CC(C)C)nc4C	1	3
Z998371530	c1cc(F)ccc1C2CN(CCO2)C(=O)c3cccn3Cc4ccncc4	13	5
Z999825438	c1cccc(c12)c(=O)n(C(C)C)nc2C(=O)N3CCCN(CC3)C(=O)Nc4cc(Cl)ccc4	-3	3

a: Absolute configuration in stereochemistry is not determined. *b*: IR: Inhibition rate, SD: standard deviation (n = 4).

Table S4. Inhibition rate of compounds assayed in secondary screening

Compound ID	canonical SMILES [a]	Screening % [b]			
		First		Second	
		IR	SD	IR	SD
Z49895016	O=C(O)c1ccc(cc1)/C=N/Nc2ncnc(c23)sc(c3)-c4ccccc4	85	9	86	4
Z410927360	c1ccccc1-c(n(c23)CCCCC3)nc2C(=O)Nc(o4)nnc4-c5ccco5	87	2	80	9
Z64663944	C1CCCN1CCN2CCN(CC2)c(ncn3)c(c34)sc(c4)-c5ccc(F)cc5	84	2	77	5
Z64663950	C1CCCN1CCN2CCN(CC2)c(ncn3)c(c34)sc(c4)-c5ccc(Cl)cc5	89	3	76	2
Z1229984790	C1CCCC1c2cc(n[nH]2)NC(=O)c3ccc(cc3)Cn4ccnc4	81	2	74	3
Z57745304	CCOc(cc1)ccc1/C=N/Nc2ncnc(c23)[nH]cn3	78	11	73	2
Z275023406	c1cccc(c12)nc(cc2)SCC(=O)Nc(ns3)nc3-c4ccc(Cl)cc4	67	5	64	3
Z199512484	Fc1c(Cl)cc(cc1)Nc2c(C#N)cnc(c23)cc(c(c3)N)OCC	66	8	62	3
Z57745307	c1c(Br)ccc(OC)c1/C=N/Nc2ncnc(c23)[nH]cn3	62	3	61	4
Z57745314	CC(C)c(cc1)ccc1/C=N/Nc2ncnc(c23)[nH]cn3	67	7	61	1
Z295464022	c1cc(Cl)ccc1/C=N/Nc2ncnc(c23)[nH]cn3	65	1	60	5
Z50080378	c1cnccc1/C=N/Nc(ncn2)c(c23)sc(c3)-c4ccc(F)cc4	67	3	55	10
Z449737600	N1C(=O)COc(c12)ccc(c2)C(=O)/C=C/c3c(cccc3)OCc4cccnc4	39	2	51	9
Z1252403274	FC(F)Oc(c1)ccc(c12)nc(C)cc2N(CC3)Cc(c34)ncn4C5CC5	72	2	50	8
Z1283491630	N1CCN(C1=O)c(cc2)ccc2C(=O)Nc([nH]n3)cc3C(C)CCC	29	3	49	8
Z50080181	CC(=O)Nc(cc1)ccc1/C=N/Nc(ncn2)c(c23)sc(c3)-c4ccc(F)cc4	51	6	47	4
Z168829182	CC(=O)N1CCN(CC1)C(=O)c2c(OC)cc(C)c(c2)Sc3cnc(s3)NC(=O)c4ccc(C<br>Cl)cc4	64	4	37	4
Z56910454	N#Cc1c(N)sc(c12)C(C)(C)CC(=C2)¥C=C¥c3ccc(o3)C	31	5	36	6
Z48850058	NC(=O)c1c(O)ccc(c1)-c2csc(n2)NCc3ccccc3	39	8	28	9
Z107727738	c1ccnc(c12)sc(c2O)-c3nc(cccc4)c4c(c35)cccc5	43	3	24	7
Z49894862	COc(c1)c(OC)c(OC)cc1/C=N/Nc2ncnc(c23)sc(c3)-c4ccccc4	34	8	22	4
Z1262045135	CC(C)(C)Cc1cc([nH]n1)NC(=O)c2cnn(c2)-c3c(Cl)cccc3	70	4	21	8
Z237376112	C1COCCN1CCn(c2=O)c(=O)n(C)c(c23)nc4n3cc(-c5ccccc5)n4-c(cc6)ccc6<br>CC	39	3	20	7
Z432289626	n1cccn1Cc(cccc2)c2CNc3cncc(n3)-n4cccn4	41	4	18	4
Z1517268554	c1nn(C)c(C)c1/C=C¥2C(=O)Nc(c23)ccc(F)c3	31	3	16	3
Z48950829	c1ccccc1-c2c(C)c(-c3ccc(O)cc3)nc(c24)ccc(Cl)c4	33	4	14	3
Z871886494	c1ncccc1C(=O)/C=C/c(c2)cnc(c23)sc(c3C)C(=O)OC	66	4	12	5
Z1723428191	n1[nH]ccc1CNC(=O)c2cc(ccc2)-c(c3)cnc(c34)[nH]c(=O)n4C	29	6	11	3
Z203565878	CC(=O)c1cc([nH]c1)C(=O)Nc(s2)ncc2Cc3c(Cl)cccc3	68	9	10	10

Z133609650	O=S1(=O)CC(CC1)N(C)S(=O)(=O)Cc2ccccc2	55	8	9	9
Z1317605702	CC(C)(C)c1nc(ccn1)Nc2cn[nH]c2	42	9	7	10
Z46114735	c1cccc(c12)nc3c(c2C(=O)O)CN(C)CC¥3=C¥c(c4C)scc4	52	7	4	2
Z271096440	CS(=O)(=O)c1c(S(=O)(=O)Cl)cccc1	59	7	2	3
Z57038735	CCOc(cc1)ccc1-c(nc(c23)cccc3)c(Cl)c2-c4ccccc4	51	6	2	8
Z385234634	CC(C)n(nc1)c(c12)nc(-c3ccco3)cc2C(=O)NC(C)CCN4CCN(CC4)c5ccccc5	62	6	1	9
Z86400859	COc(cc1)ccc1CC(c2ccc(cc2)OC)Nc3ncnc(c34)scc4	58	23	1	13
Z1578863056	Cc1nccn1-c2cncc(n2)N(CC3)CCC34CCCC4	34	20	0	11
Z45649828	n1ccccc1¥C=C¥c2cc(ccc2)NS(=O)(=O)c(c3C)c(C)[nH]n3	36	3	0	7
Z48950772	c1ccccc1-c2c3COc(cccc4)c4c3nc(c25)ccc(Cl)c5	50	6	0	4
Z286209468	COC(=O)c1c(F)cc(F)c(c1)NS(=O)(=O)c2cc(Cl)cc(Cl)c2	60	8	-2	6
Z336032562	c1cccc(c12)nc3c(c2C(=O)O)CN(C)CC¥3=C¥c(c4)c(Cl)nc(c45)n(C(C)C)nc5	54	4	-2	5
Z48950817	c1c(Cl)ccc(c12)nc(-c3ccc(O)cc3)cc2-c4ccccc4	51	18	-2	4
Z232307154	CS(=O)(=O)NCCc1ccc(s1)C2=NN=C(SC2)Nc3cccc(C)c3C	37	24	-3	6
Z248485700	CS(=O)(=O)c1ccc(S(=O)(=O)Cl)cc1	73	5	-3	7
Z128154998	C1COCCN1Cc(nc(c23)sc(C)c2C)nc3N(CC4O)Cc(c45)c(OC)ccc5OC	47	10	-4	8
Z223838602	C1COCCN1CCn(c2=O)c(=O)n(C)c(c23)nc4n3CCN4c5ccc(cc5)Oc6ccccc6	49	3	-4	8
Z48025165	C1COCCN1CCNC(=O)C(¥C#N)=c2/n(Cc3ccco3)c(=O)/c(s2)=C¥c4ccc(cc4)N(C)C	27	2	-4	9
Z764721614	C1CC(=O)Nc(c12)ccc(c2)OC(=O)c3c(-c4cccs4)nn(c3)-c5ccccc5	63	5	-5	11
Z298640636	c1cccc(c1C#N)OCC(=O)N(CC2)CC=C2c3c[nH]c(c34)nccc4	50	19	-6	5
Z204342158	s1cccc1¥C=C¥C(=O)Nc(c2C(=O)O)cc(s2)-c3cc([N+]([O-])=O)ccc3	58	6	-7	5
Z48950811	c1c(Cl)ccc(c12)nc(-c(cc3)ccc3C)cc2-c4ccccc4	59	11	-7	4
Z139929606	n1ccccc1CNC(=O)c2cc(C3CC3)nc(c24)n(C(CC5)CS5(=O)=O)nc4C	49	20	-8	10
Z46035916	c1cccc(c12)nc3c(c2C(=O)O)CCCC/3=C¥c4cc(c(cc4)OC)OCC	51	8	-8	7
Z199538216	CS(=O)(=O)c(cc1)cc(S(=O)(=O)C)c1N2CCCCCC2	51	4	-10	9
Z56805970	C1CCCCN1c(cc2)c(cc2C(F)(F)F)NC(=O)c3c(cccc3)NS(=O)(=O)c(c(Cl)cc4)cc4C(=O)Nc(cc5C(F)(F)F)c(cc5)N6CCCCC6	44	2	-10	7
Z57237939	o1cccc1C(=O)C2=C(O)C(=O)N(C2c3cc([N+]([O-])=O)ccc3)CCCn4ccnc4	62	21	-11	4
Z826860216	Cc1nn(C)c(c12)nc(-c3ccccc3)cc2C(=O)Nc(cc4)cc(c45)[nH]c(n5)CN6CCOCC6	35	31	-11	12
Z729018028	c1[nH]ncc1Nc(n2)cnc(c23)cccc3	45	10	-12	8
Z48950819	c1c(Cl)ccc(c12)nc(-c3ccc(Cl)cc3)cc2-c4ccccc4	36	9	-13	5
Z1592246310	c1cccc(c12)CC(C)(OC2=O)C(=O)Nc(cncn3)c3-c4ccccc4	35	17	-14	5
Z990870858	N#CCCn(nc1)c(c12)ncnc2N3CCOCC3C	56	9	-15	6

Z1262455575	c1ccccc1S(=O)(=O)CCS(=O)(=O)N2CCCCCC2	44	17	-16	9
Z412775020	o1cccc1C(=O)Nc2ccc(cc2)NC(=O)NCCSCc3ccccc3	44	12	-16	10
Z49625943	COc(cc1)c(O)cc1/C=N/Nc2ccnc(c23)cc(Cl)cc3	26	6	-17	8
Z56867509	C1CCCCN1S(=O)(=O)c(cc2c3c45)cc3[nH]c(=O)c4cc(nc5cc2)-c6c(Cl)cccc6	26	14	-24	7
Z56872869	O1COc(c12)ccc(c2)CNC(=O)c3c(N)n(CCc4cc(OC)c(cc4)OC)c(c35)nc6c(n5)cccc6	34	14	-24	8
Z1462541066	NC(=O)c1cc(c(C)c(F)c1)NCc(cn2)n(c23)ccc(C)c3	33	31	-26	11
Z991036884	CN(C)C(=O)c1ccc(cc1)CNCc2c(C)nn(C)c2OC	27	3	-29	8

Compounds Z49895016 to Z50080181 passed the secondary screening. *a*: Absolute configuration in stereochemistry is not determined. *b*: IR: Inhibition rate, SD: standard deviation (n = 4).

Table S5. Most similar known inhibitor of hit compounds

Compound ID	Chemical Structure of similar compound [a]	Similarity [a]	ChEMBL ID of compound [a]	Reference [a]	Group Proposed (Filter class) [b]
Z64663950	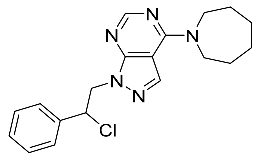	0.787	CHEMBL473154	4	3 (LB)
Z49895016	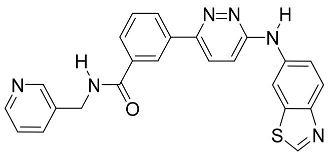	0.733	CHEMBL501182	5	3 (LB)
Z64663944	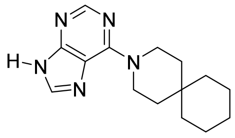	0.773	CHEMBL2420924	6	3 (LB)
Z1229984790	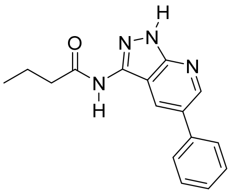	0.783	CHEMBL258701	7	10 (LB→SB)
Z57745314	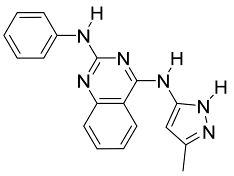	0.800	CHEMBL552038	8	3 (LB)
Z57745304	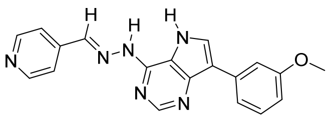	0.816	CHEMBL206010	GSK_PKIS	3 (LB)
Z199512484	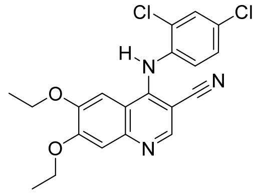	0.868	CHEMBL168319	9	3 (LB)
Z410927360	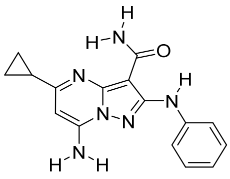	0.702	CHEMBL261450	10	10 (LB→SB)

Z2954640 22	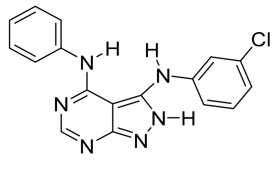	0.882	CHEMBL121260	11	3 (LB)
Z4497376 00	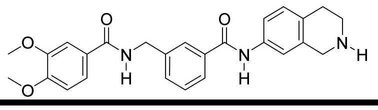	0.651	CHEMBL604087	12	11 (SB)

a: The most highest similarity of each hit compound to known inhibitors defined in Section "Preparation of compound library" is shown with its chemical structure, ChEMBL ID, and relevant literatures. GSK_PKIS is Published Kinase Inhibitor Set (PKIS) by GlaxoSmithKline. The similarity was calculated with the Tanimoto coefficient of the MACCS descriptor.[1]

b: A group ID that proposed each compound is shown with its filter class.

Table S6. Known ligand information that Group 3 used

PubChem AID	Target	Reference
613160	Itk	Bamborough, P., Brown, M. J., Christopher, J. A., Chung, C.-w., and Mellor, G. W. (2011). Selectivity of kinase inhibitor fragments. J Med Chem, 54(14):5131-5143.
651248	Fyn	Drewry, D. H., Willson, T. M., and Zuercher, W. J. (2014). Seeding collaborations to advance kinase science with the GSK published kinase inhibitor set (PKIS). Curr Top Med Chem, 14(3):340-342.
651249	Fyn	
651250	Hck	
651251	Hck	
651258	Itk	
651259	Itk	
651276	Lck	
651277	Lck	
651280	Lyn_A	
651281	Lyn_A	
651282	Lyn_B	
651283	Lyn_B	
651300	Frk	
651301	Frk	
651314	Src	
651315	Src	
651316	Srm	
651317	Srm	
651320	Tec	
651321	Tec	
651334	Txk	
651335	Txk	
651370	Abl_E255K	
651371	Abl_E255K	
651372	Abl_H396P	
651373	Abl_H396P	
651374	Abl_M351T	
651375	Abl_M351T	
651376	Abl_Q252H	
651377	Abl_Q252H	

651378	Abl_T315I	
651379	Abl_T315I	
651380	Abl_Y253F	
651381	Abl_Y253F	
651382	Abl	
651383	Abl	
651386	Arg	
651387	Arg	
651390	Blk	
651391	Blk	
651392	Bmx	
651393	Bmx	
651394	Brk	
651395	Brk	
651396	Btk	
651397	Btk	
651440	Fgr	
651441	Fgr	
651450	Yes	
651451	Yes	
686947	Yes	Patel, P. R., Sun, H., Li, S. Q., Shen, M., Khan, J., Thomas, C. J., and Davis, M. I. (2013). Identification of potent yes1 kinase inhibitors using a library screening approach. Bioorg Med Chem Lett, 23(15):4398-4403.
720031	Abl	Gao, Y., Davies, S. P., Augustin, M., Woodward, A., Patel, U. A., Kovelman, R., and Harvey, K. J. (2013). A broad activity screen in support of a chemogenomic map for kinase signalling research and drug discovery. Biochem J, 451(2):313-328.
720032	Abl	
720033	Arg	
720034	Arg	
720043	Brk	
720044	Brk	
720045	Btk	
720046	Btk	
720047	Blk	
720048	Blk	
720049	Bmx	
720050	Bmx	

720161	Fgr	
720162	Fgr	
720171	Fyn	
720172	Fyn	
720193	Hck	
720194	Hck	
720195	Hck	
720196	Hck	
720218	Frk	
720219	Frk	
720312	Itk	
720313	Itk	
720332	Lck	
720333	Lck	
720334	Lck	
720335	Lck	
720336	Lyn_A	
720337	Lyn_A	
720398	Tec	
720399	Tec	
720406	Txk	
720407	Txk	
720418	Yes	
720419	Yes	
720428	Src	
720429	Src	
745317	Abl	Cai, J., Sun, M., Wu, X., Chen, J., Wang, P., Zong, X., and Ji, M. (2013). Design and synthesis of novel 4-benzothiazole amino quinazolines dasatinib derivatives as potential anti-tumor agents. Eur J Med Chem, 63:702-712.
745318	Src	
775219	Abl	Xu, F., Zhang, L., Jia, Y., Wang, X., Li, X., Wen, Q., Zhang, Y., and Xu, W. (2013). Discovery of 4-amino-2-(thio)phenol derivatives as novel protein kinase and angiogenesis inhibitors for the treatment of cancer: synthesis and biological evaluation. part II. Eur J Med Chem, 69:191-200

References

1 Durant, J. L., Leland, B. A., Henry, D. R. & Nourse, J. G. Reoptimization of MDL keys for use in drug discovery. *J. Chem. Inf. Comput. Sci.* **42**, 1273-1280, doi:10.1021/ci010132r (2002).

2 Duan, J. X., Dixon, S. L., Lowrie, J. F. & Sherman, W. Analysis and comparison of 2D fingerprints: Insights into database screening performance using eight fingerprint methods. *J. Mol. Graph. Model.* **29**, 157-170, doi:10.1016/j.jmgm.2010.05.008 (2010).

3 Sastry, M., Lowrie, J. F., Dixon, S. L. & Sherman, W. Large-Scale Systematic Analysis of 2D Fingerprint Methods and Parameters to Improve Virtual Screening Enrichments. *J. Chem. Inf. Model.* **50**, 771-784, doi:10.1021/ci100062n (2010).

4 Tintori, C., Magnani, M., Schenone, S. & Botta, M. Docking, 3D-QSAR studies and in silico ADME prediction on c-Src tyrosine kinase inhibitors. *Eur. J. Med. Chem.* **44**, 990-1000, doi:10.1016/j.ejmech.2008.07.002 (2009).

5 Gozalbes, R. *et al.* Development and experimental validation of a docking strategy for the generation of kinase-targeted libraries. *J. Med. Chem.* **51**, 3124-3132, doi:10.1021/jm701367r (2008).

6 Allen, C. E. *et al.* Synthesis and evaluation of heteroaryl substituted diazaspirocycles as scaffolds to probe the ATP-binding site of protein kinases. *Bioorg. Med. Chem.* **21**, 5707-5724, doi:10.1016/j.bmc.2013.07.021 (2013).

7 Witherington, J. *et al.* 5-Aryl-pyrazolo[3,4-b]pyridines: potent inhibitors of glycogen synthase kinase-3 (GSK-3). *Bioorg. Med. Chem. Lett.* **13**, 1577-1580, doi:10.1016/s0960-894x(03)00134-3 (2003).

8 Bebbington, D. *et al.* The discovery of the potent aurora inhibitor MK-0457 (VX-680). *Bioorg. Med. Chem. Lett.* **19**, 3586-3592, doi:10.1016/j.bmcl.2009.04.136 (2009).

9 Boschelli, D. H. *et al.* Synthesis and Src kinase inhibitory activity of a series of 4-phenylamino-3-quinolinecarbonitriles. *J. Med. Chem.* **44**, 822-833 (2001).

10 Mukaiyama, H. *et al.* Novel pyrazolo[1,5-a]pyrimidines as c-Src kinase inhibitors that reduce IKr channel blockade. *Bioorg. Med. Chem.* **16**, 909-921, doi:10.1016/j.bmc.2007.10.068 (2008).

11 Traxler, P. *et al.* Use of a pharmacophore model for the design of EGF-R tyrosine kinase inhibitors: 4-(Phenylamino)pyrazolo[3,4-d]pyrimidines. *J. Med. Chem.* **40**, 3601-3616, doi:10.1021/jm970124v (1997).

12 Morwick, T. *et al.* Hit to lead account of the discovery of bisbenzamide and related ureidobenzamide inhibitors of Rho kinase. *J. Med. Chem.* **53**, 759-777, doi:10.1021/jm9014263 (2010).

Methods used by each group

Group 1

In this virtual screening campaign, we adopted the following strategy.

(1) First, we train a structure activity relationship (SAR) model by utilizing high throughput screening (HTS) data for small molecule inhibitor.

(2) Next, a trained model is applied to the compounds of Enamine library to predict the compound activity.

Based on HTS data for small molecule inhibitors deposited in PubChen database [1], we defined seven Yes kinase inhibitors that are active when their activity concentration is less than 1 nM as active compounds, and the others as inactive compounds. This criterion for the active compounds was placed to find compounds that show strong inhibition. We downloaded the SDF files of compounds deposited in PubChem database and Enamine library compounds that is a target library in this virtual screening campaign. Then, we generated 1444 1D and 2D descriptors through PaDEL descriptor for compounds [2].

Since class distribution of a data set is highly skewed, a typical learning algorithm tends to show a strong bias toward the majority class [3]. To address this problem, we employ Balanced Random Forests (BRF) to learn a SAR model [4-6]. Random Forests is a well-known supervised learning algorithm for its excellent generalization performance in QSAR modeling [4]. In BRF, we down-sample the majority class with replacement and grows each tree on a more balanced data set. A majority vote is taken as usual for prediction. This procedure boosts the minority class. We set the sampling size of each class to the number of active compounds that is the minority class, the number of tree to 105. Finally, we picked up the most promising 400 compounds according to the active probability obtained through BRF.

References

[1] https://pubchem.ncbi.nlm.nih.gov/assay/assay.cgi?aid=686947

[2] Yap CW, “PaDEL-Descriptor: An open source software to calculate molecular descriptors and fingerprints.” J. Comput. Chem., 32(7):1466-74, 2011

[3] Hido S et al., “Roughly balanced bagging for imbalanced data” Statistical Analysis and Data Mining, 2(5-6): 412-426, 2009

[4] Breiman L, “Random Forests” Machine Learning., 45(1):5-32, 2001

[5] Svetnk V et al., “Random forest: a classification and regression tool for compound classification and QSAR modeling.” J. Chem. Inf. Comput. Sci., 43(6):1947-58, 2003

[6] Chen C, “Using random forest to learn imbalanced data.” (http://statistics.berkeley.edu/sites/default/files/tech-reports/666.pdf)

Group 2

1) Build model and prediction

To build model, I used the data-set that is provided from PubChem AID_686947.
Randomly-chosen 80% of the data was used for training and the remaining was used for validation.
At first, Morgan Fingerprint (2048 bit) was calculated with RDKit [1], and built classification (Active/non active) model using a python tool kit for deep learning named PDNN [2]. I chose Deep Neural Network as model. Learning setting was following.
Size of batch; 16.
Number of hidden layers; 2
Activation function; hyperbolic tangent.

Finally, I selected compounds that has over 0.9 probability of active.
40K compounds were selected.

2) Selection of 400 compounds form 40K compounds.

To filter the selected compounds, I used some drug like filters.
1st step, these compounds were filtered using Lilly's Med Chem Filter[3].
Lilly-Medchem-Rules is structural filter and the cord is freely available from Git-Hub.

Then I selected molecules that have following properties and I got 10K compounds.
Number of aromatic rings; less than 6.
Molecular weight; 350 <= MolWt <= 600.
Hydrogen bond donner <= 5

Finally, Diversity focused selection using RDKit function named SimDivFilters function.
And ranked each compound by Drug likeness (QED score).

References

[1] http://www.rdkit.org/
[2] https://github.com/yajiemiao/pdnn
[3] https://github.com/IanAWatson/Lilly-Medchem-Rules

Group 3

1. Filtration of druglike compounds

Druglikeness scores of all compounds in the library were estimated with a modified QED[1]. The score was calculated based on similarity with physicochemical properties of known Src inhibitors provided whereas the original QED score was based on that of approved orally administered drugs. A dataset of the known Src inhibitors was obtained from the website of the contest[2].

Only compounds whose score values were equal or higher than 0.39 proceeded to the next step described below. As a result, one million of compounds were excluded from the inhibitor candidate to be submitted.

2. Prediction of inhibition activity with consideration of experimental conditions

Assay information of Yes and its related kinases was collected from PubChem Assay. The assay result of the contest in 2014 was used as well. Then Morgan2[3] and atom pairs fingerprint[4] were calculated for each ligand. Similarly ProtFP (Feature)[5] and Z-scales (3)[6] fingerprint were calculated for each target kinase (residue 273 to 420 in numbering of Yes). Descriptors reflecting experimental conditions were prepared as follows: concentrations of each solute (i.e. ligand, ATP, and Mg), pH of solution, flag of activation of Lck kinase, and dummy variables to identify an article in which the assay was described.

These three types of fingerprint/descriptor, i.e. the fingerprints for ligands, the descriptors for proteins, and the descriptor for experimental conditions were concatenated into a series of feature vectors. Missing values in the vectors were imputed with average values. Regression model of extremely randomized trees algorism[6] was trained by the vectors. Finally, inhibition activities of compounds which passed the filter above were predicted with the model.

3. Ordering compounds to be submitted

Compounds were sorted by the predicted activity to create a ranking of raw activity. Then, the compounds were re-sorted by a predicted ligand efficiency, which is defined as (predicted activity) / (number of heavy atom), to create a ranking of ligand efficiency. Additionally the top 1000 compounds in the ranking of raw activity were re-sorted by maximum of Tanimoto coefficients against known Src-inhibitors in ascending order to create a ranking of novelty. At the end, 400 compounds to be submitted were extracted from the top of these three rankings in rotation, avoiding duplication.

References

[1] Bickerton, G. R., Paolini, G. V., Besnard, J., Muresan, S. & Hopkins, A. L. Quantifying the chemical beauty of drugs. *Nature Chemistry* **4**, 90-98 (2012).

[2] http://www.cb.cs.titech.ac.jp/ipab/2nd_contest/Eliminated.sdf.zip

[3] Rogers, D. & Hahn, M. Extended-Connectivity fingerprints. *J. Chem. Inf. Model.* **50**, 742-754 (2010).

[4] Carhart, R. E., Smith, D. H. & Venkataraghavan, R. Atom pairs as molecular features in structure-activity studies: definition and applications. *J. Chem. Inf. Comput. Sci.* **25**, 64-73 (1985).

[5] van Westen, G. J. P. *et al.* Which compound to select in lead optimization? prospectively validated proteochemometric models guide preclinical development. *PLoS ONE* **6**, e27518+ (2011).

[6] Sandberg, M., Eriksson, L., Jonsson, J., Sjöström, M. & Wold, S. New chemical descriptors relevant for the design of biologically active peptides. a multivariate characterization of 87 amino acids. *J. Med. Chem.* **41**, 2481-2491 (1998).

[7] Geurts, P., Ernst, D. & Wehenkel, L. Extremely randomized trees. *Machine Learning* **63**, 3-42 (2006).

Group 4

We have been developing a profile based drug discovery *in silico* software named chooseLD. In this occasion, we tried to evaluate the performance of chooseLD by comparing its outcomes with the experimental validations.

Briefly, the outline of ligand discovery employed in this study was as follows;

1. The tertiary protein structure prediction based on FAMS

2. The screening of template ligands

3. Filtering template ligands from excluded potential inhibitors based on binding structure obtained using template ligands identified in the 2nd step.

4. ChooseLD based scoring (FPAScore) of candidate compounds using template ligands identified in the 3rd step and the generation of lists of compounds to be submitted

In the following, more detailed instructions will be given.

1. The tertiary Protein structure prediction based on FAMS

FAMS [1] is the profile based tertiary protein stricture inference software developed in Umeyama Lab at Kitazato University. At first, using this program, we have predicted the tertiary structure of the target protein. FAMS identifies reference protein structure using BLAST search of PDB. In this study, using NP_005424.1 as the target amino acid sequence, 1Y57 (Proto-oncogene tyrosine-protein kinase Src) was selected as the reference PDB protein structure for the inference. Although this PDB structure did not include the structure corresponding to the whole protein, since it included the structure corresponding to the supposed ligand docking regions and was associated with the smallest (thus, the most feasible) P-values, 1Y57 was selected.

２． The screening of template ligands

Next, based on BLAST search of PDB, we sought PDB protein structures whose amino acid sequences were highly homologous to NP_005424.1 and binding ligands were as many as possible. Then, identified ligands were re-mapped to the inferred tertiary structure of NP_005424.1.

The list of identified 25 ligands were

KSW_NBS 2HWO_RBS_00 3EN7_ABJ 1QCF_PP1 2QQ7_SR2_00 3F6X_IHH_01 1Y57_MPZ 2SRC_ANP 3GEQ_PP2_01 1YOL_S03_00 3D7T_STU_00 3LOK_DJK_00 1YOM_P01_01 3DQW_SAP_00 3QLG_1N1_01 2BDF_24A_01_n12 3DQX_AMP_01 3SVV_VSP_00 2BDJ_HET 3EN4_KS1_01 3UQG_B5A_00 2H8H_H8H 3EN5_KS4_01 2HCK_QUE_00 3EN6_KS5

Here the first four letters represent PDB IDs and numbers/characters following them represent ligands.

5. Filtering template ligands from excluded potential inhibitors based on binding structure obtained using template ligands identified in the 2nd step.

In the third step, in order to obtain more feasible template ligands, binding structures of compounds excluded by contest organizers as potential inhibitors were inferred by ChooseLD[2]. Among those compound binding structures, the set of seeming most promising template ligands were identified by 3-dimentional non-redundantly positioning using own criterion.

6. ChooseLD based scoring (FPAScore) of candidate compounds and the generation of lists of compounds to be submitted

Finally, FPAScore[2] were attributed to all of candidate compounds included in library using the inferred tertiary structure of NP_005424.1 and the template ligands identified in the 3rd step. FPAScore was computed based upon the alignments between the template ligands and the randomly generated structures of candidate compounds with considering the interaction between atoms. FRAScore was also supposed to have larger values if the candidate compounds had the similar binding modes to the known inhibitors (the template ligands). Based on the FPAScores associated with all candidate compounds, the top ranked 400 compounds were submitted.

References

[1] Umeyama H, Iwadate M. FAMS and FAMSBASE for protein structure, Curr Protoc Bioinformatics. 2004 Feb; Chapter 5:Unit5.2. doi: 10.1002/0471250953.bi0502s04.

[2] Takaya D, Takeda-Shitaka M, Terashi G, Kanou K, Iwadate M, Umeyama H, Bioinformatics based Ligand-Docking and in-silico screening. Chem Pharm Bull (Tokyo). 2008 May; 56(5):742-4.

Group 5

Computational Methods

Structure of Yes kinase was predicted using homology modeling. Src family kinases are known to have two distinct conformations such as DFG-IN and DFG-OUT. It is designated by conformation of the activation loop which begins with a conserved DFG (Asp-Phe-Gly) motif. Choosing the conformation is crucial in searching inhibitors as the kinase inhibitors are classified into two types (Type I and Type II) based on their binding preference to either of these two conformations. Type I inhibitors are ATP-competitive and prefer DFG-IN whereas the type II inhibitors prefer DFG-OUT conformation and do not compete for ATP binding site.

Structure of Yes kinase

The crystal structures of c-Abl kinase is available for both IN and OUT conformations (1IEP and 1OPK, respectively)[1, 2] were taken as templates and respective model was generated using Modeller software [3]. Each model was prepared by assigning bond order and charges. The appropriate protonation state of each amino acid was also ensured. The optimization of each structure includes prediction and restoration of hydrogen bond network, His tautomers and ionization states, and assigning 180° rotations of the terminal angle of Asn, Gln, and His residues. To alleviate steric clashes exist in the initial models, an all-atom constrained minimization was carried out with the Impact Refinement module (Impref). RMSD reached a maximum cutoff of 0.30 Å. To perform an independent docking and screening using IN and OUT conformations, the grid box was set at the respective site based on the ligand. PD166326, type I inhibitor (1IEP) and imatinib, type II inhibitor (1OPK) which were co-crystallized with c-Abl kinase were docked with respective IN and OUT models generated for Yes kinase. These complexes were then subjected for further screening by setting the grid box based on the ligand. The default inner box size of 10Å^3 was set whereas the outer box size was set based on the size of the PD166326 for DFG-IN and imatinib for DFG-OUT conformations.

Compound library for screening

Enamine library of 2.2 million compounds was employed to perform pharmacophore based and structure based virtual screening. The 2D to 3D conversion was performed using Ligprep program to generate number of possible states (tautomers, stereoisomers), ionization at a selected pH range (7±2), and ring conformations (1 ring conformer). Energy minimization of the 3D conformers was performed with the OPLS_2005 force field. A maximum of 32 stereoisomers and tautomers were generated for each ligand, where stereoisomers for specified chiralities were retained. Finally, a conformation with low energy per ligand was generated. The data set of about 26 Yes kinase specific inhibitors and 574 decoys (reported by iPAB2014 contest) were used to validate the screening. The

physicochemical properties were calculated using the Qikprop module. Based on these physicochemical properties we have filtered the initial compound library, which yielded 1040559 compounds. In addition, we have included 26 actives and 574 decoys and subjected a total of 1041159 compounds for High Throughput Virtual Screening (HTVS) using Glide software package [4] of Schrodinger Suite. The top hits were selected based on the glide scoring scheme which employs the following relation.

$$GlideScore = \begin{array}{c} 0.065E_{coul} + 0.130E_{vdW} + E_{Lipo} + E_{HBond} + \\ E_{Metal} + P_{BuryP} + P_{RotB} + Site \end{array}$$

Where, P_{BuryP} is penalty term for burying polar functionality in a hydrophobic environment, P_{RotB} is penalty term for freezing rotatable bonds and the "Site" term rewards polar, but non-hydrogen bonding interactions in the site.

Pharmacophore modeling and screening

The descriptor features representing the binding mode of the type I and type II inhibitors are calculated using glide XP docking protocol and e-pharmacophore module. E-pharmacophore module [5, 6] computes the features by energy based optimization of the ligand binding at the active site. The Glide XP scoring terms is used to choose the pose and maps the energies onto atoms. These energies are then used to score the sites and the final pharmacophore model is generated. The type I and type II specific pharmacophore models were then verified to ensure the complementarity to have the features that are essential for interacting with the DFG-IN and OUT pockets, respectively. The resultant compounds from the DFG-IN and DFG-OUT structure based virtual screening are subjected for DFG-IN and DFG-OUT pharmacophore based virtual screening (PBVS), respectively. The same set of actives and decoys were included to validate the screening results. Finally, the top hit compounds from the PBVS based on DFG-IN and DFG-OUT conformations were prepared and submitted for further experimental validation. Most similar compounds were removed from the top hits using clustering by structure based finger prints.

References

[1] Nagar, B., et al., *Structural basis for the autoinhibition of c-Abl tyrosine kinase.* Cell, 2003. **112**(6): p. 859-71.

[2] Nagar, B., et al., *Crystal structures of the kinase domain of c-Abl in complex with the small molecule inhibitors PD173955 and imatinib (STI-571).* Cancer Res, 2002. **62**(15): p. 4236-43.

[3] Sali, A. and T.L. Blundell, *Comparative protein modelling by satisfaction of spatial restraints.* J Mol Biol, 1993. **234**(3): p. 779-815.

[4] Friesner, R.A., et al., *Extra precision glide: docking and scoring incorporating a model of*

hydrophobic enclosure for protein-ligand complexes. J Med Chem, 2006. **49**(21): p. 6177-96.

[5] Salam, N.K., R. Nuti, and W. Sherman, *Novel method for generating structure-based pharmacophores using energetic analysis.* J Chem Inf Model, 2009. **49**(10): p. 2356-68.

[6] Loving, K., N.K. Salam, and W. Sherman, *Energetic analysis of fragment docking and application to structure-based pharmacophore hypothesis generation.* J Comput Aided Mol Des, 2009. **23**(8): p. 541-54.

Group 6

In order to identify novel inhibitors of Yes kinase in the IPAB2 contest, we used a virtual screening method called VS-APPLE[3], which we have been developing. One of the notable features of VS-APPLE is that it combines structure based virtual screening and ligand based virtual screening approach. Briefly, the method performs 3D structural comparison of a multiple-ligand template, which consists of known multiple inhibitors, against the database compounds using a geometric hashing technique. If a steric clash between a compound and the target protein is found, a score for a given ligand pose is penalized. The detailed description of the method is as follows.

Preparation of the target protein structure.

Based on a BLAST search of PDB, the closest homolog of Yes (pdb id: 2src) was identified. Using this template, the 3D structure of the target protein was obtained via homology modeling using Modeller[1].

Preparation of the multiple ligand template.

The template ligand we used here was the multiple ligand template, which was derived from complex structures of the homologs of the target and their ligands. To collect the complex structures of ligands and the proteins, the Protein Data Bank was searched for homologous of the target, which are collected into a library of protein–ligand complexes. This search was performed by the structure comparison method MICAN[4,5]. Structural similarity was evaluated by TM-score, by which homologous structures are collected if they satisfy the threshold similarity to the target structure (TM-score $\geq$0.6). As a result, about 2000 ligand-protein complex structures were obtained. From these 2000 ligands, we selected 20 ligands as components of the multiple ligand template, based on the ability to discriminate known actives from decoys. To assess this ability for the 2000 ligands, we performed virtual screening tests for each ligand using known actives and decoys for each ligand. Here we used known kinase inhibitors distributed by IPAB (Eliminated.sdf.zip) as actives and compounds defined as decoys of SRC in DUD benchmark set[2], and we used single template mode of VS-APPLE, which is equivalent to the algorithm LigMatch[8], to rank the actives and the decoys. The selected 20 proteins and their bound ligands were superimposed by the protein structure alignment program MICAN[4,5] against the target protein structure model. We used this complex structure, which consists of the 20 superimposed ligands and the target protein structure, as "the multiple ligand template".

Conformer Generation. For each of the compound of 2.4 million Enamine collection, 3D conformers were generated using Openeye's OMEGA[7] with the following parameters:
RMSD window=1.0Å, Energy window=25.0 kcal/mol, Max conformers=100.

Scoring Process.

To rank the 2.4 million compounds, we compared all conformers of all the compounds with the multiple ligand template and computed the 3D structure similarity score for all the compounds. For each of the conformer of a given compound, by performing all possible rigid transformations using the geometric hashing technique, we identify the rigid transformation that maximizes the score, which is defined as the number of coincident atoms minus the protein-ligand crash penalty. Here, the protein-ligand crash penalty is defined by the number of steric crashes between the query compound and the target protein atoms. The highest score is recorded for a given conformer of a given compound. The score for a given compound is calculated by averaging the highest scores of all conformers of the compound. By repeating the same procedure for all the library compounds, we scored and ranked all the compounds. The top 400 ranked compounds were selected as potential inhibitor of Yes kinase and submitted.

References

[1] Fiser, A. & Sali, A. Modeller: generation and refinement of homology-based protein structure models. Methods Enzymol 374, 461–491, doi: 10.1016/S0076-6879(03)74020-8 (2003).

[2] von Korff, M., Freyss, J. & Sander, T. Comparison of ligand- and structure-based virtual screening on the DUD data set. J Chem Inf Model 49, 209–231, doi: 10.1021/ci800303k (2009).

[3] Okuno, T., Kato, K., Terada, T. P., Sasai, M. & Chikenji, G. VS-APPLE: A Virtual Screening Algorithm Using Promiscuous Protein-Ligand Complexes. J Chem Inf Model 55, 1108–1119, doi: 10.1021/acs.jcim.5b00134 (2015)

[4] Minami, S., Sawada, K. & Chikenji, G. MICAN: a protein structure alignment algorithm that can handle Multiple-chains, Inverse alignments, C(alpha) only models, Alternative alignments, and Non-sequential alignments. BMC Bioinformatics 14, 24, doi: 10.1186/1471-2105-14-24 (2013).

[5] Minami S, Sawada K, Chikenji G. How a Spatial Arrangement of Secondary Structure Elements Is Dispersed in the Universe of Protein Folds. PLoS ONE 9(9): e107959. doi:10.1371/journal.pone.0107959 (2014)

[6] Kinnings, S. L. & Jackson, R. M. LigMatch: a multiple structure-based ligand matching method for 3D virtual screening. J Chem Inf Model 49, 2056–2066, doi: 10.1021/ci900204y (2009).

[7] OMEGA ver.2.4.3; Openeye Scientific Software, Inc.: Santa Fe, NM, 2010.

[8] Kinnings, S. L. & Jackson, R. M. LigMatch: a multiple structure-based ligand matching method for 3D virtual screening. J Chem Inf Model 49, 2056–2066, doi: 10.1021/ci900204y (2009).

Group 7

In recent years, deep learning, which is one of the machine learning techniques, has been widely studied, especially in fields of image recognition and speech recognition [1]. Deep learning methods have overcome classical methods in these fields. Even in the contest to predict molecular activity, "Merck Molecular Activity Challenge" held by Kaggle in 2012, the deep learning method overcame other methods [2]. From this kind of circumstance, we challenged second IPAB contest with deep learning. Figure 1 shows our prediction scheme.

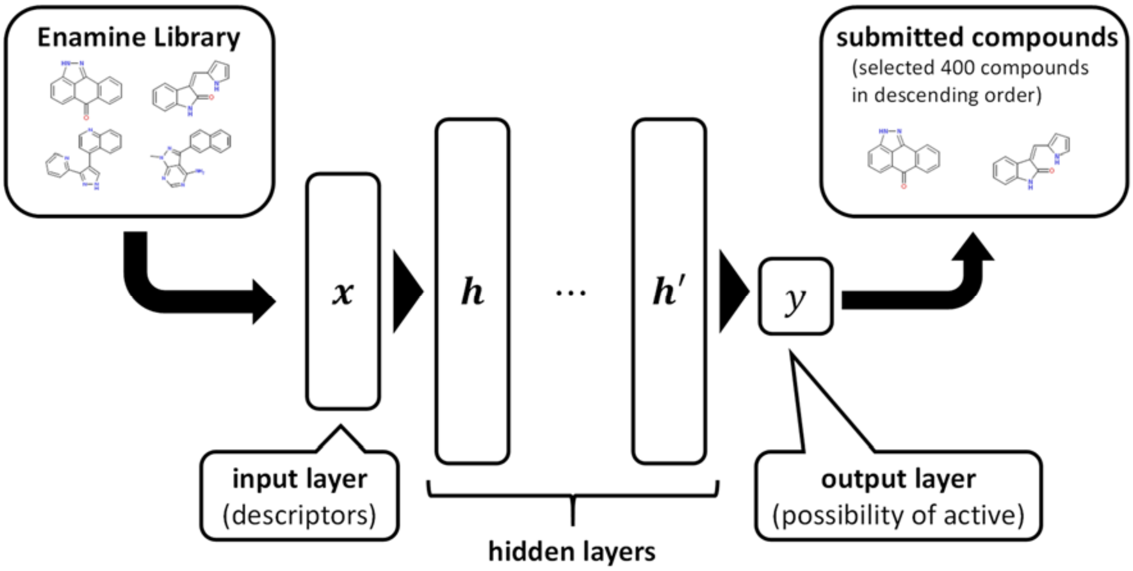

Figure 1: Our prediction scheme by deep learning.

Second IPAB contest provided 3,528 known Src family inhibitors registered in ChEMBL and BindingDB. We removed redundancy from these inhibitors and 3,465 inhibitors were left. We used those as positive samples of training data. Next, we collected inactive compounds from the result of first IPAB contest. In first IPAB contest, 600 compounds were tested in the primary and secondary assay and 593 compounds were reported as inactive. We used those as negative samples of training data (some of negative samples passed the primary assay but didn't pass the secondary assay in first IPAB contest). Therefore, we got 4,058 samples of training data totally. We calculated 590 physicochemical and topological descriptors by Canvas v2.2.013 (Schrödinger, LLC).

Deep neural network has an input layer $\boldsymbol{x}$, hidden layers $\boldsymbol{h}, \ldots, \boldsymbol{h}'$ and an output layer y. We trained a multilayer perceptron, which is one of the deep neural network models and each layer connects fully to the next one. We got initial weight of the multilayer perceptron by pre-training using Stacked Denoising Autoencoders [3]. We implemented prediction models with Pylearn2 [4].

There are many kinds of hyper-parameters to optimize for the multilayer perceptron (e.g. initial learning rate, number of hidden layers). For many hyper-parameters optimization, it's reported that random-search works well [5]. We sampled seven hyper-parameters repeatedly by random sampling

(described below) and used the combination of hyper-parameters achieving the best area under the receiver operating characteristic curve (ROC-AUC) for 5-fold cross-validation.

- an initial learning rate (log10-uniformly between 0.0001 and 1.0)
- a number of hidden layers (uniformly between 1 and 3)
- a number of hidden units (log10-uniformly between 128 and 4000)
- mini-batch size (log10-uniformly between 1 and 100)
- a number of epochs (log10-uniformly between 1 and 100)
- a time point at which to start annealing the learning rate (log10-uniformly between 1 and a number of epochs)
- an initial momentum (uniformly between 0 and 1)

Figure 2 shows our training scheme.

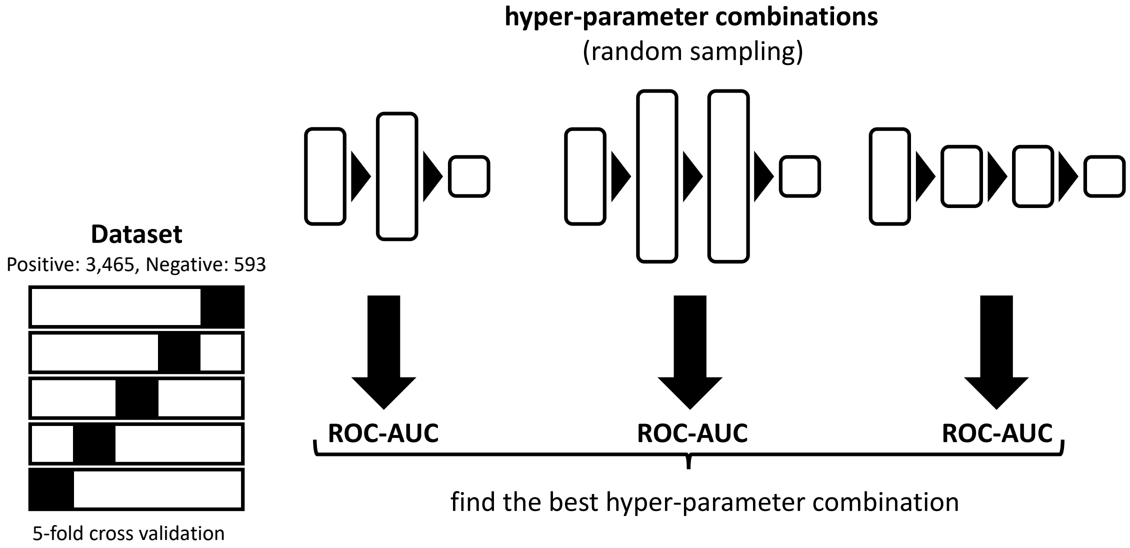

Figure 2: Our hyper-parameter optimization with random sampling.

Group 8

To predict inhibitors from ~2.4 million compounds in a library, a primitive version of PL-PatchSurfer2, a structure-based virtual screening method developed by us, was applied. The previous version of the program was published recently1.To examine the complementarity between protein and ligand, four features are implemented to describe molecular surface: shape, electrostatic potential, hydrophobicity, and hydrogen bonding. These characteristics are assigned on the molecular surface and further converted to three-dimensional Zernike descriptor to (3DZD). 3DZD is a series expansion of a 3D function in the Euclidean space and the coefficients of the series are transformed into one dimensional vector[2]. Adapting 3DZD descriptor has two advantages: 1) rotationally invariant and 2) fast to compare molecules.

To model the receptor structure, GalaxyTBM[3] was used. The Template-based model was built from the three templates found by HHPred[4]. The three-dimensional conformers of each ligand and its coordinates were generated by OMEGA[5]. Although PL-PatchSurfer2 uses 50 conformers for a ligand in an ideal case, to reduce the computational time, multiple ligand conformations could not be considered. Only the lowest energy conformation from OMEGA was used for screening. After generating structures of the protein and ligands, their surfaces were created and the 3DZD of the surface was calculated and segmented into patches. Patches from a binding pocket of a receptor and a ligand were then matched by an auction algorithm and the compatibility of the ligand to the receptor was computed. We submitted top 400 molecules as a putative c-Src kinase inhibitors.

It turned out that the result of our group was not good enough to find inhibitors for the target protein. One of the reasons is that we could not process nearly half of the library due to the time shortage, because we learned about the IPAB contest long after the starting date has passed. To save the time, the number of conformers for ligand was set to only one, but it is not optimal as we benchmarked earlier[6]. Also, many of the improvements of PL-PatchSurfer2 were undertaken after the IPAB contest period.

[1] Hu et al., PL-PatchSurfer: A Novel Molecular Local Surface-Based Method for Exploring Protein-Ligand Interactions. *Int. J. Mol. Sci.* **2014**, *15*, 15122-15145.

[2] Sael, L. and Kihara, D. Detecting local ligand-binding site similarity in nonhomologous proteins by surface patch comparison. *Proteins* **2012**, *80*, 1177-1185.

[3] Ko, J. et al., GalaxyTBM: template-based modeling by building a reliable core and refining unreliable local regions. *BMC Bioinformatics* **2012**, *13*, 198.

[4] Söding J. (2005) Protein homology detection by HMM-HMM comparison. *Bioinformatics* **2005**, *21*, 951-960.

[5] Hawkins, P. C. D. et al., Conformer Generation with OMEGA: Algorithm and Validation Using High Quality Structures from the Protein Databank and Cambridge Structural Database. *J.*

Chem. Info. Model. **2010**, *50*, 572-584.
[6] Shin, W.-H. et al., Three-Dimensional Compound Comparison Methods and Their Application in Drug Discovery. *Molecules* **2015**, *20*, 12841-12962.

Group 9

Compound Search by Docking to Allosteric Sites

The majority of previously known kinase inhibitors are type I, II, or III inhibitory compounds that strongly bind to the ATP-binding site. In this study, we conducted *in silico* compound screening to identify type IV inhibitory compounds that bind to the allosteric site and inhibit the enzyme activity without being affected by the substrate or ATP concentration.

In silico compound screening was performed according to the following scheme. Compounds in the designated library were docked to the allosteric site, and IDs of compounds with high docking scores were submitted. Unfortunately, we could not identify any compound exhibiting inhibitory activity in the present in vitro evaluation.

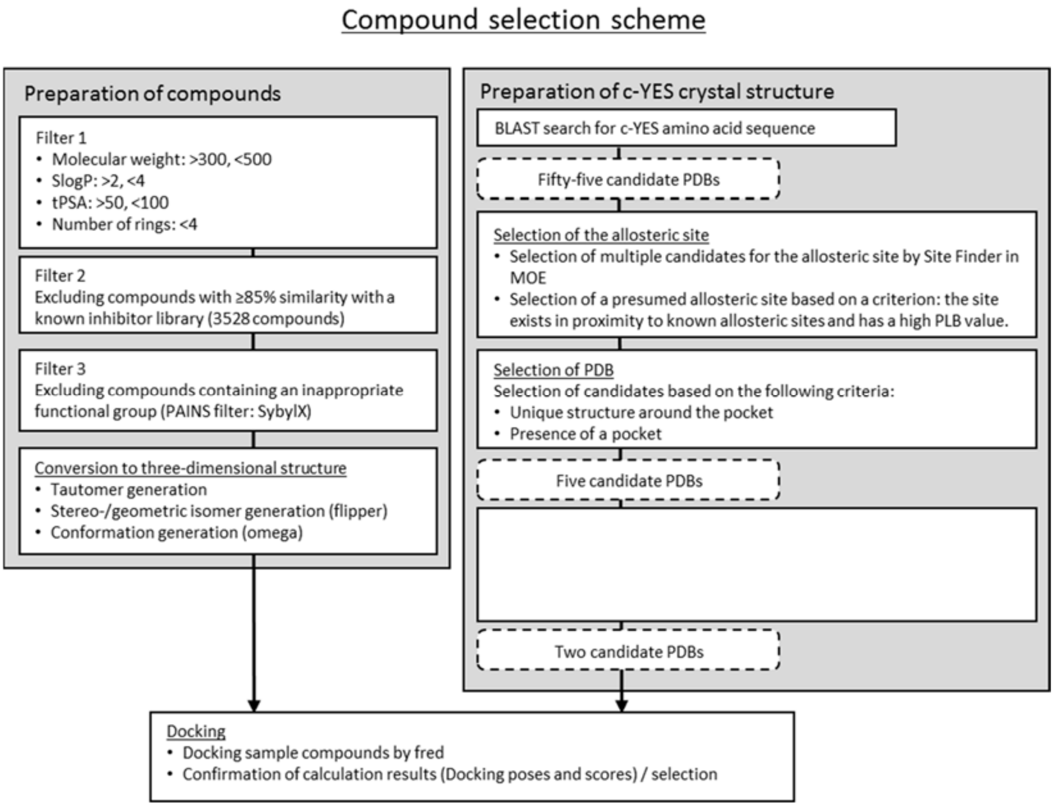

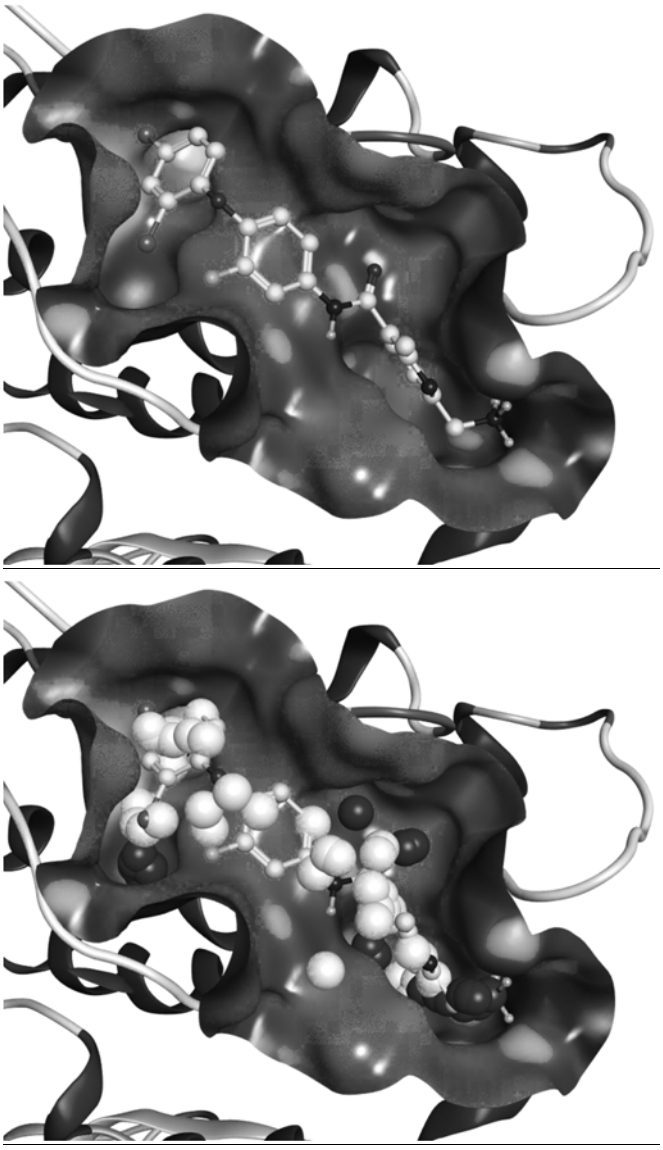

Figure. Putative binding mode of a representative selected compound to the allosteric site

Upper: The selected compound is shown with balls and sticks. The surface is an area around the presumed allosteric site of c-YES. Bottom: Simultaneous display of an alpha sphere detected by SiteFinder in MOE (Black: hydrophilic sphere, white: hydrophobic sphere)

Group 10

We participated in the past IPAB contest [11], using combination of ligand-based and structure-based approaches. Although no hit compounds were found in our proposal list in the past contest, we used essentially the same method again, with some changes in the Yes protein modeling process.

To perform structure-based molecular docking in a reasonable time, the compound database was roughly reduced by ligand-based enrichment. First, known potent compounds (List 1–3) were selected from literature [1–6], PubChem BioAssay [7, 8], and the past contest [11]. For each of those compounds in List 1 (potent compounds found in the literature and PubChem BioAssay), one conformer was generated using OMEGA (ver. 2.5.1.4, OpenEye) or directly downloaded from PubChem Compound, and then ROCS (ver. 3.2.0.4, OpenEye) search was performed against the ENAMINE database which was constructed using OMEGA with -maxconfs flag value 30. The default ROCS search yielded 500 compounds per query. For List 2 compounds (hit compounds in the past contest), similar compounds were retrieved from LigandBox chemical structure search engine [12, 13] (C-MCS mode at Tanimoto similarity threshold 0.4). List 3 was composed of compounds whose molecular weights were under 400 in List 1, to perform Docking Score Index (DSI) machine learning method of myPresto virtual screening suite [9]. The top 3000 compounds of the DSI output were chosen, of which about 1200 compounds were contained in the ENAMINE database.

In summary, we prepared the reduced database which was composed of i) the above-mentioned ROCS default output, which consisted of about ten thousand compounds in total (group I), ii) similar compounds of the hit compounds in the past contest (group II) and iii) compounds which were selected by DSI method (group III).

To build a structure model of Yes for docking simulation, the sequence of it was subjected to HHpred (http://toolkit.tuebingen.mpg.de/hhpred), and Src kinase in complex with a quinazoline inhibitor (PDB: 2H8H)[A] was suggested as the best template for homology modelling. Subsequently the structure of Yes was modeled by MODELLER implemented in the website. Residues from 1 to 94 of Yes did not match any protein structures registered in PDB, and no secondary structure was predicted in the residues by PSIPRED. Thus, residues from Ile95 to Leu543 were employed as an initial structure for MD simulations.

MD simulations were performed as follows. First, Saracatinib (CAS ID: 379231-04-6), which is

bound in Src kinase (PDB: 2H8H), was placed in the corresponding position of the modeled Yes to keep the binding site during subsequent MD simulations. RESP charge parameters of Saracatinib were obtained from the optimized structure at the level of B3LYP/6-31G(d) [B, C] with the ANTECHAMBER module of AmberTools 14 [D]. The Amber ff14 force field and GAFF were used for the protein and ligand, respectively, and the TIP3P model was used for the water molecules. The ε-nitrogen of His329 was assigned to be protonated, and δ-nitrogen of the other histidine residues 211, 239, 243, 249, 394, and 502 were protonated. The topology and initial coordinates were first generated by the LEaP module, and then converted into the Gromacs format by using ACPYPE (https://code.google.com/p/acpype/). Initial energy minimization, heating the system, and a 50-ns constant-NPT simulation were performed using GROMACS 5.0.4[E] as described in this report.[F] 10 snapshots were taken from the production run every 5 ns. For the following docking score calculation, the snapshot at 40 ns was chosen as the target structure for docking simulation.

Using the Yes structure generated by MD simulations, the active site was defined as a 12 Å radius sphere which encompassed the ATP pocket toward the P-loop. The docking scores of the compounds in the reduced database were calculated using MVD (GPU-screening mode), and the compounds were re-ranked according to the scores. The reranking was conducted for each compound group (groups 1–3), and the top-reranked compounds were chosen as potential hits by SBDD. In all, 195 compounds from group I, 10 from group II, and 195 from group III were included in the hit list and submitted to the contest committee.

According to the contest result, two compounds (Z410927360, Z1229984790) in our proposal list showed inhibitory activity against Yes. Z410927360 was group I (derived from the ROCS query of PCI32765) and Z1229984790 was group II (derived from similarity search of Z653349554). The only difference of our methods between the past contest and this time resided in target structure preparation. By comparing our newly modeled structure with the old one, we can see the docking pocket of the new structure is tighter than the old one and well-fitted to the ligand after MD simulation. In terms of LBDD-SBDD combination, it is suggested that preparation of appropriately tight docking pocket is influential in enhancing the screening efficiency.

List 1: Known potent compounds selected for LBDD screening.

PP2, SU6656, dasatinib, mc_4b, mc_25b, A-419259, KX2-391, PD166285, PCI 32765, NCGC00188382-02, NCGC00253463-01, ponatinib, SureCN431007, saracatinib, AMG-47a, CTK3C6843, CP-547632, AZD-7762, GW559768X, GW440139A, GW575808A, GW782612X, Kinome_3673, GW794607X, GW441806A, GW784752X.

List 2: Hit compounds in the past contest selected for LBDD screening.

Z1546616191 (Sunitinib), Z1546610485 (gefitinib), Z1024444840, Z653349554.

List 3: Compounds selected for myPresto DSI machine learning.

CTK3C6843, GW559768X, SU6656, AZD7762, GW440139A, Kinome_3673, GW575808A.

References

[A]

1. Hirsch, A. J., Medigeshi, G. R., Meyers, H. L., DeFilippis, V., Früh, K., Briese, T., ... & Nelson, J. A. (2005). The Src family kinase c-Yes is required for maturation of West Nile virus particles. Journal of virology, 79(18), 11943-11951.
2. Georghiou, G., Kleiner, R. E., Pulkoski-Gross, M., Liu, D. R., & Seeliger, M. A. (2012). Highly specific, bisubstrate-competitive Src inhibitors from DNA-templated macrocycles. Nature chemical biology, 8(4), 366-374.
3. Yeung, C. L., Ngo, V. N., Grohar, P. J., Arnaldez, F. I., Asante, A., Wan, X., ... & Helman, L. J. (2013). Loss-of-function screen in rhabdomyosarcoma identifies CRKL-YES as a critical signal for tumor growth. Oncogene, 32(47), 5429-38.
4. Zhang, X., Meyn III, M. A., & Smithgall, T. E. (2013). c-Yes tyrosine kinase is a potent suppressor of ES cell differentiation and antagonizes the actions of its closest phylogenetic relative, c-Src. ACS chemical biology, 9(1), 139-146.
5. Anbalagan, M., Carrier, L., Glodowski, S., Hangauer, D., Shan, B., & Rowan, B. G. (2012). KX-01, a novel Src kinase inhibitor directed toward the peptide substrate site, synergizes with tamoxifen in estrogen receptor α positive breast cancer. Breast cancer research and treatment, 132(2), 391-409.
6. Blake, R. A., Broome, M. A., Liu, X., Wu, J., Gishizky, M., Sun, L., & Courtneidge, S. A. (2000). SU6656, a selective src family kinase inhibitor, used to probe growth factor signaling. Molecular and cellular biology, 20(23), 9018-9027.

7. Patel, P. R., Sun, H., Li, S. Q., Shen, M., Khan, J., Thomas, C. J., & Davis, M. I. (2013). Identification of potent Yes1 kinase inhibitors using a library screening approach. Bioorganic & medicinal chemistry letters, 23(15), 4398-4403.

8. qHTS for small molecule inhibitors of Yes1 kinase. http://pubchem.ncbi.nlm.nih.gov/AID686946

9. myPresto http://presto.protein.osaka-u.ac.jp/myPresto4/

10. Omagari, K., Mitomo, D., Kubota, S., Nakamura, H., & Fukunishi, Y. (2008). A method to enhance the hit ratio by a combination of structure-based drug screening and ligand-based screening. Advances and applications in bioinformatics and chemistry: AABC, 1, 19.

11. Chiba, S., Ikeda, K., Ishida, T., Gromiha, M. M., Taguchi, Y., Iwadate, M., ... & Sugaya, N. (2015). Identification of potential inhibitors based on compound proposal contest: Tyrosine-protein kinase Yes as a target. Scientific reports, 5: 17209.

12. Kawabata, T. (2011). Build-up algorithm for atomic correspondence between chemical structures. Journal of chemical information and modeling, 51(8), 1775-1787.

13. LigandBox: Chemical Structure Search http://ligandbox.protein.osaka-u.ac.jp/ligandbox/

A. Hennequin, L. F.; Allen, J.; Breed, J.; Curwen, J.; Fennell, M.; Green, T. P.; Brempt, C. L.-v. d.; Morgentin, R.; Norman, R. A.; Olivier, A.; Otterbein, L.; Ple, P. A.; Warin, N.; Costello, G., N-(5-Chloro-1,3-benzodioxol-4-yl)-7-[2-(4-methylpiperazin-1-yl)ethoxy]-5-(tetrahydro-2H-pyran-4-yloxy)quinazolin-4-amine, a novel, highly selective, orally available, dual-specific c-Src/Abl kinase inhibitor. Journal of Medicinal Chemistry 2006, 49 (22), 6465–6488.

[B] Becke, A. D., Density-functional exchange-energy approximation with correct asymptotic behavior. Physical Review A 1988, 38 (6), 3098–3100

[C] Lee, C. T.; Yang, W. T.; Parr, R. G., Development of the Colle-Salvetti correlation-energy formula into a functional of the electron density. Physical Review B 1988, 37 (2), 785–789.

[D] Case, D. A.; Babin, V.; Berryman, J. T.; Betz, R. M.; Cai, Q.; Cerutti, D. S.; Cheatham III, T. E.; Darden, T. A.; Duke, R. E.; Gohlke, H.; Goetz, A. W.; Gusarov, S.; Homeyer, N.; Janowski, P.; Kaus, J.; Kolossváry, I.; Kovalenko, A.; Lee, T. S.; LeGrand, S.; Luchko, T.; Luo, R.; Madej, B.; Merz, K. M.; Paesani, F.; Roe, D. R.; Roitberg, A.; Sagui, C.; Salomon-Ferrer, R.; Seabra, G.; Simmerling, C. L.; Smith, W.; Swails, J.; Walker, R. C.; Wang, J.; Wolf, R. M.; Wu, X.; Kollman, P. A., AMBER 14. University of California, San Francisco. 2014.

[E] Abrahama, M. J.; Murtola, T.; Schulz, R.; Páll, S.; Smith, J. C.; Hess, B.; Lindahl, E., GROMACS: High performance molecular simulations through multi-level parallelism from laptops to supercomputers. SoftwareX 2015, 1–2, 19–25.

[F] Moriwaki, Y.; Terada, T.; Caaveiro, J. M. M.; Takaoka, Y.; Hamachi, I.; Tsumoto, K.; Shimizu, K., Heme binding mechanism of structurally similar iron-regulated surface determinant near transporter domains of Staphylococcus aureus exhibiting different affinities for heme.

Biochemistry 2013, 52 (49), 8866–8877.
[G] HHpred http://toolkit.tuebingen.mpg.de/hhpred

Group 11

Homology model and structure selection

To obtain target protein structure for docking simulation, we carried out homology modeling using Prime[1, 2]. First, we downloaded 1Y57[3], 2SCR[4] and 3G5D[5] as template structure, and performed homology modeling. We conducted decoy docking using 30 known active compounds and 200 negative compounds, and evaluated docking results by Wilcoxon signed-rank test and Bonferroni correction. Active compounds were obtained from previous IPAB contest and ChEMBL, and decoy compounds were obtained from previous IPAB contest. 2 poses of each compound were used in the evaluation. From three structures, model 1Y57 and model 3G5D could significantly separate active compounds and decoy compounds (p-value = 2.66×10^{-3}, 2.39×10^{-4}, respectively). We chose model 1Y57 because we failed to find hit compounds using model 3G5D in the previous contest.

Virtual screening

Tertiary structures of labeled compounds and compounds in the target library were generated using Ligprep. Docking simulations were performed using HTVS mode of Glide version 6.5[6, 7]. 2 poses of each compound were used in the evaluation.

CBM-Score

Final ranking was determined by our original scoring method. Conserved Binding Mode (CBM)-Score applied to top 50 thousand compounds of the docking score, which corresponds to top 2%. CBM-Score was calculated by the similarity of the interaction energy through docking simulation. First, interaction energy between a compound and each residue of protein were extracted from docking simulation. Second, these vectors are converted to 3 dimensional vectors using principal component analysis (PCA). Third, each labeled compound give score s to near 100 compounds. In this screening, s=7 for active compounds and s=-1 for decoy compounds. The final CBM-Score of a compound was the summation of the scores given to the compound.

Results and discussions

We proposed 400 compounds and 181 compounds of them were assayed. 8 compounds passed the primary assay and 1 compound passed the validation assay.

The advantages of this method were two points. One is that the decoy docking could able to find a suitable protein structure for virtual screening. Though the labeled compounds are not enough, this method can separate active compounds from decoy compounds. The other is that CBM-Score might be able to exclude false positive compounds in the docking simulation by using the information of labeled compounds.

References

[1] Jacobson, M. P., Friesner, R.A., *et al*., *J. Mol. Biol*., 320, 597-608 (2002).

[2] Jacobson M. P., Pincus, D. L., *et al*., *Proteins*, 55, 351-367 (2004).

[3] Cowan-Jacob S.W., Fendrich G., *et al., Structure*, 13, 861-871 (2005).

[4] Xu W., Doshi A., *et al., Mol.Cell,* 3, 629-638 (1999).

[5] Getlik M., Grutter C., *et al., J.Med.Chem.,* 52, 3915-3926 (2009).

[6] Friesner, R. A., Banks, J. L., *et al*., *J. Med. Chem*., 47, 1739–1749 (2004).

[7] Halgren, T. A., Murphy, R. B., *et al*., *J. Med. Chem*., 47, 1750–1759 (2004).
